# Comprehensive Transcriptome Profiles of *Streptococcus mutans* UA159 Map Core Streptococcal Competence Genes

**DOI:** 10.1128/mSystems.00038-15

**Published:** 2016-04-12

**Authors:** R. Khan, H. V. Rukke, H. Høvik, H. A. Åmdal, T. Chen, D. A. Morrison, F. C. Petersen

**Affiliations:** a Department of Oral Biology, Faculty of Dentistry, University of Oslo, Oslo, Norway; b The Forsyth Institute, Cambridge, Massachusetts, USA; c Department of Biological Sciences, University of Illinois at Chicago, Chicago, Illinois, USA; University of Hawaii

**Keywords:** CSP, *Streptococcus*, XIP, genetic competence, natural transformation systems, pheromone, quorum sensing

## Abstract

In *Streptococcus mutans*, an oral colonizer associated with dental caries, development of competence for natural genetic transformation is triggered by either of two types of peptide pheromones, competence-stimulating peptides (CSPs) (18 amino acids [aa]) or SigX-inducing peptides (XIPs) (7 aa). Competence induced by CSP is a late response to the pheromone that requires the response regulator ComE and the XIP-encoding gene *comS*. XIP binds to ComR to allow expression of the alternative sigma factor SigX and the effector genes it controls. While these regulatory links are established, the precise set of effectors controlled by each regulator is poorly defined. To improve the definition of all three regulons, we used a high-resolution tiling array to map global changes in gene expression in the early and late phases of the CSP response. The early phase of the CSP response was limited to increased gene expression at four loci associated with bacteriocin production and immunity. In the late phase, upregulated regions expanded to a total of 29 loci, including *comS* and genes required for DNA uptake and recombination. The results indicate that the entire late response to CSP depends on the expression of *comS* and that the immediate transcriptional response to CSP, mediated by ComE, is restricted to just four bacteriocin-related loci. Comparison of the new data with published transcriptome data permitted the identification of all of the operons in each regulon: 4 for ComE, 2 for ComR, and 21 for SigX. Finally, a core set of 27 panstreptococcal competence genes was identified within the SigX regulon by comparison of transcriptome data from diverse streptococcal species.

**IMPORTANCE** *S. mutans* has the hard surfaces of the oral cavity as its natural habitat, where it depends on its ability to form biofilms in order to survive. The comprehensive identification of *S. mutans* regulons activated in response to peptide pheromones provides an important basis for understanding how *S. mutans* can transition from individual to social behavior. Our study placed 27 of the 29 transcripts activated during competence within three major regulons and revealed a core set of 27 panstreptococcal competence-activated genes within the SigX regulon.

## Abstract (precis)

*S. mutans* has the hard surfaces of the oral cavity as its natural habitat, where it depends on its ability to form biofilms in order to survive. The comprehensive identification of *S. mutans* regulons activated in response to peptide pheromones provides an important basis for understanding how *S. mutans* can transition from individual to social behavior. Our study placed 27 of the 29 transcripts activated during competence within three major regulons and revealed a core set of 27 panstreptococcal competence-activated genes within the SigX regulon.

## INTRODUCTION

The acquisition of new genes through horizontal transfer among the prokaryotes plays an important role in ecological diversification and adaptation (1). The first evidence of such horizontal gene transfer was the recognition that virulence determinants can be transferred between pneumococci in infected mice, a phenomenon defined as natural transformation (2). Natural genetic transformation refers to the active uptake of exogenous DNA, followed by heritable incorporation of its genetic information, a capacity that is widespread but not universal in both Gram-positive and Gram-negative bacteria and in the archaea (3). In the Gram-positive genus *Streptococcus*, some members of the *Streptococcus mitis*, *S. anginosus*, *S. salivarius*, and *S. mutans* groups are recognized as naturally transformable (4–6). Recently, competence was also reported in *S. suis* and members of the *S. bovis* group (7, 8). In the remaining members of the genus, the presence of regulatory and effector homologs of proteins involved in competence for natural transformation indicates that competence development may be a general trait of streptococci (9).

Competence in streptococci is often expressed as a transient developmental state in which bacteria exhibit a capacity for natural genetic transformation (10). The competent state is triggered by autoinducing peptide pheromones, leading to increased expression of the alternative sigma factor SigX (also known as ComX), which is the master regulator of competence (11, 12). The genes differentially expressed during competence, reported as corresponding to 6% or more of the genome (13–15), include genes required for DNA uptake and recombination and genes required to scavenge DNA by killing other bacteria without causing self-damage. Some of the competence-specific genes are, however, not directly involved in these processes, indicating that the system may have evolved to control additional functions, such as adaptation to acid stress, biofilm formation, and virulence (16–20).

The streptococcal competence-inducing pheromones are unmodified linear peptides produced as propeptides (21). Competence development is coordinated within a culture by a positive feedback loop linking pheromone production to the external concentration of the secreted mature peptide. The competence-stimulating peptides (CSPs), which belong to the double-glycine family of peptides (22), are sensed on the outside of cells upon binding to the ComD histidine kinase of the ComED two-component signal transduction system (TCSTS) (23, 24), whereas SigX-inducing peptides (XIPs) are sensed by ComR intracellular regulators of the Rgg family, upon internalization by the oligopeptide permease complex Opp (5, 6). The propeptides belong to either the double-glycine CSP family, as in the *S. mitis* group, or to a distinct class of peptides associated with Rgg regulators, as in the *S. salivarius*, *S. mutans*, pyogenic, and *S. bovis* groups (25).

**FIG 1:**
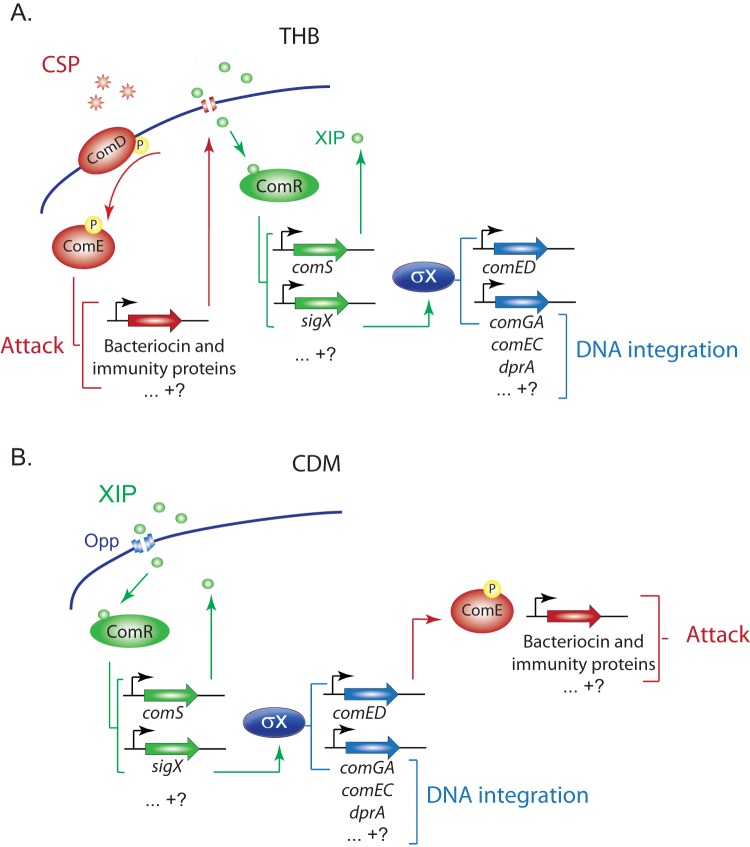
Competence regulatory networks in *S. mutans*. Regulatory links are organized into two pheromone response networks acting in peptide-rich media such as THB (A) or in peptide-free CDM (B). (A) In rich media, extracellular CSP induces the expression of bacteriocin and immunity proteins (red) through the ComED TCSTS pathway. Other genes are possibly induced by direct or indirect activation by phosphorylated ComE. The bacteriocins are thought to create pores that allow internalization of the competence pheromone XIP. XIP binds to ComR to activate the expression of at least two genes (green), *comS* (encoding XIP) and *sigX* (encoding the alternative sigma factor σ^x^). Induction of *comS* creates a positive feedback loop that increases the production of XIP and σ^x^, while σ^x^ activates the expression of genes (blue) involved in DNA integration (e.g., *comGA*, *comEC*, and *dprA*). (B) In peptide-free CDM, extracellular XIP is internalized via the Opp permease and binds to ComR, activating the expression of at least two genes, *comS* (encoding XIP) and *sigX*. Induction of *comS* creates a positive feedback loop, while elevated σ^x^ activates the expression of genes involved in DNA integration (e.g., *comGA*, *comEC*, and *dprA*). Bacteriocin and immunity proteins are upregulated as a result of *comED* induction by σ^x^. Uncertainties about the regulon assignment for the full range of genes that change expression in response to the pheromones are represented by question marks, and genes upregulated but not exemplified are represented by ellipses.

In *S. mutans*, a human oral colonizer associated with dental caries, the competence regulatory network is somewhat more complex than the analogous networks in other streptococci. While the *S. mutans* network shares with them the alternative sigma factor SigX, it employs two peptide pheromones, not one, in upstream circuits for coordination of entry into the competent state. Furthermore, its regulatory behavior in rich media differs from that in chemically defined media (CDM). Although the reasons for such differences remain unclear, it is possible that the lack of peptides in the CDM used in different studies may be a relevant factor, since addition of assorted peptides to CDM eliminates the activity of XIP (26). Each peptide pheromone circuit in *S. mutans* encompasses genes for peptide synthesis, processing, and secretion, a peptide receptor regulating the transcription of additional genes, and a set of *cis*-acting sites targeted by the pheromone receptor to create a peptide-specific regulon (Fig. 1). In recent years, the links between these regulons have emerged as a set of reasonably well-defined interactions organized in a different order during competence development in the two classes of culture media (Fig. 1).

Assignment of genes to the ComE, ComR, and SigX regulons has been supported by analysis of shared sequence motifs at *cis*-acting sites and three types of evidence showing that (i) gene expression depends on a nearby regulon-specific promoter site, (ii) gene expression depends on the cognate regulator, or (iii) elevated expression of the gene can be driven by overexpression of the regulator. The supporting experimental evidence focused principally on a limited subset of induced genes (see Table S1 in the supplemental material). Despite these extensive studies, some of the links drawn in Fig. 1 are still incompletely understood. For example, the suggestion that CipB potentiates XIP by creating pores in the membrane, allowing XIP internalization in Todd-Hewitt broth (THB) (but not CDM) (27) has not been directly tested. It also remains unknown whether CSP is the signal that binds to ComD to promote ComE phosphorylation and activation in CDM (26, 27). More broadly, it is unclear exactly which genes are in each regulon or how numerous additional inputs to competence regulation are effected.

10.1128/mSystems.00038-15.1 Table S1  Overview of *S. mutans* genes upregulated by competence-inducing pheromones and their expression in deletion mutants. Download Table S1, PDF file, 0.3 MB. Copyright © 2016 Khan et al. 2016 Khan et al. This content is distributed under the terms of the Creative Commons Attribution 4.0 International license.

With the broad pattern of these pathways of signal transduction outlined, it is a suitable time to refine the definition of the network by identifying genes and operons in each of the three known regulons more comprehensively, as well as by asking whether the genes that are upregulated specifically in competent cells are restricted to these three regulons. We report here six new transcriptome data sets obtained by the use of an improved tiling microarray that clearly distinguishes, on a genomic scale, the early responses to CSP from the late responses triggered by XIP. Detailed mapping of the transcriptomes allowed the comprehensive prediction or confirmation of start sites for the induced transcripts, enabling us to refine the assignment of transcripts to specific regulons and to identify new regulon members. The results also provide experimental evidence supporting early suggestions that upregulation of genes distal to competence regulator recognition sites often arises from readthrough past transcriptional terminators, accounting, in large part, for the wide intra- and interspecies variations in the number of genes assigned to the competence regulons. Finally, comparison of these data sets with existing competence transcriptome profiles in several other streptococcal species reveals a core set of streptococcal competence-specific genes.

## RESULTS

### Probe design and sampling strategy.

Published transcriptome surveys provide valuable views into the breadth of streptococcal competence-specific genes; however, for most species, including the competence model *S. pneumoniae*, transcriptome data for competence development have come mostly from early techniques of microarray analysis that have technical limitations, such as the use of probes that are often restricted to annotated open reading frames (ORFs), the use of probes with low spatial resolution, and the use of cDNA preparations that result in artifactual antisense signals. In *S. mutans*, newer high-density tiling arrays and RNA-sequencing methods have already been used in transcriptome surveys of the competence response, but none of them was designed specifically to distinguish the different regulons that are activated in response to CSP. The tiling array study was restricted to responses resulting from long exposure to CSP and lacked full coverage, including probes for the *comS* gene (28), whereas in the RNA-sequencing study, short exposure to CSP was investigated in a medium that does not support activation of competence by CSP (27). Therefore, to capture a more complete picture of competence regulation in *S. mutans*, we chose a strategy of probe design and sample collection and preparation that would allow comprehensive mapping of transcripts in both the early and late phases of the CSP response under conditions in which CSP induces competence.

We employed six strategies to minimize confounding errors known to affect transcriptional profiling. (i) To improve the specificity of hybridization signals, we employed 385,000 overlapping 50-mer oligonucleotide tiling probes optimized for uniqueness, *T_m_*, and probe length, with a resolution of approximately 10 bp, as described previously (29). (ii) To minimize the artifactual “antisense” signals that arise during cDNA preparation, RNA was instead directly chemically labeled (30). (iii) To maximize mRNA signals, the RNA was depleted of rRNA. (iv) To minimize loss of sRNAs and possible short transcripts coding for small peptides such as ComS and ComC, microRNA purification protocols were employed. (v) To minimize signals from irrelevant metabolic changes that might occur during the experiment, we limited cultures to the early log phase. (vi) Finally, we chose to use the mature CSP18 pheromone (31), instead of the precursor CSP-21 peptide used in previous transcriptome surveys (15, 28, 32), in order to minimize any response delay due to peptide processing steps (31, 33, 34).

**FIG 2:**
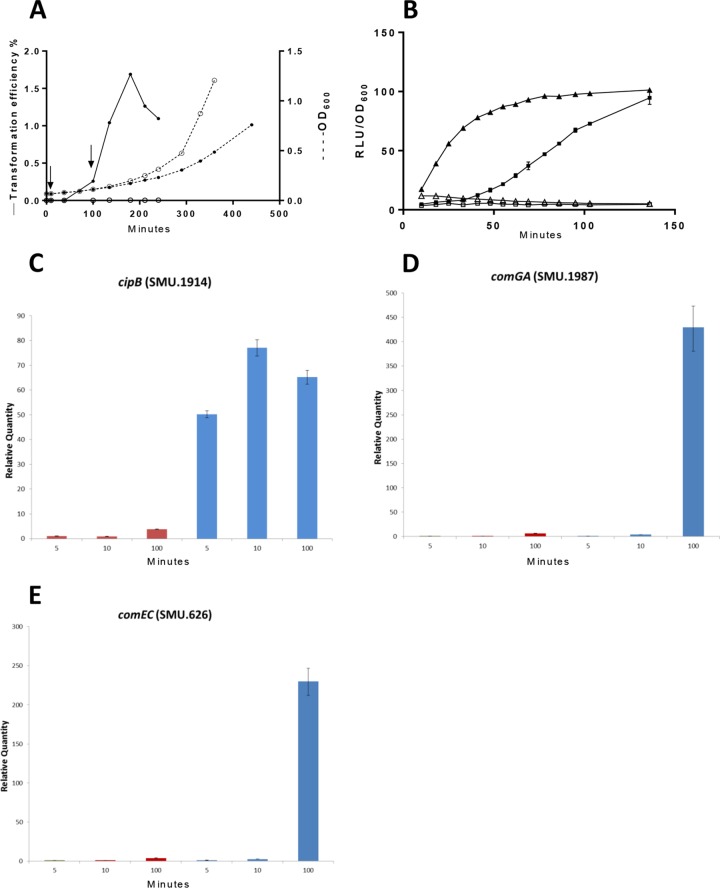
Effect of CSP18 on induction of competence and expression of *cipB*, *sigX*, *comGA*, and *comEC* during growth in TSB. (A) During growth of *S. mutans* UA159 in the presence (●) or absence (○) of CSP, transformation efficiency (solid line) and OD_600_ (broken line) were determined at the times indicated. Exposure to pVA838 was for 20 min and was followed by further incubation with DNase I for 40 min before plating. Arrows show the times of sample harvesting for microarray analysis. (B) Luciferase reporters were used in parallel cultures to measure the effect of CSP on the expression of *cipB* (*nlmC*, SMU.1914) (triangles) and *sigX* (squares). The strains were grown in the presence of 50 nM CSP (filled symbols) or without CSP (empty symbols). CSP was added at *t* = 0. Relative light units (RLU) and OD_600_ were measured in a 96-well plate with a multidetection microplate reader (SynergyHT; BioTek). Expression levels are relative to the highest value of each reporter (100%). Error bars indicate standard deviations of triplicate assays. (C to E) Relative expression of selected early responsive gene *cipB* (C) and late responsive genes *comGA* and *comEC* (D and E). Real-time PCR data were normalized to the expression values of the respective genes in the WT strain at 5 min without CSP. Mean values and standard errors for three replicates at 5, 10, and 100 min without CSP (red bars) and with CSP (blue bars) are shown.

RNA preparations were made from cultures of the wild type (WT) in the early and late stages of the response to CSP and from cultures of a *comS* mutant in the late stage. The most appropriate times to distinguish the early and late global responses during competence development were selected on the basis of measurements of growth, DNA incorporation dynamics, and expression of selected early and late genes after CSP supplementation of cultures in tryptone soya broth (TSB) (Fig. 2). TSB was chosen because this medium supports competence development stimulated by synthetic CSP, but endogenous competence development is absent or restricted to low levels and is independent of *comC* (35). During the first 120 min of incubation, cultures with and without CSP grew at equal rates and remained far from stationary phase. The culture treated with CSP became capable of rapid DNA uptake (Fig. 2A), while competence remained very low in the parallel culture without added CSP throughout 4 h. Transformation was first detected at 100 min after CSP addition, reached maximal levels at 3 h, and declined by 4 h. Using a gene fusion reporter, we found that *cipB* expression, used as an indicator of early gene expression, was strongly dependent on CSP and increased as an immediate response, replicating previous findings (33, 34). In contrast, expression of *sigX* (also CSP dependent) began only after a delay of approximately 50 min (Fig. 2B). Thus, (i) the strong dependence of both the early expression of bacteriocin genes and the late development of competence on CSP in TSB and (ii) the time difference between the two responses established optimal conditions for study of the temporally specific effects of CSP on the transcriptome. Because endogenous competence induction was absent within the first 3 h and CSP-induced DNA incorporation was robust by approximately 2 h, at a time when growth had not been inhibited by CSP, we further evaluated the use of CSP exposure times of 10 and 100 min. Reverse transcription (RT)-PCR assays confirmed that the early and late transcriptional responses were activated at 10 and 100 min, respectively (Fig. 2C to E). Expression of the ComE-regulated gene SMU.1914 (*cipB*, *nlmC*) had already increased dramatically at 10 min compared with that in the culture without CSP, but there was only a slight increase in the expression of the SigX-dependent genes *comGA* and *comEC*. By 100 min, *comGA* and *comEC* expression had increased by more than 200-fold, while expression of SMU.1914 continued at an elevated level. Thus, 10 and 100 min were selected as the earliest suitable sampling times for studying the immediate and delayed transcriptional responses to CSP in the absence of any gross effect of CSP on the growth rate.

RNA was extracted from cultures under the six conditions selected (UA159 for 10 or 100 min and the *comS* mutant for 100 min, with and without CSP) in duplicate experiments and analyzed for strand-specific genome-wide gene expression as described in Materials and Methods. Inspection of the resulting profiles gives several indications that the sampling strategy yielded expression patterns of significantly improved quality. Using directly labeled RNA to exclude the artifactual antisense signals often observed with conventional cDNA preparations offered clear benefits, as it revealed that several of the genes previously classified as upregulated in response to CSP were indeed upregulated, but only in the antisense direction. We also observed mRNAs of short length in our preparations, including that for the CSP-encoding gene *comC* (approximately 190 bp), indicating successful isolation of short transcripts. Moreover, by carefully matching the CSP-treated and untreated samples in relation to the incubation time and culture density, we could clearly distinguish changes specific to CSP exposure from nonspecific changes occurring during growth. We found that in the control samples without CSP, the expression of 98 genes differed between cultures grown for 10 min and those grown for 100 min (see Table S2 in the supplemental material). None of the genes required for competence, such as those for DNA uptake and recombination, were identified in this group, indicating that the changes were not competence related. To determine whether this information would contribute to a better definition of the overall response to the CSP pheromone, we investigated whether genes previously defined as CSP induced include the group of genes identified here as nonspecific to the CSP response. In fact, 26 of the genes showing changes associated with growth were among genes previously defined as part of the CSP response (see Table S2) (15).

10.1128/mSystems.00038-15.2 Table S2  Gene expression dynamics during culture growth. Download Table S2, PDF file, 0.4 MB. Copyright © 2016 Khan et al. 2016 Khan et al. This content is distributed under the terms of the Creative Commons Attribution 4.0 International license.

Among the 1,961 protein-encoding genes annotated in the UA159 genome sequence, a subset of 83 were represented by a >2-fold expression increase during a 100-min CSP exposure (Table 1; see Table S3 in the supplemental material). Only five genes exhibited downregulation, but all in the low range between −2.1- and −2.3-fold changes. The CSP induction ratios were, in general, similar to those previously observed in a transcriptome survey examining a competent subfraction of the *S. mutans* population (28) and higher than in surveys using mixed populations exposed to CSP for 120 min (see Fig. S1 in the supplemental material) (15, 28). Approximately 160 genes that were reported as differentially regulated in response to CSP in one of the mixed-population studies (15) were not confirmed in either this study or in the study using sorted competent cells (see Fig. S1B and Table S4 in the supplemental material). We conclude that such genes are unlikely to be reproducible parts of the late CSP response and may represent experimental error, indirect effects of competence, or metabolic changes unrelated to the response to CSP. This interpretation is strengthened by the observation that the induction levels of most genes were uniformly higher in the present data and in the previous study using sorted cells and by the fact that the former transcriptome did not provide information on direction of transcripts and had a limited set of probes for each of the ORFs. Finally, the induced genes encoding bacteriocin and bacteriocin immunity proteins were generally induced at higher ratios here than in the previous studies using mixed or sorted populations of CSP-stimulated cells. This was particularly valuable in view of our aim to differentiate early and late responses associated with the activation of distinct regulons during the CSP response.

10.1128/mSystems.00038-15.3 Table S3  Effect of CSP on gene expression (ORFs). Download Table S3, PDF file, 1.9 MB. Copyright © 2016 Khan et al. 2016 Khan et al. This content is distributed under the terms of the Creative Commons Attribution 4.0 International license.

10.1128/mSystems.00038-15.4 Table S4  Comparison of transcriptome results from four studies. Download Table S4, PDF file, 0.6 MB. Copyright © 2016 Khan et al. 2016 Khan et al. This content is distributed under the terms of the Creative Commons Attribution 4.0 International license.

10.1128/mSystems.00038-15.8 Figure S1  Correlations between gene expression changes induced by CSP in this study and in previous transcriptome studies. Download Figure S1, PDF file, 0.4 MB. Copyright © 2016 Khan et al. 2016 Khan et al. This content is distributed under the terms of the Creative Commons Attribution 4.0 International license.

**TABLE 1:** Global temporal profile of responses to CSP in TSB^a^

Gene ID^b^	Mean fold change^c^			Annotation^d^
	UA159		Δ*comS* mutant, 100 min	
	10 min	100 min		
Early response				
SMU.150	51.8	62.4	40.9	Hypothetical protein, NlmA, mutacin IV A
SMU.151	34.7	41.1	46.2	Hypothetical protein, NlmB, mutacin IV B
SMU.152	22.4	36.4	42.0	Hypothetical protein, mutacin IV immunity
SMU.153	17.6	25.9	25.6	Hypothetical protein
SMU.423	38.8	49.5	54.3	Hypothetical protein, NlmD, mutacin VI
SMU.424	3.1	5.9	3.1	CopY
SMU.426	2.9	5.5	3.6	Copper-transporting ATPase, CopA
SMU.427	2.7	4	2.3	Putative copper chaperone, CopZ
SMU.925	3.8	10.3	6.4	Hypothetical protein, ImmB, immunity protein
SMU.1902c	2.1	2.5	1.4	Hypothetical protein, putative bacteriocin, BsmK
SMU.1903c	12.4	16	24.3	Hypothetical protein
SMU.1904c	21.1	49.7	61.1	Hypothetical protein
SMU.1905c	26.4	47.8	43.8	Putative bacteriocin secretion protein, BsmL
SMU.1906c	21.3	34	45.1	Hypothetical protein, BsmB
SMU.1908c	16.4	57.1	35.8	Hypothetical protein
SMU.1909c	17.6	55.1	85.3	Hypothetical protein, immunity protein
SMU.1910c	15.5	39.2	61.6	Hypothetical protein
SMU.1912c	16.4	29.9	67.0	Hypothetical protein
SMU.1913c	13.6	29.3	49.4	Putative immunity protein, BlpL like, ImmA
SMU.1914c	15.9	17.4	31.0	Hypothetical protein, NlmC, CipB, mutacin V
Late response				
* comS*	1.7	12.7		ComS, pheromone
SMU.64	1.2	5.6	−1.2	Holliday junction DNA helicase RuvB
SMU.65	1.2	4.4	−1.1	Putative protein tyrosine phosphatase
SMU.66	1.1	2.6	−1.2	Hypothetical protein
SMU.67	−1.0	2.3	−1.1	Putative acyltransferase
SMU.68	1.5	2.3	−1.2	Hypothetical protein
SMU.109	−1.0	2.2	−1.0	Permease (efflux protein)
SMU.166	−1.1	3.7	−1.2	Hypothetical protein
SMU.167	−1.1	2.6	−1.2	Hypothetical protein
SMU.168	1.0	3.5	−1.5	Putative transcriptional regulator
SMU.325	1.3	3	−1.3	Deoxyuridine 5′-triphosphate nucleotidohydrolase
SMU.326	1.1	3.3	−1.3	Hypothetical protein
SMU.327	1.3	3.1	−1.2	DNA repair protein RadA
SMU.352	1.2	6	1.0	Ribulose-phosphate 3-epimerase
SMU.353	1.1	5.6	1.1	Hypothetical protein
SMU.354	1.1	5.4	1.5	Hypothetical protein, Ccs50
SMU.355	1.2	5.9	1.2	Putative CMP-binding factor, CBF1
SMU.356	1.0	3.2	−1.0	*pur* operon repressor
SMU.498	1.6	64.8	−1.0	Putative late competence protein, ComFA
SMU.499	1.3	40.5	−1.0	Putative late competence protein, ComFC
SMU.500	−1.4	3.2	1.1	Putative ribosome-associated protein, YflA
SMU.505	1.1	14.4	1.4	Putative adenine-specific DNA methylase
SMU.506	1.0	5.6	1.2	Putative type II restriction endonuclease
SMU.507	−1.2	4.9	−1.3	DeoR family transcriptional regulator
SMU.508	−1.0	4.9	−1.2	Hypothetical protein
SMU.539c	1.0	8.7	1.2	Signal peptidase type IV, CilC
SMU.625	1.8	131.7	−1.2	Putative competence protein, CilE, DelA, ComEA
SMU.626	1.5	61.7	1.1	Putative competence protein, CelB, ComEC
SMU.627	−1.1	3.3	1.0	Hypothetical protein
SMU.644	1.3	39.7	1.0	Putative competence protein, CoiA
SMU.645	1.1	9.8	−1.2	Putative oligopeptidase
SMU.646	1.2	10.3	−1.2	Putative phosphatase
SMU.769	1.2	18.2	−1.3	Hypothetical protein
SMU.772	1.1	4.9	−1.2	Putative glucan-binding protein D, BglB-like protein
SMU.836	1.5	74.8	−1.2	Hypothetical protein, CHAP domain
SMU.837	1.3	32	−1.2	Putative reductase
SMU.838	1.3	2.4	−1.1	Glutathione reductase
SMU.926^e^	1.9	3.7	2.2	GTP pyrophosphokinase, PsrR, RelP
SMU.927^e^	1.7	3.4	1.6	Putative response regulator
SMU.928^e^	1.5	2.7	1.7	Putative histidine kinase
SMU.1001	1.6	45	−1.1	Putative DNA-processing Smf protein, DprA
SMU.1002	1.2	6.5	1.2	DNA topoisomerase I
SMU.1003	1.2	5.8	1.1	tRNA (uracil-5-)-methyltransferase, Gid
SMU.1055	1.0	23	−1.0	DNA repair protein, RadC
SMU.1400c	−1.0	2.7	−1.1	Hypothetical protein
SMU.1916	1.4	6.3	1.7	Histidine kinase of competence regulon, ComD
SMU.1917	1.5	10	1.9	ComE, response regulator of sakacin A production
SMU.1965c	1.1	2.4	1.0	Putative histidine kinase
SMU.1966c	−1.1	3.7	−1.2	Putative periplasmic sugar-binding protein
SMU.1967	1.4	52.8	−1.1	Single-stranded-DNA-binding protein, SsbB
SMU.1978	1.3	7.5	1.1	Putative acetate kinase
SMU.1979c	1.2	29.8	−1.3	Hypothetical protein
SMU.1980c	1.4	102.3	−1.2	Hypothetical protein, ComGG, CglG
SMU.1981c	1.2	68.6	1.0	Hypothetical protein, ComGE, CglE
SMU.1982c	1.1	111.6	−1.1	Hypothetical protein
SMU.1983	1.0	68.8	−1.0	Putative competence protein ComGD, CglD
SMU.1984	1.5	70	−1.3	Putative competence protein ComGC
SMU.1985	1.4	69.8	1.1	ABC transporter ComGB
SMU.1987	1.6	106	−1.1	Putative ABC transporter, ATP-binding protein ComGA
SMU.1997	1.0	25.7	−1.3	ComX, SigX
SMU.2076c	1.0	4.2	−1.3	Hypothetical protein
SMU.2085	1.5	4.6	1.3	Recombinase A, RecA
SMU.2086	1.2	8	1.0	Competence damage-inducible protein A, CinA

a Genes upregulated in response to CSP under at least one of the conditions tested (mean change, >2-fold).

b *S. mutans* gene locus tag as in GenBank (*S. mutans* UA159, accession no. AE014133), except for *comS*, which is not annotated in the *S. mutans* genome.

c Mean fold change in gene expression in two independent biological experiments comparing CSP-treated and untreated parallel cultures.

d Annotation as in GenBank (accession no. AE014133) plus common gene names used in the literature.

e TAR initiated by early gene SMU.925.

A comparison of the expression levels of individual upregulated genes in samples treated with CSP for 10 and 100 min is shown in Table 1. The induced genes form two classes with different temporal patterns of expression; one class was induced at both times, and a second, larger, class was upregulated at 100 min but was not perceptibly affected at 10 min. For convenience in discussion, we designate the former early genes and the latter late genes. As they are likely to have different modes of regulation, transcriptionally active regions (TARs) in the two classes are described separately below.

### In the early phase of the CSP response, increases in expression were restricted to 20 genes in four distinct TARs.

**FIG 3:**
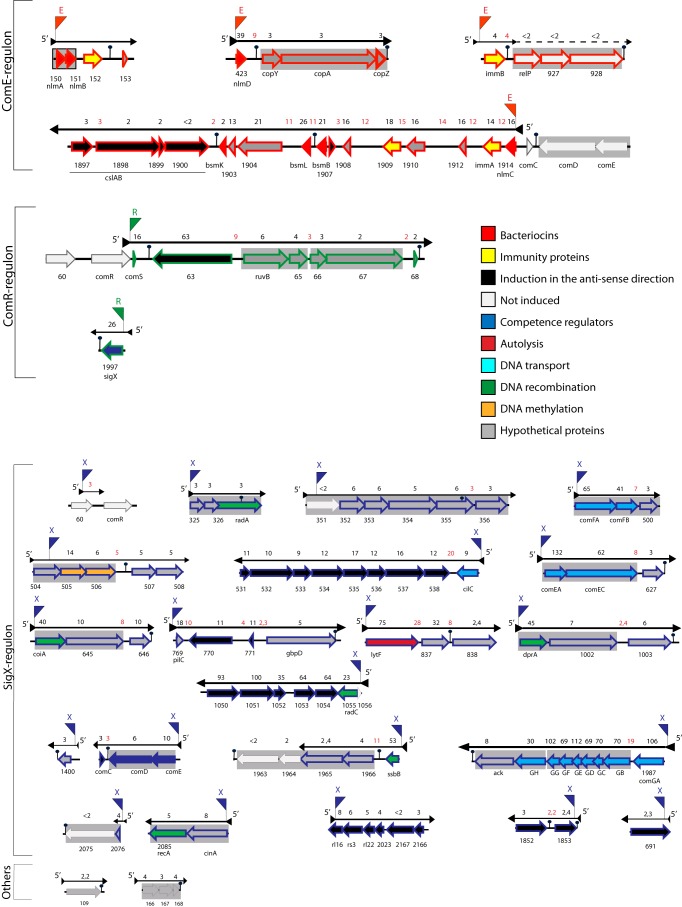
Genetic organization of TARs assigned to ComE, ComR, and SigX regulons. Operons (gray boxes) and terminators (black pins) are indicated as predicted by DOOR (38). The arrow borders of the genes in the ComE, ComR, and SigX regulons are red, green, and blue, respectively. The four TARs induced at 10 min in the WT and at 100 min in the *comS* mutant belong to the ComE regulon and comprise genes encoding bacteriocins (red), bacteriocin immunity proteins (yellow), and hypothetical proteins (gray). The 25 TARs induced only in the WT at 100 min (late response) belong to the ComR regulon (2 TARs), the SigX regulon (21 TARs), or unassigned regulons (others; 2 TARs). These include genes with products acting in autolysis (red), DNA transport (sky blue), recombination (green), and DNA methylation (orange) and genes that encode hypothetical proteins (gray). Three late genes encode key regulatory elements in the CSP response pathway (dark blue), SigX, ComS (ComR activator), and ComED. Binding site consensus elements are indicated by pennants for ComE (E), ComR (R), and SigX (X). Genes upregulated in the antisense direction are black. The mean fold changes in the expression of ORFs (black numbers) and intergenic regions (red umbers) for the WT at 10 min (ComE regulon) and 100 min (ComR, SigX, and other regulons) are shown above the black arrows indicating the directions of the transcripts. The images shown were drawn with Easyfig (70).

All five genes previously reported individually to be upregulated in the early phase of the CSP response (*nlmAB*, *nlmD*, *immB*, and *cipB*), either by means of reporter constructions or by RT-PCR (18, 28, 36), were among the early upregulated genes (Fig. 3). The genes in the early induction class clustered in four chromosomal regions. One locus contains the genes for bacteriocin NlmAB and the cognate bacteriocin immunity protein SMU.152 (37), with transcription continuing past a predicted terminator and through SMU_153, encoding a hypothetical protein. The three intergenic sequences among these genes were also upregulated, suggesting that the four genes are part of a single transcript. Two TARs, encoding the bacteriocin NlmD (BsmC, SMU.423) and the ImmB immunity protein (SMU.925), appear to be essentially monocistronic, with readthrough past terminator elements into a total of six downstream neighboring genes (Fig. 3). For *nlmD*, the induction of the downstream genes was already evident at 10 min, whereas for *immB*, the induction of downstream ORFs SMU.926 to SMU.928 was seen only at 100 min. Although they were late genes, they form a single TAR initiated at the early gene *immB*, thus appearing to be part of the same regulon as *immB* and other early genes, as discussed below. The fourth early upregulated region comprises a 13-kb island, including genes for four bacteriocins (BsmB, -L, and -K and CipB), two immunity proteins (ImmA and SMU.1909), five proteins of unknown function, and an unusually large proportion of apparent intergenic regions. Analysis of expression changes in the antisense direction reveals that a marginally upregulated region extends through SMU.1902 to SMU.1897, apparently as a result of transcriptional readthrough.

### In the late phase of the response to CSP, a total of 83 genes organized into 29 TARs were upregulated.

Comparative analysis of the RNA harvested from CSP-treated cells at 100 min to that isolated from parallel untreated controls reveals that the four TARs of the early CSP response comprising 20 genes continued to be upregulated at this time. The TAR initiated at *immB* was extended to include three other genes (SMU.926 to SMU.928) at this time. In addition, 60 other genes organized in 22 TARs were induced in the sense direction and another three TARs were induced exclusively in the antisense direction by at least 2-fold (Table 1; Fig. 3). Among these, one TAR was initiated at the 3′ end of the SMU.60 gene, upstream of *comR*, and extended to the start of *comR.* Five others included regions of antisense transcription (initiated at *comS*, *cilC*, *pilC*, *radC*, and *comE*), and at six loci, TARs (initiated at SMU.325, SMU.351, SMU.504, *lytF*, *comE*, and *ssbB*) extended past terminators in the sense direction, as predicted by DOOR (38), to include downstream ORFs. The three TARs induced only in the antisense direction of the ORFs start at SMU.691, SMU.1853, and rl16. Over half of the TARs comprise genes upregulated by 10- to 132-fold.

Three of the late upregulated genes encode known regulators of competence-specific transcription in *S. mutans* (ComS, SigX, and ComED). Consistent with a positive feedback loop mediated by XIP (Fig. 1), there was strong induction of the XIP-encoding gene *comS* at 100 min. The TAR initiated at *comS* is 8 kb long, comprising six other ORFs, one of them transcribed in the antisense direction (SMU.63). SigX, long known as part of the response to CSP in streptococci (11), is represented by a one-gene TAR upregulated by 16-fold. Finally, the *comED* genes are among the late genes and are part of a TAR that extends in the 3′ direction to include the transcription of *comC* in the antisense direction. No induction of *comC* in the sense direction was observed. Of the remaining 19 late-induced TARs initiated by genes transcribed in the sense direction, 12 include at least one gene known to be involved in competence; 4 encode proteins for DNA transport, 6 encode proteins for DNA recombination, 1 encodes a protein for DNA methylation, and 1 encodes a protein for autolysis (Fig. 3).

### Deletion of *comS* abrogates the entire late response to CSP.

ComR and ComS form a positive feedback circuit that enhances the synthesis of ComS and concomitantly upregulates *sigX* (6). Although only two copies of the complex ComR box recognition motif have been detected in the *S. mutans* genome, the inference that ComR acts only at those two sites has not been experimentally tested in *S. mutans*. To test this inference and investigate the place of ComS in the CSP response more thoroughly, transcriptome analysis of a *comS* deletion mutant was performed in parallel with the analysis of WT UA159 described above (Table 1; see Tables S4 and S5 in the supplemental material). During CSP treatment of the *comS* mutant for 100 min, none of the late-phase genes identified in the WT were upregulated, but the profile of expression seen at 10 min in the WT was closely recapitulated (Table 1). This pattern supports the inference made from previous studies of several late genes that *comS* is an integral link between the early response and all elements of the late response.

The single gene that was upregulated in the *comS* culture but not in the 10-min WT sample was SMU.926. As shown in Fig. 3, SMU.926 most probably represents a readthrough from the early gene SMU.925 (*immB*) rather than a distinct late-activated promoter. Similarly, the single gene upregulated in the 10-min sample but not upregulated in the Δ*comS* mutant culture is SMU.1902c (*bsmK*). This had the lowest degree of upregulation (2.1-fold) among the early genes and is located at the 3′ end of a long TAR extending from the early gene *cipB*, which was induced by 16-fold, again indicating a possible readthrough past a cryptic terminator site. In fact, the transcripts at all four early loci extended across predicted terminators and operon borders, indicating readthrough as a result of strong activation or incorrect terminator predictions.

### Assignment of regulon members.

**FIG 4:**
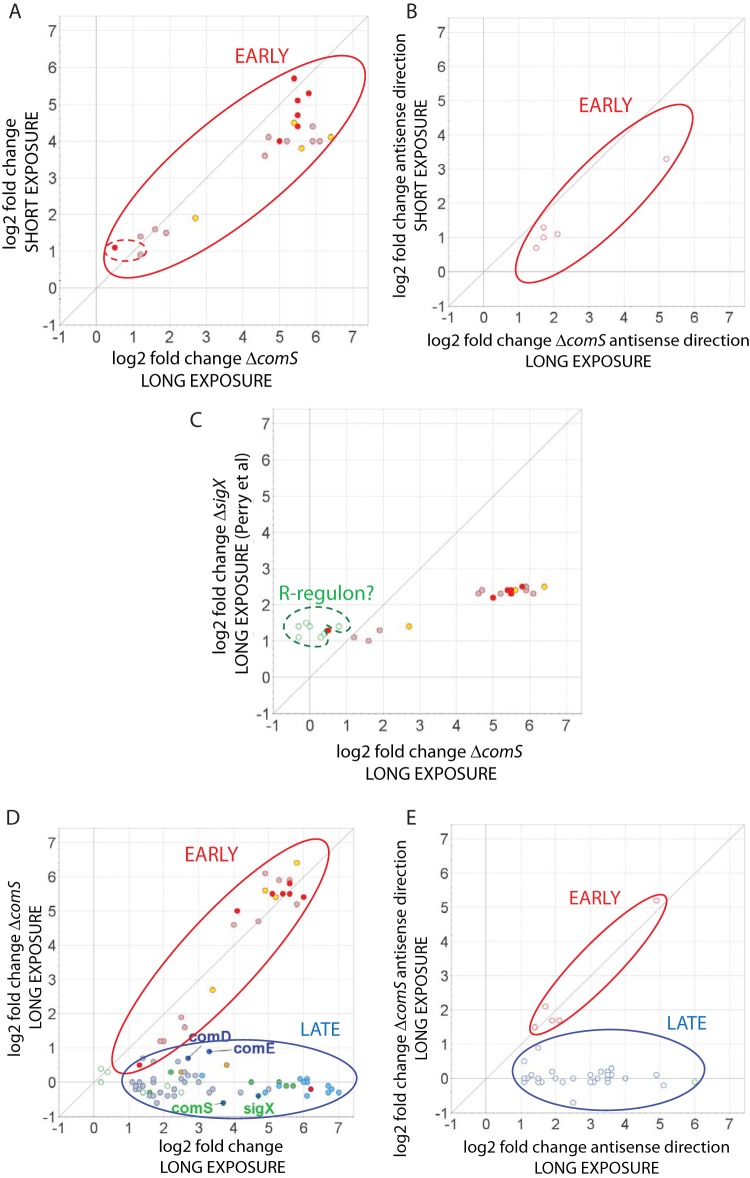
Correlations between gene expression changes induced by short (10 min) or long (100 min) exposure to CSP in *S. mutans* UA159 or by long exposure to CSP in the *comS* mutant. Fold changes are shown as log_2_ values for all induced ORF sequences in the *S. mutans* UA159 genome (accession no. AE014133) and represent mean values for comparisons of CSP-treated and untreated samples from two independent biological experiments. (A) Correlation of ratios of short CSP exposure of *S. mutans* UA159 to long CSP exposure of the *comS* mutant. The same genes were upregulated under the two conditions, with two exceptions (inner dashed circle). These are grouped as early genes. (B) Same correlation as for panel A but for changes in antisense transcripts. (C) Correlation of ratios of long CSP exposure of the *comS* mutant to long CSP exposure of the *sigX* mutant in reference 15 reveals candidate genes for the ComR regulon (dashed circle). (D) Correlation of induction ratios of long CSP exposure of *S. mutans* UA159 to the *comS* mutant formed two groups. Early genes, as in panel A, were induced in both transcriptomes, and late genes were those induced only in UA159 upon long CSP exposure. (E) Correlations as in panel D but for antisense transcripts. Early genes are defined as those induced by short CSP exposure in WT UA159 and by long CSP exposure in the *comS* mutant (red borders), and late genes are defined as those that showed induction in the WT only upon long CSP exposure (green borders for putative genes of the ComR regulon and blue borders for the remaining genes). Circles corresponding to genes encoding upregulated bacteriocins are filled in red, circles corresponding to bacteriocin immunity proteins are filled in yellow, circles corresponding to proteins involved in DNA uptake are filled in light blue, and circles corresponding to proteins involved in DNA recombination are filled in green. The points corresponding to *comE*, *comD*, *comS*, and *sigX* are indicated by name (D; dark blue). Light-gray-filled circles correspond to other genes. Multiplot v.2 was used to draw the scatterplots (http://www.broadinstitute.org/cancer/software/genepattern/modules/docs/Multiplot/2).

**FIG 5:**
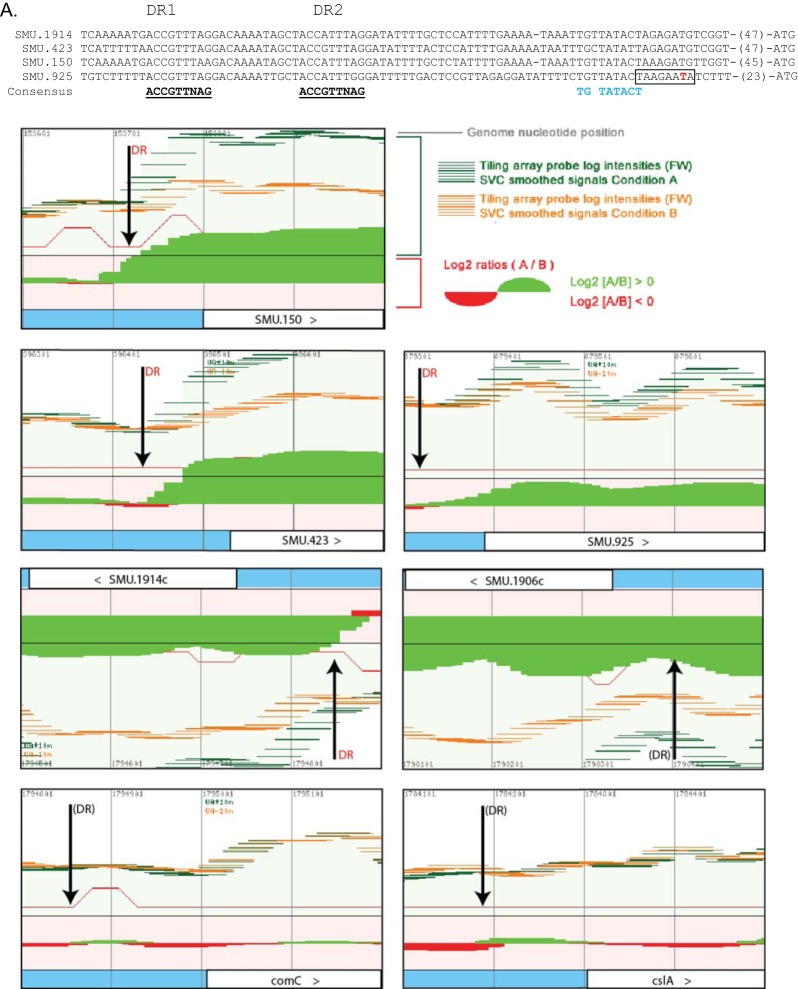

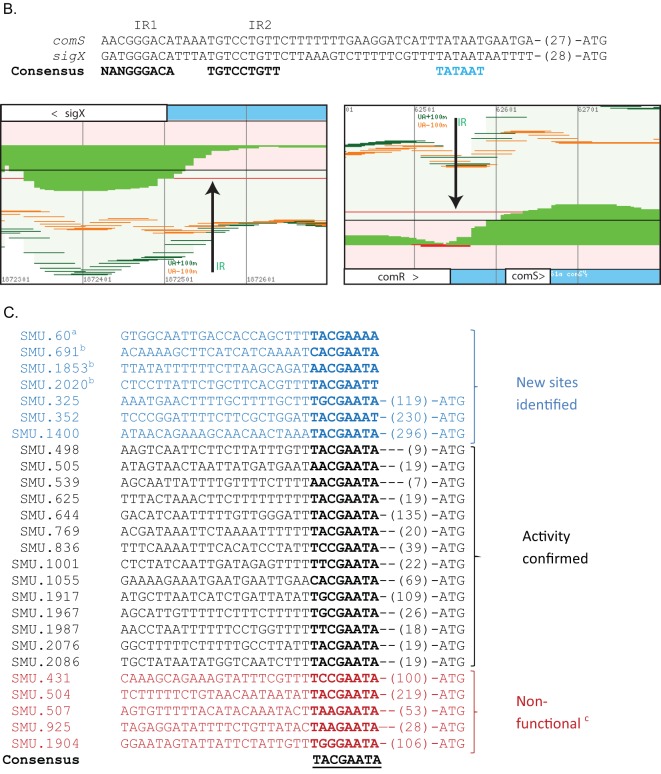

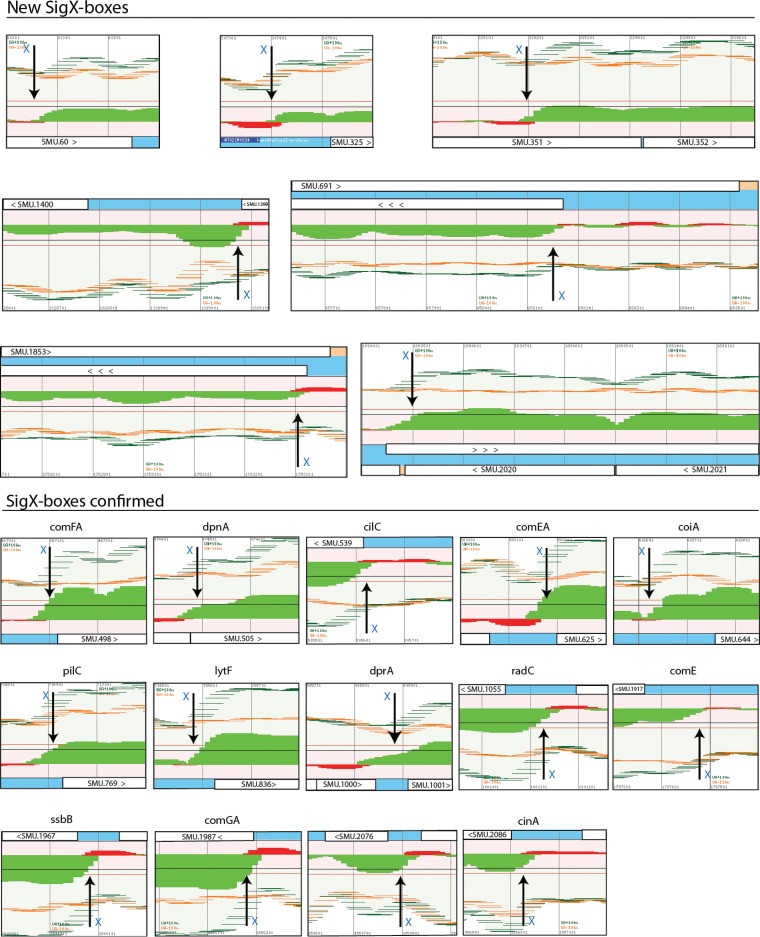
Putative regulatory sites at early and late CSP-induced loci. (A, top) Alignment of DNA sequences upstream of clusters of early genes. The presumptive canonical promoter −10 site is blue, bases corresponding to the DR consensus sequences are underlined, and the putative transcription start site is red (18). The previously suggested SigX box of SMU.925 is boxed (39). (A, bottom) Transcriptome map showing predicted ComE binding sites (15, 40). Vertical arrows show the ComE DR sites that appeared to be active (orange DR) and sites that did not appear as active (black DR). Conditions A and B are 10 min of CSP and 10 min of no CSP, respectively. (B, top) Alignment of DNA sequences upstream of *sigX* and *comS*. These are late CSP-induced loci upregulated by ComRS. The presumptive canonical promoter −10 site is blue, and conserved IRs known as the ComR box are bold (6). (B, bottom) Transcriptome maps at *comS* and *sigX*. Vertical arrows indicate the ComR IR sites (6). Conditions A and B are 100 min of CSP and 100 min of no CSP, respectively. Probe intensities for the *comS* region were above the threshold used in the visualizer program. (C, top) Alignment of DNA sequences with putative SigX boxes. The SigX boxes are in bold. Superscript letters: a, SigX box within SMU.60; b, induction in the antisense direction; c, putative SigX boxes (39) not confirmed in our transcriptome. (C, bottom) Transcriptome map showing locations of predicted SigX boxes. Vertical arrows show the sites of SigX boxes that appeared to be active. Conditions A and B are 100 min of CSP and 100 min of no CSP, respectively. Changes in gene expression are presented as log_2_ ratios (condition A/condition B), with ratios of >0 in green and ratios of <0 in red. The signal intensity of each probe (50-mer) is presented as a horizontal line in green for condition A and in orange for condition B. *S. mutans* locus tags are as in the sequence with GenBank accession no. AE014133 (>, transcript in the forward strand showing probe intensities above the locus tag boxes; <, transcript in the reverse strand showing probe intensities below the locus tag boxes). The vertical lines are separated by a distance of 100 bp. The complete maps are available at http://bioinformatics.forsyth.org/mtd/dataset=RNAseq_smut_comS.

**FIG 6:**
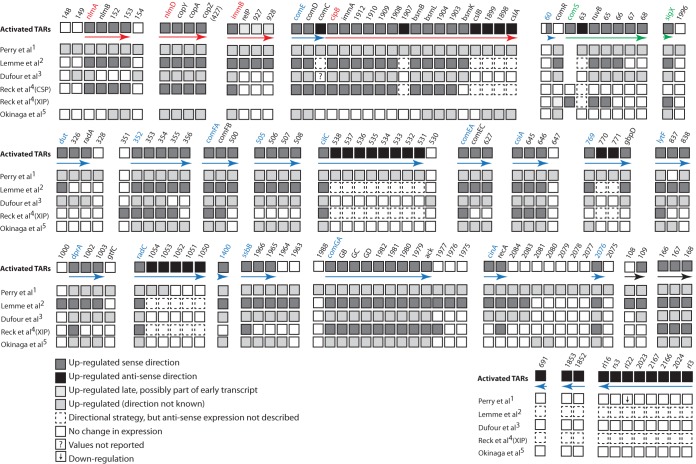
Comparison of gene expression maps from available *S. mutans* competence transcriptome studies. The directions of the activated TARs (arrows) from *S. mutans* exposed for 100 min to CSP are indicated by arrows as follows: red, TARs of the ComE regulon; green, the ComR regulon; blue and black, TARs of the SigX regulon with 5′ ends starting in the sense and antisense directions, respectively. The genes proximal to the ComE DR are red, those proximal to the ComR indirect repeat are green, and those proximal to the SigX box are blue. Gene numbers are as in the sequence with GenBank accession no. AE014133. Gene expression data for corresponding regions are indicated for five transcriptome studies, as indicated at the left. The top row is UA159 (100 min). The transcriptomes in studies 1, 3, and 5 used traditional microarrays with probes limited to annotated ORFs (15, 32, 39). The transcriptome in study 2 used a tiling microarray covering most of the *S. mutans* genome (28). Study 4 used RNA sequencing in the analysis of the response to CSP and XIP in CDM (27). The CSP response in this medium does not support activation of competence but is included for comparative analysis of early responses. The transcriptome for XIP in CDM used a 30-min exposure to XIP, which was probably too short to detect the full range of core genes regulated by SigX. The *comS* gene was not represented in the arrays for the transcriptomes in studies 1, 2, 3, and 5 (empty space).

For a comprehensive identification of the members of each of the three principal competence regulons, we combined information on the temporal responses to CSP and on the effect of *comS* deletion described above with a thorough analysis of the transcriptome map in the vicinity of conserved sequences recognized by ComE, ComR, and SigX (Table 1; Fig. 3 to 5). We then compared this information with the transcriptome data from five previous surveys investigating competence in *S. mutans* to search for conserved responses (Fig. 6). Two of the transcriptomes compared long exposure times (120 min) of strain UA159 to CSP with nontreated samples (15, 28); the third transcriptome was also for strain UA159, but the comparison was between the WT and a CSP response-defective *cipB* mutant also exposed to CSP for 120 min (32); the fourth one was for strain UA140 comparing the WT to an *hdrR* overexpression strain that shows induction of late competence genes in the absence of CSP supplementation (39); and the fifth one used short (up to 30 min) exposure of UA159 to CSP or XIP in a defined medium in which CSP responses are not linked to competence (27).

### The ComE regulon.

To delimit the ComE regulon, we first compared the transcriptome profiles of the early response of the WT to CSP and of the *comS* mutant exposed for 100 min to CSP, as illustrated in Fig. 4A and B. The expression pattern at 10 min is expected to reveal the immediate response of genes transcribed via ComE activation, before regulons dependent on ComR are upregulated, whereas data from the late response of a *comS* mutant, in which downstream regulons remain silent because of a lack of the linking XIP peptide, would potentially reveal direct targets of ComE with a slower response, as well as other downstream regulons independent of the ComR-ComS link. We found near identity between the early response in the WT and the late response in the *comS* mutant. All four early-induced TARs initiated at *nlmA*, *nlmD*, *immB*, and *cipB* are preceded by the direct repeat (DR) identified previously as a putative ComE binding site required for CSP-dependent bacteriocin expression (18) and subsequently identified (40) as a tight binding site for purified ComE protein (Fig. 4 and 5). Other DR sites that bind purified ComE have been suggested to be functional, but they appeared to be nonfunctional under the conditions examined here, including the sites distal to *bsmB* (SMU.1906), *comC* (sense direction), and *cslAB* (Fig. 5A). The results indicate that all of the targets of ComE respond immediately to CSP and establish for the first time that ComE has no additional targets activated at late times independent of the ComR-ComS link.

We further examined the conservation of the CSP response by comparing the set of genes within the induced TARs with genes within or in the vicinity of these regions showing changed expression in previous studies (Fig. 6). Although the one published transcriptome study evaluating the early CSP response (27) was conducted under conditions of culture in CDM that do not support induction of competence by CSP, it did identify 19 of the 23 genes within the ComE regulon, as determined here (Fig. 5). Notably, similar to our results, in that study, *comED* and *comC* expression was only slightly or not induced by CSP, a finding also corroborated by a previous transcriptome study using a *sigX* mutant (15). Despite the methodological limitations of the latter study, including lack of information on the direction of the transcripts and low levels of induction by CSP, all of the 23 early genes induced in the sense direction in this study were identified among the induced genes reported there (Fig. 4C). We conclude that the UA159 genome contains only four TARs that are directly regulated by ComE, defining the ComE regulon. A total of 23 genes are transcribed in the sense direction, but direct experimentation is needed to assess the biological significance of distal genes or of the antisense transcripts in all four TARs, which are the source of the greatest variation among the different transcriptome studies.

### The ComR regulon.

Genes transcribed directly via activation of ComR in the CSP response can be identified from transcription differentials between a *sigX* mutant and a *comS* mutant in the late phase of the CSP response. Both data sets would reflect upregulation of genes of the ComE regulon, which is an early response, but only the *sigX* mutant would allow increased late expression of the transcripts of the ComR regulon. Although the information derived from a previous survey of gene expression in a *sigX* mutant (15) did not address transcript polarity or intergenic sequences, we can nonetheless use the ORF expression information available there to uncover candidate members of the ComR regulon. The scatterplot in Fig. 4C compares the genes that were upregulated in the 100-min *comS* mutant culture in this study to those previously reported as upregulated in a *sigX* mutant after a similar period of CSP exposure. The genes listed as upregulated in both transcriptomes represent the ComE regulon, as described above. The remaining seven genes, which were upregulated in the *sigX* mutant but not in the *comS* mutant or within a 10-min CSP exposure, are thus candidates for the ComR regulon. Of these, we exclude three (SMU.2037, SMU.2038, and SMU.799c) that, although induced to low levels in the *sigX* mutant, were not upregulated in the late response of the WT in either of the transcriptomes here (see Table S6 in the supplemental material) or in two other previous transcriptomes (27, 28) and are not preceded by the ComR box inverted repeat (IR). The remaining candidate genes, SMU.63, SMU.64, SMU.65, and SMU.66, are contiguous with *comS*, which was not itself represented in the microarray used for analysis of the *sigX* mutant. These four genes were also upregulated in the 100-min WT transcriptome, along with the downstream genes SMU.67 and SMU.68, forming a single TAR with *comS*. The present data reveal that SMU.63, immediately downstream of *comS* but in the inverse orientation, was transcribed only from the noncoding strand, indicating strong readthrough past *comS* and suggesting that the entire upregulated region downstream of *comS* represents a single readthrough mRNA (Fig. 3). Examination of the transcription map for the WT at 100 min reveals that this TAR starts close to the ComR box IR that has been described as the binding site for ComR (Fig. 5B) (41), further supporting a role for ComR in driving the transcription of this region.

10.1128/mSystems.00038-15.5 Table S5  Effect of CSP on the expression of intergenic sequences. Mean fold changes and *P* values for all intergenic regions are shown. Download Table S5, PDF file, 2.7 MB. Copyright © 2016 Khan et al. 2016 Khan et al. This content is distributed under the terms of the Creative Commons Attribution 4.0 International license.

10.1128/mSystems.00038-15.6 Table S6  Initial selection of candidate genes of the ComE and ComR regulons. Download Table S6, PDF file, 0.4 MB. Copyright © 2016 Khan et al. 2016 Khan et al. This content is distributed under the terms of the Creative Commons Attribution 4.0 International license.

The final member of the ComR regulon is *sigX*, which was expressed as a late gene, but not in the *comS* mutant (and was not probed in the *sigX* mutant). As for *comS*, the transcription start site at *sigX* mapped to a ComR box IR motif (Fig. 5B), providing support for a direct regulatory role for ComR in the expression of both *comS* and *sigX*. A previous transcriptome study of CSP-induced competence in *S. mutans* reported a TAR extending downstream of *sigX* that, given its position and the general variation observed in the expression of the 3′-terminal genes in several of the induced TARs in different transcriptomes, may represent a readthrough (Fig. 5). We conclude that just two TARs make up the ComR regulon, one initiated at *comS* but often extending downstream to encompass two to five additional genes and the second encompassing *sigX*, with an occasional downstream readthrough.

### The *sigX* regulon.

Because *sigX* is expressed late in the CSP response, genes restricted to late expression and not expressed in a *sigX* or *comS* mutant are candidates for the SigX regulon. The late class of genes is easily distinguished from the early class, as illustrated in the scatter plots in Fig. 4D and E. Discounting the genes belonging to the ComE and ComR regulons identified above (Fig. 4A to C), 23 TARs comprising a total of 78 ORFs transcribed in either the sense or the antisense direction are candidate members of the SigX regulon (Fig. 3, bottom). To distinguish direct from indirect regulation by SigX, we focused on the apparent presence of the highly conserved noncanonical −10 promoter element recognized by SigX polymerase, the SigX box, near the start of these TARs. Twenty-one of the 23 candidate TARs are preceded by a SigX box, as illustrated in the transcriptome maps in Fig. 5C. Of these, 7 represent SigX boxes not previously described and the remaining 16 include mostly SigX boxes that have been predicted in previous *S. mutans* studies and that were confirmed here by transcriptome mapping. Five other SigX boxes have been suggested in regions preceding competence-induced genes (SMU.431, SMU.504, SMU.507, SMU.925, and SMU.1904) (39), but these boxes were not confirmed in our study. At least one gene in each of these TARs (those in the sense direction) has been previously reported as upregulated in transcriptome surveys of the *S. mutans* response to CSP (Fig. 6). In the cases where there was some variation among the different transcriptomes regarding the set of genes that were induced within the SigX-controlled TAR regions, these usually involved genes in the 3′ termini of the TARs. Only two TARs, one for SMU.109 (possibly initiated at SMU.108) and another extending from SMU.166 to SMU.168, were upregulated in multiple transcriptomes but lack an apparent upstream SigX box (Fig. 6). These two are thus the only candidates for indirect regulation by SigX. We conclude that the SigX regulon comprises 23 TARs, with conserved upregulation of genes toward the 5′ ends of the TARs and a certain level of variation among different transcriptome surveys toward the set of genes activated in the 3′-terminal regions of the TARs.

### A core *sigX* regulon.

**FIG 7:**
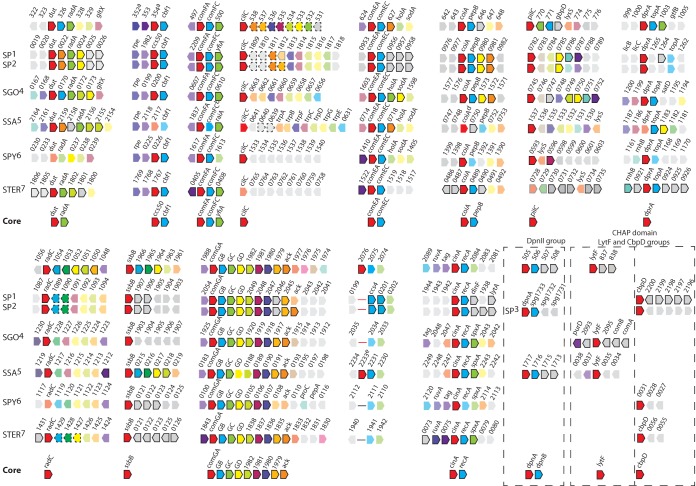
Core genes of the SigX regulon. Conservation of synteny to *S. mutans* SigX-induced TARs (top row) in *S. pneumoniae*, *S. gordonii*, *S. sanguinis*, *S. pyogenes*, and *S. thermophilus*. Red pentagons correspond to genes immediately downstream of a SigX box. Competence-induced genes, black border; upregulated genes oriented antisense to the gene proximal to the SigX box, dashed black border; no change in gene expression, borderless faded colors; orthologs in each of the 15 groups, similar colors; no orthologs in the regions analyzed, gray. Core genes in the DpnII group are shown within dashed squares. The induced *lytF* gene in *S. mutans* is also induced in *S. gordonii* and *S. sanguinis* and encodes a CHAP domain found in CbpD, an induced nonortholog in *S. pneumoniae*, *S. thermophilus*, and *S. pyogenes*. LytF and CbpD have conserved lytic functions in the different species. Three other transcripts induced in *S. mutans* are not shown, as they have no matches in the other species (SMU.1400, *comE*) or are internal to an ORF (3′ end of SMU.60). Orthology is as annotated at KEGG (http://www.genome.jp/kegg/). Note that for all of the transcriptomes compared with *S. mutans*, there is no information on TAR polarity or length. SP^1^, *S. pneumoniae* Rx (13); gene locus tag as in GenBank (*S. pneumoniae* TIGR4, accession no. AE005672). SP^2^, *S. pneumoniae* R6 (44); gene locus tag as in GenBank (*S. pneumoniae* TIGR4, accession no. AE005672). SP^3^, *S. pneumoniae* G54 (56) DpnII restriction-methylase group (competence transcriptome not available); gene locus tag as in GenBank (*S. pneumoniae* G54, accession no. CP001015). SGO^4^, *S. gordonii* Challis (14); gene locus tag as in GenBank (accession no. CP000725). SSA^5^, *S. sanguinis* SK36 (45); gene locus tag as in GenBank (accession no. CP000387.1). SPy^6^, *S. pyogenes* MGAS315 (9) and derivative strain D471 (46); gene locus tag as in GenBank (*S. pyogenes* M1 GAS SF370, accession no. AE004092.2). Shown are *S. pyogenes* induced genes in one or both studies. STER^7^, *S. thermophilus* LMD-9 (43); gene locus tag as in GenBank (accession no. GCA_000014485.1). ^a^SMU.354 is homologous to *ccs50*, but the SigX box is distal to SMU.352. ^b,c,d^Upregulation of *comFC*, *cilC*, and *coiA* was not detected in the *S. sanguinis* transcriptome but is essential for competence (45). ^e^SSA.2233 is distal to the SigX box but not homologous to SMU.2076.

**FIG 8:**
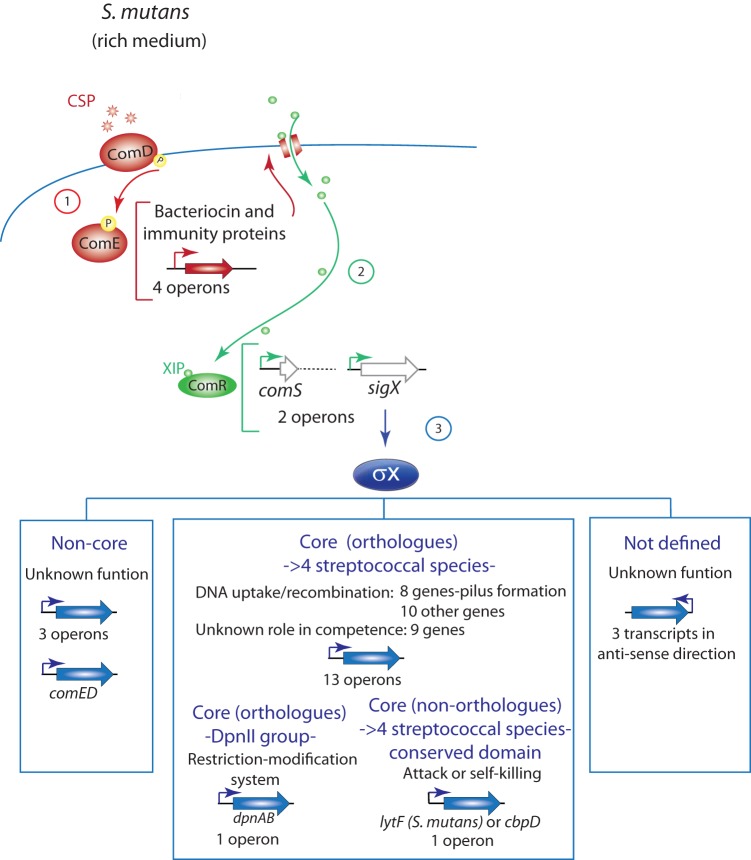
Model of the transcriptional organization of the *S. mutans* response to CSP in peptide-rich medium. In step 1, extracellular CSP activates the two-component system ComED, which then induces the expression of four operons comprising several bacteriocin and bacteriocin immunity genes. The bacteriocins are suggested to create pores in the membrane that allow the entrance of the XIP pheromone into the cells. In step 2, XIP binds to ComR, which then assumes a conformation that activates the expression of two operons, one initiated at *comS*, creating a positive feedback loop, and the other initiated at *sigX*, activating the competence response. In step 3, SigX activates the expression of 22 operons, including 4 sense transcripts that are not seen in other streptococcal transcriptomes (noncore), 3 transcripts in the antisense direction (not yet investigated in other streptococci), and 15 operons encoding genes that are in the core of the streptococcal SigX response. Three groups of core genes were identified: (i) core orthologs found in at least four of the available transcriptomes, (ii) core orthologs found only in strains that belong to the DpnII group of restriction-modification systems, and (iii) core nonorthologs that have in common a conserved domain associated with similar functions (in this class are CbpD of *S. pneumoniae* and *S. thermophilus* and LytF of *S. mutans*, *S. gordonii*, and *S. sanguinis*, both of which have the lytic CHAP domain in common).

During their evolution from a common ancestor, the streptococci became specialized for survival in diverse hosts and diverse sugar-rich niches, diverging not only by accumulation of mutations but also by gains and losses of many genes and by extensive shuffling within the genome (42). Throughout this evolution, competence for genetic transformation has been a conserved trait dependent on the alternative sigma factor SigX and the SigX regulon (12). The maintenance of the SigX regulon amid pervasive genetic change provides a natural genus-wide survey that can be used to distinguish conserved from variable components of the regulon. To mine the data provided by this natural experiment, we compared the competence-specific transcriptome data sets that are available for six species, representing five of the six major groups of species in this genus. In Fig. 7, we display alignments of competence-specific TARs in *S. mutans* with homologous regions that are upregulated in response to CSP in *S. pneumoniae*, *S. gordonii*, and *S. sanguinis* or by XIP in *S. pyogenes* and *S. thermophilus* (9, 13, 14, 43–46). Since induction levels are apparently low in *S. pyogenes* (9, 46) and transformation is observed only under particular biofilm conditions (47), the absence of core induced genes in their transcriptomes was not used as evidence for the lack of a core response. However, induction of *S. pyogenes* genes within core regions strengthened the classification of genes into the SigX core response. Inspection of the alignments reveals a broad pattern of synteny adjacent to the SigX box motifs but also variation in both gene presence and the observed length of associated TARs. Overall, the streptococcal SigX regulons contain a minority of genes invariably subject to SigX regulation, organized into three groups, (i) core genes induced in more than four streptococcal species (*dut*, *radA*, *ccs50*, *cbf1*, *comFA*, *comFC*, *yfiA*, *cilC*, *comEA*, *comEC*, *coiA*, *pepB*, *pilC*, *dprA*, *radC*, *ssbB*, *comGA* to *comGH*, *ack*, *cinA*, and *recA*), (ii) core genes in the DpnII group of strains (*dpnA* and *dpnB*), and (iii) core genes that have a domain in common but are not necessarily orthologs (*lytF* in *S. mutans*, *S. gordonii*, and *S. sanguinis* and *cbpD* in *S. pneumoniae*, *S. thermophilus*, and *S. pyogenes*), which we define as the core SigX regulon. This information was used to delineate a model of the transcriptional organization of the *S. mutans* response to CSP in peptide-rich medium (Fig. 8). A larger number of accessory genes found in SigX-dependent transcripts in some species but not in others are exemplified in Table S7 in the supplemental material. A notable feature of the core regulon is that core genes are usually at the 5′ extremity of a TAR, which immediately suggests that transcription of the accessory genes may reflect variation in terminator presence and efficacy. Indeed, in no case that we are aware of has an accessory gene downstream of a core gene been shown to provide a function in transformation. Additional accessory SigX-dependent genes are found in TARs that lack any core gene, but these are few in *S. mutans*, as only 4 of 22 TARs initiated by genes transcribed in the sense direction are not induced in the other species (3′ end of SMU.60, *comED*, SMU.1400, and possibly SMU.2076).

10.1128/mSystems.00038-15.7 Table S7  Comparison of transcriptome results for late genes in *S. mutans*, *S. pneumoniae*, and *S. thermophilus*. Download Table S7, PDF file, 0.7 MB. Copyright © 2016 Khan et al. 2016 Khan et al. This content is distributed under the terms of the Creative Commons Attribution 4.0 International license.

## DISCUSSION

A common theme among the pathways controlling competence for genetic transformation in the scores of species within the diverse genus streptococcus (12) is the use of the labile and dispensable alternative sigma factor SigX to drive the transcription of genes for DNA-processing functions. Our identification of a core of just 27 to 30 genes that are consistently placed under the control of SigX in representative streptococcal species draws attention to three classes of genes, (i) upstream regulators of *sigX* and genes they regulate in parallel with *sigX*, (ii) the core SigX regulon genes themselves, and (ii) coregulated genes beyond the core that depend on SigX for expression in species-specific patterns.

The links of SigX to upstream regulators and pheromone communication systems are found in species-specific arrangements, but in all of the cases characterized so far, *sigX* expression is coordinated by the activity of a pheromone peptide-dependent quorum-sensing system. In *S. mutans*, competence development can be provoked through two alternative but convergent regulatory pathways initiated by alternative intercellular peptide signals (Fig. 1) and regulators of both classes. Although these quorum-sensing systems vary, they both coordinate bacteriocin production with upregulation of *sigX*. Bacteriocin production accompanying competence development has been proposed as a mechanism to scavenge DNA from target cells that can then be used for genetic exchange (36, 48). Indeed, in mixed cultures, *S. mutans* UA140 can use bacteriocin induction to facilitate an attack on *S. gordonii* and increase gene transfer between the species (36). Other regulators also feed into one or the other of these pathways to modulate their activities at unknown points upstream of SigX, including, for instance, ScnC/R/K, HdrRM, and BsrRM in *S. mutans* (39, 49–52) and CiaRH and StkP in *S. pneumoniae* (53–55).

The LytR family response regulator that mediates the response to CSP in *S. mutans*, ComE, is among the most-studied regulators in this species, yet its regulatory targets have not been fully defined (18, 39, 56). The present results support the resolution of these uncertainties in favor of only four significant sites of ComE action. Their location uniquely at mutacin loci is consistent with the phylogeny placing “ComED” of *S. mutans* in the BlpRH family of bacteriocin regulators, distinct from the ComED competence regulators that are shared by the *S. mitis* and *S. anginosus* groups (10, 12, 57).

The core SigX regulon genes identified in Fig. 7 are listed in Table 2, grouped according to the known roles of their protein products and information now available about their potential roles. Of the 27 core genes with orthologs in more than 4 of the different species analyzed, 2/3 have been characterized to some extent as important for genetic transformation in *S. pneumoniae* or other species. Twelve are absolutely required for DNA uptake in *S. pneumoniae*, and six are important for subsequent recombination. The remaining nine genes have unknown roles in competence and include four orthologs of well-characterized proteins and five proteins with unknown function, some with domains that fall into known broad functional categories. All nine of these are dispensable for transformation in *S. pneumoniae* but may play a role in competence in other species (39). Two core genes are specific for the DpnII group of streptococci (*dpnA* and *dpnB*). In this group, methylation by DpnA protects incoming heterologous DNA from digestion by DpnII restrictases (56). A third class of core genes is represented by *lytF* and *cbpD*, of which the former is found in *S. mutans*, *S. sanguinis*, and *S. gordonii* and the latter is found in *S. pneumoniae*, *S. thermophilus*, and *S. pyogenes*. Although the two genes are not orthologs, they both have a CHAP domain with conserved lytic activity that is important in promoting DNA release during competence (58–61).

**TABLE 2:** Roles of the genes of the core SigX regulon in transformation

Function and locus tag^a^			Gene	Role or activity (reference[s])	Transformation of mutants (reference[s])^b^		
SMU no.	SP no.	SSA no.			SMU	SP	SSA (45)
Recombination							
327	0023	2157	*radA*	DNA repair protein RadA (71)		↓ (71)	
644	0978	0749	*coiA*	Recombination		↓ (72)	↓
1001	1266	1185	*dprA*	DNA-processing protein, mediates RecA (72, 73) loading onto internalized single-stranded DNA, competence shutoff (74–76)	↓ (77)	↓ (13, 74)	
1967	1908	0214	*ssbB*	Single-stranded DNA binding, DNA protection (78)	↓ (77)	↓ (79–81)	↓
2085	1940	2245	*recA*	Recombinase A, strand exchange (74, 82)	↓ (77, 83)	↓ (84)	↓ (85)
Recombination (core in DpnII group)							
505	**1734**	1717	*dpnA*	Single-strand methylase (56)		↓ (56)^g^	
506	**1733**	1716	*dpnB*	DpnII restrictase targeting unmethylated double-stranded DNA (56)	Yes (77)		
				Uptake			
498	2208	1836	*coMFA*	ATP-dependent DNA/RNA helicase, translocase involved in single-stranded DNA uptake (86)		↓ (87)	↓
499	2207	1835	*comFC*	Late competence protein (59)		↓ (59, 87)	↓
539	1808	0642	*cilC*, *pilD*	Signal peptidase type IV, pilin cleavage (13, 88)	↓ (39)	↓ (13)	↓
625	0954	0715	*comEA*	Membrane protein with DNA-binding motif (double-stranded DNA receptor) (89)		↓ (90)	↓
626	0955	0716	*comEC*	Membrane channel (59, 91)	↓^c^	↓ (90)	↓
1979	2045	0191		Adenine-specific DNA methylase (13)	↓ (50)	Yes^d^ (13)	
1980	2047	0190	*comGG*	Minor pilin (92–94)	↓ (50)	↓ (13, 93)	↓
1981	2048	0189	*comGF*	Minor pilin (92–94)	↓ (50)	↓ (93)	
1982	2049	0188	*comGE*	Minor pilin (92–94)	↓ (50)	↓ (93)	↓
1983	2050	0187	*comGD*	Minor pilin (92–94)	↓ (50)	↓ (93)	↓
1984	2051	0186	*comGC*	Major pilin (92, 94)	↓ (50)	↓ (59, 92, 93)	↓
1985	2052	0185	*comGB*	Competence protein, ABC transporter subunit (94)	↓ (50)	↓ (59, 93)	↓
1987	2053	0184	*comGA*	Pilus assembly ATPase (94)	↓ (50)	↓ (59, 92, 93)	↓
Lysis (CHAP domain conserved)							
836		0036	*lytF*	Murein hydrolase (39, 61, 77, 95)	↓ (39, 77)		
	2201		*cbpD*	Murein hydrolase (96)		Yes (96)	
Unknown functions in competence^*h*^							
325	0021	2160	*dut*	Deoxyuridine 5′-triphosphate nucleotidohydrolase		Yes (13)	
354	1981	2117	*rmuC*, *ccs50*	DNA recombination protein RmuC		Yes (13)	
355	1980	2116	*yhaM*, *cbf1*	CMP-binding factor 1, 23S RNA maturation		Yes (13)	
500	2206	1834	*yfiA*	Ribosome hibernation-promoting factor (Hpf), sigma factor (σ^54^) modulation		Yes (13)	
645	0979	0751	*pepB*	Oligopeptidase		Yes (13)	
769	0782	1537	*pilC*	Membrane protein of pilus assembly	Yes^e^** (32, 39, 77)	Yes (13)	
1055	1088	1218	*radC*	DNA repair protein RadC	↓ (39)	Yes (45)	
1978	2044	0192	*ackA*	Bifunctional acetaldehyde-coenzyme A/alcohol dehydrogenase (13, 97)	Yes (97)	Yes (93)	
2086	1941	2246	*cinA*	Competence damage-inducible protein A (98)	↓^f^ (98)	Yes (85)	

a Gene locus tag as in GenBank (SMU, *S. mutans* UA159, accession no. AE014133; SP, *S. pneumoniae*, TIGR4 accession no. AE005672; in bold, *S. pneumoniae* G54, accession no. CP001015; SSA, *S. sanguinis* SK36, accession no. CP000387.1).

b Yes, deletion mutants not affected in transformation; ↓, >2-fold reduction.

c Unpublished data.

d 70% of WT.

e Results with CSP showed a <2-fold reduction, but in one of the studies without CSP, there were no transformants (32).

f Deletion of *cinA* affected the expression of the downstream gene *recA*, which was not fully restored by *cinA* complementation from a plasmid.

g Transformation with unmethylated DNA was reduced in the deletion mutant.

h Annotation at KEGG (99).

The predominance of transformation functions in the core SigX regulon suggests that this is an ancient regulon maintained because of its value in promoting genetic flexibility and maintaining ready access in each species to a large pangenome. Consistent with this view is its frequent linkage to production of lysins and bacteriocins, which can facilitate access to DNA from living cells. The question that arises concerns the functions of the remaining third of the core regulon. Although competence is suggested to be a stress response and these genes might act to relieve some stresses, it is our working hypothesis that they support horizontal gene transfer and that the absence of a phenotype in a standard transformation assay may reflect some redundancy in their activities, activities important under circumstances not yet tested, or simply functions in some aspect of the natural transformation process not yet appreciated. It is interesting, for example, that two of the nine core genes with unknown function in competence appear to have targets in the ribosome and puzzling that one of these is known in other species to inactivate ribosomes under stress (62).

The noncore genes of the SigX regulons vary among species, but direct evidence connecting any of them to a SigX-controlled phenotype is rare. In a few cases, a species- or group-specific role in transformation is already known. One such case is that of ComE, the *S. mutans* bacteriocin regulator that links XIP-stimulated competence to the expression of bacteriocins by induction of ComED (27, 34). In other species, such as *S. pneumoniae* and *S. gordonii*, SigX establishes a direct link to bacteriocin production by recognizing the SigX box in the promoters of bacteriocin genes (48, 63). However, in the majority of cases, a role related to transformation is simply unknown. The frequent occurrence of apparent readthrough transcripts observed here suggests that pervasive transcription is a general feature of the competence response, which probably contributes to the list of noncore genes. Pervasive transcription represents a widespread phenomenon, as recently reviewed by Wade and Grainger (64) and as exemplified by findings that in *Bacillus subtilis* approximately 13% of the TARs seem to lack efficient termination signals (65). Bacteria have apparently developed mechanisms to minimize pervasive transcription, but these are mostly unknown in streptococci.

The comprehensive identification of *S. mutans* regulons activated in response to peptide pheromones provides an important basis for understanding how *S. mutans* can transition from individual to social behavior. *S. mutans* is an inhabitant of the oral cavity, where its ability to form biofilms is thought to be crucial for colonization. Biofilm formation by *S. mutans* in rich medium is enhanced by CSP (20, 35), whereas XIP in a defined medium has an inhibitory effect (17). Thus, it is clear that both pheromones may influence the *S. mutans* biofilm mode of growth. Once in biofilms, both XIP and CSP pheromones may provide *S. mutans* with a competitive advantage by activating the production of bacteriocin, which is used to attack competitors, and by increasing their ability to take up exogenous DNA and therefore adapt to the environment. It is also known that CSP may increase the ability of *S. mutans* to tolerate acid stress (19) and that competence is repressed under acidic conditions (66, 67). These effects are particularly relevant in view of the association of *S. mutans* with dental caries, where the abilities to produce acids and survive under acidic conditions create an environment that favors tooth demineralization. Unraveling of *S. mutans* signaling pathways will improve the focus of efforts to develop signaling interference strategies for modulating its behavior to reduce biofilm formation or reduce its ability to promote acidic conditions within dental biofilms that may contribute to caries.

## MATERIALS AND METHODS

### Bacteria and growth conditions.

*S. mutans* UA159 and the isogenic mutants used in this study are presented in Table 3. Cultures of *S. mutans* were grown in TSB (Oxoid) at 37°C in 5% CO_2_ and stored at −80°C in TSB supplemented with 15% glycerol.

**TABLE 3:** Strains and plasmid used in this study

Strain or plasmid	Description^a^	Source or reference(s)
*S. mutans* strains		
UA159	WT, transformable strain; Spc^s^ Erm^s^	100, 101
SM065	UA159 Δ*comS::spc*; Spc^r^ (from strain MW05)	6, 102
SM068	UA159::φ (P*_sigX_-luc*); Spc^r^	102
SM059	UA159*::*φ (P*_1914_-luc*); Spc^r^	102
Plasmid pVA838	Erm^r^; replicative streptococcal plasmid	103

a Spc, spectinomycin; Erm, erythromycin.

### Synthetic peptide.

CSP was used in the form of CSP18 (NH_2_-SGSLSTFFRLFNRSFTQA-COOH), synthesized by GenScript (GenScript Corporation, NJ), with a purity of >95%. The lyophilized peptide was reconstituted in distilled water at 175 µg·ml^−1^ and stored in small aliquots at −20°C.

### Transformation.

Transformation experiments were performed in the absence or presence of CSP18 (50 nM). Cultures grown overnight at 37°C in 5% CO_2_ were diluted to an optical density at 600 nm (OD_600_) of 0.04. From this point, incubation proceeded at 37°C in ambient air. Upon reaching an OD_600_ of 0.065, the cultures were distributed into Eppendorf tubes (1.2 ml) and CSP was added to a final concentration of 50 nM. At different times, a 100-µl aliquot was used for OD_600_ measurements, and a 100-µl sample was pelleted and frozen for RNA extraction as described below. Another 100-µl portion was diluted 1:2 in fresh TSB containing replicative plasmid pVA838 DNA (final concentration of 1 µg·ml^−1^). After a 20-min incubation with plasmid DNA, recombinant DNase I (Roche) was added at a final concentration of 10 U·ml^−1^ and incubation proceeded for 40 min before dilution and plating on THB agar with or without erythromycin at a final concentration of 20 µg·ml^−1^. The plates were incubated at 37°C in 5% CO_2_ for 48 h before the counting of visible colonies.

### Real-time PCR.

Bacterial samples for real-time PCRs were collected at 10 and 100 min after the addition of CSP as described above. Total RNA was extracted with the High Pure RNA isolation kit (Roche, Mannheim, Germany) according to the manufacturer’s recommendation, except that the cells were incubated at 37°C for 20 min in 200 µl of lysis buffer containing 10 mM Tris (pH 8), 20 mg of lysozyme ml^−1^, and 100 U of mutanolysin ml^−1^. DNase I was used during RNA extraction to remove the remaining DNA. Complementary DNA templates were prepared from RNA with the Transcriptor First Strand cDNA synthesis kit (Roche Diagnostics GmbH, Mannheim, Germany) in accordance with the manufacturer’s protocol. Controls without reverse transcriptase were included. Expression of *cipB* and *comGA* was examined by real-time PCR with primer pairs FP156 (TGCTCTAGGTGCTGGGCAAG)-FP157 (GAGCTCCTCCGATTCCTCCA), FP166 (ATTGGCAACAAGAGGGAATG)-FP167 (TCTTGCTGACGCAAAACATC), and FP128 (AGAAACCGCCAGAGCTGTTA)-FP129 (CCACGCAAAGCATTTTGTAA), respectively. To normalize the data, primer pair FP299 (CCATGACCATCAACCAACAT)-FP300 (ATCAGCGCGTATTACAGGTG) was used to amplify a portion of *gyrA*. Assays were carried out with quantitative PCR master mix for SYBR green I. Data were collected and compared with the software and graphics program MxPro (Stratagene).

### RNA preparation for microarrays.

RNA samples were from the WT UA159 and the *comS* deletion mutant, grown in the presence or absence of CSP in 100-ml volumes of TSB as described for the transformation assay. WT cultures were collected by centrifugation at 10 and 100 min after CSP addition, and *comS* deletion mutant cultures were collected at 100 min. At each time, samples without CSP were included as controls. Two independent biological replicates were obtained for each condition, giving a total of 12 samples. Immediately after centrifugation (9,000 × *g*, 2.5 min, 4°C), the pellets were frozen in liquid nitrogen. RNA was prepared as previously described, with a few modifications (30). Briefly, the pellets were lysed with mutanolysin-lysozyme, followed by RNA extraction with the mirVana miRNA isolation kit (Ambion). This kit was used to enhance the rate of recovery of short transcripts (down to 10 nucleotides). The samples were then treated with Turbo DNase (Ambion) and analyzed for quality with a Bioanalyzer. Samples with remaining DNA, as determined by PCR with primers for *ccpA* (FP297, [GTAGGTGTGGTTATCCCTAATATTGC] and FP298 [ATAAATCGGCTGACTGATAGATGTC]), were retreated with Turbo DNase, and repurified until no DNA was detected. The MICROBExpress kit (Ambion) was then used for mRNA enrichment. RNA was then fragmented and Cy3 labeled (Mirus Label IT µArray Cy3 labeling kit; Mirus); this was followed by hybridization to the microarray probes. UA159 genomic reference DNA was purified with the DNeasy Blood and Tissue kit (Qiagen), with mutanolysin-lysozyme treatment for the lysis step, followed by fragmentation and labeling (Mirus Label IT µArray Cy3 labeling kit).

### Microarray signal detection, data normalization, and analysis.

The genomic tiling microarray was constructed with probe sets designed from both forward and reverse complement strands of the entire target genome of *S. mutans* UA159 as described by Høvik and Chen (29). A total of 385,000 optimized probes covered the entire genome, including ORFs and intergenic regions. The probes were printed on high-density microarrays by Roche NimbleGen. To block nonspecific binding of RNA molecules, RNase-free bovine serum albumin (500 µg·ml^−1^) was added to the prehybridization solution and salmon sperm DNA (100 to 700 µg·ml^−1^) was included in both prehybridization and hybridization solutions. Hybridization was conducted at a temperature of 42°C in the presence of 25% formamide. NimbleScan v2.5 software was used for spot feature extraction from the scanned images, followed by normalization and analysis as previously described (30). Briefly, the nonspecific background was estimated from the intensity of the intergenic sequence probes and of the genomic DNA reference and used for corrections due to sequence-specific factors (68). Normalization between arrays was done with the vsn algorithm (29). The log_2_ means of the normalized signal intensities from each condition were used for downstream processes.

A Hidden Markov support vector machine (69) was used to identify the boundaries of TARs on the basis of a set of training data derived from both ORFs and intergenic regions. The expression level of annotated genes was determined by averaging the nucleotide intensities of probe signals within the length of the gene (68). Differential expression at the ORF level was measured as the difference between the log_2_ mean probe signal intensities of the control and CSP-treated samples from two independent biological experiments, except for the 10-min sample without CSP, in which one of the hybridizations failed. The *P* values were calculated with the SAM software (68) at default settings by performing 10 permutations for the inclusion of two sets of repeats. Genes that exhibited a >2-fold mean signal intensity difference (with a *P* value of <0.05) were registered as differentially expressed.

### Microarray data and nucleotide sequence accession numbers.

Original and normalized microarray data used in this study were deposited in the NCBI Gene Expression Omnibus database (http://www.ncbi.nlm.nih.gov/geo) under accession no. GSE70067. The transcriptome profiles are also available for browsing at the Microbial Transcriptome Database website (http://bioinformatics.forsyth.org/mtd/dataset=RNAseq_smut_comS).
