# Supplementary material for: Comprehensive Transcriptome Profiles of Streptococcus mutans UA159 Map Core Streptococcal Competence Genes
Source: mSystems. 2016 Apr 12;1(2):e00038-15. doi: 10.1128/mSystems.00038-15 (PMC5069739; doi:10.1128/mSystems.00038-15)
Supplement: Table S1 [file sys002162010st1.pdf]

Table S1. Overview of *S. mutans* genes upregulated by competence inducing pheromones and their expression in deletion mutants.

**Genes induced by CSP/XIP**	[a]**Peptide-rich medium**		[b]**Peptide-free medium**			
	**CSP**		**XIP**		**CSP (not linked to competence)**	
**Bacteriocins & immunity proteins**	**Deletion mutants**	**Expression -[c] / +[d]**	**Deletion mutants**	**Expression**	**Deletion mutants**	**Expression**
nlmAB (SMU.150/151) (1, 2),<br>*cipB* (SMU.1914c) (1, 3),<br>*immB* (SMU.925)(1, 4),<br>*immA* (SMU.1913) (1)<br>*nlmD* (SMU.423) (5),<br>SMU.1906 (5)	Δ*comED*	-				
nlmAB (1), *cipB* (4), *immB* (4)	Δ*sigX*	+				
nlmAB (1), *cipB* (3)	ΔDR *comE*	-				
cipB (6)	Δ*comRS*,Δ*comS*,Δ*sigX*	+	Δ*comE*,Δ*comRS*,Δ*sigX*<br>Δ*comC*	-<br>+	Δ*comED*<br>Δ*comC*,Δ*comRS*,<br>Δ*sigX*,Δ*comS*	-<br>+
immB (7), SMU.1904 (7)	HdrR overexpression<br>HdrR overexpression ΔsigX	+<br>-				
nlmAB, *cipB*, *nlmD*, *bsmB* (8)	Δ*sepM*	-				
**Regulatory genes**						
comE (6)	Δ*comR*, Δ*comS*, Δ*sigX*	-	Δ*comC*, Δ*comD*,<br>Δ*comE*<br>Δ*comRS*,Δ*sigX*	 +<br>-		
comC (3, 8)	Δ*comE*<br>Δ*comD*<br>ΔDR *comE*<br>Δ*sepM*	+<br>-<br>-<br>+				
comS, *sigX* (9)	Δ*comD*, Δ*comE*	-	Δ*comD*, Δ*comE*	+		
sigX (10)			Δ*comR*,Δ*oppD*,<br>Δ*comS*,Δ*comE*	-<br>+		
**Late genes**						
comGA, *comGB*, *comGC*, *comGD*,<br>SMU.1982, SMU.1981, SMU.1980,<br>SMU.1979, SMU.1978 (11)	Δ*comC*, Δ*ciaH*, Δ*luxS*	-				
lytF (6)	Δ*cipB*<br>*comED* and *comRS*<br>overexpression	-<br>+			Δ*cipB*	-
recA (12), *cinA* (12)	Δ*sigX*	-				
comGA, *comEA*, *coiA*, *dprA*,<br>SMU.431, SMU.505, SMU.507,<br>SMU.539, SMU.769, *lytF*, *immB*,<br>SMU.1055, SMU.1904, SMU.1967,<br>*cinA* (7)	HdrR overexpression<br>HdrR overexpression Δ*sigX*	+<br>-				
coiA, *lytF*, SMU.1055, SMU.1967 (7)	Δ*sigX*-box	-				

[a] Rich medium (THB, TSB, or BHI)
[b] Peptide-free medium (chemically defined medium(CDM))
[c] - reduced or abolished
[d] + not affected or increased

References:

1. **van der Ploeg, J. R.** 2005. Regulation of bacteriocin production in *Streptococcus mutans* by the quorum-sensing system required for development of genetic competence. J Bacteriol **187:**3980-9.
2. **Kreth, J., J. Merritt, W. Shi, and F. Qi.** 2005. Co-ordinated bacteriocin production and competence development: a possible mechanism for taking up DNA from neighbouring species. Mol Microbiol **57:**392-404.
3. **Kreth, J., D. C. Hung, J. Merritt, J. Perry, L. Zhu, S. D. Goodman, D. G. Cvitkovitch, W. Shi, and F. Qi.** 2007. The response regulator ComE in *Streptococcus mutans* functions both as a transcription activator of mutacin production and repressor of CSP biosynthesis. Microbiology **153:**1799-807.
4. **Perry, J. A., M. B. Jones, S. N. Peterson, D. G. Cvitkovitch, and C. M. Levesque.** 2009. Peptide alarmone signalling triggers an auto-active bacteriocin necessary for genetic competence. Molecular Microbiology **72:**905-17.
5. **Kreth, J., J. Merritt, L. Zhu, W. Shi, and F. Qi.** 2006. Cell density- and ComE-dependent expression of a group of mutacin and mutacin-like genes in Streptococcus mutans. FEMS Microbiol Lett **265:**11-7.
6. **Reck, M., J. Tomasch, and I. Wagner-Dobler.** 2015. The alternative sigma factor SigX controls bacteriocin synthesis and competence, the two quorum sensing regulated traits in *Streptococcus mutans*. PLoS Genet **11:**e1005353.
7. **Okinaga, T., Z. Xie, G. Niu, F. Qi, and J. Merritt.** 2010. Examination of the *hdrRM* regulon yields insight into the competence system of *Streptococcus mutans*. Mol Oral Microbiol **25:**165-77.
8. **Hossain, M. S., and I. Biswas.** 2012. An extracelluar protease, SepM, generates functional competence-stimulating peptide in *Streptococcus mutans* UA159. J Bacteriol **194:**5886-96.
9. **Wenderska, I. B., N. Lukenda, M. Cordova, N. Magarvey, D. G. Cvitkovitch, and D. B. Senadheera.** 2012. A novel function for the competence inducing peptide, XIP, as a cell death effector of *Streptococcus mutans*. Fems Microbiology Letters **336:**104-12.
10. **Mashburn-Warren, L., D. A. Morrison, and M. J. Federle.** 2010. A novel double-tryptophan peptide pheromone controls competence in Streptococcus spp. via an Rgg regulator. Mol Microbiol **78:**589-606.
11. **Merritt, J., F. Qi, and W. Shi.** 2005. A unique nine-gene *comY* operon in *Streptococcus mutans*. Microbiology **151:**157-66.
12. **Hung, D. C., J. S. Downey, E. A. Ayala, J. Kreth, R. Mair, D. B. Senadheera, F. Qi, D. G. Cvitkovitch, W. Shi, and S. D. Goodman.** 2011. Characterization of DNA binding sites of the ComE response regulator from *Streptococcus mutans*. J Bacteriol **193:**3642-52.
