# Supplementary material for: Comprehensive Transcriptome Profiles of Streptococcus mutans UA159 Map Core Streptococcal Competence Genes
Source: mSystems. 2016 Apr 12;1(2):e00038-15. doi: 10.1128/mSystems.00038-15 (PMC5069739; doi:10.1128/mSystems.00038-15)
Supplement: Table S2 [file sys002162010st2.pdf]

Table S2. Gene expression dynamics during culture growth. Changes in gene expression between 10 and 100 min incubation control cultures without CSP supplementation. Mean fold change for ratios >2 or <-2, and p < 0.05. Up-regulation that has been attributed to CSP supplementation (1) are **in bold**.

Locus	Mean fold change	p value	Annotated function[a]
SMU.30	2.2	0.000	putative phosphoribosylformylglycinamidine synthase, (FGAM synthase)
SMU.31	2.6	0.000	hypothetical protein
SMU.32	2.6	0.000	amidophosphoribosyltransferase
SMU.33	2.2	0.004	hypothetical protein
SMU.34	2.6	0.000	phosphoribosylaminoimidazole synthetase
SMU.35	2.5	0.000	phosphoribosylglycinamide formyltransferase
SMU.36	2.9	0.000	hypothetical protein
SMU.37	2.7	0.000	bifunctional phosphoribosylaminoimidazolecarboxamide formyltransferase
SMU.55	2.4	0.000	hypothetical protein
SMU.56	2.2	0.000	hypothetical protein
SMU.61a*	-2.1	0.002	comS
SMU.103	-2.3	0.000	putative PTS system, IIA component
SMU.104	-2.2	0.000	putative alpha-glucosidase; glycosyl hydrolase
SMU.184	-2.6	0.000	ABC transporter
SMU.185	-3.1	0.000	hypothetical protein
SMU.205c	-2.1	0.002	hypothetical protein
SMU.360	-2.1	0.000	glyceraldehyde-3-phosphate dehydrogenase
**SMU.402**	**-2.1**	**0.000**	**pyruvate formate-lyase**
SMU.459	-2.4	0.000	putative ABC transporter, amino acid binding protein
SMU.460	-3.0	0.000	putative amino acid ABC transporter, permease
SMU.461	-3.1	0.000	putative amino acid ABC transporter, ATP-binding protein
SMU.496	-4.6	0.000	putative cysteine synthetase A; O-acetylserine lyase
SMU.541	-2.1	0.002	hypothetical protein
SMU.545	-2.3	0.190	hypothetical protein
SMU.561c	2.1	0.000	MutT family hydrolase

SMU.589	-2.1	0.000	putative DNA-binding protein
SMU.652c	2.1	0.000	nitrate ABC transporter ATP-binding protein
SMU.674	-2.4	0.000	phosphocarrier protein HPr
SMU.675	-2.3	0.000	phosphoenolpyruvate:sugar phosphotransferase system enzyme I, PTS system EI component
SMU.714	-2.1	0.000	elongation factor Tu
SMU.871	-2.2	0.000	putative fructose-1-phosphate kinase
**SMU.872**	**-2.7**	**0.000**	**putative PTS system, fructose-specific enzyme IIABC component**
**SMU.879**	**-2.1**	**0.000**	**multiple sugar-binding ABC transporter, permease protein MsmF**
**SMU.880**	**-2.3**	**0.000**	**multiple sugar-binding ABC transporter, permease protein MsmG**
**SMU.881**	**-2.2**	**0.000**	**sucrose phosphorylase, GtfA**
**SMU.882**	**-2.1**	**0.000**	**multiple sugar-binding ABC transporter, ATP-binding protein, MsmK**
SMU.932	-3.0	0.000	hypothetical protein
SMU.933	-3.3	0.000	putative amino acid ABC transporter, periplasmic amino acid-binding protein
SMU.934	-2.7	0.000	putative amino acid ABC transporter, permease protein
SMU.935	-2.6	0.000	putative amino acid ABC transporter, permease protein
SMU.936	-2.5	0.000	putative amino acid ABC transporter, ATP-binding protein
SMU.940c	-2.1	0.000	putative hemolysin III
SMU.941c	-2.2	0.000	hypothetical protein
SMU.956	3.6	0.000	putative Clp-like ATP-dependent protease, ATP-binding subunit
SMU.957	-2.3	0.000	50S ribosomal protein L10
SMU.958	-2.1	0.000	hypothetical protein
SMU.960	-2.2	0.000	50S ribosomal protein L7/L12
SMU.961	-4.4	0.000	hypothetical protein
SMU.962	-4.5	0.000	putative dehydrogenase
**SMU.1004**	**2.8**	**0.000**	**glucosyltransferase-I, *gtfB***
SMU.1077	-2.2	0.000	putative phosphoglucomutase
**SMU.1185**	**3.0**	**0.000**	**PTS system, mannitol-specific enzyme IIBC component**
SMU.1334	-2.5	0.000	putative phosphopantetheinyl transferase
SMU.1335c	-2.7	0.000	putative enoyl-(acyl-carrier-protein) reductase
SMU.1336	-2.5	0.000	hypothetical protein
SMU.1337c	-3.0	0.000	alpha/beta superfamily hydrolase
SMU.1338c	-3.1	0.000	multidrug ABC transporter permease
SMU.1339	-2.9	0.000	putative bacitracin synthetase
**SMU.1340**	**-3.6**	**0.000**	**putative surfactin synthetase**

**SMU.1341c**	**-3.4**	**0.000**	**putative gramicidin S synthetase**
**SMU.1342**	**-3.1**	**0.000**	**putative bacitracin synthetase 1; BacA**
**SMU.1343c**	**-2.9**	**0.000**	**putative polyketide synthase**
**SMU.1344c**	**-3.8**	**0.000**	**putative malonyl-CoA acyl-carrier-protein transacylase**
**SMU.1345c**[b]	**-3.8**	**0.000**	**putative peptide synthetase MycA**
**SMU.1346**[b]	**-4.3**	**0.000**	**putative thioesterase BacT**
SMU.1347c	-2.9	0.000	permease
SMU.1348c	-3.3	0.000	putative ABC transporter, ATP-binding protein
SMU.1365c	-2.8	0.000	permease
SMU.1366c	-3.3	0.000	putative ABC transporter; ATP-binding protein
SMU.1395c	3.1	0.002	hypothetical protein
**SMU.1396**	**2.6**	**0.000**	**glucan-binding protein C, GbpC**
SMU.1488c	33.8	0.000	hypothetical protein
SMU.1489	38.5	0.000	LacX
SMU.1490	41.7	0.000	6-phospho-beta-galactosidase
SMU.1491	37.5	0.000	PTS system, lactose-specific enzyme IIBC EIIBC-LAC)
SMU.1492	55.0	0.000	PTS system, lactose-specific enzyme IIA EIIA-LAC)
SMU.1493	49.2	0.000	tagatose 1,6-diphosphate aldolase
SMU.1494	47.1	0.000	tagatose-6-phosphate kinase
SMU.1495	55.1	0.000	galactose-6-phosphate isomerase subunit LacB
SMU.1496	54.5	0.000	galactose-6-phosphate isomerase subunit LacA
SMU.1498	3.8	0.000	lactose repressor
SMU.1657c	3.0	0.000	putative nitrogen regulatory protein PII
SMU.1658	2.2	0.000	putative ammonium transporter, NrgA protein
SMU.1792c	2.1	0.000	hypothetical protein
**SMU.1877**	**-2.4**	**0.000**	**putative PTS system, mannose-specific component IIAB**
**SMU.1878**	**-2.6**	**0.000**	**putative PTS system, mannose-specific component IIC**
**SMU.1879**	**-2.6**	**0.000**	**putative PTS system, mannose-specific component IID**
SMU.1941	-2.2	0.000	putative membrane lipoprotein
SMU.1942c	-4.6	0.000	putative amino acid binding protein
**SMU.1956c**	**-6.5**	**0.000**	**hypothetical protein**
**SMU.1957**	**-6.0**	**0.000**	**putative PTS system, mannose-specific IID component**
**SMU.1958c**	**-6.0**	**0.000**	**putative PTS system, mannose-specific IIC component**
**SMU.1960c**	**-5.8**	**0.000**	**putative PTS system, mannose-specific IIB component**
**SMU.1961c**	**-5.9**	**0.000**	**putative PTS system, sugar-specific enzyme IIA component**
SMU.2004	-2.1	0.000	translation initiation factor IF-1
**SMU.2037**	**4.7**	**0.000**	**putative trehalose-6-phosphate hydrolase TreA**
**SMU.2038**	**4.2**	**0.000**	**putative PTS system, trehalose-specific IIABC component**

SMU.2133c	3.4	0.000	hypothetical protein

[a] GenBank annotation (Accession AE014133)
[b] Expression change observed also in response to CSP.
*SMU.61a is provisionally used to identify *comS* in the transcriptome maps at http://bioinformatics.forsyth.org/. *comS* is not yet annotated in GenBank.

References

1. **Perry, J. A., M. B. Jones, S. N. Peterson, D. G. Cvitkovitch, and C. M. Levesque.** 2009. Peptide alarmone signalling triggers an auto-active bacteriocin necessary for genetic competence. Molecular Microbiology **72:**905-17.
