# Supplementary material for: Comprehensive Transcriptome Profiles of Streptococcus mutans UA159 Map Core Streptococcal Competence Genes
Source: mSystems. 2016 Apr 12;1(2):e00038-15. doi: 10.1128/mSystems.00038-15 (PMC5069739; doi:10.1128/mSystems.00038-15)
Supplement: Table S3 [file sys002162010st3.pdf]

Table S3. Effect of CSP on gene expression. Mean fold change and P value for all ORFs.

Gene ID	Mean fold change[a]			p value			Annotation
	UA159 10 min	UA159 100 min	Δ*comS* 100 min	UA159 10 min	UA159 100 min	Δ*comS* 100 min	
SMU.01	-1,1	-1,1	1,1	0,117	0,293	0,176	chromosomal replication initiation protein
SMU.02	-1,2	-1,1	1,2	**0,048**	0,111	0,143	DNA polymerase III subunit beta
SMU.05	1,1	1,1	-1,2	0,501	0,501	0,305	hypothetical protein
SMU.06	1,1	-1,0	1,0	0,170	0,577	0,740	GTP-dependent nucleic acid-binding protein EngD
SMU.07	1,1	-1,1	1,2	0,601	0,583	0,235	peptidyl-tRNA hydrolase
SMU.08	-1,0	-1,0	1,2	0,906	0,476	**0,016**	putative transcription-repair coupling factor
SMU.09	-1,1	-1,1	1,6	0,720	0,675	**0,036**	hypothetical protein
SMU.10	-1,0	-1,5	1,9	0,780	**0,002**	**0,000**	hypothetical protein
SMU.11	-1,3	-1,3	-1,1	0,576	0,436	0,823	hypothetical protein
SMU.12	1,0	-1,1	1,4	0,853	0,248	**0,001**	hypothetical protein
SMU.13	-1,1	-1,0	1,2	0,574	0,824	0,145	putative cell-cycle protein
SMU.14	1,1	1,1	1,2	0,610	0,542	0,202	hypoxanthine-guanine phosphoribosyltransferase
SMU.15	1,3	1,1	1,2	**0,005**	0,155	0,100	putative cell division protein FtsH
SMU.16	-1,2	-1,1	-1,0	0,232	0,396	0,922	putative amino acid permease
SMU.18	1,1	-1,1	1,0	0,920	0,876	0,995	hypothetical protein
SMU.20	1,1	-1,1	1,0	0,620	0,662	0,913	putative cell shape-determining protein MreC
SMU.21	1,3	1,4	1,8	**0,040**	**0,007**	**0,000**	putative cell shape-determining protein MreD
SMU.22	1,4	1,3	1,6	**0,000**	**0,020**	**0,000**	putative secreted antigen GbpB/SagA; putative peptidoglycan hydrolase
SMU.23	1,0	1,0	-1,1	0,794	0,938	0,621	ribose-phosphate pyrophosphokinase
SMU.24	-1,1	1,0	-1,1	0,512	0,785	0,233	aromatic amino acid aminotransferase
SMU.25	-1,2	-1,1	1,0	0,128	0,610	0,835	DNA repair protein RecO
SMU.26	1,0	-1,2	-1,2	0,650	0,106	0,089	putative glycerol-3-phosphate acyltransferase PlsX
SMU.27	1,1	-1,3	-1,1	0,546	**0,043**	0,377	acyl carrier protein
SMU.28	-1,0	-1,0	-1,3	0,950	0,851	**0,000**	putative ATP-binding protein
SMU.29	-1,0	-1,0	-1,1	0,973	0,860	0,183	phosphoribosylaminoimidazole-succinocarboxamide synthase
SMU.30	1,0	-1,0	-1,2	0,412	0,905	**0,000**	putative phosphoribosylformylglycinamidine synthase, (FGAM synthase)
SMU.31	-1,1	-1,1	-1,2	0,740	0,618	0,146	hypothetical protein
SMU.32	1,1	1,0	-1,1	0,212	0,464	0,070	amidophosphoribosyltransferase
SMU.33	-1,1	1,1	-1,5	0,714	0,716	**0,038**	hypothetical protein
SMU.34	1,2	1,2	-1,2	**0,044**	**0,014**	**0,002**	phosphoribosylaminoimidazole synthetase
SMU.35	1,2	1,2	-1,1	0,098	0,076	0,212	phosphoribosylglycinamide formyltransferase
SMU.36	1,1	1,4	-1,5	0,164	**0,000**	**0,000**	hypothetical protein
SMU.37	1,2	1,2	-1,0	**0,007**	**0,000**	0,396	bifunctional phosphoribosylaminoimidazolecarboxamide formyltransferase/IMP cyclohydrolase
SMU.38c	1,0	1,1	1,1	0,984	0,059	0,141	putative transcriptional regulator
SMU.39	-1,2	-1,3	-1,1	0,194	**0,007**	0,306	hypothetical protein
SMU.40	1,1	1,0	-1,1	0,689	0,765	0,336	hypothetical protein
SMU.41	-1,2	-1,2	1,1	0,585	0,676	0,897	hypothetical protein
SMU.42	1,0	-1,1	-1,0	0,867	0,579	0,814	hypothetical protein
SMU.43	1,0	-1,1	1,2	0,789	**0,021**	**0,002**	putative site-specific DNA-methyltransferase restriction-modification protein
SMU.44	-1,0	-1,5	1,2	0,922	**0,000**	0,216	DNA mismatch repair protein
SMU.45	-1,1	-1,2	1,2	**0,043**	**0,031**	**0,002**	hypothetical protein
SMU.46	-1,0	-1,2	1,2	0,713	0,073	0,057	hypothetical protein
SMU.47	-1,4	-1,3	1,1	**0,021**	**0,042**	0,720	hypothetical protein
SMU.48	1,2	-1,0	-1,2	**0,004**	0,573	**0,004**	phosphoribosylamine--glycine ligase
SMU.49	1,1	-1,0	-1,2	0,165	0,668	**0,025**	hypothetical protein
SMU.50	1,2	1,0	-1,0	0,093	0,990	0,877	phosphoribosylaminoimidazole carboxylase catalytic subunit
SMU.51	1,4	1,0	1,0	**0,001**	0,930	0,800	phosphoribosylaminoimidazole carboxylase ATPase subunit
SMU.52	1,0	-1,2	-1,0	0,837	0,189	0,787	hypothetical protein
SMU.53	1,2	-1,1	1,1	**0,021**	0,233	**0,027**	hypothetical protein
SMU.54	1,3	1,0	1,1	**0,000**	0,753	0,135	putative amino acid recemase
SMU.55	1,5	1,2	1,1	**0,000**	0,107	0,522	hypothetical protein
SMU.56	1,4	1,2	-1,1	**0,012**	0,151	0,292	hypothetical protein

SMU.58	1,1	-1,0	-1,0	0,487	0,975	0,587	hypothetical protein
SMU.59	1,4	1,2	1,0	**0,000**	**0,001**	0,400	adenylosuccinate lyase
SMU.60	1,0	1,6	-1,1	0,838	**0,000**	0,213	DNA alkylation repair enzyme
SMU.61	-1,1	1,4	-1,1	0,550	**0,008**	0,624	putative transcriptional regulator
SMU.63c	1,4	-2,3	1,7	**0,000**	**0,000**	**0,000**	hypothetical protein
SMU.64	1,2	**5,6**	-1,2	**0,045**	**0,000**	0,079	Holliday junction DNA helicase RuvB
SMU.65	1,2	**4,4**	-1,1	0,228	**0,000**	0,741	putative protein tyrosine-phosphatase
SMU.66	1,1	**2,6**	-1,2	0,528	**0,000**	**0,022**	hypothetical protein
SMU.67	-1,0	**2,3**	-1,1	0,759	**0,000**	0,174	putative acyltransferase
SMU.68	1,5	**2,3**	-1,2	**0,000**	**0,000**	**0,017**	hypothetical protein
SMU.70	1,1	-1,2	-1,2	0,417	0,108	0,080	threonine synthase
SMU.71	1,2	-1,0	-1,4	0,114	0,685	**0,001**	putative cation efflux pump (multidrug resistance protein)
SMU.72	1,0	-1,2	-1,5	0,935	0,425	0,081	hypothetical protein
SMU.73	1,2	-1,1	-1,2	**0,007**	0,182	**0,003**	hypothetical protein
SMU.74	1,2	-1,0	-1,1	0,061	0,810	0,365	hypothetical protein
SMU.75	1,2	-1,0	-1,1	**0,041**	0,807	0,232	putative D-alanyl-D-alanine carboxypeptidase
SMU.76	1,0	-1,0	-1,1	0,691	0,965	0,164	putative N-acetyl-muramidase
SMU.78	-1,0	1,0	1,1	0,194	0,537	0,131	fructan hydrolase; exo-beta-D-fructosidase; fructanase, FruA
SMU.79	1,1	1,1	-1,2	**0,045**	**0,018**	**0,007**	fructan hydrolase; exo-beta-D-fructosidase; FruB
SMU.80	1,1	1,4	1,1	0,540	**0,006**	0,586	heat-inducible transcription repressor
SMU.81	1,4	1,5	-1,2	**0,002**	**0,000**	0,108	heat shock protein GrpE
SMU.82	1,2	1,4	1,2	**0,000**	**0,000**	**0,001**	molecular chaperone DnaK
SMU.83	1,5	1,5	1,0	**0,000**	**0,000**	0,750	chaperone protein DnaJ
SMU.84	1,3	-1,0	-1,1	**0,004**	0,967	0,159	tRNA pseudouridine synthase A
SMU.85	1,3	-1,0	-1,2	**0,005**	0,681	**0,031**	phosphomethylpyrimidine kinase
SMU.86	1,3	-1,2	1,1	0,086	0,324	0,476	hypothetical protein
SMU.87	1,3	-1,1	-1,1	**0,000**	0,366	**0,048**	hypothetical protein
SMU.88c	-1,2	-1,1	1,1	0,200	0,587	0,359	mechanosensitive ion channel
SMU.89c	-1,3	-1,1	-1,0	**0,003**	0,185	0,797	putative nitrite transporter
SMU.91	1,3	-1,2	1,3	**0,000**	**0,021**	**0,003**	trigger factor
SMU.92c	-1,5	-1,2	-1,4	**0,000**	0,117	**0,000**	putative putative transposase
SMU.93c	1,2	1,3	1,2	**0,002**	**0,003**	**0,002**	putative putative transposase
SMU.94c	-1,1	1,1	1,1	0,152	**0,000**	0,091	putative putative transposase
SMU.96	1,3	-1,0	1,0	**0,019**	0,690	0,879	DNA-directed RNA polymerase subunit delta
SMU.97	1,2	1,1	1,0	**0,001**	0,158	0,905	CTP synthetase
SMU.99	1,1	-1,0	1,1	0,459	0,837	0,285	fructose-bisphosphate aldolase
SMU.100	-1,3	1,0	-1,0	0,056	0,885	0,866	putative sorbose PTS system, IIB component
SMU.101	-1,3	-1,0	-1,2	0,066	0,939	0,341	putative sorbose PTS system, IIC component
SMU.102	-1,3	-1,0	1,0	**0,001**	0,617	0,808	putative PTS system, IID component
SMU.103	1,1	1,0	1,1	0,696	0,882	0,698	putative PTS system, IIA component
SMU.104	-1,1	-1,0	1,1	0,063	0,672	0,310	putative alpha-glucosidase; glycosyl hydrolase
SMU.105	-1,2	-1,1	1,1	0,070	0,430	0,420	putative transcriptional regulator; repressor of sugar transport operon
SMU.106c	1,2	-1,2	1,2	**0,000**	**0,000**	**0,002**	putative putative transposase
SMU.107	1,0	-1,5	1,2	0,674	0,056	0,218	hypothetical protein
SMU.108	1,1	1,8	-1,1	0,419	**0,004**	0,312	hypothetical protein
SMU.109	-1,0	**2,2**	-1,0	0,844	**0,000**	0,990	permease (efflux protein)
SMU.110	-1,1	1,1	1,5	0,646	0,585	**0,000**	putative transcriptional regulator MutR
SMU.112c	-1,1	1,1	1,4	**0,005**	0,131	**0,000**	putative transcriptional regulator
SMU.113	-1,0	1,1	-1,1	0,562	0,341	0,179	putative fructose-1-phosphate kinase
SMU.114	-1,1	1,1	-1,1	0,210	0,301	0,131	putative PTS system, fructose-specific IIBC component
SMU.115	1,0	1,1	-1,0	0,473	0,224	0,954	putative PTS system, fructose-specific IIA component
SMU.116	1,0	1,2	-1,1	0,984	**0,011**	0,383	tagatose 1,6-diphosphate aldolase
SMU.117c	-1,1	1,0	-1,1	**0,047**	0,944	0,053	hypothetical protein
SMU.118c	1,0	1,0	1,0	0,597	0,801	0,660	putative esterase
SMU.119	1,1	1,1	-1,1	0,166	0,430	0,239	putative alcohol dehydrogenase class III
SMU.120	1,4	-1,0	1,2	0,138	0,879	0,462	50S ribosomal protein L28
SMU.121	-1,1	1,1	-1,1	**0,033**	**0,005**	**0,015**	putative DinF, damage-inducible protein; cation efflux pump (multidrug resistance protein)
SMU.123	1,2	1,2	-1,1	**0,001**	**0,000**	**0,009**	DNA polymerase III PolC
SMU.124	1,4	1,2	-1,3	**0,002**	**0,043**	**0,012**	MarR family transcriptional regulator
SMU.125	1,1	1,0	-1,2	0,394	0,726	0,202	hypothetical protein
SMU.127	1,3	-1,2	-1,1	0,059	0,230	0,626	putative acetoin dehydrogenase (TPP-dependent), E1 component alpha subunit

SMU.128	1,3	-1,2	-1,0	**0,004**	**0,026**	0,580	putative acetoin dehydrogenase (TPP-dependent), E1 component beta subunit
SMU.129	1,3	-1,2	-1,1	**0,002**	**0,022**	0,141	branched-chain alpha-keto acid dehydrogenase subunit E2
SMU.130	1,3	-1,1	-1,0	**0,000**	**0,006**	0,874	putative dihydrolipoamide dehydrogenase
SMU.131	1,1	-1,2	-1,0	0,103	**0,049**	0,714	putative lipoate-protein ligase
SMU.132	1,2	-1,1	-1,1	**0,025**	0,212	0,134	putative hippurate amidohydrolase
SMU.133c	-1,1	1,0	-1,0	0,074	0,675	0,719	putative MDR permease; transmembrane efflux protein
SMU.134	-1,1	-1,1	-1,0	0,300	0,276	0,967	TetR/AcrR family transcriptional regulator
SMU.135	-1,0	1,0	1,4	0,840	0,860	**0,008**	putative transcriptional regulator
SMU.136c	1,1	-1,2	1,1	0,373	0,251	0,234	putative transcriptional regulator
SMU.137	1,1	-1,0	-1,0	0,089	0,699	0,502	malate dehydrogenase
SMU.138	1,1	-1,1	1,0	**0,029**	0,324	0,700	putative malate permease
SMU.139	1,1	-1,0	-1,2	0,081	0,892	**0,034**	hypothetical protein
SMU.140	1,3	1,1	-1,2	**0,000**	0,068	**0,009**	putative glutathione reductase
SMU.141	1,1	-1,0	-1,1	0,231	0,868	0,292	hypothetical protein
SMU.143c	1,1	1,1	1,1	0,282	0,369	0,561	peptide deformylase
SMU.144c	-1,1	1,0	-1,1	0,378	0,971	0,231	putative transcriptional regulator
SMU.145	-1,1	-1,2	1,1	0,384	**0,034**	0,244	hypothetical protein
SMU.148	-1,1	1,6	-1,2	**0,008**	**0,000**	**0,003**	bifunctional acetaldehyde-CoA/alcohol dehydrogenase
SMU.149	1,2	1,1	-1,0	0,079	0,261	0,938	putative transposase
SMU.150	**51,8**	**62,4**	**40,9**	**0,000**	**0,000**	**0,000**	hypothetical protein
SMU.151	**34,7**	**41,1**	**46,2**	**0,000**	**0,000**	**0,000**	hypothetical protein
SMU.152	**22,4**	**36,4**	**42,0**	**0,000**	**0,000**	**0,000**	hypothetical protein
SMU.153	**17,6**	**25,9**	**25,6**	**0,000**	**0,000**	**0,000**	hypothetical protein
SMU.154	1,6	1,5	1,5	**0,000**	**0,000**	**0,000**	30S ribosomal protein S15
SMU.155	1,1	-1,2	-1,2	**0,047**	**0,001**	**0,000**	polynucleotide phosphorylase/polyadenylase
SMU.156	-1,3	-1,5	-1,4	**0,043**	**0,000**	**0,008**	hypothetical protein
SMU.157	1,3	-1,1	-1,3	**0,001**	0,286	**0,000**	putative serine acetyltransferase; serine O-acetyltransferase
SMU.158	1,0	-1,2	-1,3	0,918	0,093	**0,007**	cysteinyl-tRNA synthetase
SMU.159	1,2	-1,0	-1,3	0,170	0,746	**0,019**	hypothetical protein
SMU.160	1,1	1,1	-1,1	0,385	0,325	0,169	metallopeptidase
SMU.161	-1,0	1,1	-1,1	0,908	0,289	0,329	putative transcriptional regulator
SMU.162c	-1,0	-1,1	1,3	0,665	0,298	**0,002**	hypothetical protein
SMU.163c	1,0	1,0	1,1	0,723	0,847	0,261	hypothetical protein
SMU.164	1,0	-1,0	-1,2	0,649	0,703	0,065	putative tRNA/rRNA methyltransferase
SMU.165	1,0	-1,2	-1,3	0,999	**0,018**	**0,000**	hypothetical protein
SMU.166	-1,1	**3,7**	-1,2	0,568	**0,000**	0,247	hypothetical protein
SMU.167	-1,1	**2,6**	-1,2	0,424	**0,000**	0,155	hypothetical protein
SMU.168	1,0	**3,5**	-1,5	0,859	**0,000**	**0,001**	putative transcriptional regulator
SMU.169	1,2	1,0	1,4	**0,027**	0,831	**0,003**	50S ribosomal protein L13
SMU.170	1,5	1,1	1,4	**0,000**	0,289	**0,001**	30S ribosomal protein S9
SMU.172	-1,2	-1,4	-1,3	0,200	0,079	0,180	putative cell growth regulatory protein
SMU.173	1,1	1,0	-1,2	0,800	0,874	0,252	putative ppGpp-regulated growth inhibitor
SMU.174c	1,0	1,0	1,2	0,992	0,627	**0,042**	hypothetical protein
SMU.175	-1,2	1,0	-1,1	**0,027**	0,391	0,505	hypothetical protein
SMU.176	-1,0	1,1	-1,2	0,902	0,728	0,178	hypothetical protein
SMU.177	-1,1	-1,3	-1,0	0,554	0,059	0,962	hypothetical protein
SMU.178	-1,1	-1,2	-1,1	0,229	0,147	0,345	hypothetical protein
SMU.179	-1,2	1,3	-1,3	**0,006**	**0,000**	**0,000**	hypothetical protein
SMU.180	-1,0	1,2	-1,1	0,974	**0,001**	0,052	putative oxidoreductase; fumarate reductase
SMU.181	1,1	-1,0	-1,4	0,247	0,912	**0,006**	putative mevalonate kinase
SMU.182	-2,2	-1,1	-1,5	**0,000**	0,613	**0,022**	iron/manganese ABC transporter ATP-binding protein
SMU.183	-1,8	-1,1	-1,2	**0,000**	0,701	0,261	putative Mn/Zn ABC transporter
SMU.184	-1,3	-1,0	-1,1	**0,010**	0,843	0,613	ABC transporter
SMU.185	-1,2	-1,3	1,3	0,278	0,267	0,384	hypothetical protein
SMU.186	-1,1	1,1	-1,1	0,555	0,396	0,255	putative metal-dependent transcriptional regulator
SMU.187c	1,2	-1,1	-1,0	0,061	0,204	0,675	hypothetical protein
SMU.188c	1,2	-1,1	-1,1	0,240	0,517	0,461	Hsp33-like chaperonin
SMU.189	1,3	-1,0	-1,3	0,484	0,950	0,571	hypothetical protein
SMU.191c	-1,3	-1,1	1,5	**0,001**	0,502	**0,000**	putative integrase
SMU.193c	-1,2	1,2	1,2	0,050	0,067	0,072	hypothetical protein
SMU.194c	-1,2	-1,0	1,4	0,289	0,956	**0,022**	bacteriophage P2 associated
SMU.195c	-1,2	1,1	-1,1	0,438	0,619	0,748	hypothetical protein
SMU.196c	-1,0	1,2	1,1	0,625	**0,000**	0,178	putative transfer protein

SMU.197c	-1,2	1,1	1,1	**0,004**	0,212	0,279	hypothetical protein
SMU.198c	-1,1	1,1	1,2	**0,040**	0,393	**0,015**	putative conjugative transposon protein
SMU.199c	-1,2	1,1	1,1	0,318	0,665	0,685	hypothetical protein
SMU.200c	-1,0	1,1	1,3	0,931	0,686	0,219	hypothetical protein
SMU.201c	-1,1	1,0	1,2	0,303	0,855	0,069	putative transposon protein
SMU.202c	-1,1	1,0	1,2	0,487	0,782	0,241	hypothetical protein
SMU.204c	1,1	1,1	1,3	0,432	0,494	0,081	hypothetical protein
SMU.205c	-1,2	1,0	1,1	0,443	0,978	0,725	hypothetical protein
SMU.206c	-1,0	-1,0	1,3	0,969	0,892	0,063	hypothetical protein
SMU.207c	1,0	1,0	1,0	0,995	0,653	0,889	putative transposon protein
SMU.208c	-1,1	1,0	1,1	0,508	0,618	0,188	putative transposon protein; DNA segregation ATPase
SMU.209c	-1,1	1,0	1,4	0,596	0,911	0,150	hypothetical protein
SMU.210c	-1,2	1,0	1,0	0,310	0,905	0,848	hypothetical protein
SMU.211c	1,1	1,2	1,2	0,683	0,276	0,336	hypothetical protein
SMU.212c	1,0	-1,1	1,4	0,838	0,765	0,055	hypothetical protein
SMU.213c	1,0	1,1	1,2	0,845	0,436	0,179	hypothetical protein
SMU.214c	-1,1	-1,0	1,2	0,474	0,869	0,188	hypothetical protein
SMU.215c	1,1	1,0	1,4	0,732	0,790	**0,041**	hypothetical protein
SMU.216c	1,1	-1,0	1,3	0,785	0,869	0,237	hypothetical protein
SMU.217c	1,0	-1,0	1,5	0,979	0,917	**0,046**	hypothetical protein
SMU.218	-1,3	-1,0	1,1	0,170	0,927	0,585	putative transcriptional regulator
SMU.219	-1,1	-1,1	1,3	0,115	0,288	**0,002**	hypothetical protein
SMU.220c	1,0	-1,2	-1,0	0,922	0,343	0,996	hypothetical protein
SMU.221c	1,5	-1,3	1,3	0,069	0,271	0,227	putative integrase
SMU.222c	1,2	-1,2	-1,2	0,560	0,440	0,598	integrase fragment
SMU.223c	1,0	1,2	1,2	0,786	0,218	0,282	hypothetical protein
SMU.224c	1,2	-1,3	1,0	0,306	0,071	0,905	hypothetical protein
SMU.225c	-1,0	-1,6	1,4	0,929	0,146	0,284	hypothetical protein
SMU.226c	1,4	-1,2	1,2	**0,000**	**0,024**	**0,013**	putative transposase
SMU.227c	1,2	-1,2	1,1	0,354	0,146	0,535	hypothetical protein
SMU.228	1,1	-1,0	-1,2	0,233	0,997	0,060	putative alkaline-shock protein
SMU.229	1,3	1,1	-1,1	**0,001**	0,457	0,262	hypothetical protein
SMU.231	1,1	-1,2	-1,1	0,292	0,081	0,295	acetolactate synthase catalytic subunit
SMU.232	1,1	-1,2	-1,0	0,214	0,056	0,896	acetolactate synthase 3 regulatory subunit
SMU.233	1,2	-1,2	-1,0	**0,018**	0,059	0,611	ketol-acid reductoisomerase
SMU.234	1,3	-1,0	-1,1	**0,004**	0,709	0,340	threonine dehydratase
SMU.235	1,1	1,0	1,0	0,379	0,781	0,721	hypothetical protein
SMU.236c	-1,3	-1,2	1,2	**0,027**	0,170	0,193	putative transcriptional regulator
SMU.237c	-1,1	1,0	1,0	0,208	0,630	0,513	putative integral membrane protein
SMU.238c	-1,1	1,1	-1,1	0,079	0,155	0,081	putative ABC transporter, ATP-binding protein
SMU.239c	-1,0	-1,2	1,2	0,882	0,142	0,096	hypothetical protein
SMU.241c	-1,1	-1,3	1,1	0,137	**0,003**	0,156	putative ABC transporter, ATP-binding protein; amino acid transport system
SMU.242c	-1,2	-1,2	1,1	**0,022**	**0,001**	0,127	putative amino acid ABC transporter, permease protein, glutamine transport system
SMU.243	-1,0	-1,2	-1,3	0,984	0,077	**0,009**	hypothetical protein
SMU.244	-1,2	-1,2	-1,3	**0,023**	**0,010**	**0,001**	undecaprenyl pyrophosphate phosphatase
SMU.245	1,4	1,2	-1,1	**0,003**	0,113	0,432	adaptor protein
SMU.246	1,1	1,1	1,1	0,615	0,556	0,605	putative glycosyl transferase N-acetylglucosaminyltransferase), RgpG
SMU.247	1,2	-1,0	1,0	0,112	0,649	0,755	putative ABC transporter, ATP-binding protein
SMU.248	1,3	-1,0	-1,1	**0,000**	0,913	**0,048**	putative ABC transporter, membrane protein
SMU.249	1,3	-1,1	-1,1	**0,008**	0,527	0,406	putative NifS protein homologue, class-V aminotransferase
SMU.250	1,2	-1,1	-1,2	**0,004**	0,341	**0,003**	putative nitrogen fixation-like protein, NifU
SMU.251	1,3	-1,1	-1,0	**0,008**	0,293	0,796	ABC transporter membrane protein
SMU.252	-1,2	-1,1	-1,2	0,050	0,107	**0,049**	hypothetical protein
SMU.253	-1,0	-1,1	-1,0	0,589	0,417	0,783	putative D-alanyl-D-alanine carboxypeptidase; penicillin-binding protein
SMU.255	1,0	-1,1	1,1	0,743	0,410	0,199	putative oligopeptide ABC transporter, substrate-binding protein OppA
SMU.256	-1,0	-1,1	1,0	0,901	0,548	0,947	putative oligopeptide transport system, permease protein OppB
SMU.257	1,0	-1,2	-1,1	0,903	0,137	0,363	putative transmembrane protein, permease OppC
SMU.258	-1,0	-1,1	-1,0	0,981	0,339	0,555	putative oligopeptide ABC transporter, ATP-binding protein OppD

SMU.259	1,1	-1,0	-1,1	0,188	0,910	0,360	putative oligopeptide ABC transporter, ATP-binding protein OppF
SMU.260	-1,0	-1,0	-1,1	0,652	0,689	0,143	hypothetical protein
SMU.261c	-1,1	-1,0	1,0	0,265	0,445	0,791	putative transcriptional regulator
SMU.262	-1,0	1,0	-1,1	0,117	0,286	0,166	putrescine carbamoyltransferase
SMU.263	1,1	1,1	1,0	0,215	0,165	0,362	putative amino acid antiporter
SMU.264	-1,0	1,2	-1,2	0,696	**0,000**	**0,000**	agmatine deiminase
SMU.265	-1,0	1,3	-1,2	0,811	**0,000**	**0,001**	carbamate kinase
SMU.267c	-1,1	-1,0	1,0	0,222	0,614	0,748	bifunctional glutamate--cysteine ligase/glutathione synthetase
SMU.268	1,2	-1,1	-1,2	0,090	0,140	0,062	adenylosuccinate synthetase
SMU.270	1,0	-1,2	-1,1	0,834	**0,025**	0,096	PTS system ascorbate-specific transporter subunit IIC
SMU.271	1,2	-1,2	-1,4	0,050	**0,046**	**0,004**	putative PTS system, enzyme IIB component
SMU.272	1,1	-1,2	-1,2	0,496	0,058	**0,046**	putative PTS system, enzyme IIA component
SMU.273	1,2	-1,2	-1,4	**0,049**	**0,005**	**0,000**	3-keto-L-gulonate-6-phosphate decarboxylase
SMU.274	1,0	-1,2	-1,1	0,626	**0,016**	0,073	putative L-xylulose 5-phosphate 3-epimerase
SMU.275	1,0	-1,2	-1,3	0,533	**0,012**	**0,000**	L-ribulose-5-phosphate 4-epimerase
SMU.276c	-1,4	1,1	-1,2	**0,002**	0,528	0,265	hypothetical protein
SMU.277	1,5	-1,1	-1,3	**0,003**	0,254	0,089	hypothetical protein
SMU.278	1,4	-1,3	-1,0	**0,016**	0,145	0,751	hypothetical protein
SMU.279	-1,8	-1,2	1,1	**0,000**	**0,005**	0,587	hypothetical protein
SMU.281	1,3	-1,1	-1,4	0,108	0,469	**0,028**	hypothetical protein
SMU.283	1,3	-1,0	-1,1	**0,010**	0,690	0,200	hypothetical protein
SMU.284	-1,1	-1,4	-1,0	0,645	**0,017**	0,824	hypothetical protein
SMU.285	-1,3	-2,1	-1,1	0,274	**0,018**	0,618	hypothetical protein
SMU.286	1,1	-1,2	1,1	0,349	**0,008**	0,357	putative ABC transporter, ATP-binding protein ComA
SMU.287	1,1	-1,4	1,2	0,545	**0,001**	**0,046**	putative ComB, accessory factor for ComA
SMU.289	-1,0	-1,2	1,0	0,496	**0,002**	0,768	putative transcriptional regulator
SMU.290	1,0	-1,0	-1,1	0,542	0,786	0,146	putative L-ascorbate 6-phosphate lactonase
SMU.291	1,1	-1,1	-1,1	**0,036**	0,295	0,234	transketolase
SMU.292	-1,0	1,1	-1,2	0,905	0,305	**0,004**	putative transcriptional regulator
SMU.293	-1,1	-1,0	-1,4	0,220	0,844	**0,006**	hypothetical protein
SMU.294	-1,0	-1,0	-1,3	0,901	0,736	**0,034**	hypothetical protein
SMU.295	-1,0	1,0	-1,1	0,863	0,920	0,175	hypothetical protein
SMU.296	1,1	1,1	-1,3	0,232	0,291	**0,001**	hypothetical protein
SMU.297	1,1	-1,0	-1,1	0,078	0,478	0,273	DNA polymerase I
SMU.298	1,2	-1,0	-1,2	0,230	0,686	0,217	hypothetical protein
SMU.299c	1,2	-1,1	-1,1	0,211	0,443	0,362	putative bacteriocin peptide precursor
SMU.300	1,1	1,1	-1,0	0,117	0,123	0,829	queuine tRNA-ribosyltransferase
SMU.301	1,2	1,1	-1,4	**0,033**	0,162	**0,000**	hypothetical protein
SMU.302	-1,2	1,0	1,2	0,065	0,800	0,089	hypothetical protein
SMU.303	1,0	1,1	1,0	0,973	0,102	0,986	hypothetical protein
SMU.304	1,2	1,0	-1,1	0,077	0,534	0,112	putative deaminase
SMU.305	-1,1	-1,0	1,0	0,378	0,771	0,931	hypothetical protein
SMU.307	1,1	1,2	1,0	0,259	**0,017**	0,971	glucose-6-phosphate isomerase
SMU.308	-1,0	1,1	-1,3	0,459	0,219	**0,000**	sorbitol-6-phosphate 2-dehydrogenase
SMU.309	-1,1	-1,1	-1,1	0,055	0,235	0,157	regulator of sorbitol operon
SMU.310	1,0	1,0	-1,0	0,871	0,871	0,737	sorbitol operon activator
SMU.311	-1,0	1,2	-1,1	0,979	0,151	0,524	PTS system, sorbitol (glucitol) phosphotransferase enzyme IIC2
SMU.312	1,0	1,1	-1,2	0,652	0,374	**0,011**	PTS system, sorbitol phosphotransferase enzyme IIBC
SMU.313	1,1	1,2	-1,2	0,343	0,124	0,065	putative PTS system, sorbitol-specific enzyme IIA
SMU.314	1,1	1,0	-1,1	0,060	0,545	0,317	hypothetical protein
SMU.317	1,4	1,0	-1,0	**0,014**	0,941	0,893	putative tetrahydrodipicolinate succinylase
SMU.318	1,1	-1,0	-1,0	0,209	0,906	0,569	putative hippurate hydrolase
SMU.320	1,0	1,0	-1,3	0,886	0,838	0,082	putative 5-formyltetrahydrofolate cyclo-ligase
SMU.321	1,0	-1,0	-1,1	0,676	0,770	0,207	hypothetical protein
SMU.322c	1,0	-1,0	1,1	0,969	0,785	0,267	glucose-1-phosphate uridylyltransferase
SMU.323	1,3	1,1	1,1	**0,027**	0,207	0,223	NAD(P)H-dependent glycerol-3-phosphate dehydrogenase
SMU.325	1,3	**3,0**	-1,3	0,062	**0,000**	**0,048**	deoxyuridine 5'-triphosphate nucleotidohydrolase
SMU.326	1,1	**3,3**	-1,3	0,391	**0,000**	**0,009**	hypothetical protein
SMU.327	1,3	**3,1**	-1,2	**0,001**	**0,000**	**0,010**	DNA repair protein RadA
SMU.328	1,3	1,9	-1,2	**0,003**	**0,000**	**0,034**	putative carbonic anhydrase
SMU.329	1,2	1,7	-1,1	**0,013**	**0,000**	0,260	hypothetical protein
SMU.330	-1,0	-1,0	-1,1	0,943	0,713	0,302	glutamyl-tRNA synthetase

SMU.331	1,2	1,0	-1,1	0,226	0,957	0,395	putative transcriptional regulator
SMU.332	1,1	1,0	1,0	0,437	0,643	0,780	hypothetical protein
SMU.333	-1,1	-1,3	1,2	0,597	**0,041**	0,164	hypothetical protein
SMU.334	1,1	-1,3	-1,2	0,143	**0,000**	**0,001**	argininosuccinate synthase
SMU.335	1,0	-1,3	-1,3	0,708	**0,000**	**0,000**	argininosuccinate lyase
SMU.336	1,1	-1,1	-1,2	0,348	0,052	**0,016**	ribonuclease P
SMU.337	1,1	-1,1	1,0	0,307	0,113	0,618	hypothetical protein
SMU.338	1,1	-1,2	1,2	0,290	0,082	0,115	Jag family RNA-binding protein
SMU.339	-1,2	-1,4	1,4	0,448	0,107	0,124	hypothetical protein
SMU.340	1,2	1,1	1,3	0,313	0,476	0,109	50S ribosomal protein L34
SMU.341	1,2	1,1	1,0	**0,040**	0,305	0,955	putative deoxyribonuclease
SMU.342	1,2	-1,0	-1,1	0,051	0,874	0,267	hypothetical protein
SMU.343	1,1	1,1	-1,1	0,504	0,168	0,493	hypothetical protein
SMU.344	1,1	1,3	-1,0	0,391	0,075	0,874	hypothetical protein
SMU.345c	-1,1	1,0	-1,1	0,228	0,735	0,426	hypothetical protein
SMU.346	1,2	1,2	-1,2	0,111	0,070	0,132	putative NADH dehydrogenase; NAD(P)H nitroreductase
SMU.348	1,2	-1,1	-1,3	0,051	0,285	**0,002**	putative histidine triad (HIT) hydrolase
SMU.349	1,3	1,1	-1,0	**0,000**	0,093	0,438	dimethyladenosine transferase
SMU.350	1,0	1,1	1,0	0,777	0,594	0,963	hypothetical protein
SMU.351	-1,0	1,3	-1,1	0,948	**0,037**	0,448	ribosome-associated GTPase
SMU.352	1,2	**6,0**	1,0	0,155	**0,000**	0,945	ribulose-phosphate 3-epimerase
SMU.353	1,1	**5,6**	1,1	0,361	**0,000**	0,433	hypothetical protein
SMU.354	1,1	**5,4**	1,5	0,171	**0,000**	**0,000**	hypothetical protein
SMU.355	1,2	**5,9**	1,2	0,082	**0,000**	**0,040**	putative CMP-binding factor
SMU.356	1,0	**3,2**	-1,0	0,910	**0,000**	0,823	pur operon repressor
SMU.357	1,6	1,1	1,5	**0,001**	0,390	**0,007**	30S ribosomal protein S12
SMU.358	1,7	1,2	1,3	**0,000**	**0,001**	**0,000**	30S ribosomal protein S7
SMU.359	1,4	1,1	1,2	**0,000**	0,182	**0,000**	elongation factor G
SMU.360	1,4	1,1	1,5	**0,000**	0,135	**0,000**	glyceraldehyde-3-phosphate dehydrogenase
SMU.361	-1,0	-1,0	-1,0	0,564	0,854	0,889	phosphoglycerate kinase
SMU.362	-1,2	-1,1	-1,0	0,248	0,567	0,993	hypothetical protein
SMU.363	1,2	-1,1	-1,2	0,141	0,450	0,128	transcriptional regulator; glutamine synthetase repressor
SMU.364	1,2	-1,2	-1,0	0,073	0,081	0,858	glutamine synthetase type 1; glutamate--ammonia ligase
SMU.365	1,1	-1,3	-1,2	0,056	**0,000**	**0,000**	glutamate synthase (large subunit)
SMU.366	1,1	-1,3	-1,2	0,157	**0,000**	**0,005**	glutamate synthase subunit beta
SMU.367	1,2	-1,1	-1,2	**0,045**	0,210	0,094	hypothetical protein
SMU.368c	1,2	1,1	1,2	**0,001**	0,381	**0,001**	hypothetical protein
SMU.369c	-1,1	-1,2	1,5	0,426	0,165	**0,041**	hypothetical protein
SMU.370	-1,1	1,0	-1,0	0,095	0,814	0,449	putative ABC transporter, ATP-binding protein
SMU.371	1,0	-1,0	1,0	0,736	0,627	0,563	hypothetical protein
SMU.372	-1,1	-1,0	1,2	0,234	0,592	**0,036**	hypothetical protein
SMU.373	1,1	1,1	-1,1	0,427	0,276	0,409	hypothetical protein
SMU.374	1,0	-1,0	1,1	0,852	0,763	0,350	putative oxidoreductase
SMU.375	-1,0	1,1	1,1	0,611	0,459	0,228	hypothetical protein
SMU.376	-1,0	1,0	1,1	0,687	0,563	0,302	putative aminotransferase
SMU.378	-1,1	-1,2	-1,3	0,645	0,670	0,427	hypothetical protein
SMU.379	1,4	1,3	-1,5	**0,030**	0,057	**0,008**	hypothetical protein
SMU.381c	1,2	1,0	-1,2	**0,048**	0,705	0,076	hypothetical protein
SMU.382c	-1,1	-1,0	1,1	0,193	0,378	0,238	putative oxidoreductase
SMU.383c	-1,1	-1,0	1,1	0,106	0,641	0,193	putative reductase
SMU.384	-1,0	-1,1	-1,4	0,781	0,207	**0,000**	hypothetical protein
SMU.385	1,1	1,1	-1,3	0,320	0,604	**0,025**	putative glycoprotein endopeptidase
SMU.386	1,0	1,0	-1,3	0,866	0,801	0,181	putative ribosomal-protein-alanine acetyltransferase
SMU.387	1,2	1,0	-1,1	0,128	0,936	0,338	putative DNA-binding/iron metalloprotein/AP endonuclease
SMU.388	1,0	-1,1	1,0	0,941	0,483	0,976	putative integral membrane protein; branched-chain amino acid permease
SMU.389	-1,0	1,1	1,3	0,949	0,815	0,475	hypothetical protein
SMU.390	1,1	-1,1	-1,5	0,661	0,658	0,101	hypothetical protein
SMU.391c	1,2	1,0	1,0	**0,034**	0,952	0,548	hypothetical protein
SMU.392c	1,1	-1,0	-1,0	0,174	0,896	0,557	hypothetical protein
SMU.393	1,1	-1,1	-1,0	0,290	0,331	0,869	hypothetical protein
SMU.394c	1,2	1,0	1,1	0,322	0,931	0,745	hypothetical protein
SMU.395	-1,0	-1,1	1,1	0,478	0,160	0,246	x-prolyl-dipeptidyl aminopeptidase
SMU.396	1,2	-1,2	-1,0	**0,046**	0,137	0,854	putative glycerol uptake facilitator protein
SMU.399	-1,1	1,6	-1,3	0,403	**0,000**	**0,011**	hypothetical protein

SMU.400	-1,0	**2,0**	-1,1	0,863	**0,000**	0,516	putative secreted esterase
SMU.401c	-1,0	-1,2	-1,2	0,687	**0,041**	0,091	hypothetical protein
SMU.402	-1,1	1,1	1,4	0,213	0,118	**0,000**	pyruvate formate-lyase
SMU.403	1,1	1,1	-1,0	0,085	0,108	0,455	DNA polymerase IV
SMU.404c	-1,4	1,2	-1,2	**0,010**	**0,039**	0,124	hypothetical protein
SMU.405c	-1,1	-1,2	1,2	0,213	0,131	**0,018**	putative transcriptional regulator
SMU.406c	1,0	-1,1	1,1	0,589	0,373	0,318	hypothetical protein
SMU.407	-1,1	1,0	-1,1	0,727	0,877	0,692	hypothetical protein
SMU.408	1,1	-1,1	-1,1	0,255	0,069	0,312	putative permease
SMU.409	1,4	1,2	-1,1	0,082	0,283	0,554	hypothetical protein
SMU.410	1,2	-1,0	-1,0	0,058	0,671	0,608	putative transcriptional regulator
SMU.411c	-1,4	1,1	1,1	**0,002**	0,254	0,328	hypothetical protein
SMU.412c	-1,2	-1,0	1,2	**0,032**	0,942	0,089	putative Hit-like protein involved in cell-cycle regulation
SMU.413	1,2	1,0	-1,2	**0,026**	0,567	**0,044**	putative ABC transporter, ATP-binding protein
SMU.414	1,0	-1,1	-1,1	0,969	0,564	0,510	putative ABC transporter, permease protein
SMU.415	1,1	-1,1	-1,1	0,427	0,568	0,183	hypothetical protein
SMU.416	1,2	-1,1	-1,1	0,201	0,499	0,384	tRNA (guanine-N(7)-)-methyltransferase
SMU.417	1,1	-1,1	1,1	0,254	0,513	0,447	hypothetical protein
SMU.418	1,4	1,1	1,1	**0,000**	0,162	**0,017**	transcription elongation factor NusA
SMU.419	1,2	-1,3	-1,2	0,512	0,343	0,556	hypothetical protein
SMU.420	1,2	-1,1	1,0	0,340	0,410	0,992	hypothetical protein
SMU.421	1,4	-1,0	1,5	**0,000**	0,682	**0,000**	translation initiation factor IF-2
SMU.422	1,3	-1,2	1,2	0,094	0,188	0,282	ribosome-binding factor A
SMU.423	**38,8**	**49,5**	**54,3**	**0,000**	**0,000**	**0,000**	hypothetical protein
SMU.424	**3,1**	**5,9**	**3,1**	**0,000**	**0,000**	**0,000**	negative transcriptional regulator, CopY
SMU.426	**2,9**	**5,5**	**3,6**	**0,000**	**0,000**	**0,000**	copper-transporting ATPase; P-type ATPase
SMU.427	**2,7**	**4,0**	**2,3**	**0,000**	**0,000**	**0,000**	putative copper chaperone
SMU.428	1,1	1,1	-1,2	0,161	0,346	**0,038**	hypothetical protein
SMU.429c	-1,2	-1,3	1,2	**0,041**	**0,002**	**0,005**	hypothetical protein
SMU.431	-1,1	-1,0	1,0	0,192	0,918	0,871	putative ABC transporter, ATP-binding protein
SMU.432	1,0	1,0	1,0	0,868	0,864	0,777	putative ABC transporter, integral membrane protein
SMU.433	1,1	-1,2	-1,3	0,505	0,281	0,075	putative transcriptional regulator
SMU.434	-1,2	-1,3	-1,3	0,157	**0,033**	0,061	hypothetical protein
SMU.435	-1,1	1,0	-1,4	0,247	0,651	**0,000**	putative N-acetylglucosamine-6-phosphate deacetylase
SMU.436c	1,0	-1,1	1,1	0,995	0,086	0,342	putative transposase, ISSmu1
SMU.438c	-1,1	-1,0	-1,3	**0,000**	0,859	**0,000**	putative (R)-2-hydroxyglutaryl-CoA dehydratase activator-related protein
SMU.439	-1,2	1,2	1,0	0,190	0,156	0,978	putative transcriptional regulator
SMU.440	1,1	1,2	-1,3	0,417	0,197	**0,022**	hypothetical protein
SMU.441	1,1	1,1	-1,2	0,521	0,113	**0,014**	putative transcriptional regulator
SMU.442	1,2	1,1	-1,1	0,198	0,510	0,557	hypothetical protein
SMU.444	-1,1	-1,3	-1,5	0,929	0,621	0,294	hypothetical protein
SMU.445	1,1	-1,2	-1,1	0,146	0,073	0,608	glycyl-tRNA synthetase subunit alpha
SMU.446	1,1	-1,3	1,1	0,174	**0,000**	0,149	glycyl-tRNA synthetase subunit beta
SMU.447	1,2	-1,2	1,4	0,106	0,108	**0,030**	hypothetical protein
SMU.448	1,1	-1,1	1,5	0,160	0,441	**0,000**	hypothetical protein
SMU.449	1,2	-1,3	-1,2	0,234	0,070	0,301	gamma-glutamyl kinase
SMU.450	1,1	-1,1	-1,2	**0,011**	**0,014**	**0,000**	gamma-glutamyl phosphate reductase
SMU.451	1,5	-1,1	-1,1	0,317	0,778	0,859	hypothetical protein
SMU.453	1,2	-1,0	1,1	**0,048**	0,792	0,288	S-adenosyl-methyltransferase MraW
SMU.454	1,0	-1,1	1,1	0,746	0,572	0,584	putative cell division protein
SMU.455	1,1	-1,0	1,1	**0,014**	0,408	0,253	putative penicillin-binding protein 2X
SMU.456	1,1	-1,1	1,0	0,330	0,380	0,808	phospho-N-acetylmuramoyl-pentapeptide-transferase
SMU.457	-1,2	-1,3	-1,2	0,613	0,463	0,672	hypothetical protein
SMU.458	1,1	-1,1	-1,0	0,196	0,083	0,996	putative ATP-dependent RNA helicase
SMU.459	1,1	1,0	1,0	0,398	0,918	0,680	putative ABC transporter, amino acid binding protein
SMU.460	1,0	1,0	-1,0	0,832	0,979	0,847	putative amino acid ABC transporter, permease
SMU.461	-1,0	-1,1	-1,1	0,692	0,727	0,715	putative amino acid ABC transporter, ATP-binding protein
SMU.462	-1,2	-1,5	-1,3	0,620	0,181	0,493	hypothetical protein
SMU.463	1,2	-1,1	-1,1	**0,001**	0,389	0,210	putative thioredoxin reductase (NADPH)
SMU.464	1,1	-1,1	-1,1	0,208	0,130	0,170	nicotinate phosphoribosyltransferase
SMU.465	1,2	-1,2	-1,1	**0,045**	0,090	0,243	NAD synthetase
SMU.466	1,2	-1,1	-1,1	0,119	0,158	0,414	cysteine aminopeptidase C
SMU.467	1,2	1,0	1,2	**0,004**	0,780	**0,009**	penicillin-binding protein 1a; membrane carboxypeptidase
SMU.469	1,2	1,0	1,3	**0,048**	0,843	**0,022**	Holliday junction-specific endonuclease

SMU.470	1,1	1,4	1,4	0,656	0,058	**0,022**	hypothetical protein
SMU.471	1,0	-1,2	1,1	0,939	0,354	0,705	hypothetical protein
SMU.472	1,1	-1,1	1,0	0,480	0,228	0,828	N6-adenine-specific DNA methylase
SMU.473	1,3	-1,0	1,1	**0,000**	0,984	**0,042**	hypothetical protein
SMU.474	-1,1	-1,2	-1,2	0,218	0,066	0,183	S-ribosylhomocysteinase
SMU.475	1,4	1,1	1,1	**0,000**	0,195	0,065	hypothetical protein
SMU.478	1,3	1,0	-1,1	**0,001**	0,926	0,144	guanylate kinase
SMU.479	1,1	-1,2	1,1	0,570	0,450	0,671	DNA-directed RNA polymerase subunit omega
SMU.480	1,1	-1,2	-1,0	0,386	**0,001**	0,985	primosome assembly protein PriA
SMU.481	1,3	-1,1	-1,0	**0,002**	0,336	0,575	methionyl-tRNA formyltransferase
SMU.482	1,2	-1,1	-1,0	0,064	0,235	0,652	putative RNA-binding Sun protein; rRNA methylase
SMU.483	1,2	1,1	-1,1	**0,020**	0,485	0,522	putative phosphoprotein phosphatase (pppL protein)
SMU.484	1,2	1,0	1,1	**0,017**	0,874	0,298	putative serine/threonine protein kinase
SMU.485	-1,1	1,1	-1,2	0,348	0,464	0,218	hypothetical protein
SMU.486	1,1	1,1	-1,0	0,381	0,225	0,871	putative histidine kinase
SMU.487	1,1	-1,1	-1,1	0,499	0,494	0,394	putative response regulator
SMU.488	1,0	-1,1	-1,1	0,981	0,377	0,155	putative hydrolase
SMU.489	1,0	-1,2	-1,1	0,982	0,363	0,443	hypothetical protein
SMU.490	-1,1	1,2	1,3	0,100	0,057	**0,000**	putative pyruvate formate-lyase activating enzyme
SMU.491	-1,1	-1,1	-1,1	0,418	0,501	0,451	putative DeoR-type transcriptional regulator
SMU.493	-1,1	1,2	-1,1	0,131	**0,000**	**0,040**	formate acetyltransferase (pyruvate formate-lyase 2)
SMU.494	-1,0	1,2	-1,2	0,749	**0,017**	**0,005**	fructose-6-phosphate aldolase
SMU.495	-1,1	1,1	-1,1	0,473	0,215	0,469	glycerol dehydrogenase
SMU.496	-1,0	1,0	1,1	0,807	0,993	0,361	putative cysteine synthetase A; O-acetylserine lyase
SMU.497c	-1,1	-1,1	-1,2	0,164	0,385	**0,028**	hypothetical protein
SMU.498	1,6	**64,8**	-1,0	**0,000**	**0,000**	0,912	putative late competence protein
SMU.499	1,3	**40,5**	-1,0	**0,000**	**0,000**	0,518	putative late competence protein
SMU.500	-1,4	**3,2**	1,1	**0,000**	**0,000**	0,537	putative ribosome-associated protein
SMU.501	1,1	-1,5	1,1	0,582	**0,002**	0,342	hypothetical protein
SMU.502	1,2	-1,4	1,5	**0,022**	**0,000**	**0,000**	hypothetical protein
SMU.503c	-1,3	-1,3	-1,2	**0,037**	**0,030**	0,197	hypothetical protein
SMU.504	-1,0	-1,3	1,2	0,590	**0,005**	**0,023**	putative site-specific DNA-methyltransferase
SMU.505	1,1	**14,4**	1,4	0,324	**0,000**	**0,004**	putative adenine-specific DNA methylase
SMU.506	1,0	**5,6**	1,2	0,873	**0,000**	0,139	putative type II restriction endonuclease
SMU.507	-1,2	**4,9**	-1,3	**0,017**	**0,000**	**0,002**	DeoR family transcriptional regulator
SMU.508	-1,0	**4,9**	-1,2	0,718	**0,000**	**0,047**	hypothetical protein
SMU.509	1,1	1,0	-1,2	0,278	0,893	0,160	hypothetical protein
SMU.510c	-1,2	-1,5	-1,0	0,119	**0,000**	0,891	hypothetical protein
SMU.512c	-1,0	-1,0	-1,0	0,949	0,956	0,877	hypothetical protein
SMU.513	1,3	-1,3	-1,1	0,296	0,243	0,782	hypothetical protein
SMU.514	1,1	-1,2	-1,1	0,329	**0,012**	0,282	putative transcriptional regulator
SMU.515	1,0	-1,0	-1,2	0,812	0,936	**0,000**	myosin-cross-reactive antigen
SMU.516	1,3	-1,1	-1,2	0,101	0,559	0,117	hypothetical protein
SMU.517	1,1	-1,2	-1,0	0,454	0,265	0,783	phosphopantetheine adenylyltransferase
SMU.518	1,2	-1,1	-1,2	0,101	0,165	**0,028**	hypothetical protein
SMU.520	1,1	-1,0	-1,3	0,285	0,866	**0,005**	hypothetical protein
SMU.521	1,1	-1,2	1,0	0,622	0,336	0,899	hypothetical protein
SMU.522	1,1	-1,1	1,0	0,334	0,366	0,684	ribosomal RNA large subunit methyltransferase N
SMU.523	-1,1	-1,3	1,2	0,411	0,069	0,193	hypothetical protein
SMU.524	-1,0	-1,1	-1,1	0,980	0,127	0,099	putative ABC transporter, ATP-binding protein
SMU.525	1,0	-1,1	-1,1	0,687	**0,009**	**0,014**	putative ABC transporter, ATP-binding protein
SMU.526c	1,1	-1,0	1,0	0,433	0,942	0,595	putative transcriptional regulator
SMU.527	1,2	-1,1	-1,2	**0,018**	0,173	**0,046**	hypothetical protein
SMU.528c	1,1	-1,0	-1,5	0,121	0,963	**0,000**	hypothetical protein
SMU.529	1,0	1,1	-1,0	0,950	0,837	0,920	hypothetical protein
SMU.530c	-1,1	1,3	1,1	0,581	**0,012**	0,308	hypothetical protein
SMU.531	-1,2	-1,4	-1,1	0,115	**0,000**	0,342	putative chorismate mutase
SMU.532	1,1	-1,4	-1,2	0,166	**0,000**	**0,001**	anthranilate synthase component I
SMU.533	1,1	-1,4	-1,1	0,503	**0,006**	0,304	anthranilate synthase component II
SMU.534	1,0	-1,5	-1,3	0,618	**0,000**	**0,005**	anthranilate phosphoribosyltransferase
SMU.535	1,2	-1,5	-1,3	**0,040**	**0,000**	**0,002**	indole-3-glycerol-phosphate synthase
SMU.536	-1,0	-1,7	-1,4	0,769	**0,000**	**0,010**	N-(5'-phosphoribosyl)anthranilate isomerase
SMU.537	1,3	-1,4	-1,2	**0,001**	**0,000**	**0,012**	tryptophan synthase subunit beta
SMU.538	1,2	-1,7	-1,1	0,137	**0,000**	0,282	tryptophan synthase subunit alpha
SMU.539c	1,0	**8,7**	1,2	0,826	**0,000**	0,050	signal peptidase type IV
SMU.540	1,3	1,0	1,1	**0,025**	0,884	0,308	peroxide resistance protein Dpr

SMU.541	1,1	1,1	-1,2	0,637	0,652	0,454	hypothetical protein
SMU.542	1,2	1,0	-1,1	0,103	0,884	0,530	putative glucose kinase
SMU.543	-1,2	-1,3	1,0	**0,001**	**0,000**	0,593	hypothetical protein
SMU.545	1,3	-1,4	-1,4	0,647	0,622	0,636	hypothetical protein
SMU.546	1,2	-1,1	1,1	**0,000**	0,071	**0,048**	putative GTP-binding protein
SMU.547	-1,0	-1,3	1,2	0,881	0,283	0,532	hypothetical protein
SMU.548	1,2	-1,1	1,1	**0,029**	0,372	0,356	UDP-N-acetylmuramoyl-L-alanyl-D-glutamate synthetase
SMU.549	1,1	-1,2	1,5	0,331	0,143	**0,016**	undecaprenyldiphospho-muramoylpentapeptide beta-N-acetylglucosaminyltransferase
SMU.550	1,0	-1,3	1,4	0,717	**0,006**	**0,000**	putative cell division protein FtsQ (DivIB)
SMU.551	1,3	-1,0	-1,1	**0,000**	0,500	0,310	cell division protein FtsA
SMU.552	1,4	-1,0	1,2	**0,001**	0,698	0,060	cell division protein FtsZ
SMU.553	1,2	-1,1	1,1	**0,016**	0,200	0,429	hypothetical protein
SMU.554	1,3	-1,1	1,2	**0,005**	0,455	**0,037**	hypothetical protein
SMU.555	-1,1	-1,1	1,3	0,809	0,793	0,503	hypothetical protein
SMU.556	1,0	-1,3	1,3	0,813	0,118	0,100	hypothetical protein
SMU.557	1,3	-1,1	1,2	**0,003**	0,419	0,050	putative cell division protein DivIVA
SMU.558	1,1	-1,3	-1,0	0,151	**0,000**	0,497	isoleucyl-tRNA synthetase
SMU.560c	-1,1	-1,2	1,9	0,481	0,350	**0,000**	hypothetical protein
SMU.561c	1,1	-1,0	1,2	0,168	0,544	0,114	MutT family hydrolase
SMU.562	1,2	-1,1	1,2	**0,001**	0,176	**0,011**	ATP-dependent protease ClpE
SMU.563	1,0	-1,2	-1,3	0,594	**0,023**	**0,001**	ornithine carbamoyltransferase
SMU.564	1,1	1,0	-1,1	0,637	0,996	0,606	hypothetical protein
SMU.565c	-1,0	-1,2	1,1	0,576	0,076	0,480	putative transposase, ISSmu1
SMU.566c	1,0	-1,1	-1,1	0,863	0,480	0,535	hypothetical protein
SMU.567	1,1	-1,2	-1,2	0,741	0,174	0,246	putative glutamine ABC transporter, permease component
SMU.568	1,1	-1,2	-1,4	0,398	0,186	**0,006**	putative amino acid ABC transporter, ATP-binding protein
SMU.569	1,0	1,2	-1,3	0,729	0,069	**0,033**	putative ferrous ion transport protein A
SMU.570	1,0	1,2	-1,1	0,797	**0,011**	0,125	putative ferrous ion transport protein B
SMU.571	1,1	1,3	1,0	0,190	**0,004**	0,909	hypothetical protein
SMU.572	1,3	1,1	-1,0	**0,000**	0,180	0,500	bifunctional 5,10-methylene-tetrahydrofolate dehydrogenase/ 5,10-methylene-tetrahydrofolate cyclohydrolase
SMU.573	1,2	-1,1	-1,1	**0,028**	0,533	0,320	hypothetical protein
SMU.574c	-1,5	-1,0	-1,5	**0,000**	0,930	**0,000**	hypothetical protein
SMU.575c	-2,0	-1,0	-2,1	**0,000**	0,654	**0,000**	hypothetical protein
SMU.576	-1,1	1,1	-1,0	0,111	0,457	0,803	putative response regulator LytR
SMU.577	-1,1	-1,0	-1,1	0,066	0,461	0,083	putative histidine kinase LytS
SMU.580	1,2	1,1	-1,2	**0,034**	0,125	**0,032**	exodeoxyribonuclease VII large subunit
SMU.581	-1,0	-1,1	-1,2	0,797	0,534	0,139	exodeoxyribonuclease VII small subunit
SMU.582	1,2	-1,1	-1,0	**0,037**	0,152	0,635	putative farnesyl diphosphate synthase
SMU.583	1,3	-1,1	-1,1	**0,021**	0,394	0,183	putative hemolysin
SMU.584	1,1	-1,0	-1,1	0,339	0,945	0,283	putative arginine repressor
SMU.585	1,1	1,3	-1,2	0,087	**0,000**	**0,007**	DNA repair protein RecN
SMU.586	1,0	1,0	1,1	0,975	0,572	0,536	hypothetical protein
SMU.587	1,1	-1,1	1,2	0,302	0,581	0,090	hypothetical protein
SMU.588	-1,0	-1,0	1,3	0,810	0,613	**0,002**	hypothetical protein
SMU.589	1,4	1,0	1,3	**0,028**	0,794	0,129	putative DNA-binding protein
SMU.590c	-1,1	-1,2	-1,0	0,586	0,097	0,925	putative transposase, fragment
SMU.591c	-1,0	-1,2	-1,0	0,423	**0,020**	0,985	hypothetical protein
SMU.592c	1,0	1,4	1,1	0,562	**0,000**	0,058	putative transcriptional regulator
SMU.593	1,1	1,0	-1,1	0,221	0,843	0,409	putative ferric uptake regulator protein FurR
SMU.594	-1,2	1,5	-1,3	0,432	0,161	0,230	hypothetical protein
SMU.595	-1,1	-1,2	-1,2	0,275	**0,013**	**0,012**	dihydroorotate dehydrogenase 1A
SMU.596	1,1	-1,2	1,1	0,315	0,154	0,354	phosphoglyceromutase
SMU.597	1,1	-1,0	1,1	0,349	0,869	0,315	penicillin-binding protein 2b
SMU.598	1,2	1,0	-1,1	**0,020**	0,474	0,364	recombination protein RecR
SMU.599	1,0	-1,1	-1,4	0,662	0,271	**0,000**	D-alanyl-alanine synthetase A
SMU.600c	1,2	1,1	1,0	0,098	0,562	0,974	hypothetical protein
SMU.602	1,0	-1,0	-1,5	0,676	0,656	**0,000**	putative sodium-dependent transporter
SMU.603	1,0	-1,1	-1,0	0,447	0,152	0,530	putative D-Ala-D-Ala adding enzyme; UDP-N-acetylmuramoylalanyl-D-glutamyl-2,6- diaminopimelate-D-alanyl-D-alanyl ligase pentapeptide synthase
SMU.604	-1,2	-1,2	1,0	0,338	0,220	0,787	hypothetical protein
SMU.605	1,1	-1,2	1,5	0,545	0,209	**0,001**	hypothetical protein

SMU.606	1,1	-1,2	1,2	0,481	0,054	0,082	hypothetical protein
SMU.607	-1,0	-1,1	1,1	0,691	0,354	0,221	hypothetical protein
SMU.608	1,1	-1,1	-1,1	0,241	0,158	0,174	peptide chain release factor 3
SMU.609	-1,1	-1,2	-1,2	0,243	**0,042**	**0,004**	putative 40K cell wall protein precursor
SMU.610	1,3	-1,2	1,5	**0,000**	**0,000**	**0,000**	cell surface antigen SpaP
SMU.611	1,4	1,3	1,1	**0,001**	**0,009**	0,246	ATP-dependent RNA helicase
SMU.613	1,4	1,3	-1,2	**0,025**	0,076	0,331	hypothetical protein
SMU.614	1,4	1,0	-1,1	0,389	0,929	0,881	hypothetical protein
SMU.616	1,3	1,3	-1,1	0,190	0,198	0,602	hypothetical protein
SMU.618	1,0	-1,2	1,0	0,941	0,531	0,953	hypothetical protein
SMU.620	1,1	1,1	-1,1	0,686	0,707	0,775	hypothetical protein
SMU.621c	-1,0	-1,0	-1,0	0,717	0,991	0,887	hypothetical protein
SMU.622c	-1,1	-1,2	1,1	0,262	0,106	0,643	hypothetical protein
SMU.623c	-1,1	-1,0	1,1	0,479	0,587	0,256	putative deacetylase
SMU.624	-1,0	-1,1	-1,2	0,958	0,171	**0,045**	putative 1-acylglycerol-3-phosphate O-acyltransferase
SMU.625	1,8	**131,7**	-1,2	**0,000**	**0,000**	**0,000**	putative competence protein
SMU.626	1,5	**61,7**	1,1	**0,000**	**0,000**	0,229	putative competence protein
SMU.627	-1,1	**3,3**	1,0	0,561	**0,000**	0,882	hypothetical protein
SMU.628	1,0	1,3	-1,2	0,943	**0,006**	**0,023**	DNA polymerase III subunit delta
SMU.629	1,3	-1,4	1,1	**0,003**	**0,000**	0,415	putative manganese-type superoxide dismutase, Fe/Mn-SOD
SMU.630	1,1	-1,2	-1,1	0,544	**0,038**	0,249	hypothetical protein
SMU.631	-1,0	-1,2	-1,2	0,827	0,157	0,089	hypothetical protein
SMU.632	-1,1	1,0	-1,3	0,692	0,772	0,160	putative transcriptional regulator
SMU.633	-1,1	-1,2	-1,3	0,083	**0,004**	**0,000**	putative thioesterase
SMU.634	1,0	-1,0	-1,0	0,747	0,706	0,974	S-adenosylmethionine:tRNA ribosyltransferase-isomerase
SMU.635	1,1	1,5	-1,1	0,246	**0,000**	0,415	hypothetical protein
SMU.636	-1,0	1,3	-1,2	0,767	**0,004**	0,059	putative N-acetylglucosamine-6-phosphate isomerase
SMU.637c	-1,0	1,1	-1,1	0,531	0,399	0,440	hypothetical protein
SMU.638	1,1	1,1	-1,1	0,184	0,370	0,344	putative 16S pseudouridylate synthase
SMU.639	1,0	1,1	-1,1	0,664	0,371	0,332	putative acetyltransferase
SMU.640c	-1,1	-1,0	-1,0	0,095	0,226	0,448	GntR family transcriptional regulator
SMU.641	1,2	1,2	-1,0	0,069	0,077	0,672	putative oxidoreductase
SMU.642	1,1	-1,1	-1,0	0,784	0,480	0,905	hypothetical protein
SMU.643	-1,0	-1,0	1,2	0,739	0,784	**0,013**	putative esterase
SMU.644	1,3	**39,7**	1,0	**0,000**	**0,000**	0,873	putative competence protein/transcription factor
SMU.645	1,1	**9,8**	-1,2	0,168	**0,000**	**0,001**	putative oligopeptidase
SMU.646	1,2	**10,3**	-1,2	0,083	**0,000**	0,178	putative phosphatase
SMU.647	1,0	1,8	1,0	0,923	**0,000**	0,704	putative methyltransferase
SMU.648	1,1	1,0	1,3	0,277	0,808	**0,034**	foldase protein PrsA
SMU.649	-1,1	-1,2	1,0	0,245	0,109	0,966	hypothetical protein
SMU.650	1,2	-1,0	-1,0	**0,000**	0,314	0,568	alanyl-tRNA synthetase
SMU.651c	1,1	1,4	-1,0	0,318	**0,000**	0,767	putative ABC transporter, substrate-binding protein
SMU.652c	-1,1	1,3	-1,1	0,196	**0,000**	**0,046**	nitrate ABC transporter ATP-binding protein
SMU.653c	-1,1	1,3	-1,0	0,100	**0,000**	0,677	putative ABC transporter, permease protein
SMU.654	1,0	-1,0	1,0	0,606	0,963	0,623	putative ABC transporter, ATP-binding protein MutF
SMU.655	-1,1	-1,0	1,2	0,389	0,684	**0,017**	putative MutE
SMU.656	-1,0	1,0	-1,1	0,523	0,581	0,252	putative MutE
SMU.657	1,1	1,0	-1,1	0,386	0,956	0,119	putative MutG
SMU.658	-1,1	1,0	-1,1	0,075	0,935	0,198	hypothetical protein
SMU.659	1,1	1,1	-1,2	0,464	0,475	**0,026**	putative response regulator SpaR
SMU.660	-1,1	-1,0	-1,1	0,333	0,572	0,146	putative histidine kinase SpaK
SMU.661	-1,0	-1,1	-1,2	0,764	0,186	**0,017**	putative transcriptional regulator
SMU.662	-1,2	-1,1	-1,0	0,128	0,487	0,736	hypothetical protein
SMU.663	1,0	1,0	-1,1	0,796	0,775	0,235	N-acetyl-gamma-glutamyl-phosphate reductase
SMU.664	-1,0	1,1	-1,1	0,754	0,061	0,098	bifunctional ornithine acetyltransferase/N-acetylglutamate synthase protein
SMU.665	-1,0	1,2	-1,2	0,605	**0,032**	**0,004**	acetylglutamate kinase
SMU.666	1,0	1,1	-1,1	0,758	0,341	**0,015**	acetylornithine aminotransferase
SMU.667	1,1	-1,1	1,1	0,077	0,065	0,091	ribonucleotide-diphosphate reductase subunit beta
SMU.668c	1,1	-1,3	1,2	0,262	**0,001**	0,052	ribonucleotide-diphosphate reductase subunit alpha
SMU.669c	1,2	-1,3	1,1	0,411	0,211	0,499	putative glutaredoxin
SMU.670	1,2	-1,2	-1,2	**0,030**	**0,011**	**0,027**	aconitate hydratase
SMU.671	1,1	-1,1	-1,0	0,308	0,155	0,668	citrate synthase
SMU.672	1,1	-1,1	-1,1	0,060	0,100	0,086	isocitrate dehydrogenase
SMU.673	1,1	-1,0	-1,3	0,506	0,846	0,102	hypothetical protein

SMU.674	1,2	1,2	1,1	0,282	0,345	0,796	phosphocarrier protein HPr
SMU.675	1,3	1,1	1,3	**0,000**	**0,016**	**0,000**	phosphoenolpyruvate:sugar phosphotransferase system enzyme I, PTS system EI component
SMU.676	1,3	1,0	1,1	**0,000**	0,552	0,059	NADP-dependent glyceraldehyde-3-phosphate dehydrogenase
SMU.677	1,1	-1,1	-1,4	0,481	0,335	**0,021**	MerR family transcriptional regulator
SMU.678	-1,0	-1,0	-1,2	0,751	0,637	**0,007**	oxidoreductase
SMU.679	1,0	1,0	-1,2	0,662	0,762	0,106	oxidoreductase
SMU.680	1,1	-1,0	-1,0	0,374	0,962	0,898	putative gamma-carboxymuconolactone decarboxylase subunit
SMU.681	-1,3	1,1	-1,2	0,117	0,484	0,353	hypothetical protein
SMU.682	1,1	-1,3	1,2	0,334	**0,000**	**0,000**	hypothetical protein
SMU.683	-1,0	-1,2	1,2	0,579	**0,000**	**0,000**	putative ATP-binding protein
SMU.684	1,1	-1,2	1,4	0,193	0,093	**0,000**	hypothetical protein
SMU.685	1,1	-1,4	1,5	0,299	**0,017**	**0,009**	hypothetical protein
SMU.687c	-1,2	1,1	-1,1	0,198	0,755	0,429	hypothetical protein
SMU.688	-1,0	-1,1	1,0	0,818	0,315	0,836	hypothetical protein
SMU.689	1,2	-1,1	1,0	**0,001**	0,263	0,378	hypothetical protein
SMU.690	1,0	-1,4	1,2	0,805	0,050	0,245	hypothetical protein
SMU.691	1,2	-1,2	-1,2	0,096	0,099	0,083	peptidase T
SMU.692	1,0	-1,1	1,1	0,970	0,629	0,677	hypothetical protein
SMU.694c	-1,4	1,3	-1,0	**0,010**	0,050	0,735	putative ferredoxin [4Fe-4S]
SMU.695	1,1	1,1	1,2	0,487	0,502	0,252	hypothetical protein
SMU.696	1,1	-1,0	-1,0	0,457	0,988	0,833	cytidylate kinase
SMU.697	1,1	-1,2	1,0	0,200	0,131	0,976	translation initiation factor IF-3
SMU.698	-1,0	-1,2	1,7	0,967	**0,004**	**0,000**	50S ribosomal protein L35
SMU.699	1,2	-1,0	1,3	**0,019**	0,986	**0,002**	50S ribosomal protein L20
SMU.700c	-1,0	-1,4	1,3	0,795	**0,007**	**0,016**	putative phosphoglycerate mutase-like protein
SMU.701c	-1,2	-1,2	1,2	0,226	0,242	0,224	hypothetical protein
SMU.702c	1,1	-1,1	-1,0	0,601	0,295	0,922	putative transcriptional regulator
SMU.703c	-1,1	-1,2	-1,0	0,338	0,053	0,858	hypothetical protein
SMU.704c	1,0	-1,1	1,3	0,743	0,533	**0,005**	putative autolysin; amidase
SMU.706c	1,1	-1,2	1,3	0,753	0,411	0,220	hypothetical protein
SMU.707c	1,1	-1,1	1,1	0,350	0,242	0,435	putative endolysin
SMU.709	-1,1	-1,1	-1,0	0,588	0,541	0,822	hypothetical protein
SMU.711	-1,1	-1,0	-1,5	0,602	0,836	**0,046**	hypothetical protein
SMU.712	-1,0	-1,3	1,0	0,609	**0,000**	0,540	phosphoenolpyruvate carboxylase
SMU.713	1,0	-1,1	-1,1	0,667	0,266	0,538	putative cell division protein FtsW
SMU.714	1,5	1,2	1,6	**0,000**	**0,007**	**0,000**	elongation factor Tu
SMU.715	1,3	1,1	-1,0	**0,003**	0,365	0,942	triosephosphate isomerase
SMU.716	-1,1	-1,2	1,2	0,220	**0,025**	**0,006**	putative peptidoglycan branched peptide synthesis protein; alanine adding enzyme; beta-lactam resistance factor MurN
SMU.717	-1,0	-1,2	1,3	0,719	**0,003**	**0,000**	putative peptidoglycan branched peptide synthesis protein MurM
SMU.718c	1,1	-1,1	1,2	0,142	0,119	**0,013**	hypothetical protein
SMU.719c	-1,1	1,0	1,2	0,292	0,975	0,087	hypothetical protein
SMU.720	-1,0	-1,3	-1,1	0,900	**0,020**	0,492	Na+/solute symporter
SMU.721	-1,0	-1,4	1,1	0,904	**0,024**	0,591	hypothetical protein
SMU.722	-1,1	-1,2	1,2	0,828	0,657	0,642	hypothetical protein
SMU.723	1,3	1,0	1,0	**0,000**	0,683	0,628	putative calcium-transporting ATPase; P-type ATPase
SMU.724	-1,1	-1,1	-1,1	0,608	0,360	0,656	putative glycerophosphoryl diester phosphodiesterase
SMU.725c	1,1	-1,0	1,0	0,414	0,954	0,877	hypothetical protein
SMU.727	-1,1	-1,0	-1,5	0,515	0,984	**0,013**	putative transcriptional regulator
SMU.728	-1,0	1,1	-1,1	0,705	0,535	0,386	putative oxidoreductase
SMU.730	1,2	1,1	1,0	0,420	0,707	0,777	hypothetical protein
SMU.731	1,1	1,0	-1,0	0,147	0,723	0,934	putative ABC transporter, ATP-binding protein
SMU.732	-1,2	-1,3	1,0	0,110	**0,009**	0,747	hypothetical protein
SMU.734	-1,1	-1,2	-1,2	0,202	0,136	0,059	hypothetical protein
SMU.735	-1,2	-1,0	-1,3	0,147	0,942	0,067	hypothetical protein
SMU.737	-1,1	1,1	-1,3	0,323	0,302	**0,008**	hypothetical protein
SMU.738	1,2	-1,1	-1,6	0,102	0,578	**0,008**	hypothetical protein
SMU.739c	-1,1	-1,0	-1,1	0,122	0,361	**0,037**	hypothetical protein
SMU.741	1,0	1,0	1,1	0,635	0,943	0,307	hypothetical protein
SMU.742	1,1	-1,1	1,1	0,538	0,380	0,398	hypothetical protein
SMU.743	1,2	-1,0	1,1	0,247	0,778	0,268	hypothetical protein

SMU.744	1,1	-1,0	1,0	0,075	0,680	0,518	putative cell division protein FtsY; signal recognition particle (docking protein)
SMU.745	-1,1	-1,2	1,2	0,463	0,054	0,072	putative drug-export protein; multidrug resistance protein
SMU.746c	1,0	1,1	1,1	0,690	0,536	0,365	hypothetical protein
SMU.747c	-1,1	-1,1	1,1	0,579	0,523	0,575	putative permease
SMU.748	1,0	-1,1	1,1	0,866	0,592	0,336	hypothetical protein
SMU.750c	1,3	-1,4	-1,4	**0,011**	0,132	**0,047**	hypothetical protein
SMU.751	1,1	-1,0	-1,1	0,125	0,695	0,423	transcriptional accessory protein
SMU.752	1,1	-1,2	-1,1	0,383	0,160	0,362	hypothetical protein
SMU.753	1,3	1,9	-1,0	0,286	**0,005**	0,964	hypothetical protein
SMU.754	1,6	1,2	1,1	**0,000**	0,102	0,640	HPr kinase/phosphorylase
SMU.755	1,3	1,1	1,1	0,091	0,462	0,617	prolipoprotein diacylglyceryl transferase
SMU.756	1,1	-1,1	-1,0	0,637	0,712	0,784	hypothetical protein
SMU.757	1,1	-1,1	1,0	0,613	0,611	0,786	hypothetical protein
SMU.758c	-1,1	1,0	1,0	0,561	0,998	0,927	hypothetical protein
SMU.759	1,1	1,1	1,0	0,213	0,313	0,752	putative protease
SMU.761	1,3	1,2	-1,1	**0,000**	**0,009**	**0,021**	putative protease
SMU.764	1,3	1,2	-1,0	**0,000**	0,055	0,504	alkyl hydroperoxide reductase
SMU.765	1,3	-1,0	-1,0	**0,005**	0,985	0,605	NADH oxidase/alkyl hydroperoxidase reductase peroxide-forming
SMU.766	1,0	-1,1	-1,1	0,947	0,448	0,596	hypothetical protein
SMU.767	-1,0	-1,2	1,1	0,904	0,076	0,201	putative transposase, ISSmu1
SMU.768c	-1,2	-1,8	1,4	0,153	**0,000**	0,099	hypothetical protein
SMU.769	1,2	**18,2**	-1,3	0,181	**0,000**	0,087	hypothetical protein
SMU.770c	-1,4	-1,3	-1,0	**0,000**	**0,002**	0,771	putative manganese transporter
SMU.771c	-1,0	-1,3	1,3	0,851	0,068	0,162	hypothetical protein
SMU.772	1,1	**4,9**	-1,2	0,350	**0,000**	**0,024**	putative glucan-binding protein D; BglB-like protein
SMU.773c	1,2	-1,2	1,1	**0,002**	**0,012**	0,122	lysyl-tRNA synthetase
SMU.774	1,0	-1,0	-1,3	0,877	0,770	**0,002**	hypothetical protein
SMU.775c	-1,0	-1,1	1,3	0,585	0,306	**0,000**	integral membrane protein
SMU.776	1,1	1,0	-1,4	0,556	0,960	**0,005**	hypothetical protein
SMU.777	1,1	1,1	-1,1	0,358	0,353	0,664	3-dehydroquinate dehydratase
SMU.778	1,1	1,0	1,0	0,358	0,748	0,663	shikimate 5-dehydrogenase
SMU.779	1,3	1,2	-1,1	**0,018**	0,129	0,539	3-dehydroquinate synthase
SMU.780	1,4	1,2	-1,1	**0,000**	**0,042**	0,178	chorismate synthase
SMU.781	1,2	1,1	1,0	0,057	0,438	0,740	prephenate dehydrogenase
SMU.782	1,1	1,0	1,1	0,586	0,881	0,566	hypothetical protein
SMU.784	1,1	-1,1	-1,2	0,383	0,079	**0,005**	3-phosphoshikimate 1-carboxyvinyltransferase
SMU.785	-1,0	-1,2	-1,3	0,778	0,055	**0,002**	putative shikimate kinase
SMU.786	-1,1	-1,3	-1,1	0,176	**0,003**	0,355	prephenate dehydratase
SMU.787	1,1	-1,1	-1,1	0,228	0,222	0,289	putative transcriptional regulator
SMU.788	1,1	-1,0	-1,2	**0,045**	0,475	**0,010**	putative RNA methyltransferase
SMU.789	-1,0	-1,1	-1,1	0,904	0,462	0,508	hypothetical protein
SMU.790	1,3	-1,1	1,1	**0,019**	0,521	0,692	hypothetical protein
SMU.791c	1,1	-1,1	-1,0	0,166	0,498	0,951	hypothetical protein
SMU.793	-1,0	-1,1	-1,3	0,846	0,423	**0,002**	hypothetical protein
SMU.794	-1,0	-1,1	-1,2	0,685	0,177	0,118	hypothetical protein
SMU.795	-1,2	-1,1	-1,1	0,319	0,455	0,730	esterase
SMU.796	-1,1	-1,0	-1,1	0,346	0,620	0,350	hypothetical protein
SMU.797	1,1	-1,2	-1,2	0,761	0,326	0,377	hypothetical protein
SMU.798c	1,0	1,1	-1,0	0,757	0,449	0,909	hypothetical protein
SMU.799c	-1,0	1,1	1,0	0,871	0,140	0,802	hypothetical protein
SMU.800	-1,2	-1,1	-1,3	0,708	0,787	0,530	hypothetical protein
SMU.801	1,3	1,1	-1,2	**0,002**	0,165	**0,021**	GTPase ObgE
SMU.802	1,1	1,1	-1,5	0,560	0,579	**0,046**	hypothetical protein
SMU.803c	-1,1	1,2	-1,0	0,197	**0,007**	0,950	putative ABC transporter, ATP-binding protein
SMU.804	-1,0	-1,1	1,3	0,875	0,328	**0,003**	hypothetical protein
SMU.805c	1,1	1,0	1,2	0,150	0,860	**0,044**	putative amino acid ABC transporter, ATP-binding protein
SMU.806c	1,1	1,0	1,2	**0,019**	0,833	**0,000**	putative glutamine ABC transporter, permease protein
SMU.807	1,1	1,2	-1,2	0,367	0,056	**0,027**	hypothetical protein
SMU.809	1,2	-1,0	-1,1	**0,010**	0,826	0,052	excinuclease ABC subunit B
SMU.811	1,1	-1,2	1,1	0,648	**0,039**	0,288	hypothetical protein
SMU.812	-1,3	-1,1	-1,1	0,215	0,489	0,720	hypothetical protein
SMU.813	1,1	-1,1	-1,2	0,354	0,488	0,058	transcription regulator
SMU.814	1,3	-1,0	-1,0	0,098	0,742	0,825	putative MutT-like protein

SMU.815	1,0	-1,3	-1,1	0,779	**0,026**	0,450	putative amino acid transporter, amino acid-binding protein
SMU.816	1,1	-1,0	-1,1	0,160	0,848	0,419	transaminase
SMU.817	1,0	-1,3	-1,2	0,915	0,089	0,318	putative amino acid transporter, amino acid-binding protein
SMU.818	1,1	-1,1	1,3	0,672	0,746	0,195	30S ribosomal protein S21
SMU.819	1,1	1,0	1,4	0,358	0,803	**0,045**	putative large conductance mechanosensitive channel
SMU.820	1,1	1,1	1,3	0,092	0,407	**0,008**	hypothetical protein
SMU.821	1,1	1,1	1,1	0,093	0,110	0,139	DNA primase
SMU.822	1,2	1,1	1,1	**0,004**	0,296	0,158	RNA polymerase sigma factor RpoD
SMU.823	1,0	1,0	1,2	0,963	0,982	**0,045**	hypothetical protein
SMU.824	1,1	-1,0	1,0	0,148	0,842	0,566	dTDP-4-keto-L-rhamnose reductase
SMU.825	1,1	1,1	-1,0	0,450	0,368	0,844	putative RgpAc; glycosyltransferase
SMU.826	1,0	1,1	-1,1	0,806	0,602	0,632	rhamnosyltransferase
SMU.827	1,0	-1,1	1,1	0,982	0,732	0,596	putative polysaccharide ABC transporter, permease protein
SMU.828	1,3	1,1	-1,1	**0,001**	0,237	0,071	putative polysaccharide ABC transporter, ATP-binding protein
SMU.829	1,0	-1,1	1,2	0,541	0,213	0,077	putative glycosyltransferase
SMU.830	1,2	-1,0	1,1	0,090	0,615	0,110	RgpFc protein
SMU.831	-1,0	-1,2	1,3	0,815	0,100	**0,005**	hypothetical protein
SMU.832	1,0	-1,2	1,4	0,835	0,153	**0,044**	hypothetical protein
SMU.833	1,3	1,1	-1,0	**0,029**	0,636	0,949	putative glycosyltransferase
SMU.834	-1,0	-1,0	-1,0	0,818	0,726	0,834	hypothetical protein
SMU.835	-1,1	1,5	1,2	0,671	**0,000**	0,182	hypothetical protein
SMU.836	1,5	**74,8**	-1,2	**0,000**	**0,000**	**0,005**	hypothetical protein
SMU.837	1,3	**32,0**	-1,2	**0,013**	**0,000**	0,112	putative reductase
SMU.838	1,3	**2,4**	-1,1	**0,000**	**0,000**	0,079	glutathione reductase
SMU.839	-1,1	-1,1	-1,1	**0,009**	**0,006**	0,059	putative folyl-polyglutamate synthetase
SMU.840c	-1,2	1,1	1,9	0,303	0,684	**0,001**	hypothetical protein
SMU.841	1,2	1,0	-1,2	**0,020**	0,605	**0,025**	putative aminotransferase
SMU.842	1,2	-1,0	-1,3	0,101	0,825	**0,013**	thiamine biosynthesis protein ThiI
SMU.843	1,2	1,1	-1,1	**0,043**	0,316	0,166	hypothetical protein
SMU.844	1,2	1,0	-1,0	0,204	0,847	0,827	hypothetical protein
SMU.845	1,1	-1,1	1,2	0,585	0,330	0,099	hypothetical protein
SMU.847c	-1,0	-1,1	1,2	0,248	0,311	**0,000**	hypothetical protein
SMU.846	1,3	-1,0	1,2	**0,039**	0,795	**0,047**	50S ribosomal protein L21
SMU.848	1,3	-1,1	1,1	0,388	0,680	0,785	hypothetical protein
SMU.849	1,2	-1,1	1,5	0,363	0,541	0,054	50S ribosomal protein L27
SMU.850	1,1	-1,0	-1,3	0,530	0,658	**0,011**	hypothetical protein
SMU.851	1,1	-1,1	-1,3	0,418	0,509	**0,039**	hypothetical protein
SMU.852	1,0	1,2	-1,0	0,819	0,109	0,853	putative transcriptional regulator; CpsY-like protein
SMU.853	-1,1	-1,1	1,0	0,519	0,412	0,796	lipoprotein signal peptidase
SMU.854	1,2	1,0	-1,2	**0,013**	0,598	**0,033**	putative pseudouridylate synthase
SMU.855	-1,0	-1,0	-1,1	0,883	0,782	0,686	hypothetical protein
SMU.856	1,0	1,2	-1,1	0,972	**0,002**	0,149	bifunctional pyrimidine regulatory protein PyrR uracil phosphoribosyltransferase
SMU.857	-1,1	1,1	-1,0	0,495	0,220	0,550	putative uracil permease
SMU.858	-1,1	1,1	-1,2	0,082	0,365	**0,007**	aspartate carbamoyltransferase catalytic subunit
SMU.859	-1,0	1,2	-1,2	0,953	**0,009**	**0,021**	carbamoyl phosphate synthase small subunit
SMU.860	-1,0	1,2	-1,1	0,869	**0,001**	**0,006**	carbamoyl phosphate synthase large subunit
SMU.862	1,3	1,1	1,0	**0,001**	0,363	0,562	putative permease
SMU.863	1,2	1,1	1,2	0,153	0,365	0,232	putative ABC transporter, ATP-binding protein
SMU.864	-1,0	1,2	-1,0	0,647	**0,028**	0,767	putative ABC transporter, permease component
SMU.865	-1,0	-1,1	1,2	0,919	0,500	0,316	30S ribosomal protein S16
SMU.866	1,0	-1,1	1,3	0,983	0,751	0,439	hypothetical protein
SMU.867	-1,1	-1,1	-1,2	0,436	0,373	**0,030**	16S rRNA-processing protein RimM
SMU.868	1,2	1,0	-1,4	0,057	0,817	**0,000**	tRNA (guanine-N(1)-)-methyltransferase
SMU.869	1,0	-1,2	-1,3	0,813	0,217	0,057	putative thioredoxin reductase
SMU.870	-1,3	1,2	-1,0	**0,000**	**0,004**	0,624	putative transcriptional regulator of sugar metabolism
SMU.871	-1,3	-1,0	-1,1	0,059	0,741	0,332	putative fructose-1-phosphate kinase
SMU.872	-1,1	-1,0	1,1	0,107	0,847	**0,029**	putative PTS system, fructose-specific enzyme IIABC component
SMU.873	-1,0	1,1	-1,0	0,295	**0,010**	0,274	5-methyltetrahydropteroyltriglutamate--homocysteine S-methyltransferase
SMU.874	-1,0	1,2	-1,3	0,908	**0,001**	**0,000**	bifunctional homocysteine S-methyltransferase/5,10-

							methylenetetrahydrofolate reductase protein
SMU.875c	1,0	1,0	1,1	0,962	0,599	0,080	putative transposase, IS150-like
SMU.876	-1,1	-1,1	1,0	**0,000**	**0,036**	0,665	putative MSM operon regulatory protein
SMU.877	-1,3	1,4	1,2	**0,000**	**0,000**	**0,015**	alpha-galactosidase
SMU.878	-1,3	1,4	1,2	**0,000**	**0,000**	**0,009**	multiple sugar-binding ABC transporter, sugar-binding protein precursor MsmE
SMU.879	-1,2	1,4	1,2	0,165	**0,003**	0,114	multiple sugar-binding ABC transporter, permease protein MsmF
SMU.880	-1,2	1,3	1,4	0,115	**0,013**	**0,001**	multiple sugar-binding ABC transporter, permease protein MsmG
SMU.881	-1,0	1,6	1,3	0,806	**0,000**	**0,000**	sucrose phosphorylase, GtfA
SMU.882	1,0	1,6	1,1	0,978	**0,000**	0,206	multiple sugar-binding ABC transporter, ATP-binding protein, MsmK
SMU.883	-1,1	1,7	1,2	0,082	**0,000**	**0,009**	dextran glucosidase DexB
SMU.885	-1,0	1,1	-1,1	0,635	0,073	0,067	galactose operon repressor GalR
SMU.886	-1,0	1,4	-1,1	0,493	**0,000**	**0,017**	galactokinase
SMU.887	-1,0	1,5	-1,0	0,817	**0,000**	0,843	galactose-1-phosphate uridylyltransferase
SMU.888	1,2	1,1	1,0	0,058	0,188	0,916	UDP-galactose 4-epimerase, GalE
SMU.889	1,0	1,1	-1,0	0,708	0,154	0,671	putative penicillin-binding protein, class C; fmt-like protein
SMU.890	-1,0	-1,3	-1,1	0,850	0,061	0,401	hypothetical protein
SMU.891	1,0	-1,2	1,4	0,546	**0,019**	**0,000**	type I restriction-modification system DNA methylase
SMU.892	1,2	-1,2	1,4	**0,018**	0,076	**0,000**	putative type I restriction-modification system, specificity determinant; restriction endonuclease
SMU.893	1,1	-1,2	1,3	0,153	**0,024**	**0,003**	putative anticodon nuclease
SMU.895	-1,3	-1,1	1,5	**0,038**	0,154	**0,002**	DNA-damage-inducible protein
SMU.896	1,0	-1,3	1,1	0,891	0,238	0,671	hypothetical protein
SMU.897	-1,2	-1,2	1,3	**0,006**	**0,000**	**0,000**	putative type I restriction-modification system, helicase subunits
SMU.898	-1,0	-1,2	-1,2	0,779	**0,019**	**0,048**	hypothetical protein
SMU.899	1,1	-1,2	-1,1	0,315	0,093	0,351	hypothetical protein
SMU.900	1,2	1,0	-1,2	**0,036**	0,923	**0,038**	dihydrodipicolinate reductase
SMU.901	1,2	1,0	-1,2	**0,014**	0,694	**0,020**	tRNA CCA-pyrophosphorylase
SMU.902	1,1	1,1	-1,1	**0,023**	0,234	0,089	putative ABC transporter, ATP-binding protein
SMU.905	1,1	1,2	-1,1	0,227	**0,007**	0,106	putative ABC transporter, ATP-binding protein
SMU.906	-1,0	1,1	-1,1	0,898	0,232	0,238	putative ABC transporter, ATP-binding protein
SMU.909	-1,1	-1,0	1,0	0,168	0,517	0,490	putative permease
SMU.910	1,1	-1,3	1,0	**0,004**	**0,000**	0,702	glucosyltransferase-S
SMU.911c	-1,2	1,2	-1,1	0,082	0,212	0,301	hypothetical protein
SMU.913	1,2	-1,1	1,0	**0,001**	0,183	0,478	glutamate dehydrogenase
SMU.914c	-1,2	1,1	-1,2	0,099	0,362	0,303	hypothetical protein
SMU.915c	-1,3	-1,1	1,2	**0,043**	0,533	0,239	7-cyano-7-deazaguanine reductase
SMU.916c	-1,1	1,1	1,0	0,070	0,095	0,665	hypothetical protein
SMU.917c	-1,1	1,2	1,0	**0,011**	**0,001**	0,723	putative 6-pyruvoyl tetrahydropterin synthase
SMU.919c	-1,1	1,1	-1,1	0,171	0,073	0,100	putative ATPase, confers aluminum resistance
SMU.921	-1,2	-1,2	-1,3	0,317	0,481	0,272	putative transcriptional regulator
SMU.922	1,1	1,1	-1,0	**0,039**	0,079	0,622	putative ABC transporter, ATP-binding protein
SMU.923	1,1	1,2	-1,0	0,209	**0,024**	0,841	putative ABC transporter, ATP-binding protein
SMU.924	1,2	-1,3	-1,1	0,163	0,147	0,613	thiol peroxidase
SMU.925	**3,8**	**10,3**	**6,4**	**0,000**	**0,000**	**0,000**	hypothetical protein
SMU.926	1,9	**3,7**	**2,2**	**0,000**	**0,000**	**0,000**	GTP-pyrophosphokinase
SMU.927	1,7	**3,4**	1,6	**0,000**	**0,000**	**0,001**	putative response regulator
SMU.928	1,5	**2,7**	1,7	**0,000**	**0,000**	**0,000**	putative histidine kinase
SMU.929c	-1,0	-1,2	-1,3	0,806	0,261	**0,046**	hypothetical protein
SMU.930c	-1,0	-1,1	-1,2	0,640	0,145	**0,045**	putative transcriptional regulator
SMU.932	-1,0	-1,2	-1,4	0,688	0,204	**0,001**	hypothetical protein
SMU.933	1,1	-1,0	-1,3	0,182	0,803	**0,031**	putative amino acid ABC transporter, periplasmic amino acid-binding protein
SMU.934	-1,0	1,0	-1,2	0,597	0,908	0,087	putative amino acid ABC transporter, permease protein
SMU.935	1,2	-1,1	1,1	0,052	0,430	0,333	putative amino acid ABC transporter, permease protein
SMU.936	-1,0	-1,0	-1,3	0,707	0,795	**0,024**	putative amino acid ABC transporter, ATP-binding protein
SMU.937	1,0	-1,2	-1,2	0,892	**0,007**	**0,000**	putative mevalonate diphosphate decarboxylase
SMU.938	1,0	-1,1	-1,2	0,832	0,427	0,052	putative phosphomevalonate kinase
SMU.939	-1,0	1,0	-1,3	0,993	0,583	**0,000**	isopentenyl pyrophosphate isomerase
SMU.940c	-1,1	1,0	1,3	0,698	0,808	0,144	putative hemolysin III
SMU.941c	-1,0	1,2	**2,0**	0,724	0,109	**0,000**	hypothetical protein

SMU.942	1,1	1,0	1,1	0,254	0,899	0,364	putative hydroxymethylglutaryl-CoA reductase
SMU.943c	1,1	1,1	-1,0	0,268	0,364	0,993	putative hydroxymethylglutaryl-CoA synthase
SMU.944	1,1	-1,1	1,1	0,545	0,151	0,223	thymidylate synthase
SMU.946	1,2	1,1	-1,1	0,156	0,467	0,512	putative permease
SMU.947	1,5	1,1	1,1	0,103	0,583	0,588	putative dihydrofolate reductase
SMU.948	1,3	1,2	-1,0	0,245	0,323	0,879	hypothetical protein
SMU.949	1,3	1,0	-1,1	**0,012**	0,748	0,644	ATP-dependent protease ATP-binding subunit ClpX
SMU.950	1,4	1,0	-1,0	**0,025**	0,973	0,723	ribosome biogenesis GTP-binding protein YsxC
SMU.951	1,1	1,0	-1,2	0,503	0,624	0,098	putative amino acid permease
SMU.952	1,1	1,0	-1,3	0,109	0,453	**0,000**	homocysteine methyltransferase
SMU.953c	-1,2	1,1	-1,0	**0,032**	0,256	0,971	putative transcriptional regulator/aminotransferase
SMU.954	-1,0	-1,3	-1,2	0,652	**0,002**	**0,034**	pyridoxamine kinase
SMU.955	-1,1	-1,2	-1,2	0,439	**0,049**	0,152	hypothetical protein
SMU.956	1,2	1,1	1,0	**0,006**	0,058	0,932	putative Clp-like ATP-dependent protease, ATP-binding subunit
SMU.957	1,2	-1,2	1,0	**0,016**	**0,010**	0,805	50S ribosomal protein L10
SMU.958	1,3	-1,0	1,0	**0,011**	0,777	0,769	hypothetical protein
SMU.959c	-1,0	-1,0	1,1	0,998	0,846	0,591	hypothetical protein
SMU.960	1,4	-1,0	1,1	0,056	0,862	0,483	50S ribosomal protein L7/L12
SMU.961	1,1	1,1	-1,1	0,409	0,623	0,458	hypothetical protein
SMU.962	1,0	-1,1	-1,2	0,932	0,423	0,055	putative dehydrogenase
SMU.963c	-1,0	-1,1	1,2	0,751	0,230	0,213	putative deacetylase
SMU.965	1,2	-1,1	-1,2	**0,019**	0,237	**0,045**	homoserine dehydrogenase
SMU.966	1,2	-1,1	-1,1	0,146	0,360	0,371	homoserine kinase
SMU.967	1,1	-1,1	-1,2	0,573	0,326	0,051	putative folyl-polyglutamate synthetase
SMU.968	1,3	-1,0	-1,1	**0,000**	0,836	0,219	GTP cyclohydrolase I
SMU.969	1,3	1,0	-1,1	**0,010**	0,939	0,383	dihydropteroate synthase
SMU.970	1,2	1,0	-1,3	0,283	0,759	0,084	putative dihydroneopterin aldolase
SMU.971	1,2	1,1	-1,0	**0,024**	0,538	0,576	putative 2-amino-4-hydroxy-6-hydroxymethylpteridine pyrophosphokinase
SMU.972	1,1	-1,1	-1,1	0,400	0,605	0,391	UDP-N-acetylenolpyruvoylglucosamine reductase
SMU.973	1,2	1,0	-1,1	0,078	0,673	0,320	putative spermidine/putrescine ABC transporter, ATP-binding protein
SMU.974	-1,0	1,0	-1,0	0,979	0,864	0,749	putative spermidine/putrescine ABC transporter, permease protein
SMU.975	-1,1	-1,1	-1,1	0,391	0,232	0,554	putative spermidine/putrescine ABC transporter, permease protein
SMU.976	-1,0	1,0	-1,1	0,956	0,826	0,530	putative ABC transporter, periplasmic spermidine/putrescine-binding protein
SMU.977	-1,0	1,0	-1,1	0,727	0,694	0,410	putative transcriptional antiterminator LicT (fragment)
SMU.980	-1,1	1,2	-1,1	**0,039**	**0,006**	0,445	putative PTS system, beta-glucoside-specific EII component
SMU.981	1,0	1,2	-1,1	0,615	**0,015**	0,420	putative BglB fragment
SMU.982	-1,1	1,1	-1,1	0,323	0,617	0,247	putative BglB fragment
SMU.983	-1,1	-1,2	1,3	0,333	**0,001**	**0,000**	putative transcriptional regulator
SMU.984	-1,1	-1,2	-1,5	0,515	0,129	**0,002**	hypothetical protein
SMU.985	1,0	-1,1	-1,2	0,990	0,285	**0,005**	putative beta-glucosidase
SMU.986c	1,1	-1,2	1,1	0,361	0,066	0,583	hypothetical protein
SMU.987	-1,0	-1,3	1,3	0,751	**0,000**	**0,001**	cell wall-associated protein precursor WapA
SMU.988	-1,1	-1,3	1,0	0,558	**0,002**	0,809	putative cardiolipin synthase
SMU.989	1,1	1,0	-1,1	0,177	0,904	0,308	aspartate-semialdehyde dehydrogenase
SMU.990	1,3	1,1	-1,1	**0,006**	0,352	0,270	dihydrodipicolinate synthase
SMU.991	1,2	-1,1	-1,1	0,160	0,674	0,703	putative ribonucleotide reductase
SMU.992	1,0	-1,2	-1,3	0,793	0,185	**0,033**	hypothetical protein
SMU.993	1,1	-1,1	-1,4	0,477	0,134	**0,001**	ribosomal biogenesis GTPase
SMU.994	1,2	-1,1	-1,3	0,151	0,267	**0,015**	ribonuclease HII
SMU.995	-1,1	-1,2	1,0	0,622	0,117	0,954	ferrichrome ABC transporter permease protein
SMU.996	-1,2	-1,3	1,2	0,307	0,071	0,151	ferrichrome ABC transporter permease protein
SMU.997	1,0	-1,1	1,4	0,799	0,553	**0,004**	putative inorganic ion ABC transporter, ATP-binding protein; ferrichrome transport system
SMU.998	-1,1	-1,2	1,3	0,609	0,083	0,113	putative ABC transporter, periplasmic ferrichrome-binding protein
SMU.999	-1,4	-1,3	1,0	0,086	0,229	0,994	hypothetical protein
SMU.1000	-1,3	-1,2	1,1	0,221	0,372	0,430	hypothetical protein
SMU.1001	1,6	**45,0**	-1,1	**0,000**	**0,000**	0,175	putative DNA processing Smf protein
SMU.1002	1,2	**6,5**	1,2	**0,015**	**0,000**	**0,005**	DNA topoisomerase I

SMU.1003	1,2	**5,8**	1,1	**0,034**	**0,000**	0,321	tRNA (uracil-5-)-methyltransferase Gid
SMU.1004	1,3	1,2	1,5	**0,000**	**0,004**	**0,000**	glucosyltransferase-I
SMU.1005	1,3	-1,1	1,2	**0,000**	**0,015**	**0,000**	glucosyltransferase-Si
SMU.1006	1,0	-1,3	-1,1	0,641	**0,018**	0,261	putative ABC transporter, ATP-binding protein
SMU.1007	-1,0	-1,4	1,0	0,911	**0,000**	0,558	putative ABC transporter, permease protein
SMU.1008	1,1	-1,1	-1,0	0,540	0,269	0,737	putative response regulator
SMU.1009	-1,0	-1,3	1,1	0,852	0,059	0,637	putative histidine kinase
SMU.1010	-1,1	-1,3	-1,1	0,130	**0,000**	0,293	putative citrate lyase ligase
SMU.1011	1,0	1,0	1,0	0,482	0,639	0,463	putative CitG protein
SMU.1012c	1,1	-1,1	-1,2	0,358	0,389	**0,008**	putative transcriptional regulator
SMU.1013c	-1,1	-1,1	-1,1	**0,024**	0,296	0,096	putative Mg2+/citrate transporter
SMU.1014	-1,1	-1,3	-1,6	0,378	**0,019**	**0,000**	hypothetical protein
SMU.1016	1,1	-1,2	-1,3	0,670	0,189	**0,042**	acetyl-CoA carboxylase biotin carboxyl carrier protein subunit
SMU.1017	-1,0	-1,2	-1,2	0,935	**0,020**	**0,027**	putative oxaloacetate decarboxylase, sodium ion pump subunit
SMU.1018	1,4	-1,2	1,2	0,339	0,416	0,666	hypothetical protein
SMU.1019	1,1	-1,1	-1,2	0,512	0,316	0,107	citrate lyase subunit gamma
SMU.1020	-1,1	-1,2	-1,2	0,482	**0,025**	0,078	putative citrate lyase CilB, citryl-CoA lyase, beta subunit
SMU.1021	-1,1	-1,1	-1,2	0,276	0,063	**0,003**	putative citrate lyase, alfa subunit
SMU.1022	-1,1	-1,2	-1,1	0,128	0,121	0,143	2'-(5''-triphosphoribosyl)-3'-dephospho-CoA:apo-citrate lyase
SMU.1023	1,0	-1,0	-1,2	0,459	0,440	**0,017**	oxaloacetate decarboxylase
SMU.1024c	1,0	-1,2	-1,0	0,663	0,225	0,785	putative putative transposase
SMU.1025	-1,0	-1,0	-1,1	0,618	0,775	0,443	putative transcriptional regulator
SMU.1026	-1,1	-1,1	1,1	0,599	0,386	0,216	hypothetical protein
SMU.1027	-1,0	-1,4	-1,0	0,943	**0,007**	0,969	putative transcription regulator
SMU.1028	-1,1	-1,2	1,3	0,253	**0,017**	**0,000**	putative hydrolase or acyltransferase
SMU.1029	1,0	-1,1	-1,2	0,647	0,436	**0,006**	hypothetical protein
SMU.1030	1,2	-1,1	-1,2	**0,000**	**0,017**	**0,002**	putative polyribonucleotide nucleotidyltransferase; Tn916 ORF8-like
SMU.1031	1,1	1,1	-1,1	0,080	0,055	0,060	putative transposon excisionase; Tn916 ORF1-like
SMU.1032	1,0	-1,1	-1,0	0,764	0,143	0,802	putative transposon integrase; Tn916 ORF3-like
SMU.1034c	-1,2	-1,1	1,0	**0,021**	0,300	0,935	site-specific tyrosine recombinase XerS
SMU.1035	1,0	-1,0	-1,1	0,444	0,783	**0,009**	putative ABC transporter, ATP-binding protein
SMU.1036	-1,0	1,0	1,2	0,633	0,490	**0,002**	hypothetical protein
SMU.1037c	1,1	-1,1	1,1	0,438	0,415	0,439	putative histidine kinase
SMU.1038c	1,1	-1,1	1,3	0,278	0,382	**0,006**	putative response regulator
SMU.1039c	-1,0	-1,2	1,2	0,727	0,115	**0,045**	putative lipopolysaccharide glycosyltransferase
SMU.1040c	-1,0	-1,1	-1,1	0,951	0,379	0,130	putative oxidoreductase, short-chain dehydrogenase/reductase
SMU.1041	-1,2	-1,3	-1,2	0,150	**0,006**	0,127	putative ABC transporter, ATP-binding protein
SMU.1042	-1,2	-1,3	1,1	0,051	**0,004**	0,181	hypothetical protein
SMU.1043c	1,2	1,5	1,0	0,057	**0,000**	0,765	phosphotransacetylase
SMU.1044c	1,2	1,4	1,2	0,206	**0,004**	0,278	putative pseudouridylate synthase
SMU.1045c	1,1	1,2	1,5	0,596	0,198	**0,010**	inorganic polyphosphate/ATP-NAD kinase
SMU.1046c	1,4	1,5	1,8	**0,000**	**0,000**	**0,000**	putative GTP pyrophosphokinase
SMU.1047c	-1,2	1,1	-2,3	0,748	0,942	0,227	hypothetical protein
SMU.1048	1,0	-1,3	1,2	0,874	**0,022**	**0,038**	hypothetical protein
SMU.1050	1,2	-1,2	1,1	**0,025**	0,073	0,198	ribose-phosphate pyrophosphokinase
SMU.1051	-1,1	-1,5	1,1	0,140	**0,000**	0,070	iron-sulfur cofactor synthesis protein
SMU.1052	-1,3	-1,7	1,3	0,115	**0,001**	0,146	hypothetical protein
SMU.1053	1,0	-1,8	1,0	0,874	**0,000**	0,977	redox-sensing transcriptional repressor Rex
SMU.1054	-1,0	-1,6	1,1	0,711	**0,000**	0,504	putative glutamine amidotransferase
SMU.1055	1,0	**23,0**	-1,0	0,838	**0,000**	0,799	DNA repair protein RadC
SMU.1056	-1,7	-1,1	-1,8	**0,004**	0,392	**0,000**	hypothetical protein
SMU.1057	1,1	-1,3	1,3	0,730	0,126	0,093	hypothetical protein
SMU.1058	1,0	1,0	1,3	0,795	0,860	0,117	hypothetical protein
SMU.1059	1,1	-2,0	1,1	0,729	**0,010**	0,765	hypothetical protein
SMU.1060	1,2	-1,2	1,2	0,070	0,066	**0,011**	signal recognition particle protein subunit, Ffh
SMU.1061	1,2	-1,2	1,2	0,195	0,053	0,119	putative DNA-binding protein
SMU.1062	1,1	-1,3	1,2	0,064	**0,000**	**0,003**	putative ABC transporter, proline/glycine betaine permease protein
SMU.1063	1,2	-1,3	1,2	**0,048**	**0,005**	**0,017**	putative ABC transporter, ATP-binding protein, proline/glycine betaine transport system
SMU.1064c	-1,1	-1,1	-1,4	0,319	0,624	**0,001**	GntR family transcriptional regulator

SMU.1065c	-1,1	-1,0	-1,3	0,282	0,651	**0,003**	GntR family transcriptional regulator
SMU.1066	1,1	-1,1	-1,1	0,062	0,174	0,321	GMP synthase
SMU.1067c	-1,2	1,1	1,1	0,070	0,272	0,243	putative ABC transporter, permease protein
SMU.1068c	-1,1	1,1	-1,1	0,356	0,214	0,298	putative ABC transporter, ATP-binding protein
SMU.1069c	1,2	1,1	1,2	0,509	0,779	0,364	hypothetical protein
SMU.1070c	-1,0	1,4	-1,2	0,880	**0,031**	0,230	hypothetical protein
SMU.1071c	1,1	1,1	-1,0	0,200	0,175	0,711	hypothetical protein
SMU.1072c	1,1	-1,1	-1,1	0,464	0,061	0,293	putative acetyltransferase
SMU.1073	-1,0	-1,3	-1,1	0,868	**0,000**	0,089	formate--tetrahydrofolate ligase
SMU.1074	-1,1	1,2	1,2	0,204	0,171	0,082	phosphopantothenate--cysteine ligase
SMU.1075	-1,1	-1,0	1,3	0,185	0,935	**0,004**	phosphopantothenoylcysteine decarboxylase
SMU.1076	-1,1	-1,1	1,4	0,553	0,651	0,101	hypothetical protein
SMU.1077	-1,3	1,1	1,1	**0,000**	**0,024**	0,271	putative phosphoglucomutase
SMU.1078c	1,0	-1,0	-1,1	0,777	0,439	0,178	putative ABC transporter, ATP-binding protein
SMU.1079c	1,1	-1,1	-1,0	0,329	0,069	0,704	putative ABC transporter, ATP-binding protein
SMU.1080c	1,3	-1,0	1,2	0,209	0,886	0,247	transposon-like protein
SMU.1081c	1,0	-1,3	1,3	0,773	**0,041**	**0,011**	hypothetical protein
SMU.1082	1,1	-1,1	1,2	0,269	0,146	0,111	serine hydroxymethyltransferase
SMU.1083c	1,2	-1,1	-1,0	0,251	0,426	0,738	hypothetical protein
SMU.1084	1,2	-1,1	1,1	0,056	0,255	0,668	putative protoporphyrinogen oxidase
SMU.1085	1,4	-1,1	1,2	**0,000**	**0,048**	**0,044**	peptide chain release factor 1
SMU.1086	1,2	-1,1	1,1	0,320	0,595	0,395	thymidine kinase
SMU.1087	-1,1	-1,2	-1,3	0,542	0,074	**0,026**	4-oxalocrotonate tautomerase
SMU.1088	-1,2	-1,0	1,0	**0,019**	0,939	0,818	putative thiamine biosynthesis lipoprotein
SMU.1089	-1,3	-1,1	1,5	**0,048**	0,531	**0,017**	hypothetical protein
SMU.1090	-1,0	-1,0	1,4	0,733	0,999	**0,005**	hypothetical protein
SMU.1091	1,0	-1,1	1,2	0,489	0,277	**0,025**	cell wall protein, WapE
SMU.1093	-1,0	-1,1	-1,1	0,766	**0,004**	**0,017**	putative ABC transporter, permease protein
SMU.1094	1,3	1,0	-1,1	**0,000**	0,489	0,149	putative ABC transporter, ATP-binding protein
SMU.1095	-1,1	-1,3	1,3	0,469	**0,005**	**0,004**	putative choline ABC transporter, osmoprotectant binding protein
SMU.1096	-1,1	-1,1	-1,0	0,488	0,214	0,784	putative ABC transporter, ATP-binding protein, choline transporter
SMU.1097c	1,0	-1,1	-1,0	0,988	0,382	0,995	putative transcriptional regulator protein
SMU.1098c	1,1	-1,2	-1,1	0,482	0,124	0,523	putative oxidoreductase
SMU.1100c	-1,1	-1,3	-1,1	0,650	**0,007**	0,524	putative permease
SMU.1102	-1,0	-1,0	-1,1	0,487	0,889	**0,042**	6-phospho-beta-glucosidase
SMU.1104c	1,1	1,1	-1,3	0,061	0,245	**0,003**	phosphoglycerate mutase-like protein
SMU.1105c	1,1	1,0	1,1	0,434	0,973	0,481	putative phosphoglycerate mutase-like protein
SMU.1106c	1,1	1,0	-1,0	0,072	0,987	0,667	phosphoglycerate mutase-like protein
SMU.1107c	1,1	1,0	1,1	0,440	0,825	0,394	hypothetical protein
SMU.1108c	-1,0	1,0	-1,1	0,852	0,981	0,239	hypothetical protein
SMU.1109c	1,1	1,4	1,0	0,448	**0,000**	0,588	putative integral membrane protein; permease
SMU.1111c	1,2	1,6	-1,0	0,259	**0,000**	0,950	hypothetical protein
SMU.1112c	1,3	1,1	-1,1	0,221	0,812	0,589	hypothetical protein
SMU.1113	1,2	1,1	1,0	0,186	0,394	0,692	putative sortase
SMU.1114	1,1	1,1	-1,0	**0,016**	0,326	0,629	DNA gyrase subunit A
SMU.1115	1,2	1,0	1,2	**0,021**	0,822	**0,001**	L-lactate dehydrogenase
SMU.1116c	1,2	-1,0	1,3	0,178	0,996	**0,021**	hypothetical protein
SMU.1117	1,1	-1,0	1,4	0,161	0,914	**0,000**	NADH oxidase (H2O-forming)
SMU.1118c	1,1	-1,1	-1,1	0,681	0,359	0,423	putative ABC sugar transporter, permease protein
SMU.1119c	1,1	-1,1	-1,1	0,112	0,590	0,243	putative sugar ABC transporter, permease protein
SMU.1120	1,1	-1,1	-1,1	0,050	0,427	0,056	putative sugar ABC transporter, ATP-binding protein
SMU.1121c	1,2	-1,1	-1,0	**0,009**	0,070	0,575	putative ABC transporter
SMU.1122	1,0	-1,1	-1,0	0,841	0,432	0,990	cytidine deaminase
SMU.1123	1,1	-1,1	-1,1	0,269	0,313	0,386	deoxyribose-phosphate aldolase
SMU.1124	1,1	-1,0	-1,1	0,445	0,900	0,506	pyrimidine-nucleoside phosphorylase
SMU.1125c	-1,2	-1,0	-1,0	0,253	0,834	0,845	hypothetical protein
SMU.1126	-1,1	-1,4	1,2	**0,033**	**0,000**	**0,013**	pantothenate kinase
SMU.1127	1,0	-1,1	1,3	0,897	0,270	**0,002**	30S ribosomal protein S20
SMU.1128	1,1	-1,2	1,2	**0,039**	**0,003**	**0,005**	putative histidine kinase sensor CiaH
SMU.1129	1,1	-1,2	-1,0	0,519	0,089	0,980	putative response regulator CiaR
SMU.1131c	1,3	-1,0	1,2	**0,019**	0,727	0,126	hypothetical protein
SMU.1132	1,2	1,0	1,1	**0,016**	0,622	0,082	aminopeptidase N, PepN
SMU.1133	1,2	-1,2	1,0	0,242	0,136	0,877	putative phosphate transport system regulatory protein
SMU.1134c	1,2	-1,1	1,1	**0,014**	0,259	0,581	phosphate transporter ATP-binding protein

SMU.1135	1,0	-1,2	1,2	0,525	**0,025**	0,081	phosphate transporter ATP-binding protein
SMU.1136	1,1	-1,1	1,0	0,478	0,203	0,745	putative phosphate ABC transporter, permease protein
SMU.1137	1,2	-1,2	-1,1	0,212	0,238	0,374	putative phosphate ABC transporter, permease protein
SMU.1138	1,2	-1,0	-1,0	0,109	0,791	0,811	putative ABC transporter, phosphate-binding protein
SMU.1139c	-1,1	1,0	-1,1	0,587	0,786	0,226	methylase
SMU.1140c	1,1	-1,0	-1,2	0,449	0,797	0,184	hypothetical protein
SMU.1141c	1,2	1,0	-1,6	0,495	0,959	0,190	hypothetical protein
SMU.1142c	1,2	-1,0	-1,0	0,255	0,905	0,945	transcriptional regulator Spx
SMU.1143c	1,0	1,0	-1,1	0,925	0,909	0,250	bifunctional riboflavin kinase/FMN adenylyltransferase
SMU.1144	1,1	1,1	1,0	0,306	0,417	0,764	tRNA pseudouridine synthase B
SMU.1145c	-1,1	1,4	1,1	0,280	**0,000**	**0,034**	putative histidine kinase; homolog of RumK and ScnK
SMU.1146c	-1,1	1,4	1,0	0,146	**0,000**	0,918	putative response regulator; homolog of RumR and ScnR
SMU.1147c	-1,4	1,1	-1,1	**0,000**	0,279	0,576	hypothetical protein
SMU.1148	-1,1	1,0	-1,3	**0,002**	0,484	**0,000**	putative transporter, ATP-binding protein; bacteriocin immunity protein
SMU.1149	1,0	1,0	1,0	0,685	0,405	0,444	transporter
SMU.1150	-1,1	1,0	1,1	0,159	0,435	0,302	transporter
SMU.1151c	1,1	1,1	-1,1	0,147	0,213	0,294	hypothetical protein
SMU.1152c	-1,1	1,1	1,1	0,335	0,407	0,580	hypothetical protein
SMU.1153c	1,1	1,0	-1,1	0,429	0,702	0,050	hypothetical protein
SMU.1154c	1,2	1,0	-1,0	0,134	0,750	0,590	hypothetical protein
SMU.1155	1,0	1,0	1,0	0,392	0,943	0,933	hypothetical protein
SMU.1156c	1,2	1,0	-1,1	**0,028**	0,673	0,445	hypothetical protein
SMU.1157c	1,0	-1,3	1,2	0,445	**0,000**	**0,000**	hypothetical protein
SMU.1158c	1,1	-1,2	1,2	0,148	**0,009**	**0,019**	hypothetical protein
SMU.1159c	1,1	-1,2	1,2	0,323	**0,003**	**0,011**	hypothetical protein
SMU.1160c	-1,0	-1,2	1,2	0,779	0,053	0,059	hypothetical protein
SMU.1161c	-1,0	-1,1	1,2	0,857	0,396	**0,048**	hypothetical protein
SMU.1163c	1,0	-1,0	1,0	0,508	0,802	0,969	putative ABC transporter, ATP-binding protein
SMU.1164c	1,0	-1,0	-1,1	0,643	0,724	0,066	putative ABC transporter, ATP-binding protein
SMU.1165c	1,0	-1,0	-1,4	0,876	0,919	**0,010**	putative transcription regulator
SMU.1166c	1,0	-1,0	-1,1	0,899	0,929	**0,018**	putative ABC transporter, permease protein
SMU.1167c	1,1	1,1	-1,2	0,168	0,117	**0,002**	putative ABC transporter, ATP-binding protein
SMU.1168	1,1	-1,1	-1,2	**0,029**	**0,038**	**0,002**	putative transcriptional regulator
SMU.1169c	1,1	-1,1	-1,0	0,429	0,378	0,773	putative thioredoxin family protein
SMU.1170	-1,0	-1,2	-1,2	0,825	0,212	0,384	putative cytochrome C biogenesis protein
SMU.1171c	1,1	-1,1	-1,2	0,492	0,640	0,101	hypothetical protein
SMU.1172c	1,0	1,4	-1,1	0,695	**0,006**	0,538	hypothetical protein
SMU.1173	1,0	1,3	1,1	0,583	**0,000**	**0,048**	putative O-acetylhomoserine sulfhydrylase
SMU.1174	1,1	1,2	1,1	**0,027**	**0,000**	0,300	ATP-dependent DNA helicase
SMU.1175	1,1	-1,3	-1,2	0,421	**0,001**	**0,031**	putative sodium/amino acid (alanine) symporter
SMU.1176	-1,2	-1,2	-1,0	**0,005**	**0,001**	0,791	putative cation efflux transporter
SMU.1177c	-1,0	-1,3	1,1	0,824	**0,011**	0,228	putative ABC transporter, glutamine binding protein
SMU.1178c	1,1	-1,2	1,2	0,498	0,233	0,452	putative amino acid ABC transporter, ATP-binding protein
SMU.1179c	1,2	-1,1	1,1	0,321	0,402	0,535	putative amino acid ABC transporter, permease protein
SMU.1180	1,1	-1,1	-1,3	0,524	0,585	0,127	putative alkylphosphonate uptake protein
SMU.1182	-1,0	-1,0	-1,1	0,783	0,917	0,453	mannitol-1-phosphate 5-dehydrogenase
SMU.1183	-1,0	1,1	-1,1	0,939	0,607	0,438	PTS system, mannitol-specific enzyme IIA
SMU.1184c	-1,2	-1,1	-1,0	**0,007**	**0,039**	0,730	putative transcriptional regulator, antiterminator
SMU.1185	-1,7	1,1	-1,2	**0,000**	0,080	**0,019**	PTS system, mannitol-specific enzyme IIBC component
SMU.1187	1,2	-1,0	1,0	**0,004**	0,555	0,874	glucosamine--fructose-6-phosphate aminotransferase
SMU.1188	1,0	-1,3	-1,5	0,871	0,252	0,126	putative signal peptidase
SMU.1189c	1,1	1,1	-1,3	0,521	0,647	0,057	hypothetical protein
SMU.1190	1,3	1,1	1,1	**0,000**	0,249	0,136	pyruvate kinase
SMU.1191	1,5	1,2	1,1	**0,000**	**0,021**	0,068	6-phosphofructokinase
SMU.1192	1,0	1,1	-1,0	0,469	0,136	0,428	DNA polymerase III DnaE
SMU.1193	1,2	1,1	1,3	**0,027**	0,415	**0,036**	putative transcriptional regulator
SMU.1194	-1,1	1,0	1,4	0,586	0,558	**0,000**	putative ABC transporter, ATP-binding protein
SMU.1195	-1,1	-1,1	1,1	0,422	0,384	0,283	permease
SMU.1196c	1,1	-1,1	-1,1	0,328	0,733	0,651	hypothetical protein
SMU.1197	-1,0	1,2	1,2	0,892	0,092	0,165	hypothetical protein
SMU.1200	1,5	-1,1	1,2	**0,000**	0,450	**0,027**	30S ribosomal protein S1
SMU.1201c	1,0	-1,2	-1,2	0,997	0,748	0,752	hypothetical protein
SMU.1203	1,0	1,0	-1,2	0,598	0,840	**0,012**	branched-chain amino acid aminotransferase
SMU.1204	1,1	1,0	1,0	**0,024**	0,771	0,824	DNA topoisomerase IV subunit A
SMU.1205c	-1,1	-1,3	-1,1	0,456	**0,044**	0,325	hypothetical protein

SMU.1206c	1,1	-1,1	1,2	0,539	0,579	0,080	hypothetical protein
SMU.1207	1,4	-1,0	1,2	**0,000**	0,953	**0,001**	mobilization/cell filamentation proteins
SMU.1208c	1,1	-1,0	1,2	0,101	0,783	**0,021**	hypothetical protein
SMU.1209c	1,0	-1,3	1,1	0,695	**0,010**	0,567	hypothetical protein
SMU.1210	1,1	-1,1	1,0	0,085	**0,010**	0,633	DNA topoisomerase IV subunit B
SMU.1211	-1,1	-1,2	1,1	0,659	0,378	0,468	putative glycerol-3-phosphate acyltransferase PlsY
SMU.1213c	1,2	-1,0	-1,0	**0,029**	0,934	0,888	putative 5'-nucleotidase precursor
SMU.1214	1,2	-1,0	-1,1	**0,003**	0,871	0,130	dihydroorotase
SMU.1215	1,1	-1,0	1,0	0,292	0,874	0,594	uracil-DNA glycosylase
SMU.1216c	1,1	1,0	-1,0	0,574	0,921	0,797	putative amino acid ABC transporter, permease protein
SMU.1217c	-1,1	-1,2	-1,1	0,375	0,098	0,302	putative ABC transporter, amino acid binding protein
SMU.1218	1,1	1,1	-1,1	0,291	**0,045**	**0,040**	amidase
SMU.1219c	-1,1	1,1	-1,5	0,308	0,473	**0,000**	hypothetical protein
SMU.1220c	-1,2	-1,0	-1,0	0,056	0,805	0,714	hypothetical protein
SMU.1221	1,1	1,1	-1,2	0,481	0,133	0,063	orotate phosphoribosyltransferase
SMU.1222	1,2	1,2	-1,1	**0,039**	0,085	0,451	orotidine 5'-phosphate decarboxylase
SMU.1223	-1,1	1,1	-1,1	0,373	0,375	0,092	dihydroorotate dehydrogenase 1B
SMU.1224	-1,2	1,1	-1,3	**0,013**	0,321	**0,001**	dihydroorotate dehydrogenase electron transfer subunit
SMU.1225	-1,1	-1,0	-1,0	0,335	0,958	0,837	putative transcriptional regulator
SMU.1226c	1,0	-1,2	-1,1	0,741	0,148	0,516	hypothetical protein
SMU.1227	1,2	1,0	-1,3	**0,013**	0,769	**0,001**	putative purine nucleoside phosphorylase
SMU.1228c	-1,0	-1,1	-1,2	0,757	0,492	**0,043**	glutamine amidotransferase
SMU.1229	1,1	-1,0	-1,3	0,568	0,868	**0,020**	purine nucleoside phosphorylase
SMU.1230c	1,0	-1,2	-1,1	0,810	0,382	0,791	hypothetical protein
SMU.1231c	1,2	1,1	-1,8	0,548	0,796	0,063	hypothetical protein
SMU.1232c	-1,1	-1,1	1,0	0,756	0,745	0,869	hypothetical protein
SMU.1233	1,1	1,0	1,1	**0,035**	0,937	0,412	phosphopentomutase
SMU.1234	1,3	1,1	-1,2	**0,032**	0,494	0,159	ribose-5-phosphate isomerase A
SMU.1235	1,1	1,1	-1,1	**0,049**	0,055	0,147	tRNA modification GTPase TrmE
SMU.1236c	-1,0	-1,2	-1,2	0,912	**0,018**	**0,006**	hypothetical protein
SMU.1237c	-1,2	-1,2	-1,4	0,073	0,062	**0,011**	hypothetical protein
SMU.1238c	1,0	1,1	1,0	0,801	0,660	0,773	hypothetical protein
SMU.1239	1,2	1,1	-1,1	**0,014**	0,195	0,113	dipeptidase PepV
SMU.1240c	1,0	1,1	-1,1	0,984	0,474	0,210	putative nitroreductase
SMU.1241	1,1	1,1	-1,0	**0,009**	**0,049**	0,779	excinuclease ABC subunit C
SMU.1243	-1,2	-1,1	1,0	**0,001**	**0,014**	0,417	putative low temperature requirement A protein
SMU.1245c	1,1	1,0	-1,0	0,268	0,653	0,808	hypothetical protein
SMU.1246c	-1,0	-1,0	1,0	0,925	0,733	0,889	putative transcriptional regulator
SMU.1247	1,3	1,0	1,4	**0,000**	0,558	**0,000**	phosphopyruvate hydratase
SMU.1249c	1,2	-1,3	1,3	0,054	**0,037**	**0,035**	hypothetical protein
SMU.1250c	1,1	-1,3	-1,1	0,471	**0,039**	0,625	hypothetical protein
SMU.1251	-1,2	1,1	-1,1	**0,001**	0,136	0,465	hypothetical protein
SMU.1252	1,1	1,1	-1,4	0,230	0,462	**0,000**	putative glycerate kinase
SMU.1253c	-1,0	1,0	-1,4	0,942	0,829	0,073	hypothetical protein
SMU.1254	1,0	1,1	-1,3	0,707	0,377	**0,000**	hypothetical protein
SMU.1255c	-1,0	-1,2	1,4	0,973	0,655	0,321	hypothetical protein
SMU.1256c	1,2	1,0	1,2	0,367	0,889	0,454	hypothetical protein
SMU.1257c	1,2	1,0	1,0	0,179	0,832	0,810	hypothetical protein
SMU.1258c	1,0	-1,1	-1,1	0,536	0,332	**0,040**	restriction endonuclease
SMU.1259	-1,2	1,1	-1,2	0,231	0,359	0,195	restriction endonuclease
SMU.1260c	1,1	-1,4	-1,2	0,338	**0,002**	0,116	hypothetical protein
SMU.1261c	1,2	-1,4	-1,1	**0,035**	**0,000**	**0,004**	putative phosphoribosyl-ATP pyrophosphohydrolase
SMU.1262c	-1,0	-1,4	1,1	0,895	**0,000**	0,231	hypothetical protein
SMU.1263	1,2	-1,5	-1,3	**0,003**	**0,000**	**0,000**	putative phosphoribosyl-ATP pyrophosphatase / phosphoribosyl-AMP cyclohydrolase
SMU.1264	1,2	-1,5	-1,3	**0,014**	**0,000**	**0,001**	imidazole glycerol phosphate synthase subunit HisF
SMU.1265	1,1	-1,5	-1,4	0,190	**0,000**	**0,000**	1-(5-phosphoribosyl)-5-[(5-phosphoribosylamino)methylideneamino] imidazole-4-carboxamide isomerase
SMU.1266	1,1	-1,5	-1,5	0,198	**0,000**	**0,000**	imidazole glycerol phosphate synthase subunit HisH
SMU.1267c	1,1	-1,6	-1,2	0,482	**0,001**	0,105	hypothetical protein
SMU.1268	1,2	-1,7	-1,3	0,196	**0,000**	**0,042**	imidazoleglycerol-phosphate dehydratase
SMU.1269	1,1	-1,6	-1,4	0,111	**0,000**	**0,000**	putative phosphoserine phosphatase
SMU.1270	1,2	-1,6	-1,4	**0,009**	**0,000**	**0,000**	histidinol dehydrogenase
SMU.1271	1,2	-1,7	-1,5	0,061	**0,000**	**0,000**	ATP phosphoribosyltransferase catalytic subunit
SMU.1272	1,2	-1,6	-1,5	0,138	**0,000**	**0,000**	putative histidyl-tRNA synthetase

SMU.1273	1,2	-1,6	-1,6	0,057	**0,000**	**0,000**	histidinol-phosphate aminotransferase
SMU.1276c	1,2	-1,1	1,3	**0,039**	0,112	**0,001**	septation ring formation regulator EzrA
SMU.1277	1,2	-1,1	1,1	**0,001**	0,439	0,351	DNA gyrase subunit B
SMU.1278c	1,2	1,1	-1,2	0,071	0,338	0,102	hypothetical protein
SMU.1279c	1,1	-1,1	-1,1	0,344	0,338	0,356	putative cell division protein (cell shape determining protein)
SMU.1280c	1,0	1,1	-1,1	0,616	0,244	0,088	hypothetical protein
SMU.1282	-1,0	-1,1	-1,1	0,988	0,254	0,142	putative transcriptional regulator
SMU.1284c	1,2	1,0	-1,2	0,157	0,672	0,075	hypothetical protein
SMU.1286c	-1,2	-1,2	-1,1	0,137	**0,046**	0,120	putative permease; multidrug efflux protein
SMU.1287	-1,0	-1,2	-1,5	0,770	0,214	**0,007**	putative transcriptional regulator
SMU.1288	1,6	1,1	1,2	**0,000**	0,289	**0,007**	50S ribosomal protein L19
SMU.1289c	-1,1	-1,1	-1,2	0,574	0,355	0,154	putative permease, chloride channel
SMU.1290c	-1,1	-1,1	-1,0	0,369	0,370	0,833	putative permease, chloride channel
SMU.1291c	1,3	-1,0	-1,6	0,137	0,913	**0,003**	hypothetical protein
SMU.1292c	-1,0	1,1	-1,0	0,751	0,628	0,982	hypothetical protein
SMU.1293c	-1,1	1,1	-1,2	0,115	0,059	**0,000**	hypothetical protein
SMU.1294	1,4	-1,1	-1,2	**0,026**	0,540	0,177	flavodoxin
SMU.1295	-1,0	-1,0	-1,2	0,771	0,572	**0,028**	adenosine deaminase
SMU.1296	1,0	-1,2	-1,1	0,900	0,120	0,168	putative glutathione S-transferase YghU
SMU.1297	1,1	-1,4	-1,0	0,395	**0,002**	0,654	hypothetical protein
SMU.1298	1,1	1,1	1,3	0,452	0,318	**0,015**	50S ribosomal protein L31 type B
SMU.1299c	1,2	1,3	1,0	**0,035**	**0,002**	0,579	putative acetate kinase
SMU.1300c	1,1	1,2	-1,1	0,188	**0,002**	0,253	hypothetical protein
SMU.1301c	1,2	1,2	1,2	0,065	**0,026**	0,101	putative methyltransferase
SMU.1302	1,1	-1,1	1,1	0,130	0,068	0,322	putative surface adhesin; AdcA protein homolog; putative Zn-binding lipoprotein
SMU.1303c	1,0	1,1	1,1	0,719	0,439	0,461	putative dipeptidase
SMU.1304c	-1,0	-1,1	-1,0	0,754	0,225	0,595	hypothetical protein
SMU.1305c	1,2	1,1	-1,0	0,088	0,556	0,649	hypothetical protein
SMU.1306c	1,1	1,0	-1,1	0,315	0,950	0,332	hypothetical protein
SMU.1307c	1,0	-1,1	-1,2	0,819	0,067	**0,005**	hypothetical protein
SMU.1308	1,1	-1,1	-1,1	0,234	0,270	0,378	putative translation initiation inhibitor; aldR regulator-like protein
SMU.1309c	1,2	-1,1	-1,1	**0,006**	0,425	0,071	putative glycerol dehydrogenase
SMU.1310	1,2	-1,2	1,0	**0,030**	0,132	0,821	hypothetical protein
SMU.1311	1,3	-1,1	-1,0	**0,004**	0,152	0,628	asparaginyl-tRNA synthetase
SMU.1312	1,2	-1,1	-1,1	**0,014**	0,174	**0,035**	aspartate aminotransferase
SMU.1313c	1,1	-1,1	-1,1	0,335	0,157	0,253	bifunctional ATP-dependent DNA helicase/DNA polymerase III subunit epsilon
SMU.1314	1,0	-1,1	1,0	1,000	0,277	0,815	hypothetical protein
SMU.1315c	-1,2	-1,1	1,0	0,283	0,469	0,860	putative ATP-binding protein
SMU.1316c	-1,1	-1,0	1,0	0,459	0,833	0,858	hypothetical protein
SMU.1317c	1,1	-2,1	-1,2	0,710	**0,011**	0,411	hypothetical protein
SMU.1319c	1,1	-1,1	-1,3	0,095	0,154	**0,002**	hypothetical protein
SMU.1321c	1,0	-1,1	-1,3	0,657	0,195	**0,005**	hypothetical protein
SMU.1322	1,1	1,2	-1,3	0,390	0,149	**0,030**	acetoin reductase
SMU.1323	-1,0	1,2	-1,0	0,729	**0,046**	0,676	hydrolase
SMU.1324	1,0	-1,3	1,1	0,874	0,071	0,673	putative cell-division protein FtsX
SMU.1325	1,3	-1,0	1,2	**0,000**	0,967	**0,032**	putative ABC transporter, ATP-binding component
SMU.1326	1,3	1,0	1,1	**0,003**	0,868	0,333	peptide chain release factor 2
SMU.1327c	-1,1	1,1	-1,0	0,229	0,247	0,917	4Fe-4S ferredoxin
SMU.1329c	1,0	1,0	-1,1	0,959	0,577	**0,038**	putative transposase
SMU.1330c	1,1	-1,3	-1,2	0,103	**0,015**	0,171	putative transposase
SMU.1331c	1,1	-1,0	-1,0	0,232	0,549	0,628	putative transposase
SMU.1332c	-1,1	1,1	1,1	**0,022**	0,088	0,094	putative transposase
SMU.1334	-1,1	-1,3	-1,2	0,315	0,187	0,246	putative phosphopantetheinyl transferase
SMU.1335c	1,1	1,0	1,3	0,705	0,849	0,155	putative enoyl-(acyl-carrier-protein) reductase
SMU.1336	-1,1	1,0	1,7	0,381	0,960	**0,001**	hypothetical protein
SMU.1337c	1,2	-1,3	1,8	0,094	0,148	**0,001**	alpha/beta superfamily hydrolase
SMU.1338c	-1,1	-1,1	1,6	0,477	0,580	**0,000**	multidrug ABC transporter permease
SMU.1339	1,0	-1,2	1,4	0,439	**0,016**	**0,000**	putative bacitracin synthetase
SMU.1340	1,0	-1,5	1,4	0,946	**0,000**	**0,000**	putative surfactin synthetase
SMU.1341c	-1,0	-1,7	1,5	0,676	**0,000**	**0,000**	putative gramicidin S synthetase
SMU.1342	-1,1	-2,0	1,4	**0,026**	**0,000**	**0,000**	putative bacitracin synthetase 1; BacA
SMU.1343c	1,1	-1,8	1,4	0,126	**0,000**	**0,000**	putative polyketide synthase

SMU.1344c	1,0	-2,0	1,4	0,490	**0,000**	**0,002**	putative malonyl-CoA acyl-carrier-protein transacylase
SMU.1345c	1,1	-2,1	1,3	0,218	**0,000**	0,050	putative peptide synthetase MycA
SMU.1346	-1,2	-2,3	1,1	0,325	**0,000**	0,783	putative thioesterase BacT
SMU.1347c	1,0	-1,8	1,5	0,627	**0,000**	**0,000**	permease
SMU.1348c	1,0	-1,8	1,1	0,853	**0,001**	0,485	putative ABC transporter, ATP-binding protein
SMU.1349	1,4	1,1	-1,0	**0,027**	0,568	0,969	hypothetical protein
SMU.1351	1,3	-1,1	-1,1	0,077	0,683	0,392	putative putative transposase
SMU.1352	1,0	-1,0	-1,2	0,611	0,841	0,061	putative transposase
SMU.1353	-1,1	1,0	1,0	0,125	0,415	0,950	putative transposase
SMU.1354c	1,2	1,1	1,3	0,413	0,767	0,310	putative putative transposase
SMU.1355c	-1,0	1,0	1,3	0,630	0,947	**0,002**	putative transposase fregment
SMU.1356c	1,1	1,3	1,1	0,515	**0,025**	0,158	putative putative transposase
SMU.1357	-1,2	-1,0	1,3	**0,035**	0,771	**0,015**	putative putative transposase
SMU.1358	1,3	-1,5	1,3	**0,000**	**0,002**	**0,021**	putative putative transposase
SMU.1359	1,1	-2,0	1,2	0,185	**0,000**	0,524	hypothetical protein
SMU.1360c	1,0	-1,4	1,5	0,907	**0,022**	**0,004**	hypothetical protein
SMU.1361c	-1,2	-1,2	1,5	0,309	0,195	**0,003**	TetR family transcriptional regulator
SMU.1363c	1,2	1,2	1,3	0,099	**0,049**	**0,003**	putative transposase
SMU.1365c	1,0	-1,8	1,5	0,589	**0,000**	**0,000**	permease
SMU.1366c	1,0	-1,8	1,1	0,768	**0,001**	0,543	putative ABC transporter; ATP-binding protein
SMU.1367c	-1,2	1,0	1,5	0,188	0,807	**0,000**	hypothetical protein
SMU.1368	-1,3	1,3	1,2	**0,004**	**0,010**	0,236	hypothetical protein
SMU.1369	1,1	1,7	1,0	0,402	**0,000**	0,986	hypothetical protein
SMU.1370c	1,0	-1,0	1,1	0,869	0,917	0,278	putative transposase, IS150-like
SMU.1372c	1,1	1,0	-1,2	0,501	0,585	0,051	hypothetical protein
SMU.1373c	-1,2	1,0	-1,2	0,226	0,698	**0,010**	hypothetical protein
SMU.1374	-1,0	**2,0**	1,1	0,919	**0,000**	0,453	hypothetical protein
SMU.1375c	1,0	-1,1	-1,2	0,549	0,061	**0,004**	hypothetical protein
SMU.1377c	1,2	-1,2	-1,1	**0,027**	**0,034**	0,275	hypothetical protein
SMU.1378	-1,0	1,2	1,0	0,314	**0,000**	0,433	hypothetical protein
SMU.1379	1,0	-1,1	-1,0	0,900	0,487	0,704	hypothetical protein
SMU.1381	-1,2	1,1	-1,1	0,168	0,463	0,300	isopropylmalate isomerase small subunit
SMU.1382	1,1	1,3	-1,2	0,063	**0,000**	**0,001**	isopropylmalate isomerase large subunit
SMU.1383	1,1	1,2	-1,1	**0,014**	**0,001**	**0,013**	3-isopropylmalate dehydrogenase
SMU.1384	1,1	1,2	-1,2	0,320	**0,002**	**0,009**	2-isopropylmalate synthase
SMU.1386	1,1	1,0	-1,0	0,224	0,579	0,906	uridine kinase
SMU.1387	-1,1	-1,1	-1,2	0,329	0,311	**0,000**	putative oxidoreductase
SMU.1388	-1,1	-1,2	-1,0	0,251	0,129	0,746	putative RNA helicase
SMU.1389	-1,0	1,4	1,1	0,849	**0,000**	0,554	hypothetical protein
SMU.1390	-1,2	-1,2	1,0	0,084	0,099	0,997	hypothetical protein
SMU.1391c	1,2	-1,1	1,2	0,264	0,613	0,373	hypothetical protein
SMU.1392c	1,1	-1,1	-1,1	0,134	0,203	0,454	putative acetyltransferase
SMU.1393c	1,1	-1,2	-1,0	0,430	0,069	0,987	hypothetical protein
SMU.1394	1,3	-1,1	-1,1	**0,000**	0,119	0,087	GTP-binding protein LepA
SMU.1395c	1,0	-1,1	-1,1	0,993	0,862	0,698	hypothetical protein
SMU.1396	1,5	1,4	1,6	**0,000**	**0,000**	**0,000**	glucan-binding protein C, GbpC
SMU.1397c	1,7	1,1	-1,0	0,214	0,782	0,927	hypothetical protein
SMU.1398	-1,3	-1,2	1,1	**0,000**	**0,028**	0,502	putative transcriptional regulator
SMU.1399	1,0	-1,2	1,1	0,293	**0,000**	**0,045**	hypothetical protein
SMU.1400c	-1,0	**2,7**	-1,1	0,887	**0,000**	0,589	hypothetical protein
SMU.1402c	-1,1	-1,2	1,2	0,257	0,054	0,111	hypothetical protein
SMU.1403c	1,0	1,0	1,2	0,722	0,787	0,126	hypothetical protein
SMU.1404c	-1,1	1,0	1,1	0,122	0,990	0,121	hypothetical protein
SMU.1405c	-1,1	-1,2	1,4	0,144	**0,000**	**0,000**	hypothetical protein
SMU.1406c	-1,1	-1,1	-1,1	0,055	0,212	0,072	hypothetical protein
SMU.1407c	-1,0	-1,1	1,1	0,830	0,093	0,340	putative transposase, ISSmu1
SMU.1408c	1,0	-1,1	-1,1	0,843	0,500	0,492	hypothetical protein
SMU.1409c	-1,0	-1,0	1,1	0,525	0,609	0,293	putative transcriptional regulator
SMU.1410	-1,0	1,1	-1,1	0,780	**0,041**	**0,017**	putative reductase
SMU.1411	-1,0	-1,1	1,2	0,584	0,276	**0,001**	hypothetical protein
SMU.1412c	-1,0	1,2	1,1	0,691	**0,035**	0,050	putative ABC transporter, membrane protein subunit and ATP-binding protein
SMU.1414c	-1,1	-1,2	-1,1	0,647	0,175	0,578	hypothetical protein
SMU.1415c	1,1	-1,1	-1,1	0,272	0,226	0,376	putative phosphatase involved in N-acetyl-glucosamine catabolism
SMU.1416c	1,2	-1,1	-1,0	0,100	0,398	0,720	putative mutator protein MutT

SMU.1417c	1,1	-1,2	-1,2	0,378	**0,007**	0,091	putative oleoyl-acyl carrier protein thioesterase
SMU.1418	1,1	-1,1	-1,3	0,439	0,439	**0,009**	coproporphyrinogen III oxidase
SMU.1419	-1,0	1,0	1,0	0,978	0,704	0,945	putative transcriptional regulator
SMU.1420	1,0	-1,1	1,1	0,966	0,559	0,525	putative oxidoreductase
SMU.1421	1,0	1,4	-1,2	0,936	**0,000**	**0,000**	branched-chain alpha-keto acid dehydrogenase subunit E2
SMU.1422	1,0	1,5	-1,2	0,510	**0,000**	**0,025**	putative pyruvate dehydrogenase E1 component beta subunit)
SMU.1423	1,1	1,2	-1,1	0,358	**0,004**	0,065	putative pyruvate dehydrogenase, TPP-dependent E1 component alpha-subunit
SMU.1424	-1,1	1,2	-1,4	0,385	**0,032**	**0,000**	putative dihydrolipoamide dehydrogenase
SMU.1425	-1,0	1,0	-1,2	0,373	0,695	**0,000**	putative Clp proteinase, ATP-binding subunit ClpB
SMU.1426c	1,2	1,0	-1,1	**0,002**	0,764	0,184	phosphoglucosamine mutase
SMU.1427c	-1,0	-1,1	-1,0	0,723	0,497	0,755	hypothetical protein
SMU.1428c	1,1	-1,0	-1,1	0,483	0,983	0,405	hypothetical protein
SMU.1429	1,0	-1,0	-1,0	0,947	0,350	0,632	putative UDP-N-acetylmuramyl tripeptide synthetase MurC
SMU.1430	1,0	-1,1	-1,1	0,584	0,146	0,147	putative cobyric acid synthase CobQ
SMU.1431c	1,1	1,0	-1,2	**0,048**	0,525	**0,007**	putative ABC transporter, ATP-binding protein
SMU.1432c	1,2	1,1	-1,0	0,073	0,251	0,767	putative endoglucanase precursor
SMU.1434c	1,1	1,1	-1,0	0,080	0,375	0,856	putative glycosyltransferase
SMU.1435c	1,4	-1,2	1,1	0,142	0,505	0,387	hypothetical protein
SMU.1436c	-1,0	1,0	-1,0	0,988	0,770	0,764	hypothetical protein
SMU.1437	1,1	1,1	-1,0	0,094	0,172	0,931	putative UDP-N-acetylglucosamine 2-epimerase
SMU.1438c	1,0	1,0	1,1	0,845	0,910	0,563	putative Zn-dependent protease
SMU.1442c	1,1	-1,2	1,1	0,563	0,078	0,319	hypothetical protein
SMU.1443c	1,1	-1,1	1,1	0,336	0,387	0,142	putative tributyrin esterase
SMU.1444c	1,0	-1,1	-1,0	0,772	0,261	0,888	hypothetical protein
SMU.1445c	1,1	-1,1	-1,2	0,163	0,201	**0,003**	putative ABC transporter, ATP-binding protein
SMU.1446c	-1,1	-1,1	-1,1	0,645	0,254	0,337	putative ABC transporter, permease protein
SMU.1447c	1,1	-1,2	1,1	0,095	**0,009**	0,349	hypothetical protein
SMU.1449	1,0	1,0	1,1	0,697	0,908	0,099	putative fibronectin/fibrinogen-binding protein
SMU.1450	-1,0	-1,4	1,0	0,759	**0,000**	0,917	putative amino acid permease
SMU.1451	1,2	-1,0	-1,3	0,068	0,853	**0,008**	putative alpha-acetolactate decarboxylase
SMU.1452	1,2	-1,0	-1,1	**0,002**	0,571	0,326	acetolactate synthase
SMU.1453c	1,0	1,1	-1,1	0,661	0,447	0,308	hypothetical protein
SMU.1454c	1,1	1,0	1,0	0,438	0,753	0,728	putative membrane protein; permease
SMU.1455	1,2	1,2	-1,2	0,211	0,269	0,271	mutator protein, pyrophosphohydrolase
SMU.1456c	-1,3	1,1	-2,0	**0,000**	**0,027**	**0,000**	hypothetical protein
SMU.1457	1,2	1,0	1,2	**0,000**	0,771	**0,001**	putative dTDP-glucose-4,6-dehydratase
SMU.1459c	1,4	-1,1	1,1	0,120	0,728	0,756	hypothetical protein
SMU.1460	1,1	-1,1	-1,1	0,655	0,614	0,430	putative dTDP-4-keto-L-rhamnose reductase
SMU.1461	1,1	-1,0	-1,0	0,228	0,847	0,655	putative glucose-1-phosphate thymidyltransferase
SMU.1462c	1,2	1,0	-1,3	**0,023**	0,499	**0,000**	putative oxidoreductase
SMU.1463c	1,1	-1,1	-1,2	0,546	0,487	0,168	hypothetical protein
SMU.1464c	1,2	1,0	-1,4	0,051	0,826	**0,003**	hypothetical protein
SMU.1465c	1,0	1,1	-1,2	0,611	0,113	0,058	replication protein DnaD-like
SMU.1466	1,1	-1,0	-1,1	0,236	0,544	0,068	homoserine O-succinyltransferase
SMU.1467	1,2	1,0	-1,2	0,084	0,749	0,117	adenine phosphoribosyltransferase
SMU.1470c	-1,0	-1,2	-1,0	0,947	**0,005**	0,844	hypothetical protein
SMU.1471c	-1,0	-1,1	-1,2	0,893	0,267	**0,034**	hypothetical protein
SMU.1472	1,1	-1,0	-1,2	0,150	0,455	**0,008**	putative single-strand DNA-specific exonuclease RecJ
SMU.1473c	1,1	-1,2	1,0	0,320	0,217	0,801	putative oxidoreductase
SMU.1474c	1,1	-1,1	-1,1	0,166	0,101	0,102	ribonuclease Z
SMU.1475c	1,1	-1,1	-1,1	0,392	0,455	0,471	hypothetical protein
SMU.1476c	1,1	-1,1	-1,0	0,076	0,409	0,955	putative GTP-binding protein
SMU.1477	1,1	-1,0	-1,2	0,085	0,785	**0,003**	tRNA delta(2)-isopentenylpyrophosphate transferase
SMU.1479	1,2	1,1	-1,0	0,605	0,889	0,905	hypothetical protein
SMU.1480	-1,0	1,1	1,2	0,523	0,079	**0,015**	hypothetical protein
SMU.1482c	1,1	-1,3	-1,3	0,615	**0,044**	**0,046**	hypothetical protein
SMU.1483c	1,2	-1,1	-1,1	0,139	0,343	0,288	hypothetical protein
SMU.1484c	1,1	-1,1	-1,1	**0,033**	0,250	0,054	hypothetical protein
SMU.1485c	1,0	-1,3	1,0	0,616	**0,008**	0,918	putative endonuclease
SMU.1486c	1,1	-1,1	1,1	**0,026**	0,286	**0,021**	hypothetical protein
SMU.1487	-1,0	1,6	-1,3	0,310	**0,000**	**0,000**	hypothetical protein
SMU.1488c	1,3	1,2	-1,2	0,113	0,205	0,169	hypothetical protein

SMU.1489	1,2	1,1	-1,4	**0,023**	0,194	**0,000**	LacX
SMU.1490	1,5	1,1	-1,4	**0,000**	0,246	**0,000**	6-phospho-beta-galactosidase
SMU.1491	1,3	1,0	-1,2	**0,000**	0,503	**0,003**	PTS system, lactose-specific enzyme IIBC EIIBC-LAC)
SMU.1492	1,6	1,2	-1,6	**0,000**	0,173	**0,001**	PTS system, lactose-specific enzyme IIA EIIA-LAC)
SMU.1493	1,6	1,0	-1,5	**0,000**	0,552	**0,000**	tagatose 1,6-diphosphate aldolase
SMU.1494	1,4	1,1	-1,5	**0,003**	0,411	**0,000**	tagatose-6-phosphate kinase
SMU.1495	1,5	-1,0	-1,3	**0,000**	0,997	0,053	galactose-6-phosphate isomerase subunit LacB
SMU.1496	1,5	-1,1	-1,4	**0,006**	0,568	**0,008**	galactose-6-phosphate isomerase subunit LacA
SMU.1498	1,3	-1,0	-1,1	**0,021**	0,709	0,194	lactose repressor
SMU.1499	1,0	1,1	-1,1	0,351	0,076	**0,004**	putative exonuclease RexA
SMU.1500	1,1	1,1	-1,2	**0,010**	0,180	**0,000**	putative exonuclease RexB
SMU.1502c	1,0	-1,0	-1,2	0,928	0,779	0,062	hypothetical protein
SMU.1504c	1,2	-1,1	-1,1	0,097	0,630	0,242	hypothetical protein
SMU.1505c	1,0	-1,4	-1,0	0,979	0,341	0,984	hypothetical protein
SMU.1506c	-1,1	-1,0	1,1	0,287	0,923	0,074	hypothetical protein
SMU.1507c	1,1	1,1	-2,0	0,424	0,471	**0,000**	hypothetical protein
SMU.1508c	1,0	-1,1	1,1	0,814	0,510	0,241	putative coenzyme PQQ synthesis protein
SMU.1509	1,1	-1,0	1,1	0,484	0,745	0,449	putative transcriptional regulator
SMU.1510	1,2	-1,2	-1,2	**0,000**	**0,000**	**0,000**	phenylalanyl-tRNA synthetase subunit beta
SMU.1511c	1,1	-1,4	-1,3	0,448	**0,036**	0,093	putative acetyltransferase
SMU.1512	1,1	-1,1	-1,2	0,085	0,075	**0,019**	phenylalanyl-tRNA synthetase subunit alpha
SMU.1513	1,2	-1,0	1,2	**0,000**	0,610	**0,001**	putative chromosome segregation ATPase; SMC protein
SMU.1514	-1,0	-1,1	-1,2	0,866	0,136	**0,044**	ribonuclease III
SMU.1515	1,3	1,0	1,0	**0,000**	0,879	0,968	hypothetical protein
SMU.1516	1,2	-1,1	1,2	0,078	0,230	0,197	putative histidine kinase CovS; VicK-like protein
SMU.1517	1,3	-1,1	1,2	**0,000**	0,068	**0,011**	putative response regulator CovR; VicR-like protein
SMU.1519	1,2	1,0	1,2	**0,026**	0,641	0,054	putative amino acid ABC transporter, ATP-binding protein
SMU.1520	1,0	-1,1	1,3	0,993	0,425	0,098	putative ABC transporter, glutamine binding protein
SMU.1521	1,1	-1,1	1,2	0,254	0,216	**0,041**	putative amino acid ABC transporter, permease protein
SMU.1522	-1,2	-1,1	1,0	0,172	0,447	0,840	putative amino acid ABC transporter, integral membrane protein
SMU.1523	1,1	1,0	-1,1	0,074	0,627	**0,047**	putative membrane nuclease EndA
SMU.1524c	-1,2	-1,2	1,0	0,225	0,369	0,963	hypothetical protein
SMU.1525	1,3	1,0	-1,1	**0,000**	0,667	**0,032**	UDP-N-acetylglucosamine 1-carboxyvinyltransferase
SMU.1526c	-1,1	1,1	-1,0	0,675	0,680	0,863	hypothetical protein
SMU.1527	1,4	1,1	-1,1	**0,002**	0,648	0,515	F0F1 ATP synthase subunit epsilon
SMU.1528	1,2	-1,0	-1,0	**0,002**	0,444	0,847	F0F1 ATP synthase subunit beta
SMU.1529	-1,0	-1,1	-1,0	0,713	0,311	0,909	F0F1 ATP synthase subunit gamma
SMU.1530	1,2	1,0	-1,0	**0,040**	0,724	0,972	F0F1 ATP synthase subunit alpha
SMU.1531	-1,1	-1,2	-1,0	0,401	0,121	0,844	F0F1 ATP synthase subunit delta
SMU.1532	1,2	1,0	-1,2	0,050	0,488	**0,015**	F0F1 ATP synthase subunit B
SMU.1533	-1,1	-1,1	1,0	0,465	0,519	0,944	F0F1 ATP synthase subunit A
SMU.1534	-1,1	-1,1	-1,3	0,669	0,730	0,217	F0F1 ATP synthase subunit C
SMU.1535	-1,0	1,0	1,0	0,821	0,899	0,938	glycogen phosphorylase
SMU.1536	1,0	1,0	-1,1	0,954	0,669	0,170	glycogen synthase
SMU.1537	-1,1	-1,1	-1,0	0,342	0,528	0,959	putative glycogen biosynthesis protein GlgD
SMU.1538	1,1	1,0	1,0	0,290	0,867	0,929	glucose-1-phosphate adenylyltransferase
SMU.1539	1,0	1,0	-1,1	0,444	0,867	0,062	glycogen branching enzyme
SMU.1541	1,2	-1,1	-1,0	**0,002**	0,095	0,527	putative pullulanase
SMU.1542c	1,2	-1,0	-1,1	0,053	0,909	0,340	putative lipid kinase
SMU.1543	1,2	-1,0	-1,1	**0,000**	0,868	**0,017**	NAD-dependent DNA ligase LigA
SMU.1545c	-1,1	-1,3	-1,1	0,633	0,270	0,675	hypothetical protein
SMU.1546	-1,4	-1,2	1,1	**0,000**	**0,025**	0,243	hypothetical protein
SMU.1547c	1,1	1,1	-1,1	0,207	0,264	0,452	putative response regulator
SMU.1548c	1,2	1,1	-1,2	**0,034**	0,447	**0,024**	putative histidine kinase
SMU.1550c	-1,0	-1,1	-1,1	0,678	0,279	0,376	integral membrane protein
SMU.1551c	1,1	-1,0	-1,1	0,211	0,973	0,117	putative ABC transporter, ATP-binding protein
SMU.1552c	1,1	1,2	-1,3	0,463	**0,044**	**0,003**	hypothetical protein
SMU.1553c	1,0	-1,2	-1,0	0,795	0,090	0,731	hypothetical protein
SMU.1554c	1,2	-1,1	-1,4	0,305	0,662	0,073	hypothetical protein
SMU.1555c	-1,2	-1,2	1,1	0,124	**0,027**	0,567	hypothetical protein
SMU.1556	1,4	1,1	-1,1	**0,000**	0,415	0,101	methionine aminopeptidase
SMU.1557c	1,1	1,0	-1,2	0,519	0,852	0,064	hypothetical protein
SMU.1558c	1,0	1,2	-1,2	0,832	0,119	0,123	putative acetyltransferase
SMU.1560	-1,2	1,0	1,1	0,120	0,971	0,462	hypothetical protein
SMU.1561	1,1	-1,0	-1,1	0,445	0,840	0,252	putative potassium uptake system protein TrkB

SMU.1562	1,0	1,0	-1,3	0,755	0,841	**0,001**	putative potassium uptake protein TrkA
SMU.1563	1,1	1,1	-1,2	0,307	0,061	**0,004**	putative cation-transporting P-type ATPase PacL
SMU.1564	-1,3	1,0	1,2	**0,000**	0,969	**0,014**	putative glycogen phosphorylase
SMU.1565	-1,3	1,1	1,0	**0,002**	0,398	0,592	4-alpha-glucanotransferase
SMU.1566	-1,1	-1,2	-1,3	0,137	0,063	**0,006**	putative maltose operon transcriptional repressor
SMU.1568	-1,4	-1,1	1,3	**0,000**	0,539	**0,001**	putative maltose/maltodextrin ABC transporter, sugar-binding protein MalX
SMU.1569	-1,2	1,0	1,0	**0,016**	0,468	0,548	putative maltose/maltodextrin ABC transporter, permease protein MalF
SMU.1570	-1,1	-1,1	1,1	0,159	0,368	0,280	putative maltose/maltodextrin ABC transporter, MalG permease
SMU.1571	-1,2	1,0	1,0	**0,021**	0,958	0,573	putative ABC transporter, ATP-binding protein, MsmK-like protein
SMU.1572	1,4	1,1	-1,1	**0,000**	0,090	**0,041**	UDP-N-acetylglucosamine 1-carboxyvinyltransferase
SMU.1573	1,3	-1,1	-1,3	**0,000**	0,130	**0,000**	S-adenosylmethionine synthetase
SMU.1574c	1,1	-1,4	1,4	0,244	**0,001**	**0,001**	hypothetical protein
SMU.1575c	-1,0	-1,4	-1,0	0,943	0,067	0,878	hypothetical protein
SMU.1576c	-1,1	-1,3	1,2	0,251	**0,000**	**0,018**	hypothetical protein
SMU.1577c	-1,0	-1,2	1,2	0,297	**0,000**	**0,000**	hypothetical protein
SMU.1578	1,2	1,2	-1,4	0,088	0,078	**0,000**	biotin--protein ligase
SMU.1579	-1,2	-1,2	-1,2	0,369	0,236	0,222	hypothetical protein
SMU.1581	1,2	1,1	1,0	**0,001**	0,353	0,903	DNA polymerase III subunits gamma and tau
SMU.1582c	1,0	-1,0	-1,1	0,802	0,998	0,511	hypothetical protein
SMU.1584c	-1,1	-1,1	1,0	0,185	**0,007**	0,523	myosin-cross-reactive antigen
SMU.1585c	-1,0	-1,0	-1,1	0,899	0,804	0,615	putative transcriptional regulator
SMU.1586	1,3	-1,1	1,0	**0,000**	**0,016**	0,387	threonyl-tRNA synthetase
SMU.1587c	1,2	-1,1	-1,2	**0,003**	0,298	**0,004**	hypothetical protein
SMU.1588c	1,1	-1,0	-1,1	0,316	0,917	0,297	putative hexosyltransferase
SMU.1589c	1,2	-1,0	-1,2	0,059	0,956	**0,025**	putative hexosyltransferase
SMU.1590	1,1	1,2	-1,1	0,265	**0,003**	0,124	cytoplasmic alpha-amylase
SMU.1591	1,1	1,3	-1,0	0,461	**0,000**	0,762	catabolite control protein A, CcpA
SMU.1592	-1,2	-1,2	-1,0	0,092	0,055	0,819	putative dipeptidase PepQ
SMU.1593c	1,1	1,1	-1,1	0,518	0,732	0,729	putative CDP-diglyceride synthetase
SMU.1595	-1,1	-1,2	1,0	0,183	0,139	0,771	putative carbonic anhydrase precursor
SMU.1596	1,1	-1,0	-1,0	0,259	0,840	0,759	cellobiose phosphotransferase system IIC component
SMU.1597c	1,2	-1,2	1,0	**0,004**	**0,036**	0,964	hypothetical protein
SMU.1598	1,0	1,1	-1,1	0,445	**0,003**	**0,034**	cellobiose phosphotransferase system IIA component
SMU.1599	-1,0	1,0	-1,1	0,336	0,696	0,105	putative transcriptional regulator; possible antiterminator
SMU.1600	-1,2	1,0	-1,2	**0,007**	0,963	**0,007**	cellobiose phosphotransferase system IIB component
SMU.1601	-1,1	1,0	-1,2	0,135	0,816	**0,005**	6-phospho-beta-glucosidase
SMU.1602	-1,0	1,2	-1,1	0,661	**0,012**	0,300	putative NAD(P)H-flavin oxidoreductase
SMU.1603	1,1	1,2	1,0	0,719	0,197	0,767	putative lactoylglutathione lyase
SMU.1604c	-1,1	-1,5	-1,0	0,243	**0,001**	0,730	hypothetical protein
SMU.1605	-1,1	1,0	-1,1	**0,048**	0,975	**0,024**	putative MDR permease; transmembrane efflux protein
SMU.1606	1,2	-1,0	-1,3	**0,002**	0,729	**0,000**	SsrA-binding protein
SMU.1607	1,2	-1,1	-1,2	**0,001**	0,303	**0,002**	putative exoribonuclease R (RNase R)
SMU.1609c	-1,2	-1,1	-1,1	0,230	0,454	0,438	preprotein translocase subunit SecG
SMU.1610	-1,3	-1,1	-1,4	0,471	0,791	0,304	50S ribosomal protein L33
SMU.1611c	-1,0	-1,0	-1,1	0,828	0,827	0,144	putative permease; multi-drug resistance efflux pump
SMU.1612c	1,2	1,0	-1,1	**0,046**	0,740	0,166	hypothetical protein
SMU.1613c	1,3	1,1	1,0	**0,028**	0,511	0,907	dephospho-CoA kinase
SMU.1614	1,1	1,1	-1,3	0,254	0,396	**0,007**	formamidopyrimidine-DNA glycosylase
SMU.1615c	1,1	1,1	-1,2	0,292	0,550	0,101	hypothetical protein
SMU.1616c	1,2	-1,0	-1,2	0,157	0,968	0,296	hypothetical protein
SMU.1617	-1,0	-1,1	-1,1	0,763	0,486	0,107	GTP-binding protein Era
SMU.1618	-1,0	-1,0	-1,4	0,797	0,921	0,052	diacylglycerol kinase
SMU.1619c	1,2	1,1	-1,1	0,064	0,194	0,399	putative metalloprotease
SMU.1620	1,2	1,1	-1,2	**0,011**	0,056	**0,016**	putative phosphate starvation-induced protein PhoH
SMU.1621c	-1,0	1,2	-1,5	0,656	0,214	**0,001**	hypothetical protein
SMU.1622	1,1	1,1	-1,2	0,156	**0,041**	**0,003**	methionine sulfoxide reductase A
SMU.1623c	1,0	-1,2	-1,1	0,648	**0,047**	0,354	hypothetical protein
SMU.1624	1,2	-1,1	-1,1	0,225	0,379	0,471	ribosome recycling factor
SMU.1625	1,2	-1,0	-1,0	**0,047**	0,838	0,770	uridylate kinase
SMU.1626	1,2	-1,0	1,3	**0,017**	0,666	**0,001**	50S ribosomal protein L1
SMU.1627	1,4	1,0	1,5	**0,015**	0,777	**0,001**	50S ribosomal protein L11

SMU.1628	1,1	-1,2	-1,1	0,632	0,197	0,567	hypothetical protein
SMU.1629c	1,2	-1,0	-1,2	**0,001**	0,845	**0,002**	putative cell division protein; DNA segregation ATPase
SMU.1631	-1,1	-1,4	1,1	0,367	**0,004**	0,360	putative peptidyl-prolyl cis-trans isomerase
SMU.1632	1,2	-1,0	-1,2	0,174	0,854	0,215	5'-methylthioadenosine/S-adenosylhomocysteine nucleosidase
SMU.1633c	1,1	-1,0	1,0	0,769	0,838	0,972	hypothetical protein
SMU.1634c	1,1	-1,1	-1,1	0,543	0,260	0,670	hypothetical protein
SMU.1635	1,1	-1,0	-1,1	0,067	0,984	0,398	putative UDP-N-acetylglucosamine pyrophosphorylase
SMU.1636c	1,2	1,1	-1,0	0,143	0,280	0,948	hypothetical protein
SMU.1637c	1,1	1,1	-1,2	0,479	0,439	0,161	hypothetical protein
SMU.1638c	1,3	-1,2	-1,8	0,379	0,426	0,051	hypothetical protein
SMU.1639	1,2	1,1	1,0	**0,003**	0,059	0,499	methionyl-tRNA synthetase
SMU.1641c	1,5	-1,1	-1,1	**0,022**	0,616	0,443	hypothetical protein
SMU.1642c	-1,0	1,1	1,1	0,874	0,109	0,127	hypothetical protein
SMU.1643c	1,1	1,1	1,1	0,480	0,734	0,366	hypothetical protein
SMU.1644c	-1,1	-1,4	1,7	0,351	**0,033**	**0,000**	hypothetical protein
SMU.1645	1,1	-1,2	1,2	0,140	0,067	**0,023**	tellurite resistance protein TehB
SMU.1646c	-1,1	-1,2	1,0	0,608	0,166	0,671	hemolysis inducing protein
SMU.1647c	-1,0	1,1	-1,4	0,985	0,475	**0,000**	putative transcriptional regulator
SMU.1648c	-1,1	-1,0	-1,3	0,593	0,905	0,106	hypothetical protein
SMU.1649	1,2	-1,1	1,1	**0,002**	0,054	0,080	putative exodeoxyribonuclease III
SMU.1650	-1,0	-1,0	-1,3	0,677	0,829	**0,000**	putative endonuclease III (DNA repair)
SMU.1651	-1,1	1,7	-1,1	0,089	**0,000**	0,319	putative arsenate reductase
SMU.1652	1,1	1,2	-1,4	0,230	0,067	**0,001**	putative methylated-DNA--protein-cysteine S-methyltransferase
SMU.1653	-1,1	-1,1	-1,1	0,532	0,260	0,384	putative D-3-phosphoglycerate dehydrogenase
SMU.1654c	1,1	1,0	-1,3	0,397	0,853	0,117	putative acetyltransferase
SMU.1655c	-1,0	1,0	-1,0	0,943	0,918	0,925	hypothetical protein
SMU.1656	-1,1	1,0	-1,1	0,554	0,642	0,359	phosphoserine aminotransferase
SMU.1657c	1,3	-1,0	-1,3	**0,036**	0,774	**0,003**	putative nitrogen regulatory protein PII
SMU.1658	1,1	-1,1	-1,2	0,326	0,317	**0,011**	putative ammonium transporter, NrgA protein
SMU.1659c	1,0	-1,1	1,0	0,968	0,256	0,774	hypothetical protein
SMU.1660c	1,1	1,1	-1,5	0,357	0,601	**0,005**	DNA replication intiation control protein YabA
SMU.1661c	1,1	-1,1	1,1	0,644	0,611	0,634	putative signal peptidase II
SMU.1662	1,0	-1,1	1,1	0,990	0,410	0,356	DNA polymerase III subunit delta'
SMU.1663	1,1	1,1	1,0	0,445	0,547	0,987	thymidylate kinase
SMU.1664c	1,1	-1,0	-1,2	0,370	0,935	**0,047**	putative acetoin utilization protein, acetoin dehydrogenase
SMU.1665	1,2	-1,0	-1,2	0,055	0,977	0,068	putative branched chain amino acid ABC transporter, ATP-binding protein
SMU.1666	1,2	1,0	-1,0	0,298	0,995	0,754	putative branched chain amino acid ABC transporter, ATP-binding protein
SMU.1667	1,3	-1,0	-1,0	**0,027**	0,893	0,893	putative branched chain amino acid ABC transporter, permease protein
SMU.1668	1,2	1,0	-1,1	0,073	0,952	0,228	putative branched chain amino acid ABC transporter, permease protein
SMU.1669	1,2	-1,1	1,3	**0,013**	0,088	**0,002**	putative ABC transporter, branched chain amino acid-binding protein
SMU.1670c	1,2	-1,3	1,1	0,225	0,061	0,340	hypothetical protein
SMU.1671c	1,2	1,0	1,1	0,422	0,949	0,533	hypothetical protein
SMU.1672	1,1	-1,0	1,2	0,455	0,964	0,135	ATP-dependent Clp protease proteolytic subunit
SMU.1673	1,0	-1,1	-1,2	0,891	0,528	0,157	uracil phosphoribosyltransferase
SMU.1674	-1,0	1,1	-1,2	0,895	0,128	**0,008**	putative aminotransferase; beta-cystathionase
SMU.1675	-1,1	1,1	-1,2	0,393	0,203	0,117	cystathionine gamma-synthase
SMU.1676c	-1,1	-1,0	-1,1	0,318	0,610	0,288	hypothetical protein
SMU.1677	-1,3	1,3	-1,1	**0,004**	**0,002**	0,142	UDP-N-acetylmuramoylalanyl-D-glutamate--L-lysine ligase
SMU.1678	-1,2	1,3	1,0	**0,007**	**0,000**	0,603	acyl-CoA thioesterase
SMU.1679c	-1,1	1,1	-1,1	0,240	0,404	0,597	hypothetical protein
SMU.1680c	-1,1	1,1	-1,2	0,526	0,579	0,184	hypothetical protein
SMU.1681c	1,1	1,2	-1,1	0,235	0,061	0,303	hypothetical protein
SMU.1682c	1,1	-1,1	-1,2	0,196	0,156	**0,003**	intracellular protease
SMU.1683c	1,0	-1,1	-1,0	0,823	0,128	0,609	hypothetical protein
SMU.1685c	1,0	-1,1	-1,1	0,990	0,565	0,630	hypothetical protein
SMU.1687	1,1	-1,0	1,1	0,122	0,760	0,473	putative manganese-dependent inorganic pyrophosphatase

SMU.1688	1,0	-1,1	1,1	0,989	0,389	0,487	putative extramembranal protein, DltD protein
SMU.1689	1,1	-1,1	-1,4	0,746	0,793	0,209	D-alanine--poly(phosphoribitol) ligase subunit 2
SMU.1690	-1,1	-1,2	-1,0	0,365	0,067	0,958	hypothetical protein
SMU.1691	1,0	-1,0	-1,0	0,588	0,839	0,755	D-alanine--poly(phosphoribitol) ligase subunit 1
SMU.1692	1,1	-1,1	-1,3	0,298	0,400	0,059	pyruvate-formate lyase activating enzyme
SMU.1693	1,2	-1,0	-1,1	**0,031**	0,808	0,556	putative hemolysin
SMU.1694c	-1,3	-1,2	-1,3	**0,035**	0,068	**0,009**	putative permease
SMU.1695	-1,1	-1,0	-1,0	0,341	0,687	0,696	molybdenum ABC transporter ATP-binding protein
SMU.1697c	1,0	1,1	1,0	0,720	0,735	0,770	hypothetical protein
SMU.1699c	1,2	1,2	-1,1	0,146	0,110	0,339	hypothetical protein
SMU.1700c	-1,1	-1,1	-1,2	0,572	0,607	0,214	LrgB family protein
SMU.1701c	1,0	-1,2	-1,4	0,800	0,092	**0,003**	hypothetical protein
SMU.1702c	-1,0	-1,2	-1,0	0,856	0,240	0,962	putative phosphatase
SMU.1703c	-1,0	-1,1	-1,1	0,899	0,546	0,678	hypothetical protein
SMU.1704	1,1	1,4	1,3	0,412	0,067	**0,029**	hypothetical protein
SMU.1705	-1,1	1,2	1,2	0,655	0,077	0,129	hypothetical protein
SMU.1706	-1,1	1,1	1,4	0,459	0,390	**0,007**	hypothetical protein
SMU.1707c	-1,1	-1,1	-1,3	0,464	0,508	0,190	putative rRNA methylase
SMU.1708	-1,1	-1,0	-1,1	0,589	0,771	0,322	potassium transporter peripheral membrane component
SMU.1709	-1,1	-1,0	1,1	0,286	0,690	0,138	putative potassium uptake protein TrkH
SMU.1710c	-1,0	-1,1	1,2	0,961	0,721	0,486	hypothetical protein
SMU.1711	1,3	1,0	1,1	**0,014**	0,832	0,433	putative pseudouridylate synthase B, large subunit
SMU.1712c	1,0	-1,1	1,1	0,887	0,243	0,276	segregation and condensation protein B
SMU.1713c	-1,0	-1,1	1,2	0,988	0,232	0,156	segregation and condensation protein A
SMU.1714c	-1,1	-1,3	1,2	0,725	0,224	0,211	site-specific tyrosine recombinase XerD-like protein
SMU.1715c	1,1	-1,1	1,1	0,777	0,841	0,725	hypothetical protein
SMU.1716c	1,1	-1,0	1,1	0,279	0,751	0,152	hypothetical protein
SMU.1717c	1,2	1,0	-1,0	0,097	0,957	0,825	putative deoxyribonucleotide triphosphate pyrophosphatase/unknown domain fusion protein
SMU.1718	1,0	1,0	1,1	0,560	0,780	0,509	glutamate racemase
SMU.1719c	-1,0	-1,1	-1,1	0,972	0,677	0,510	hypothetical protein
SMU.1721c	1,1	1,0	-1,0	0,371	0,866	0,587	putative diaminopimelate decarboxylase
SMU.1722c	1,0	-1,1	-1,0	0,833	0,312	0,733	putative integral membrane protein
SMU.1723c	1,3	1,2	-1,1	0,064	0,155	0,448	hypothetical protein
SMU.1724c	1,0	1,1	-1,1	0,995	0,415	0,716	putative rRNA methylase
SMU.1725	-1,2	-1,0	-1,3	0,373	0,910	0,122	acylphosphatase
SMU.1727	-1,0	1,1	1,1	0,918	0,308	0,386	OxaA-like protein precursor
SMU.1728	1,2	-1,2	1,3	0,176	0,108	0,057	transcription elongation factor GreA
SMU.1729c	1,1	-1,2	1,4	0,380	**0,018**	**0,001**	putative aminodeoxychorismate lyase (fragment)
SMU.1730c	1,0	-1,3	-1,1	0,798	**0,025**	0,403	putative acetyltransferase
SMU.1731	-1,0	-1,2	1,1	0,627	**0,045**	0,216	UDP-N-acetylmuramate--L-alanine ligase
SMU.1732c	-1,1	-1,3	1,4	0,628	0,077	**0,034**	hypothetical protein
SMU.1733c	1,0	-1,1	-1,0	0,647	0,128	0,807	putative SNF helicase
SMU.1734	1,3	1,1	-1,0	0,075	0,749	0,811	acetyl-CoA carboxylase subunit alpha
SMU.1735	1,2	-1,0	1,0	0,136	0,882	0,846	acetyl-CoA carboxylase subunit beta
SMU.1736	1,3	-1,0	1,0	**0,001**	0,475	0,889	acetyl-CoA carboxylase biotin carboxylase subunit
SMU.1737	1,2	-1,1	-1,1	0,167	0,433	0,435	(3R)-hydroxymyristoyl-ACP dehydratase
SMU.1738	1,4	1,0	-1,2	**0,006**	0,735	0,090	acetyl-CoA carboxylase biotin carboxyl carrier protein subunit
SMU.1739	1,3	-1,0	1,0	**0,031**	0,871	0,700	3-oxoacyl-(acyl carrier protein) synthase II
SMU.1740	1,2	-1,0	-1,0	0,071	0,953	0,936	3-ketoacyl-(acyl-carrier-protein) reductase
SMU.1741	1,2	-1,1	1,1	0,062	0,261	0,509	acyl-carrier-protein S-malonyltransferase
SMU.1742c	1,2	-1,1	1,0	0,135	0,667	0,761	putative trans-2-enoyl-ACP reductase
SMU.1743	1,2	-1,1	1,1	0,402	0,731	0,556	acyl carrier protein
SMU.1744	1,2	-1,0	1,0	**0,013**	0,968	0,899	3-oxoacyl-(acyl carrier protein) synthase III
SMU.1745c	1,2	-1,1	-1,1	0,251	0,608	0,321	putative transcriptional regulator
SMU.1746c	1,2	1,1	-1,0	**0,036**	0,247	0,805	enoyl-CoA hydratase
SMU.1747c	1,1	-1,0	-1,0	0,627	0,866	0,973	putative phosphatase
SMU.1748	-1,1	-1,1	-1,0	0,315	0,345	0,753	aspartate kinase
SMU.1750c	-1,2	-1,2	-1,1	0,704	0,496	0,692	hypothetical protein
SMU.1752c	1,1	1,1	1,1	0,420	0,348	0,290	hypothetical protein
SMU.1753c	1,4	1,2	1,1	**0,019**	0,187	0,470	hypothetical protein
SMU.1754c	-1,0	1,0	1,0	0,979	0,906	0,847	hypothetical protein
SMU.1755c	1,2	1,1	1,3	0,175	0,385	0,076	hypothetical protein
SMU.1757c	1,2	-1,0	1,2	0,132	0,916	0,283	hypothetical protein
SMU.1758c	1,2	1,1	1,1	0,300	0,526	0,369	hypothetical protein

SMU.1760c	1,5	1,1	1,2	**0,000**	0,172	**0,007**	hypothetical protein
SMU.1761c	1,1	-1,1	1,5	0,358	0,196	**0,000**	hypothetical protein
SMU.1762c	1,2	-1,2	1,1	0,480	0,415	0,689	hypothetical protein
SMU.1763c	1,4	1,1	1,3	**0,001**	0,212	**0,018**	hypothetical protein
SMU.1764c	1,2	-1,0	1,4	0,058	0,758	**0,000**	hypothetical protein
SMU.1765c	1,1	-1,1	-1,0	0,556	0,173	0,582	hypothetical protein
SMU.1766c	1,1	-1,3	1,0	0,589	0,096	0,733	hypothetical protein
SMU.1767c	1,2	-1,2	1,3	0,490	0,516	0,491	hypothetical protein
SMU.1768c	1,3	-1,1	-1,2	0,096	0,592	0,325	hypothetical protein
SMU.1770	1,2	-1,2	1,1	**0,000**	**0,000**	**0,014**	valyl-tRNA synthetase
SMU.1771c	-1,1	-1,4	-1,0	0,670	**0,033**	0,859	hypothetical protein
SMU.1772c	1,2	-1,4	1,3	0,187	**0,017**	**0,046**	hypothetical protein
SMU.1773c	1,0	-1,3	1,1	0,727	0,054	0,286	hypothetical protein
SMU.1774c	1,1	1,0	-1,1	0,175	0,956	0,486	hypothetical protein
SMU.1775c	1,2	-1,0	1,3	0,544	0,982	0,237	hypothetical protein
SMU.1776c	-1,0	-1,2	-1,1	0,987	0,356	0,550	hypothetical protein
SMU.1777	-1,0	-1,1	1,2	0,786	0,407	0,350	flavoprotein NrdI
SMU.1779c	1,0	-1,1	-1,0	0,633	0,067	0,825	putative RNA methyltransferase
SMU.1780	-1,3	-1,2	1,2	**0,000**	**0,021**	**0,021**	recombination regulator RecX
SMU.1781	-1,0	-1,2	1,0	0,995	0,230	0,871	hypothetical protein
SMU.1782	1,1	-1,2	1,5	0,678	0,391	**0,031**	hypothetical protein
SMU.1783	1,2	-1,1	-1,1	**0,014**	0,215	0,289	prolyl-tRNA synthetase
SMU.1784c	1,1	-1,1	-1,1	0,113	0,123	0,264	membrane-associated Zn-dependent protease
SMU.1785	1,1	-1,1	-1,0	0,297	0,448	0,728	putative phosphatidate cytidylyltransferase synthase)
SMU.1786	1,2	-1,0	-1,0	0,153	0,804	0,918	undecaprenyl pyrophosphate synthase
SMU.1787c	1,3	-1,0	-1,1	0,068	0,961	0,645	preprotein translocase subunit YajC
SMU.1788c	1,1	-1,1	-1,1	0,461	0,427	0,457	putative bacterocin transport accessory protein, Bta
SMU.1789c	1,3	-1,0	-1,2	**0,001**	0,930	**0,009**	hypothetical protein
SMU.1790c	1,2	1,2	-1,3	0,197	0,267	0,051	putative transcriptional regulator
SMU.1791c	1,1	1,1	-1,1	**0,011**	0,132	0,334	hypothetical protein
SMU.1792c	-1,0	1,2	1,2	0,892	0,086	0,237	hypothetical protein
SMU.1794c	1,1	1,0	-1,1	0,344	0,813	0,215	hypothetical protein
SMU.1795c	-1,0	1,0	-1,1	0,978	0,831	0,264	hypothetical protein
SMU.1797c	1,4	1,1	-1,0	**0,003**	0,341	0,888	hypothetical protein
SMU.1798c	1,1	1,1	-1,2	0,309	0,576	0,193	hypothetical protein
SMU.1799	1,1	-1,1	-1,1	0,575	0,585	0,339	nicotinic acid mononucleotide adenylyltransferase
SMU.1800c	1,2	-1,0	1,0	0,153	0,890	0,985	hypothetical protein
SMU.1801c	1,2	1,0	-1,1	0,069	0,975	0,154	GTP-binding protein YqeH
SMU.1802c	1,4	1,1	1,0	**0,001**	0,293	0,911	hypothetical protein
SMU.1803c	1,1	-1,2	1,1	0,520	0,217	0,473	hypothetical protein
SMU.1804c	-1,4	-1,5	-1,9	0,284	0,323	0,117	hypothetical protein
SMU.1805	1,1	1,1	1,0	0,381	0,465	0,857	putative transcriptional regulator
SMU.1806	-1,0	-1,0	1,0	0,256	0,723	0,258	putative glycosyltransferase
SMU.1807c	1,0	-1,0	-1,3	0,729	0,754	**0,004**	putative integral membrane protein, permease
SMU.1808c	1,1	-1,1	-2,1	0,490	0,217	**0,000**	putative integrase fragment
SMU.1809	1,0	1,1	-1,4	0,742	0,548	**0,001**	putative bacteroiocin operon protein ScnG-like protein
SMU.1810	-1,1	1,0	-1,1	0,337	0,996	0,103	putative bacteriocin operon component, ScnE-like protein
SMU.1811	-1,0	1,0	-1,3	0,835	0,941	**0,000**	putative bacteriocin component ScnF homolog, putative ABC transporter, ATP-binding protein
SMU.1812	-1,0	-1,0	-1,0	0,262	0,886	0,538	putative transposase, ISSmu2
SMU.1813	-1,1	1,3	-1,0	**0,000**	**0,023**	0,839	putative putative transposase
SMU.1814	-1,0	-1,2	1,0	0,739	**0,002**	0,541	putative histidine kinase, ScnK-like protein
SMU.1815	-1,0	-1,2	-1,1	0,999	0,113	0,313	putative response regulator; ScnR-like protein
SMU.1816c	1,2	1,0	-1,2	0,247	0,907	0,157	putative maturase-related protein
SMU.1817c	1,2	-1,2	1,1	**0,025**	0,140	**0,021**	putative maturase-related protein
SMU.1818c	1,1	-1,1	-1,3	0,814	0,811	0,605	hypothetical protein
SMU.1819	1,1	-1,2	1,1	0,197	0,072	0,184	aspartyl/glutamyl-tRNA amidotransferase subunit B
SMU.1820c	1,1	-1,1	1,0	0,368	0,097	0,730	aspartyl/glutamyl-tRNA amidotransferase subunit A
SMU.1821c	1,1	-1,2	-1,1	0,339	0,165	0,456	aspartyl/glutamyl-tRNA amidotransferase subunit C
SMU.1822	1,2	-1,1	-1,2	**0,000**	**0,011**	**0,000**	aspartyl-tRNA synthetase
SMU.1823	1,3	1,1	1,0	0,096	0,362	0,862	putative pyrazinamidase/nicotinamidase
SMU.1824c	1,1	-1,0	1,1	0,396	0,994	0,648	transcriptional repressor CodY
SMU.1826	1,1	1,0	-1,1	0,364	0,749	0,415	aminotransferase AlaT
SMU.1827	-1,2	-1,1	-1,1	0,119	0,285	0,593	putative biotin biosynthesis protein
SMU.1828	-1,1	1,1	1,1	0,439	0,609	0,610	hypothetical protein

SMU.1830c	1,0	1,1	-1,2	0,566	0,112	**0,023**	hypothetical protein
SMU.1831	1,1	1,0	-1,0	0,077	0,504	0,617	putative L-asparaginase
SMU.1832	-1,4	-1,4	1,0	**0,003**	**0,031**	0,936	hypothetical protein
SMU.1833	1,1	1,3	-1,2	0,111	**0,000**	**0,002**	putative ATP-dependent DNA helicase, RecG
SMU.1834	1,3	1,2	-1,0	**0,000**	**0,003**	0,480	alanine racemase
SMU.1835	1,1	1,0	-1,2	0,515	0,968	0,329	4'-phosphopantetheinyl transferase
SMU.1836	1,1	-1,0	1,0	0,630	0,937	0,872	phospho-2-dehydro-3-deoxyheptonate aldolase
SMU.1837	1,0	1,1	-1,1	0,948	0,687	0,675	phospho-2-dehydro-3-deoxyheptonate aldolase
SMU.1838	1,2	1,1	1,1	**0,000**	0,164	0,257	preprotein translocase subunit SecA
SMU.1839	1,2	-1,0	-1,0	0,065	0,730	0,716	mannose-6-phosphate isomerase
SMU.1840	1,0	-1,1	-1,0	0,726	0,365	0,967	putative fructokinase
SMU.1841	-1,2	1,2	-1,1	**0,001**	**0,005**	**0,047**	putative PTS system, sucrose-specific IIABC component
SMU.1843	-1,3	1,0	-1,0	**0,000**	0,838	0,775	sucrose-6-phosphate hydrolase
SMU.1844	-1,3	-1,1	1,1	**0,000**	0,127	0,221	sucrose operon repressor
SMU.1845	1,1	-1,1	-1,1	0,435	0,456	0,722	transcription antitermination protein NusB
SMU.1846c	1,4	-1,0	1,1	**0,000**	0,703	0,327	hypothetical protein
SMU.1847	1,0	-1,2	-1,0	0,742	0,130	0,891	elongation factor P
SMU.1848	-1,1	1,1	1,1	0,352	0,414	0,525	hypothetical protein
SMU.1849	1,2	1,2	1,2	0,131	0,172	0,188	putative deoxycytidylate deaminase
SMU.1850	1,3	1,2	1,0	**0,004**	**0,034**	0,763	putative aminopeptidase P
SMU.1851	1,2	1,2	-1,0	**0,000**	**0,002**	0,776	excinuclease ABC subunit A
SMU.1852	-1,0	-1,1	1,0	0,776	0,289	0,557	putative magnesium/cobalt transport protein
SMU.1853	-1,3	-1,1	1,2	**0,039**	0,385	0,135	hypothetical protein
SMU.1854	-1,3	-1,1	1,3	0,094	0,614	0,153	hypothetical protein
SMU.1855	-1,1	1,0	1,5	0,258	0,789	**0,000**	hypothetical protein
SMU.1856c	1,0	1,0	-1,0	0,799	0,675	0,685	hypothetical protein
SMU.1858	1,5	-1,0	1,4	**0,018**	0,872	0,088	30S ribosomal protein S18
SMU.1859	1,5	1,2	1,2	**0,002**	0,244	0,171	single-stranded DNA-binding protein
SMU.1860	1,2	1,1	1,3	0,130	0,161	**0,049**	30S ribosomal protein S6
SMU.1861c	-1,5	-1,9	-1,0	**0,004**	**0,000**	0,960	hypothetical protein
SMU.1862	1,1	1,1	-1,4	0,612	0,542	0,076	hypothetical protein
SMU.1865	-1,0	-1,0	1,0	0,557	0,535	0,573	putative A/G-specific DNA glycosylase
SMU.1867c	1,4	-1,1	-1,2	**0,000**	0,311	**0,011**	putative alcohol dehydrogenase
SMU.1869	1,1	-1,1	-1,1	0,492	0,706	0,584	putative thioredoxin
SMU.1870	1,1	-1,1	1,0	0,091	0,069	0,803	putative DNA mismatch repair protein MutS2
SMU.1871c	-1,0	-1,4	-1,1	0,717	**0,002**	0,351	hypothetical protein
SMU.1872c	-1,1	-1,4	-1,4	0,628	**0,006**	**0,009**	hypothetical protein
SMU.1873	-1,1	-1,1	1,1	0,608	0,311	0,482	ribonuclease HIII
SMU.1874	1,0	-1,1	1,1	0,779	0,228	0,313	putative signal peptidase I
SMU.1875	1,0	-1,1	1,1	0,616	**0,005**	**0,010**	putative exodeoxyribonuclease V, conjugation transfer protein
SMU.1876	-1,3	-1,4	1,1	0,159	0,122	0,516	hypothetical protein
SMU.1877	-1,1	-1,0	1,2	0,117	0,800	0,059	putative PTS system, mannose-specific component IIAB
SMU.1878	-1,2	-1,2	1,3	0,066	0,102	**0,009**	putative PTS system, mannose-specific component IIC
SMU.1879	-1,1	-1,1	1,1	0,272	0,157	0,188	putative PTS system, mannose-specific component IID
SMU.1881c	1,0	-1,3	-1,3	0,644	**0,000**	**0,000**	putative ABC transporter, ATP-binding protein
SMU.1882c	-1,1	-1,5	-1,7	0,467	**0,015**	**0,004**	hypothetical protein
SMU.1883	-1,2	-1,1	-1,2	0,235	0,642	0,163	hypothetical protein
SMU.1884c	-1,1	-1,1	-1,1	0,078	0,293	0,145	hypothetical protein
SMU.1886	1,1	-1,3	-1,1	0,291	**0,000**	0,055	seryl-tRNA synthetase
SMU.1888	-1,1	1,1	1,0	0,134	0,284	0,733	putative transposase
SMU.1889c	1,1	-1,0	-1,5	0,501	0,909	**0,041**	hypothetical protein
SMU.1891c	-1,0	1,1	-1,4	0,944	0,656	**0,004**	hypothetical protein
SMU.1892c	-1,1	1,1	-1,0	0,275	0,130	0,775	hypothetical protein
SMU.1893c	-1,0	-1,2	1,1	0,946	0,065	0,289	putative transposase, ISSmu1
SMU.1894c	-1,0	-1,1	-1,1	0,979	0,413	0,610	hypothetical protein
SMU.1895c	1,3	-1,7	-1,4	0,528	0,207	0,495	hypothetical protein
SMU.1896c	1,6	-1,4	-1,5	**0,009**	**0,010**	**0,034**	hypothetical protein
SMU.1897	-1,0	1,7	1,0	0,986	**0,000**	0,936	putative ABC transporter, ATP-binding protein
SMU.1898	-1,1	1,1	1,1	0,112	**0,041**	0,272	putative ABC transporter, ATP-binding and permease protein
SMU.1899	-1,2	1,2	-1,0	0,084	0,199	0,991	putative ABC transporter, ATP-binding and permease protein (fragment)
SMU.1900	1,0	1,1	-1,0	0,523	**0,046**	0,724	hypothetical protein
SMU.1902c	**2,1**	**2,5**	1,4	**0,000**	**0,000**	**0,014**	hypothetical protein
SMU.1903c	**12,4**	**16,0**	**24,3**	**0,000**	**0,000**	**0,000**	hypothetical protein

SMU.1904c	**21,1**	**49,7**	**61,1**	**0,000**	**0,000**	**0,000**	hypothetical protein
SMU.1905c	**26,4**	**47,8**	**43,8**	**0,000**	**0,000**	**0,000**	putative bacteriocin secretion protein
SMU.1906c	**21,3**	**34,0**	**45,1**	**0,000**	**0,000**	**0,000**	hypothetical protein
SMU.1907	1,0	-1,1	1,2	0,904	**0,002**	**0,016**	hypothetical protein
SMU.1908c	**16,4**	**57,1**	**35,8**	**0,000**	**0,000**	**0,000**	hypothetical protein
SMU.1909c	**17,6**	**55,1**	**85,3**	**0,000**	**0,000**	**0,000**	hypothetical protein
SMU.1910c	**15,5**	**39,2**	**61,6**	**0,000**	**0,000**	**0,000**	hypothetical protein
SMU.1912c	**16,4**	**29,9**	**67,0**	**0,000**	**0,000**	**0,000**	hypothetical protein
SMU.1913c	**13,6**	**29,3**	**49,4**	**0,000**	**0,000**	**0,000**	putative immunity protein, BLpL-like
SMU.1914c	**15,9**	**17,4**	**31,0**	**0,000**	**0,000**	**0,000**	hypothetical protein
SMU.1915	-1,1	1,0	1,4	0,887	0,967	0,452	competence stimulating peptide, precursor
SMU.1916	1,4	**6,3**	1,7	**0,006**	**0,000**	**0,000**	putative histidine kinase of the competence regulon, ComD
SMU.1917	1,5	**10,0**	1,9	**0,000**	**0,000**	**0,000**	putative response regulator of the competence regulon, ComE; response regulator of sakacin A production
SMU.1918	1,0	1,2	1,2	0,804	0,247	0,262	putative membrane-associated protein DedA
SMU.1919	1,1	1,3	-1,1	0,334	**0,000**	0,261	hypothetical protein
SMU.1920	1,4	1,4	1,0	**0,000**	**0,000**	0,669	GTP-binding protein EngA
SMU.1921	1,2	-1,1	1,0	**0,020**	0,341	0,629	primosomal protein DnaI
SMU.1922	1,0	-1,2	1,2	0,746	0,120	0,088	putative chromosome replication protein
SMU.1923c	1,1	-1,1	-1,0	0,389	0,436	0,995	transcriptional regulator NrdR
SMU.1924	1,2	-1,1	1,0	0,061	0,590	0,803	response regulator GcrR for glucan-binding protein C
SMU.1925c	1,1	-1,1	1,0	0,437	0,678	0,766	hypothetical protein
SMU.1926	1,1	-1,0	1,6	0,486	0,819	**0,009**	putative transcriptional regulator
SMU.1927	-1,0	1,1	1,3	0,717	0,137	**0,001**	putative ABC transporter, ATP-binding protein
SMU.1928	-1,1	1,0	1,2	0,112	0,338	**0,000**	putative ABC transporter, permease protein
SMU.1929	1,3	1,1	1,0	**0,019**	0,453	0,797	heat shock protein HtpX
SMU.1930	-1,0	-1,1	-1,0	0,974	0,562	0,842	putative cytoplasmic membrane protein; LemA-like protein
SMU.1931	-1,1	-1,1	-1,0	0,133	0,230	0,625	16S rRNA methyltransferase GidB
SMU.1933c	-1,1	1,1	-1,1	0,528	0,255	0,213	cobalt permease
SMU.1934c	-1,0	1,2	-1,1	0,970	**0,002**	0,166	putative cobalt ABC transporter, ATP-binding protein
SMU.1935c	-1,2	1,1	-1,2	0,320	0,691	0,338	hypothetical protein
SMU.1936c	-1,0	1,2	-1,1	0,653	0,050	0,261	hypothetical protein
SMU.1937	1,0	1,2	-1,4	0,592	**0,037**	**0,000**	putative carbon-nitrogen hydrolase
SMU.1938c	-1,1	-1,0	-1,2	0,597	0,985	0,150	putative ABC transporter, permease protein
SMU.1939c	1,0	1,1	-1,2	0,932	0,253	0,057	putative ABC transporter, ATP-binding protein
SMU.1940c	-1,0	1,0	-1,1	0,606	0,968	0,482	hypothetical protein
SMU.1941	-1,1	-1,3	1,2	0,455	**0,034**	0,086	putative membrane lipoprotein
SMU.1942c	-1,0	-1,1	1,3	0,609	0,521	**0,003**	putative amino acid binding protein
SMU.1943	1,1	-1,1	1,0	**0,003**	0,082	0,605	leucyl-tRNA synthetase
SMU.1945	-1,1	-1,2	1,3	0,314	0,054	**0,012**	hypothetical protein
SMU.1946	-1,3	-1,2	1,2	**0,028**	0,201	0,209	hypothetical protein
SMU.1947	-1,1	-1,1	-1,1	0,617	0,633	0,237	transcription antitermination protein NusG
SMU.1948	-1,1	1,2	-1,4	0,594	0,451	0,135	preprotein translocase subunit SecE
SMU.1949	1,1	1,0	-1,0	**0,028**	0,609	0,785	putative membrane carboxypeptidase, penicillin-binding protein 2a
SMU.1950	-1,1	-1,0	1,0	0,080	0,676	0,615	putative pseudouridylate synthase
SMU.1951c	1,1	1,1	1,1	**0,024**	0,105	**0,006**	hypothetical protein
SMU.1954	1,3	1,3	1,1	**0,001**	**0,003**	0,162	chaperonin GroEL
SMU.1955	1,3	1,2	-1,0	0,168	0,362	0,848	co-chaperonin GroES
SMU.1956c	-1,1	-1,1	1,1	0,532	0,478	0,429	hypothetical protein
SMU.1957	-1,1	-1,1	1,2	0,464	0,714	0,178	putative PTS system, mannose-specific IID component
SMU.1958c	-1,0	1,1	1,2	0,752	0,286	**0,041**	putative PTS system, mannose-specific IIC component
SMU.1960c	-1,2	-1,1	-1,2	0,174	0,316	0,242	putative PTS system, mannose-specific IIB component
SMU.1961c	-1,1	1,1	-1,4	0,247	0,331	**0,001**	putative PTS system, sugar-specific enzyme IIA component
SMU.1963c	-1,0	1,9	1,1	0,688	**0,000**	0,583	putative sugar-binding periplasmic protein
SMU.1964c	-1,1	**2,0**	-1,0	0,567	**0,000**	0,829	putative response regulator
SMU.1965c	1,1	**2,4**	1,0	0,627	**0,000**	0,934	putative histidine kinase
SMU.1966c	-1,1	**3,7**	-1,2	0,189	**0,000**	**0,013**	putative periplasmic sugar-binding protein
SMU.1967	1,4	**52,8**	-1,1	**0,001**	**0,000**	0,364	single-stranded DNA-binding protein
SMU.1968c	1,1	-1,2	-1,5	0,511	0,051	**0,000**	hypothetical protein
SMU.1969c	-1,0	-1,1	-1,3	0,718	0,070	**0,001**	putative transcriptional regulator
SMU.1970c	1,2	-1,1	-1,2	0,118	0,571	**0,040**	putative phenylalanyl-tRNA synthetase, beta subunit
SMU.1971c	1,2	-1,0	-1,2	0,067	0,758	**0,011**	putative thioredoxin H1

SMU.1972c	1,1	-1,1	-1,6	0,521	0,596	**0,016**	hypothetical protein
SMU.1973	-1,1	-1,2	-1,1	0,396	**0,026**	0,178	putative glutamyl-aminopeptidase; endo-1,4-beta-glucanase
SMU.1974	-1,2	-1,4	-1,0	**0,016**	**0,000**	0,497	pyrroline-5-carboxylate reductase
SMU.1975c	-1,0	1,6	-1,2	0,866	**0,002**	0,243	hypothetical protein
SMU.1976c	-1,0	1,6	1,1	0,927	**0,019**	0,572	hypothetical protein
SMU.1977c	1,5	1,9	1,1	0,062	**0,001**	0,820	putative transcriptional regulator
SMU.1978	1,3	**7,5**	1,1	**0,011**	**0,000**	0,135	putative acetate kinase
SMU.1979c	1,2	**29,8**	-1,3	0,052	**0,000**	**0,034**	hypothetical protein
SMU.1980c	1,4	**102,3**	-1,2	**0,001**	**0,000**	**0,014**	hypothetical protein
SMU.1981c	1,2	**68,6**	1,0	0,135	**0,000**	0,988	hypothetical protein
SMU.1982c	1,1	**111,6**	-1,1	0,739	**0,000**	0,305	hypothetical protein
SMU.1983	1,0	**68,8**	-1,0	0,754	**0,000**	0,790	putative competence protein ComYD
SMU.1984	1,5	**70,0**	-1,3	**0,007**	**0,000**	**0,003**	putative competence protein ComYC
SMU.1985	1,4	**69,8**	1,1	**0,000**	**0,000**	0,409	ABC transporter ComYB
SMU.1987	1,6	**106,0**	-1,1	**0,000**	**0,000**	0,317	putative ABC transporter, ATP-binding protein ComYA; late competence gene
SMU.1988c	1,1	1,0	-1,0	0,710	0,845	0,908	putative DNA binding protein
SMU.1989	1,4	1,2	1,1	**0,000**	**0,000**	**0,027**	DNA-directed RNA polymerase subunit beta'
SMU.1990	1,3	1,2	1,1	**0,000**	**0,002**	0,276	DNA-directed RNA polymerase subunit beta
SMU.1991	1,2	1,1	-1,1	0,063	0,484	0,270	putative membrane carboxypeptidase, penicillin-binding protein 1b
SMU.1992	-1,1	-1,2	1,2	0,306	**0,017**	**0,014**	tyrosyl-tRNA synthetase
SMU.1993	1,1	-1,1	-1,0	0,499	0,402	0,835	putative ABC transporter, zinc permease protein
SMU.1994	1,1	1,0	-1,4	0,331	0,948	**0,000**	putative ABC transporter, ATP-binding protein
SMU.1995c	1,0	1,1	-1,1	0,772	0,494	0,609	putative transcriptional regulator
SMU.1996	-1,0	1,6	-1,1	0,851	**0,000**	0,299	4-diphosphocytidyl-2-C-methyl-D-erythritol kinase
SMU.1997	1,0	**25,7**	-1,3	0,973	**0,000**	**0,038**	putative ComX1, transcriptional regulator of competence-specific genes
SMU.1999c	-1,0	-1,3	-1,2	0,767	**0,013**	0,117	hypothetical protein
SMU.2000	1,3	-1,1	1,6	0,053	0,325	**0,001**	50S ribosomal protein L17
SMU.2001	1,3	-1,2	1,6	**0,002**	**0,009**	**0,000**	DNA-directed RNA polymerase subunit alpha
SMU.2002	1,6	-1,1	**2,0**	**0,000**	0,477	**0,000**	30S ribosomal protein S11
SMU.2003	1,6	-1,1	1,7	**0,000**	0,231	**0,000**	30S ribosomal protein S13
SMU.2003a	1,6	-1,2	1,7	**0,002**	0,240	**0,000**	50S ribosomal protein L36
SMU.2004	1,5	-1,2	1,5	**0,012**	0,241	**0,018**	translation initiation factor IF-1
SMU.2005	1,0	-1,1	1,1	0,852	0,432	0,596	adenylate kinase
SMU.2006	1,2	-1,2	1,2	0,133	0,206	0,203	preprotein translocase subunit SecY
SMU.2007	1,2	-1,1	-1,0	0,375	0,552	0,877	50S ribosomal protein L15
SMU.2008	1,4	-1,1	1,1	**0,036**	0,637	0,213	50S ribosomal protein L30
SMU.2009	1,5	-1,0	1,2	**0,001**	0,729	0,111	30S ribosomal protein S5
SMU.2010	1,5	-1,0	1,5	0,052	0,933	0,081	50S ribosomal protein L18
SMU.2011	1,5	-1,0	1,2	**0,000**	0,692	0,103	50S ribosomal protein L6
SMU.2012	1,3	-1,2	1,3	**0,011**	0,176	**0,030**	30S ribosomal protein S8
SMU.2014	1,3	-1,1	1,4	0,147	0,567	0,079	30S ribosomal protein S14
SMU.2015	1,1	-1,3	1,5	0,272	**0,045**	**0,003**	50S ribosomal protein L5
SMU.2016	1,1	-1,3	1,3	0,430	**0,014**	0,114	50S ribosomal protein L24
SMU.2017	1,5	-1,1	1,5	**0,000**	0,422	**0,000**	50S ribosomal protein L14
SMU.2018	1,3	-1,2	1,2	**0,040**	0,241	0,163	30S ribosomal protein S17
SMU.2019	1,3	-1,1	-1,0	0,198	0,634	0,905	50S ribosomal protein L29
SMU.2020	1,6	-1,1	1,5	**0,000**	0,445	**0,001**	50S ribosomal protein L16
SMU.2021	1,5	-1,1	1,3	**0,000**	0,555	**0,038**	30S ribosomal protein S3
SMU.2022	1,1	-1,3	1,3	0,476	0,078	**0,047**	50S ribosomal protein L22
SMU.2023c	1,3	-1,1	1,4	**0,000**	0,051	**0,000**	30S ribosomal protein S19
SMU.2024c	1,5	-1,1	1,3	**0,002**	0,289	**0,003**	50S ribosomal protein L4
SMU.2025	1,5	-1,1	1,3	**0,001**	0,595	**0,038**	50S ribosomal protein L3
SMU.2026c	1,1	-1,1	1,2	0,670	0,722	0,391	30S ribosomal protein S10
SMU.2027	-1,2	1,2	1,1	0,126	0,157	0,357	putative transcriptional regulator
SMU.2028	1,1	-1,1	-1,0	0,340	0,061	0,917	levansucrase precursor; beta-D-fructosyltransferase
SMU.2029	1,1	-1,1	-1,2	**0,042**	0,336	**0,014**	class III stress response-related ATP-dependent Clp protease, ATP-binding subunit
SMU.2030	1,3	1,0	-1,2	**0,035**	0,819	0,075	putative transcriptional regulator CtsR
SMU.2031	1,2	-1,1	1,0	0,056	0,352	0,871	elongation factor Ts
SMU.2032	1,3	-1,1	1,1	**0,041**	0,642	0,391	30S ribosomal protein S2
SMU.2033c	-1,1	-1,3	1,2	0,115	**0,002**	**0,041**	hypothetical protein
SMU.2035	-1,1	-1,1	1,0	0,219	0,299	0,856	bacteriocin immunity protein

SMU.2036	1,2	-1,0	1,1	**0,010**	0,762	0,424	putative peptidase
SMU.2037	1,0	1,3	1,3	0,616	**0,000**	**0,002**	putative trehalose-6-phosphate hydrolase TreA
SMU.2038	-1,1	1,2	1,3	0,380	**0,003**	**0,000**	putative PTS system, trehalose-specific IIABC component
SMU.2040	-1,0	1,0	-1,0	0,947	0,684	0,781	putative transcriptional regulator; repressor of the trehalose operon
SMU.2042	1,2	-1,1	-1,1	**0,008**	**0,027**	0,221	dextranase precursor
SMU.2043c	1,1	-1,1	-1,2	0,372	0,132	**0,005**	D-tyrosyl-tRNA(Tyr) deacylase
SMU.2044	1,1	-1,2	-1,2	0,114	**0,003**	**0,003**	putative stringent response protein, ppGpp synthetase
SMU.2046c	-1,2	1,0	-1,2	**0,028**	0,803	**0,039**	hypothetical protein
SMU.2047	-1,2	1,0	1,1	**0,012**	0,746	0,095	putative PTS system, glucose-specific IIABC component
SMU.2048	1,0	1,0	-1,1	0,304	0,509	0,144	hypothetical protein
SMU.2049c	1,2	1,1	-1,1	**0,048**	0,438	0,074	16S ribosomal RNA methyltransferase RsmE
SMU.2050c	1,3	1,2	-1,2	**0,000**	**0,022**	**0,014**	ribosomal protein L11 methyltransferase
SMU.2052c	-1,2	1,0	-1,0	0,284	0,922	0,958	hypothetical protein
SMU.2053c	-1,2	-1,3	1,6	0,377	0,171	0,084	hypothetical protein
SMU.2054c	1,4	1,2	-1,2	**0,002**	0,143	0,160	hypothetical protein
SMU.2055	-1,0	-1,0	-1,1	0,865	0,721	0,442	putative acetyltransferase
SMU.2056	-1,0	1,1	1,0	0,741	0,160	0,482	recombination factor protein RarA
SMU.2057c	-1,2	1,3	-1,3	**0,024**	**0,002**	**0,001**	putative cadmium-transporting ATPase; P-type ATPase
SMU.2058	1,0	-1,1	1,2	0,668	0,181	**0,004**	putative transcriptional regulator
SMU.2059c	-1,1	-1,1	-1,1	0,167	0,129	0,536	putative integral membrane protein
SMU.2060	1,1	-1,0	1,1	0,193	0,949	0,125	LysR family transcriptional regulator
SMU.2061	1,1	-1,0	1,4	0,312	0,727	**0,000**	hypothetical protein
SMU.2063	-1,0	-1,0	1,0	0,694	0,629	0,784	ferrochelatase
SMU.2064c	-1,1	-1,1	-1,1	0,488	0,062	0,294	putative transmembrane protein
SMU.2065	1,1	-1,1	-1,2	0,358	0,428	0,141	putative UDP-glucose 4-epimerase
SMU.2066c	-1,0	-1,1	1,0	0,931	0,312	0,733	putative transmembrane protein
SMU.2067	1,1	-1,0	-1,1	0,142	0,621	0,261	putative stress response protein; glycosyltransferase involved in cell wall biogenesis
SMU.2069	-1,2	-1,1	1,0	**0,045**	0,207	0,745	zinc transporter ZupT
SMU.2070	-1,0	1,0	1,0	0,567	0,637	0,482	hypothetical protein
SMU.2071	1,1	-1,0	-1,3	0,544	0,983	**0,005**	putative anaerobic ribonucleotide reductase activating protein
SMU.2072c	1,2	-1,0	-1,3	**0,007**	0,834	**0,001**	acetyltransferase
SMU.2073c	-1,1	1,0	-1,5	0,209	0,900	**0,000**	hypothetical protein
SMU.2074	1,2	-1,0	1,0	**0,012**	0,551	0,976	anaerobic ribonucleoside triphosphate reductase
SMU.2075c	-1,1	1,8	1,1	0,281	**0,000**	0,564	hypothetical protein
SMU.2076c	1,0	**4,2**	-1,3	0,903	**0,001**	**0,034**	hypothetical protein
SMU.2077c	1,2	1,1	-1,1	0,281	0,746	0,738	hypothetical protein
SMU.2078c	1,5	1,3	-1,2	**0,032**	0,200	0,394	Holliday junction resolvase-like protein
SMU.2079c	1,4	1,3	-1,1	**0,035**	0,110	0,654	hypothetical protein
SMU.2080	-1,1	-1,1	1,1	**0,011**	**0,004**	**0,046**	hypothetical protein
SMU.2081	1,2	-1,0	1,1	**0,037**	0,929	0,545	hypothetical protein
SMU.2083c	1,4	1,8	1,0	0,238	**0,027**	0,987	hypothetical protein
SMU.2084c	-1,1	1,9	1,6	0,578	**0,000**	**0,017**	transcriptional regulator Spx
SMU.2085	1,5	**4,6**	1,3	**0,000**	**0,000**	**0,018**	recombinase A
SMU.2086	1,2	**8,0**	1,0	0,067	**0,000**	0,772	competence damage-inducible protein A
SMU.2087	1,2	-1,1	1,1	0,250	0,397	0,648	putative 3-methyl-adenine DNA glycosylase I
SMU.2088	1,1	-1,1	-1,0	0,450	0,344	0,664	Holliday junction DNA helicase RuvA
SMU.2089	1,0	-1,1	1,1	0,437	**0,035**	**0,031**	DNA mismatch repair protein
SMU.2090c	1,0	1,3	-1,2	0,956	0,478	0,530	hypothetical protein
SMU.2091c	1,1	1,1	-1,0	0,227	0,131	0,725	DNA mismatch repair protein MutS
SMU.2092c	1,1	1,1	-1,1	0,234	0,083	0,156	hypothetical protein
SMU.2093	1,2	1,0	1,1	0,266	0,991	0,590	putative transcriptional regulator of arginine metabolism
SMU.2094c	1,1	-1,1	1,2	0,600	0,595	**0,027**	hypothetical protein
SMU.2096c	1,1	-1,0	1,1	0,356	0,745	0,598	hypothetical protein
SMU.2097	1,0	-1,1	1,5	0,766	0,409	**0,001**	hypothetical protein
SMU.2098	1,1	-1,0	1,1	0,122	0,753	**0,046**	arginyl-tRNA synthetase
SMU.2099c	-1,1	1,0	-1,0	0,531	0,983	0,854	hypothetical protein
SMU.2100c	-1,1	-1,1	-1,0	0,691	0,421	0,908	hypothetical protein
SMU.2101	1,1	-1,0	-1,1	0,391	0,775	0,173	aspartyl-tRNA synthetase
SMU.2102	1,0	-1,1	-1,2	0,928	0,199	0,056	histidyl-tRNA synthetase
SMU.2104	-1,2	-1,1	-1,0	**0,002**	0,337	0,952	integral membrane protein
SMU.2104a	-1,3	-1,2	1,6	0,130	0,243	**0,002**	50S ribosomal protein L32
SMU.2105	-1,1	-1,1	-1,3	0,834	0,746	0,385	hypothetical protein
SMU.2106c	1,1	1,1	1,1	0,656	0,807	0,733	transcription regulator

SMU.2107c	-1,1	1,0	-1,2	0,634	0,885	0,250	hypothetical protein
SMU.2108c	-1,1	-1,1	1,0	0,114	0,169	0,829	putative transcriptional regulator
SMU.2109	-1,2	-1,2	-1,1	**0,019**	0,067	0,383	putative MDR permease; multidrug efflux pump
SMU.2111c	-1,1	1,1	-1,0	0,103	0,430	0,971	hypothetical protein
SMU.2112	1,2	-1,1	-1,1	**0,000**	0,148	0,060	glucan-binding protein A, GbpA
SMU.2113c	1,4	-1,1	1,2	**0,003**	0,198	0,131	hypothetical protein
SMU.2114c	1,0	-1,1	-1,0	0,821	0,295	0,760	putative transcriptional regulator
SMU.2115	-1,1	-1,0	1,1	**0,049**	0,661	0,124	putative short-chain dehydrogenase
SMU.2116	1,1	-1,1	-1,0	0,323	0,477	0,607	putative osmoprotectant amino acid ABC transporter, ATP-binding protein
SMU.2117	1,1	-1,0	-1,1	0,315	0,860	0,361	putative osmoprotectant ABC transporter; permease protein
SMU.2118	1,0	-1,1	1,1	0,859	0,352	0,357	putative ABC transporter; osmoprotectant-binding protein, glycine betaine/carnitine/choline ABC transporter
SMU.2119	1,3	-1,1	1,0	0,078	0,576	0,852	putative osmoprotectant ABC transporter; permease protein
SMU.2120c	1,2	1,1	-1,3	0,238	0,545	**0,030**	putative 3-methyladenine DNA glycosylase
SMU.2121c	1,1	-1,2	-1,2	0,672	0,446	0,326	hypothetical protein
SMU.2123	-1,2	-1,0	-1,2	**0,043**	0,691	**0,049**	hypothetical protein
SMU.2124	-1,2	-1,2	-1,1	0,483	0,297	0,520	hypothetical protein
SMU.2125	-1,3	-1,0	-1,0	0,203	0,891	0,767	hypothetical protein
SMU.2126c	-1,1	1,1	-1,2	0,431	0,375	0,311	putative purine-nucleoside phosphorylase
SMU.2127	-1,2	-1,0	-1,0	**0,004**	0,991	0,483	putative succinate semialdehyde dehydrogenase
SMU.2128	1,1	1,0	-1,1	0,060	0,703	0,215	dihydroxy-acid dehydratase
SMU.2129c	1,0	-1,1	-1,2	0,872	0,724	0,380	hypothetical protein
SMU.2130	-1,1	-1,1	1,1	0,324	0,109	**0,043**	hypothetical protein
SMU.2131	-1,1	-1,3	1,2	0,455	**0,040**	0,081	hypothetical protein
SMU.2133c	1,0	1,0	-1,1	0,461	0,938	**0,001**	hypothetical protein
SMU.2134	-1,1	1,1	-1,0	0,477	0,179	0,678	putative transcriptional regulator
SMU.2135c	1,1	-1,0	1,3	0,649	0,814	**0,016**	30S ribosomal protein S4
SMU.2136c	1,1	-1,2	1,0	0,892	0,756	0,974	hypothetical protein
SMU.2137c	-1,1	-1,2	1,1	0,717	0,414	0,595	hypothetical protein
SMU.2138	1,3	-1,0	1,1	**0,006**	0,586	0,557	replicative DNA helicase
SMU.2139c	1,0	-1,3	1,1	1,000	0,146	0,453	50S ribosomal protein L9
SMU.2140c	1,2	-1,1	-1,0	**0,015**	0,064	0,621	hypothetical protein
SMU.2141	1,2	-1,1	1,0	**0,013**	0,116	0,902	tRNA uridine 5-carboxymethylaminomethyl modification enzyme GidA
SMU.2142	1,2	-1,0	1,1	0,420	0,828	0,774	hypothetical protein
SMU.2143c	1,3	1,2	-1,1	**0,005**	0,093	0,527	tRNA-specific 2-thiouridylase MnmA
SMU.2146c	1,2	-1,2	1,1	0,251	0,260	0,664	hypothetical protein
SMU.2147c	1,4	-1,2	1,5	**0,006**	0,178	**0,002**	hypothetical protein
SMU.2148c	-1,0	-1,2	-1,0	0,969	0,217	0,824	cobalt permease
SMU.2149c	1,0	-1,2	-1,1	0,911	0,350	0,667	cobalt transporter ATP-binding subunit
SMU.2150c	1,1	-1,1	-1,0	0,343	0,536	0,879	cobalt transporter ATP-binding subunit
SMU.2151	-1,1	-1,3	1,2	0,560	0,280	0,453	putative phosphotidylglycerophosphate synthase
SMU.2152c	1,1	-1,2	1,2	0,644	0,057	0,057	hypothetical protein
SMU.2153c	1,0	-1,2	1,4	0,995	**0,024**	**0,001**	putative peptidase
SMU.2154c	1,0	-1,2	1,4	0,886	**0,016**	**0,000**	putative peptidase
SMU.2155	-1,0	-1,0	1,3	0,971	0,852	0,214	hypothetical protein
SMU.2156	-1,1	-1,0	1,4	0,440	0,995	**0,003**	recombination protein F
SMU.2157	1,1	-1,1	-1,1	0,260	0,616	0,320	inosine 5'-monophosphate dehydrogenase
SMU.2158c	1,0	-1,2	-1,1	0,730	0,109	0,523	tryptophanyl-tRNA synthetase II
SMU.2159	-1,0	-1,1	1,0	0,672	0,605	0,879	putative ABC transporter, ATP-binding protein
SMU.2160	-1,0	-1,0	1,1	0,885	0,583	0,216	transmembrane protein
SMU.2161c	1,1	-1,1	1,1	0,620	0,494	0,403	hypothetical protein
SMU.2162c	-1,1	-1,3	-1,3	0,606	0,171	0,187	rRNA large subunit methyltransferase
SMU.2164	-1,0	-1,2	1,4	0,968	0,197	**0,004**	serine protease HtrA
SMU.2165	-1,1	-1,1	1,5	0,223	0,384	**0,000**	putative SpoJ
