# Supplementary material for: Comprehensive Transcriptome Profiles of Streptococcus mutans UA159 Map Core Streptococcal Competence Genes
Source: mSystems. 2016 Apr 12;1(2):e00038-15. doi: 10.1128/mSystems.00038-15 (PMC5069739; doi:10.1128/mSystems.00038-15)
Supplement: Table S4 [file sys002162010st4.pdf]

Table S4. Comparison of transcriptome results among four studies. Mean fold changes induced by CSP in *S. mutans* UA159 (>2 or <-2) in transcriptome studies using long exposure to CSP in rich medium.

	Gene	Other names	100 min this study	Perry et al[a](1)	Lemme et al (GFP)(2)	Lemme et al (MIXED)(2)
			**Differentially expressed in the 4 transcriptomes**			
1	SMU.625	*comEA*	131.7	14.1	70.9	20.3
2	SMU.1982c	*comGE*	111.6	22.3	116.8	35.2
3	SMU.1987	*comGA*	106.0	17.5	164.4	44.8
4	SMU.1980c	*comGG*	102.3	24.6	112.0	37.5
5	SMU.836	*lytF*	74.8	18.3	34.4	9.9
6	SMU.1984	*comGC*	70.0	26.2	140.4	40.3
7	SMU.1985	*comGB*	69.8	23.9	91.4	30.6
8	SMU.1983	*comGD*	68.8	26.6	74.4	23.5
9	SMU.1981c		68.6	25.9	134.9	41.4
10	SMU.498	*comFA*	64.8	22.4	31.8	9.4
11	SMU.150	*nlmA*	62.4	11.2	5.7	2.4
12	SMU.626	*comEC*	61.7	25.6	55.2	18.6
13	SMU.1908c		57.1	18.3	6.4	6.4
14	SMU.1909c	*imm*	55.1	19.6	6.0	6.2
15	SMU.1967	*ssbB*	52.8	16.4	105.8	28.9
16	SMU.1904c		49.7	12.2	5.0	4.5
17	SMU.423	*nlmD*	49.5	14.6	7.2	6.7
18	SMU.1905c	*bsmL*	47.8	10.1	5.4	4.8
19	SMU.1001	*dprA*	45.0	19.0	45.2	12.7
20	SMU.151	*nlmB*	41.1	12.4	7.0	6.5
21	SMU.499	*comFC*	40.5	18.0	48.0	12.6
22	SMU.644	*coiA*	39.7	27.2	20.1	5.9
23	SMU.1910c		39.2	18.3	6.6	6.8
24	SMU.152		36.4	16.1	6.1	4.9
25	SMU.1906c	*bsmB*	34.0	11.4	6.4	6.6
26	SMU.1912c		29.9	22.2	6.1	6.1
27	SMU.1979c		29.8	22.0	44.9	16.3
28	SMU.1913c	*immA*	29.3	15.2	5.9	5.7
29	SMU.153		25.9	12.2	6.8	5.4
30	SMU.1997	*sigX*	25.7	14.3	13.8	3.6
31	SMU.1055	*radC*	23.0	14.1	20.8	6.4
32	SMU.769	*pilC*	18.2	9.5	36.0	12.2
33	SMU.1914c	*cipB*	17.4	20.4	8.2	8.6
34	SMU.1903c		16.0	16.0	5.7	4.9
35	SMU.505		14.4	11.8	16.6	6.0

36	SMU.925	*immB*	10.3	18.2	8.7	6.2
37	SMU.1917	*comE*	10.0	11.3	7.3	3.9
38	SMU.645	*pepB*	9.8	10.4	11.0	3.7
39	SMU.539c	*cilC*	8.7	22.4	8.7	2.8
40	SMU.2086	*cinA*	8.0	10.2	13.1	4.9
41	SMU.1978	*ack*	7.5	9.0	14.9	6.6
42	SMU.1002	*topA*	6.5	8.2	7.9	3.2
43	SMU.1916	*comD*	6.3	10.5	7.0	3.9
44	SMU.352		6.0	6.0	7.2	3.0
45	SMU.353		5.6	5.3	7.1	3.0
46	SMU.506		5.6	9.0	9.5	3.6
47	SMU.354	*ccs50*	5.4	6.2	7.7	3.2
48	SMU.772	*gbpD*	4.9	10.0	7.3	2.4
49	SMU.2085	*recA*	4.6	6.7	6.6	3.2
50	SMU.2076c		4.2	10.1	5.1	2.4
51	SMU.927		3.4	4.8	3.7	2.5
52	SMU.500		3.2	2.7	5.3	3.4
53	SMU.928		2.7	5.3	3.6	2.3
54	SMU.1400c		2.7	4.7	3.8	2.1
55	SMU.1902c	*bsmK*	2.5	9.8	3.1	2.8
**Differentially expressed in all, except in Lemme et al. mixed population**						
1	SMU.424		5.9	8.4	2.3	
2	SMU.64		5.6	8.0	3.7	
3	SMU.426		5.5	7.3	2.3	
4	SMU.508		4.9	7.2	2.7	
5	SMU.507		4.9	6.0	3.1	
6	SMU.65		4.4	6.6	3.4	
7	SMU.427		4.0	6.5	2.3	
8	SMU.1966c		3.7	7.1	4.4	
9	SMU.166		3.7	9.1	2.3	
10	SMU.168		3.5	8.7	2.6	
11	SMU.627		3.3	4.5	3.6	
12	SMU.356		3.2	3.8	2.5	
13	SMU.167		2.6	10.0	2.1	
14	SMU.66		2.6	5.0	2.1	
15	SMU.838		2.4	6.5	2.2	
16	SMU.1965c		2.4	5.5	3.5	
**Differentially expressed in the present study and in Perry et al, but not in Lemme et al.**						
1	SMU.837		32.0	14.1		
2	SMU.646		10.3	9.5		
3	SMU.355		5.9	6.8		

4	SMU.1003			5.8	7.1	
5	SMU.926			3.7	5.4	
6	SMU.326			3.3	3.3	
7	SMU.327			3.1	3.2	
8	SMU.325			3.0	4.1	
9	SMU.67			2.3	4.0	
10	SMU.68			2.3	2.9	
11	SMU.109			2.2	5.7	
12	SMU.1345c			-2.1	-2.7	
13	SMU.1346			-2.3	-2.5	
14	SMU.63c			-2.3	5.3	
**Differentially expressed only in our study**						
1	SMU.61a	*comS*	12.6	no probe	no probe	no probe
2	SMU.285		-2.1			
3	SMU.1317c		-2.1			
**Differentially expressed only in Perry et al.**						
1	SMU.1907			8.9		
2	SMU.771c			8.0		
3	SMU.2081			7.5		
4	SMU.2037			6.7		
5	SMU.1053			6.4		
6	SMU.1047c			6.2		
7	SMU.1054			6.0		
8	SMU.1004			6.0		
9	SMU.1900			5.9		
10	SMU.1056			5.7		
11	SMU.2038			5.3		
12	SMU.1899			5.2		
13	SMU.1147c			5.0		
14	SMU.2027			4.7		
15	SMU.207c			4.4		
16	SMU.531			4.3		
17	SMU.1898			4.2		
18	SMU.149			4.1		
19	SMU.2083c			4.0		
20	SMU.212c			3.8		
21	SMU.1915			3.8		
22	SMU.1977c			3.8		
23	SMU.209c			3.8		

24	SMU.535	3.7
25	SMU.1069c	3.7
26	SMU.205c	3.5
27	SMU.148	3.4
28	SMU.532	3.4
29	SMU.534	3.4
30	SMU.758c	3.3
31	SMU.1996	3.3
32	SMU.2080	3.3
33	SMU.195c	3.3
34	SMU.208c	3.3
35	SMU.215c	3.2
36	SMU.210c	3.2
37	SMU.636	3.1
38	SMU.61	3.1
39	SMU.1327c	3.0
40	SMU.196c	3.0
41	SMU.1068c	3.0
42	SMU.204c	3.0
43	SMU.637c	2.9
44	SMU.193c	2.9
45	SMU.1424	2.9
46	SMU.2075c	2.9
47	SMU.1006	2.9
48	SMU.199c	2.8
49	SMU.400	2.8
50	SMU.2079c	2.8
51	SMU.538	2.8
52	SMU.200c	2.8
53	SMU.202c	2.8
54	SMU.1067c	2.7
55	SMU.2077c	2.7
56	SMU.41	2.7
57	SMU.1197	2.6
58	SMU.198c	2.6
59	SMU.1975c	2.5
60	SMU.401c	2.5
61	SMU.378	2.5
62	SMU.536	2.5
63	SMU.1044c	2.5
64	SMU.216c	2.5
65	SMU.537	2.5
66	SMU.1193	2.5

67	SMU.217c	2.5
68	SMU.399	2.5
69	SMU.1048	2.4
70	SMU.632	2.4
71	SMU.470	2.4
72	SMU.2078c	2.4
73	SMU.1145c	2.4
74	SMU.1409c	2.4
75	SMU.1677	2.4
76	SMU.1185	2.4
77	SMU.1651	2.4
78	SMU.1046c	2.4
79	SMU.1862	2.4
80	SMU.1196c	2.3
81	SMU.954	2.3
82	SMU.1509	2.2
83	SMU.214c	2.2
84	SMU.1372c	2.2
85	SMU.154	2.2
86	SMU.328	2.2
87	SMU.1194	2.2
88	SMU.1034c	2.2
89	SMU.735	2.1
90	SMU.1438c	2.1
91	SMU.862	2.1
92	SMU.807	2.1
93	SMU.1848	2.1
94	SMU.840c	2.1
95	SMU.641	2.1
96	SMU.1267c	-2.1
97	SMU.1271	-2.1
98	SMU.2016	-2.1
99	SMU.2012	-2.1
100	SMU.2002	-2.1
101	SMU.1266	-2.1
102	SMU.872	-2.1
103	SMU.1886	-2.1
104	SMU.2017	-2.1
105	SMU.558	-2.1
106	SMU.503c	-2.2
107	SMU.127	-2.2
108	SMU.1268	-2.2
109	SMU.629	-2.2

110	SMU.402	-2.2
111	SMU.959c	-2.2
112	SMU.2001	-2.2
113	SMU.2022	-2.3
114	SMU.1512	-2.3
115	SMU.1270	-2.3
116	SMU.2000	-2.3
117	SMU.1341c	-2.4
118	SMU.131	-2.4
119	SMU.48	-2.4
120	SMU.1273	-2.4
121	SMU.72	-2.4
122	SMU.128	-2.5
123	SMU.2015	-2.5
124	SMU.610	-2.5
125	SMU.50	-2.6
126	SMU.887	-2.6
127	SMU.886	-2.6
128	SMU.129	-2.6
129	SMU.30	-2.6
130	SMU.888	-2.7
131	SMU.73	-2.7
132	SMU.130	-2.7
133	SMU.1342	-2.9
134	SMU.49	-2.9
135	SMU.1877	-2.9
136	SMU.1344c	-2.9
137	SMU.1878	-3.0
138	SMU.58	-3.1
139	SMU.56	-3.1
140	SMU.1961c	-3.2
141	SMU.1073	-3.3
142	SMU.877	-3.3
143	SMU.1960c	-3.4
144	SMU.51	-3.4
145	SMU.1343c	-3.4
146	SMU.53	-3.5
147	SMU.54	-3.5
148	SMU.55	-3.6
149	SMU.52	-3.6
150	SMU.1879	-3.9
151	SMU.1958c	-4.4
152	SMU.878	-4.4

153	SMU.79	-4.5	
154	SMU.1956c	-5.0	
155	SMU.1957	-5.1	
156	SMU.879	-5.4	
157	SMU.78	-5.5	
158	SMU.883	-5.6	
159	SMU.880	-5.6	
160	SMU.881	-5.8	
161	SMU.882	-5.9	
**Differentially expressed only in Lemme et al mixed population**			
	None		
**Differentially expressed only in Lemme et al competent population (sorted GFP-sigX population)[b]**			
1	SMU.93c		-2.5
2	SMU.658		-2.5
3	SMU.656		-2.5
4	SMU.566c		-2.5
5	SMU.436c		-2.5
6	SMU.262		-2.5
7	SMU.1596		-2.5
8	SMU.1259		-2.5
9	SMU.1150		-2.5
10	SMU.657		-3.3
11	SMU.565c		-3.3
12	SMU.1411		-3.3
13	SMU.750c		-5.0
14	SMU.1599		-5.0
15	SMU.1597c		-5.0
16	SMU.1031		-5.0
17	SMU.1029		-10.0
**Differentially expressed in at least 2 of the previous studies, but not in the present study**			
1	SMU.2084c	5.6	2.8
2	SMU.1897	9.2	2.7
3	SMU.1964c	4.8	2.6
4	SMU.647	2.7	2.6
5	SMU.108	4.9	2.5
6	SMU.1963c	4.0	2.5
7	SMU.1070c	3.4	2.3
8	SMU.1976c	3.6	2.2
9	SMU.753	4.0	2.1
10	SMU.2057c	4.0	2.1

11	SMU.80	3.0	2.1
12	SMU.655	3.9	-3.3
13	SMU.1148	2.4	-3.3

[a] No information on the direction of the transcripts.

[b] All genes in this list were also down-regulated in the gfp-sigX negative population (not included in this table) that was separated from the positive population by cell-sorting, indicating that these effects were not related to competence.

1. **Perry, J. A., M. B. Jones, S. N. Peterson, D. G. Cvitkovitch, and C. M. Levesque.** 2009. Peptide alarmone signalling triggers an auto-active bacteriocin necessary for genetic competence. Mol Microbiol **72:**905-17.
2. **Lemme, A., L. Grobe, M. Reck, J. Tomasch, and I. Wagner-Dobler.** 2011. Subpopulation-Specific Transcriptome Analysis of Competence-Stimulating-Peptide-Induced *Streptococcus mutans*. J Bacteriol **193:**1863-77.
