# Supplementary material for: Comprehensive Transcriptome Profiles of Streptococcus mutans UA159 Map Core Streptococcal Competence Genes
Source: mSystems. 2016 Apr 12;1(2):e00038-15. doi: 10.1128/mSystems.00038-15 (PMC5069739; doi:10.1128/mSystems.00038-15)
Supplement: Figure S1 [file sys002162010sf8.pdf]

Figure S1. Correlations between gene expression changes induced by CSP in our study and in previous transcriptome studies. Induced expression is shown as log_2 ratios for all annotated ORF sequences in the *S. mutans* UA159 genome (Accession AE014133) with expression ratios induced by > than 2 fold in one or both of the axis. **(A)** Correlation of ratios between short (10 min) and long (100 min) CSP exposure of *S. mutans* UA159 in our study, showing a similar distinction pattern between EARLY and LATE genes as in the scatter plot in Fig. 4D (*S. mutans* UA159 100 min vs ΔcomS 100 min) ; **(B)** Correlation of ratios between long CSP exposure of *S. mutans* UA159 in our study and in Perry et al (1). [a] Induced and [b] repressed genes in response to CSP that were exclusive for Perry et al. **(D)** Correlation of ratios between long CSP exposure of *S. mutans* UA159 in our study and in the sorted population of competent *S. mutans* used by Lemme et al (2); [c] Genes exclusively induced in Lemme et al., or [d] in our study (changes that were not significant in Lemme et al received an arbitrary value of 0) **(C)** Correlation of ratios between long CSP exposure of *S. mutans* UA159 in our study and in the mixed population used by Lemme et al (2); (changes that were not significant in Lemme et al received an arbitrary value of 0). EARLY genes are defined as those induced by short CSP exposure in the UA159 wild type (red borders), and LATE genes as those that showed induction only by long CSP exposure in the wild type (green borders for putative genes of the ComR regulon, and blue borders for the remaining genes). Circles corresponding to genes encoding upregulated bacteriocins are filled in red, bacteriocin immunity protens in yellow, proteins involved in DNA uptake in light blue, and in DNA recombination in green. The points corresponding to *comE*, *comD*, *comS*, and *sigX*, are indicated by name (D; dark blue). Light gray filled circles correspond to other genes. Note that the transcriptomes by Perry et al. (1) used traditional microarrays with probes limited to annotated ORFs, with no discrimination of the transcription direction. Lemme et al. (2) used a tiling microarray analysed for transcription in the sense direction. Neither of the two previous microarrays had probes for *comS*.

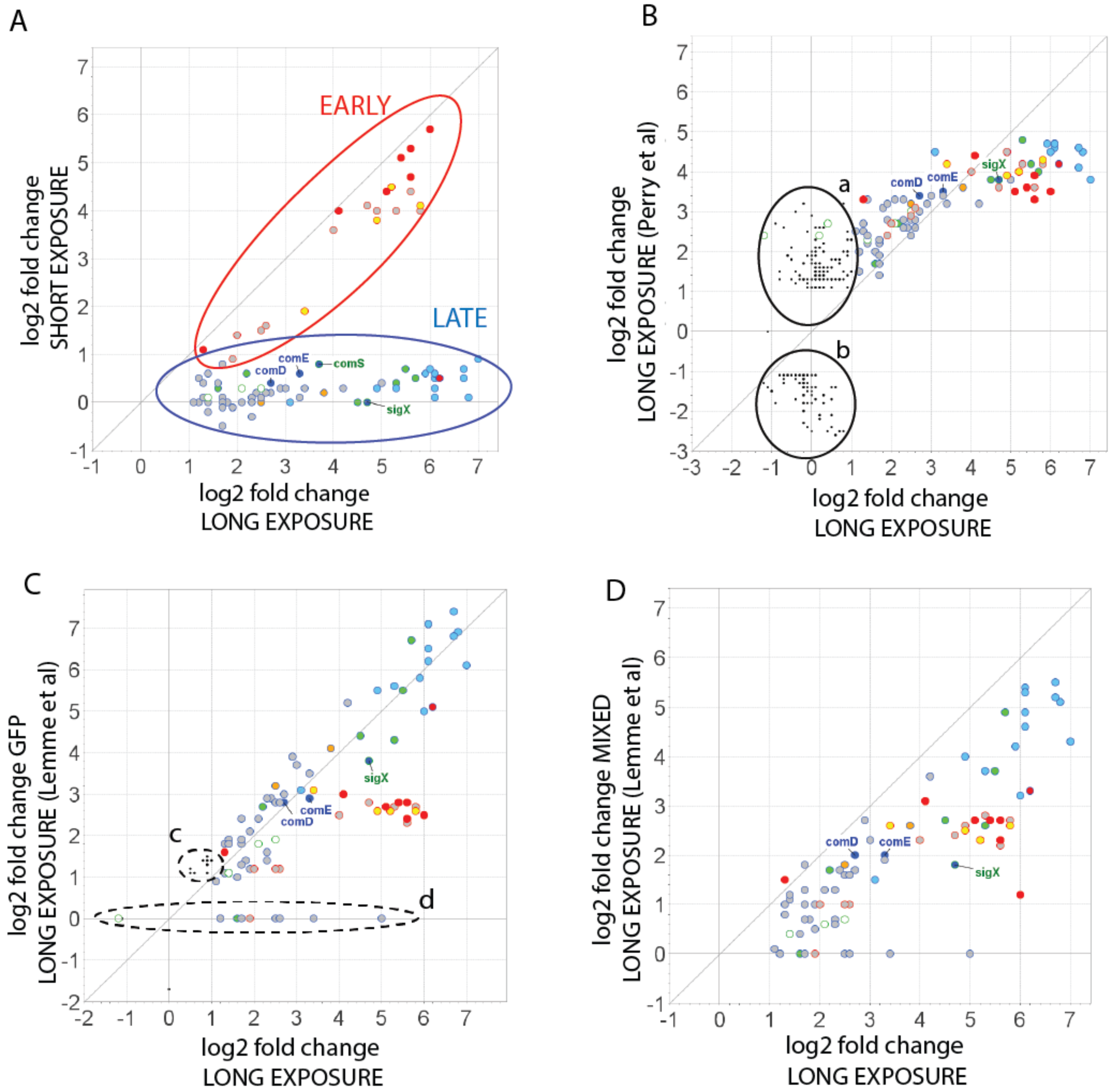
A
EARLY
LATE
comE
comD
comS
sigX
log2 fold change
SHORT EXPOSURE
log2 fold change
LONG EXPOSURE
B
a
b
comD
comE
sigX
log2 fold change
LONG EXPOSURE (Perry et al)
log2 fold change
LONG EXPOSURE
C
c
d
sigX
comE
comD
log2 fold change GFP
LONG EXPOSURE (Lemme et al)
log2 fold change
LONG EXPOSURE
D
comD
comE
sigX
log2 fold change MIXED
LONG EXPOSURE (Lemme et al)
log2 fold change
LONG EXPOSURE

References:

1. **Perry, J. A., M. B. Jones, S. N. Peterson, D. G. Cvitkovitch, and C. M. Levesque.** 2009. Peptide alarmone signalling triggers an auto-active bacteriocin necessary for genetic competence. Molecular Microbiology **72:**905-17.
2. **Lemme, A., L. Grobe, M. Reck, J. Tomasch, and I. Wagner-Dobler.** 2011. Subpopulation-specific transcriptome analysis of competence stimulating peptide induced *Streptococcus mutans*. Journal of Bacteriology **193:**1863-77.
