# Supplementary material for: Comprehensive Transcriptome Profiles of Streptococcus mutans UA159 Map Core Streptococcal Competence Genes
Source: mSystems. 2016 Apr 12;1(2):e00038-15. doi: 10.1128/mSystems.00038-15 (PMC5069739; doi:10.1128/mSystems.00038-15)
Supplement: Table S5 [file sys002162010st5.pdf]

Table S5. Effect of CSP on expression of inter-genic sequences. Mean fold change and P value for all inter-genic sequences.

IGS	Mean fold change[a]			p value					
	UA159 10 min	UA159 100 min	Δ*comS* 100 min	UA159 10 min	UA159 100 min	Δ*comS* 100 min	ORF left	ORF right	Direction
igs0002	-1,3	1,7	1,2	0,014	0,000	0,029	SMU,01	SMU,02	fw
igs0002	-1,1	-1,3	1,9	0,622	0,193	0,034	SMU,01	SMU,02	rc
igs0003	-1,2	-1,3	-1,4	0,008	0,018	0,014	SMU,02	SMU,05	fw
igs0003	-1,4	-1,3	1,4	0,042	0,166	0,124	SMU,02	SMU,05	rc
igs0004	-1,4	1,1	-1,4	0,000	0,359	0,000	SMU,05	SMU,06	fw
igs0004	-1,1	-1,1	-1,1	0,567	0,518	0,743	SMU,05	SMU,06	rc
igs0005	1,3	-1,1	1,3	0,005	0,160	0,012	SMU,06	SMU,07	fw
igs0005	1,0	-1,4	-1,9	0,958	0,177	0,002	SMU,06	SMU,07	rc
igs0007	1,2	-1,2	1,4	0,153	0,136	0,226	SMU,08	SMU,09	fw
igs0007	-1,1	-1,1	1,3	0,690	0,698	0,163	SMU,08	SMU,09	rc
igs0008	-1,1	-1,2	-1,1	0,150	0,259	0,649	SMU,11	SMU,12	fw
igs0008	-1,3	-1,6	1,0	0,617	0,156	0,916	SMU,11	SMU,12	rc
igs0011	-1,1	1,1	1,3	0,301	0,190	**0,000**	SMU,15	SMU,16	fw
igs0011	1,0	1,1	1,3	0,970	0,796	0,511	SMU,15	SMU,16	rc
igs0012	1,1	-1,4	1,1	0,434	**0,020**	0,615	SMU,16	SMU,18	fw
igs0012	-1,4	-1,4	-1,3	0,137	0,110	0,081	SMU,16	SMU,18	rc
igs0013	1,1	-1,1	1,8	**0,003**	0,366	**0,000**	SMU,18	SMUr01	fw
igs0013	**2,1**	1,1	1,8	**0,000**	0,263	**0,000**	SMU,18	SMUr01	rc
igs0014	1,2	1,3	1,1	**0,022**	0,076	0,300	SMUr01	SMUt01	fw
igs0014	**2,3**	1,1	1,6	**0,000**	**0,031**	**0,000**	SMUr01	SMUt01	rc
igs0015	1,0	-1,1	1,5	0,764	0,532	**0,001**	SMUt01	SMUr02	fw
igs0015	1,5	-1,1	1,5	**0,000**	0,100	**0,000**	SMUt01	SMUr02	rc
igs0016	1,0	1,1	-1,1	0,849	0,294	0,126	SMUr02	SMUr03	fw
igs0016	**2,1**	1,1	1,6	**0,000**	0,347	**0,000**	SMUr02	SMUr03	rc
igs0017	1,1	-1,1	-1,2	**0,034**	**0,036**	**0,029**	SMUt02	SMUt03	fw
igs0017	**2,0**	-1,0	1,9	**0,000**	0,710	**0,000**	SMUt02	SMUt03	rc
igs0018	1,0	-1,2	-1,1	0,707	**0,010**	**0,006**	SMUt03	SMUt04	fw
igs0018	**2,3**	1,1	1,3	**0,000**	**0,037**	**0,000**	SMUt03	SMUt04	rc
igs0024	1,2	-1,2	1,3	**0,000**	**0,000**	**0,000**	SMUt09	SMUt10	fw
igs0024	**2,3**	1,1	1,4	**0,000**	0,094	**0,011**	SMUt09	SMUt10	rc
igs0031	1,1	-1,1	1,0	**0,002**	0,296	0,502	SMUt16	SMUt17	fw
igs0031	1,7	1,0	1,8	**0,001**	0,922	**0,000**	SMUt16	SMUt17	rc
igs0033	-1,1	1,3	1,1	0,748	0,332	0,683	SMUt18	SMU,20	fw
igs0033	1,7	1,2	-1,4	**0,010**	0,357	0,379	SMUt18	SMU,20	rc
igs0035	1,7	-1,6	1,6	**0,021**	**0,009**	**0,004**	SMU,21	SMU,22	fw
igs0035	1,5	-1,3	-1,5	0,403	0,573	0,294	SMU,21	SMU,22	rc
igs0036	1,1	1,4	-1,0	0,590	**0,015**	0,667	SMU,22	SMU,23	fw
igs0036	**2,1**	1,3	-2,2	0,124	0,594	0,145	SMU,22	SMU,23	rc
igs0037	-1,2	-1,1	1,5	**0,008**	0,186	**0,002**	SMU,23	SMU,24	fw
igs0037	-1,2	1,3	1,2	0,687	0,562	0,696	SMU,23	SMU,24	rc
igs0038	-1,0	-1,1	-1,3	0,694	0,547	0,188	SMU,25	SMU,26	fw
igs0038	-1,5	1,1	1,1	0,316	0,888	0,840	SMU,25	SMU,26	rc
igs0040	-1,1	-1,1	1,1	**0,028**	0,117	0,398	SMU,27	SMU,28	fw
igs0040	1,1	-1,2	1,1	0,840	0,394	0,702	SMU,27	SMU,28	rc
igs0041	-1,0	1,1	-1,6	0,494	0,261	**0,000**	SMU,28	SMU,29	fw
igs0041	1,4	-2,1	-1,2	**0,035**	**0,000**	0,448	SMU,28	SMU,29	rc
igs0045	-1,1	-1,0	1,1	0,105	0,984	0,434	SMU,32	SMU,33	fw
igs0045	1,1	1,1	-1,5	0,410	0,756	**0,024**	SMU,32	SMU,33	rc
igs0046	-1,3	-1,5	-1,0	**0,001**	**0,000**	0,794	SMU,33	SMU,34	fw
igs0046	-1,4	-1,1	-1,1	0,082	0,484	0,343	SMU,33	SMU,34	rc
igs0047	1,0	1,3	1,3	0,653	**0,000**	**0,000**	SMU,35	SMU,36	fw
igs0047	1,9	1,6	-1,7	**0,001**	**0,000**	**0,001**	SMU,35	SMU,36	rc

igs0048	-1,1	1,3	-1,4	0,066	**0,028**	**0,039**	SMU,36	SMU,37	fw
igs0048	1,1	-1,0	1,0	**0,021**	0,529	0,559	SMU,36	SMU,37	rc
igs0049	1,2	1,1	1,0	0,109	0,245	0,948	SMU,37	SMU,38c	fw
igs0049	1,2	1,4	-1,6	0,751	0,306	0,210	SMU,37	SMU,38c	rc
igs0050	1,1	1,0	1,0	0,510	0,980	0,944	SMU,38c	SMU,39	fw
igs0050	1,0	-1,0	1,2	0,656	0,795	0,067	SMU,38c	SMU,39	rc
igs0051	1,1	-1,5	-1,2	0,180	**0,029**	**0,003**	SMU,39	SMU,40	fw
igs0051	-1,6	-1,3	-1,1	0,061	0,329	0,856	SMU,39	SMU,40	rc
igs0052	1,0	1,4	-1,4	0,995	0,124	**0,000**	SMU,41	SMU,42	fw
igs0052	-1,7	-1,5	-1,0	0,059	0,183	0,928	SMU,41	SMU,42	rc
igs0053	-1,1	1,3	-1,7	0,170	0,108	**0,000**	SMU,42	SMU,43	fw
igs0053	-1,1	-1,8	-1,5	0,854	0,438	0,468	SMU,42	SMU,43	rc
igs0055	-1,3	1,3	1,9	**0,001**	**0,011**	**0,000**	SMU,44	SMU,45	fw
igs0055	-1,1	-1,4	1,1	0,724	0,074	0,670	SMU,44	SMU,45	rc
igs0057	1,1	-1,1	-1,6	0,450	0,488	**0,005**	SMU,47	SMU,48	fw
igs0057	1,1	-1,2	1,1	0,697	0,519	0,629	SMU,47	SMU,48	rc
igs0058	-1,1	1,3	1,4	0,132	**0,012**	**0,000**	SMU,48	SMU,49	fw
igs0058	-1,5	-1,4	-1,1	**0,006**	**0,003**	0,199	SMU,48	SMU,49	rc
igs0059	-1,1	1,2	-1,1	0,374	**0,013**	0,057	SMU,49	SMU,50	fw
igs0059	1,1	-1,0	-1,1	0,747	0,999	0,790	SMU,49	SMU,50	rc
igs0062	-1,0	1,0	1,4	0,948	0,756	**0,030**	SMU,54	SMU,55	fw
igs0062	2,0	1,1	-1,1	**0,000**	0,490	0,495	SMU,54	SMU,55	rc
igs0064	-1,4	-1,0	-1,2	**0,006**	0,592	**0,002**	SMU,56	SMU,58	fw
igs0064	1,2	1,0	1,2	0,297	0,697	0,444	SMU,56	SMU,58	rc
igs0065	-1,1	-1,1	1,1	0,121	0,363	0,570	SMU,58	SMU,59	fw
igs0065	-1,2	-1,1	1,0	0,363	0,583	0,700	SMU,58	SMU,59	rc
igs0067	-1,2	1,0	-1,1	**0,005**	0,744	0,337	SMU,60	SMU,61	fw
igs0067	1,1	**2,7**	-1,1	0,430	**0,000**	0,524	SMU,60	SMU,61	rc
igs0068	1,0	-1,4	1,2	0,977	**0,016**	0,217	SMU,61	SMU,63c	fw
igs0068 comS	-1,1	**15,5**	-1,0	0,620	**0,000**	0,826	SMU,61	SMU,63c	rc
igs0069	-1,0	-1,4	1,1	0,814	0,159	0,691	SMU,63c	SMU,64	fw
igs0069	1,5	**9,0**	-1,0	**0,048**	**0,000**	0,812	SMU,63c	SMU,64	rc
igs0070	1,2	1,0	-1,3	0,114	0,852	0,162	SMU,64	SMU,65	fw
igs0070	-1,6	**3,5**	-2,1	0,060	**0,008**	**0,045**	SMU,64	SMU,65	rc
igs0071	-1,2	-1,8	-1,4	**0,021**	**0,003**	**0,020**	SMU,65	SMU,66	fw
igs0071	1,2	**4,1**	-1,8	0,713	**0,001**	0,107	SMU,65	SMU,66	rc
igs0072	1,1	-1,1	1,2	0,239	0,333	**0,032**	SMU,67	SMU,68	fw
igs0072	1,0	**2,1**	-1,4	0,934	**0,036**	0,403	SMU,67	SMU,68	rc
igs0073	1,1	1,1	1,1	**0,007**	**0,003**	0,237	SMU,68	SMU,70	fw
igs0073	1,1	1,4	-1,2	0,840	0,326	0,563	SMU,68	SMU,70	rc
igs0074	1,1	1,2	-1,2	**0,028**	**0,001**	**0,001**	SMU,70	SMU,71	fw
igs0074	-1,0	-1,1	-1,5	0,866	0,620	**0,004**	SMU,70	SMU,71	rc
igs0075	-1,0	-1,1	-1,2	0,519	0,289	**0,003**	SMU,71	SMU,72	fw
igs0075	-1,3	-1,1	-1,4	0,241	0,670	0,134	SMU,71	SMU,72	rc
igs0077	1,2	1,1	-1,0	**0,006**	0,297	0,983	SMU,73	SMU,74	fw
igs0077	-1,2	1,2	-1,4	0,390	0,481	0,213	SMU,73	SMU,74	rc
igs0079	1,1	-1,1	-1,1	0,310	0,077	0,411	SMU,76	SMU,78	fw
igs0079	-1,0	-1,0	-1,2	0,663	0,803	0,082	SMU,76	SMU,78	rc
igs0080	-1,0	1,1	-1,0	0,947	0,081	0,993	SMU,78	SMU,79	fw
igs0080	-1,8	-1,5	1,6	**0,002**	0,092	0,164	SMU,78	SMU,79	rc
igs0081	1,0	1,1	-1,1	0,953	0,472	0,653	SMU,79	SMU,80	fw
igs0081	1,0	1,2	-1,1	0,997	0,816	0,912	SMU,79	SMU,80	rc
igs0082	-1,3	-1,2	1,1	0,137	0,262	0,710	SMU,80	SMU,81	fw
igs0082	-1,1	1,3	-2,2	**0,000**	**0,000**	**0,000**	SMU,80	SMU,81	rc
igs0083	-1,1	-1,2	-1,0	0,239	0,082	0,828	SMU,81	SMU,82	fw
igs0083	-1,1	1,1	-1,0	0,634	0,572	0,929	SMU,81	SMU,82	rc
igs0084	1,0	1,0	1,1	0,187	0,785	0,250	SMU,82	SMU,83	fw
igs0084	-1,0	1,3	1,1	0,733	**0,036**	0,409	SMU,82	SMU,83	rc
igs0085	-1,0	1,1	1,0	0,193	0,114	0,533	SMU,83	SMU,84	fw
igs0085	1,1	1,3	-1,2	0,818	0,448	0,632	SMU,83	SMU,84	rc
igs0086	-1,1	-1,1	1,3	0,170	0,081	**0,001**	SMU,86	SMU,87	fw
igs0086	1,1	-1,3	-1,3	0,210	**0,000**	**0,002**	SMU,86	SMU,87	rc
igs0087	-1,1	-1,1	1,2	0,627	0,604	0,615	SMU,87	SMU,88c	fw
igs0087	-1,2	-1,4	-1,2	0,282	**0,031**	0,262	SMU,87	SMU,88c	rc
igs0088	-1,4	1,2	-1,0	**0,000**	0,175	0,975	SMU,88c	SMU,89c	fw

igs0088	1,1	1,3	-1,4	0,064	**0,000**	**0,008**	SMU,88c	SMU,89c	rc
igs0089	1,3	1,6	1,2	**0,000**	**0,000**	**0,002**	SMU,89c	SMU,91	fw
igs0089	-1,0	-1,3	1,1	0,843	0,216	0,772	SMU,89c	SMU,91	rc
igs0090	-1,2	-1,0	-1,1	0,330	0,914	0,190	SMU,91	SMU,92c	fw
igs0090	1,3	1,0	-1,3	0,381	0,912	0,157	SMU,91	SMU,92c	rc
igs0091	-1,2	1,3	-1,2	0,104	**0,000**	0,055	SMU,92c	SMU,93c	fw
igs0091	1,0	1,2	1,1	0,803	**0,003**	0,255	SMU,92c	SMU,93c	rc
igs0092	-1,2	1,3	1,1	**0,047**	**0,013**	0,322	SMU,93c	SMU,94c	fw
igs0092	-1,1	1,0	1,0	0,100	0,601	0,657	SMU,93c	SMU,94c	rc
igs0093	-1,2	-1,2	1,3	0,487	0,391	0,225	SMU,94c	SMU,96	fw
igs0093	1,0	1,1	-1,1	0,843	0,651	0,644	SMU,94c	SMU,96	rc
igs0094	-1,2	1,0	1,6	**0,015**	0,873	**0,000**	SMU,96	SMU,97	fw
igs0094	-1,0	-1,8	1,0	0,925	**0,002**	0,935	SMU,96	SMU,97	rc
igs0095	-1,1	-1,3	-1,1	0,536	0,369	0,850	SMU,97	SMUt19	fw
igs0095	1,0	-1,2	-1,1	0,778	0,281	0,523	SMU,97	SMUt19	rc
igs0096	-1,2	-1,0	-1,1	**0,000**	0,803	**0,025**	SMUt19	SMU,99	fw
igs0096	1,1	1,0	-1,4	0,710	0,981	0,443	SMUt19	SMU,99	rc
igs0097	1,1	-1,0	-1,6	0,257	0,810	**0,000**	SMU,99	SMU,100	fw
igs0097	-1,2	-1,2	-1,1	0,628	0,586	0,733	SMU,99	SMU,100	rc
igs0099	1,2	-1,2	-1,2	0,642	0,726	0,630	SMU,105	SMU,106c	fw
igs0099	-1,2	1,2	1,0	0,128	0,313	0,750	SMU,105	SMU,106c	rc
igs0100	1,0	-1,3	1,3	0,859	0,300	0,345	SMU,106c	SMUt20	fw
igs0100	-1,1	1,0	1,0	0,421	0,881	0,810	SMU,106c	SMUt20	rc
igs0101	-1,0	-1,1	1,0	0,399	0,202	0,918	SMUt20	SMU,107	fw
igs0101	-1,2	1,1	1,2	0,438	0,720	0,484	SMUt20	SMU,107	rc
igs0103	1,0	-1,1	1,6	0,616	0,273	**0,000**	SMU,108	SMU,109	fw
igs0103	1,2	1,9	1,0	0,374	**0,033**	1,000	SMU,108	SMU,109	rc
igs0104	-1,0	-1,0	1,2	0,672	0,841	0,058	SMU,109	SMU,110	fw
igs0104	1,0	1,4	-1,0	0,891	**0,014**	0,825	SMU,109	SMU,110	rc
igs0105	-1,1	-1,0	1,2	0,520	0,635	0,091	SMU,110	SMU,112c	fw
igs0105	-1,1	1,0	-1,2	0,450	0,997	**0,042**	SMU,110	SMU,112c	rc
igs0106	-1,7	1,0	1,4	**0,005**	0,900	**0,001**	SMU,112c	SMU,113	fw
igs0106	1,2	-1,5	1,4	0,208	**0,043**	0,176	SMU,112c	SMU,113	rc
igs0110	1,0	1,5	1,5	0,268	**0,001**	**0,000**	SMU,116	SMU,117c	fw
igs0110	-1,7	**2,1**	1,9	**0,000**	**0,000**	**0,000**	SMU,116	SMU,117c	rc
igs0111	-1,3	-1,0	1,1	0,078	0,954	0,477	SMU,117c	SMU,118c	fw
igs0111	-1,1	-1,2	1,3	**0,021**	**0,003**	**0,000**	SMU,117c	SMU,118c	rc
igs0113	-1,0	1,1	-1,2	0,859	0,769	0,517	SMU,119	SMU,120	fw
igs0113	1,0	-1,0	1,3	0,937	0,884	0,395	SMU,119	SMU,120	rc
igs0114	-1,2	1,0	-1,0	**0,016**	0,706	0,764	SMU,120	SMU,121	fw
igs0114	1,6	-1,3	**2,7**	0,704	0,824	0,402	SMU,120	SMU,121	rc
igs0115	1,1	1,1	1,2	0,363	0,647	0,222	SMU,121	SMU,123	fw
igs0115	-1,1	-1,3	1,3	0,801	0,309	0,180	SMU,121	SMU,123	rc
igs0116	-1,0	1,1	-1,2	0,798	0,588	0,227	SMU,123	SMU,124	fw
igs0116	1,4	1,3	-1,3	0,121	0,199	0,183	SMU,123	SMU,124	rc
igs0117	-1,3	-1,1	-3,4	0,073	0,808	**0,000**	SMU,124	SMU,125	fw
igs0117	-1,1	-1,1	1,4	0,854	0,816	0,386	SMU,124	SMU,125	rc
igs0118	-1,0	-1,1	-1,1	0,325	0,242	**0,012**	SMU,125	SMU,127	fw
igs0118	1,0	-1,0	-1,0	0,920	0,874	0,929	SMU,125	SMU,127	rc
igs0119	-1,1	1,4	-1,2	0,105	**0,003**	**0,009**	SMU,127	SMU,128	fw
igs0119	-1,1	-1,3	-1,4	0,507	0,105	**0,029**	SMU,127	SMU,128	rc
igs0120	1,4	1,1	1,1	**0,000**	0,342	0,269	SMU,128	SMU,129	fw
igs0120	1,2	-1,3	-1,6	0,492	0,312	0,101	SMU,128	SMU,129	rc
igs0121	-1,2	1,9	1,5	0,144	**0,002**	**0,005**	SMU,129	SMU,130	fw
igs0121	-1,1	-1,9	-2,9	0,174	**0,000**	**0,000**	SMU,129	SMU,130	rc
igs0122	-1,1	1,0	1,0	0,506	0,902	0,897	SMU,130	SMU,131	fw
igs0122	1,1	-1,4	1,9	0,722	0,430	0,209	SMU,130	SMU,131	rc
igs0123	1,2	1,1	-1,0	**0,004**	0,488	0,777	SMU,131	SMU,132	fw
igs0123	-1,0	-1,5	-1,2	0,950	0,130	0,427	SMU,131	SMU,132	rc
igs0124	1,6	1,6	-1,6	**0,000**	**0,000**	**0,000**	SMU,132	SMU,133c	fw
igs0124	-1,3	-1,6	1,7	0,314	**0,009**	**0,016**	SMU,132	SMU,133c	rc
igs0125	-1,2	-1,1	-1,1	0,360	0,711	0,538	SMU,133c	SMU,134	fw
igs0125	1,7	-1,0	1,2	**0,000**	0,548	0,146	SMU,133c	SMU,134	rc
igs0126	1,2	-1,3	-1,4	0,241	0,197	0,099	SMU,134	SMU,135	fw
igs0126	1,1	-1,2	-1,2	0,341	0,108	0,181	SMU,134	SMU,135	rc
igs0127	-1,4	-1,4	1,6	**0,000**	**0,000**	**0,003**	SMU,135	SMU,136c	fw

igs0127	1,2	-1,4	-1,2	0,264	0,199	0,333	SMU,135	SMU,136c	rc
igs0128	-1,4	-2,5	**2,5**	**0,009**	**0,000**	**0,000**	SMU,136c	SMU,137	fw
igs0128	-1,5	-1,6	1,6	**0,007**	**0,028**	0,079	SMU,136c	SMU,137	rc
igs0129	-1,0	1,1	1,3	0,553	0,491	0,053	SMU,137	SMU,138	fw
igs0129	1,1	-1,2	-1,1	0,237	0,184	0,382	SMU,137	SMU,138	rc
igs0130	-1,2	-1,3	-1,3	0,318	0,123	0,178	SMU,138	SMU,139	fw
igs0130	-1,5	-1,8	-1,4	**0,014**	**0,000**	**0,035**	SMU,138	SMU,139	rc
igs0131	-1,1	1,1	1,2	**0,002**	**0,008**	**0,003**	SMU,139	SMU,140	fw
igs0131	-1,2	-1,1	-1,3	0,401	0,441	0,195	SMU,139	SMU,140	rc
igs0133	1,1	-1,1	-1,0	0,744	0,717	0,952	SMU,141	SMU,143c	fw
igs0133	-1,2	-1,1	1,1	0,481	0,550	0,667	SMU,141	SMU,143c	rc
igs0134	-1,5	-1,7	1,7	**0,000**	**0,010**	**0,002**	SMU,143c	SMU,144c	fw
igs0134	1,2	-1,1	-1,3	**0,004**	**0,014**	**0,018**	SMU,143c	SMU,144c	rc
igs0135	-1,4	1,0	-1,1	0,552	0,927	0,917	SMU,144c	SMU,145	fw
igs0135	1,2	-2,4	-1,3	0,617	**0,000**	0,222	SMU,144c	SMU,145	rc
igs0136	1,3	-1,1	1,3	**0,003**	0,532	**0,000**	SMU,145	SMU,148	fw
igs0136	-1,0	1,3	-1,3	0,828	0,445	0,373	SMU,145	SMU,148	rc
igs0137	-1,2	-1,0	1,2	0,102	0,966	0,169	SMU,148	SMU,149	fw
igs0137	-1,1	1,4	-1,2	0,748	0,237	0,580	SMU,148	SMU,149	rc
igs0138	1,0	-1,1	1,2	0,753	0,361	**0,000**	SMU,149	SMU,150	fw
igs0138	1,7	**2,0**	1,7	**0,033**	**0,007**	0,059	SMU,149	SMU,150	rc
igs0140	-1,0	-1,1	1,4	0,891	0,364	**0,033**	SMU,151	SMU,152	fw
igs0140	**19,1**	**17,6**	**13,4**	**0,000**	**0,000**	**0,000**	SMU,151	SMU,152	rc
igs0141	-1,0	-1,1	1,0	0,772	0,354	0,670	SMU,152	SMU,153	fw
igs0141	**28,9**	**34,7**	**42,7**	**0,000**	**0,000**	**0,000**	SMU,152	SMU,153	rc
igs0142	1,2	1,1	-1,0	**0,000**	**0,024**	0,651	SMU,153	SMU,154	fw
igs0142	**12,6**	**15,8**	**16,9**	**0,000**	**0,000**	**0,000**	SMU,153	SMU,154	rc
igs0143	-1,1	-1,1	-1,0	0,097	**0,022**	0,554	SMU,154	SMU,155	fw
igs0143	-1,0	1,3	-1,2	0,960	0,191	0,368	SMU,154	SMU,155	rc
igs0144	-1,1	-1,0	1,3	0,447	0,979	**0,020**	SMU,157	SMU,158	fw
igs0144	-1,6	-1,4	-1,2	0,098	0,146	0,617	SMU,157	SMU,158	rc
igs0145	-1,1	1,2	-1,4	0,356	**0,012**	**0,000**	SMU,159	SMU,160	fw
igs0145	-1,1	-1,2	1,2	0,826	0,536	0,422	SMU,159	SMU,160	rc
igs0146	-1,2	-1,1	**3,7**	0,262	0,701	**0,000**	SMU,161	SMU,162c	fw
igs0146	**2,1**	1,1	1,0	**0,036**	0,847	0,940	SMU,161	SMU,162c	rc
igs0147	-1,1	-1,3	1,6	0,305	**0,020**	**0,004**	SMU,162c	SMU,163c	fw
igs0147	1,0	1,5	-1,3	0,977	**0,004**	0,169	SMU,162c	SMU,163c	rc
igs0148	-1,0	-1,2	-1,2	0,989	0,568	0,528	SMU,163c	SMU,164	fw
igs0148	-1,0	1,1	1,1	0,694	0,097	0,430	SMU,163c	SMU,164	rc
igs0150	-1,1	-2,3	-1,7	0,691	**0,009**	**0,036**	SMU,165	SMU,166	fw
igs0150	-1,4	1,2	-1,3	0,521	0,660	0,578	SMU,165	SMU,166	rc
igs0151	1,1	-1,0	-1,0	0,091	0,995	0,600	SMU,168	SMU,169	fw
igs0151	-1,1	1,6	-1,3	0,886	0,133	0,526	SMU,168	SMU,169	rc
igs0153	-1,3	-1,1	1,1	**0,010**	0,568	0,310	SMU,170	SMU,172	fw
igs0153	-1,0	1,0	1,0	0,938	0,954	0,979	SMU,170	SMU,172	rc
igs0154	1,0	1,2	1,1	0,938	0,220	0,738	SMU,173	SMU,174c	fw
igs0154	-1,5	-1,5	-1,3	**0,045**	**0,004**	0,126	SMU,173	SMU,174c	rc
igs0155	1,1	-1,0	1,3	0,700	0,955	0,093	SMU,174c	SMU,175	fw
igs0155	-1,1	1,2	1,3	0,550	**0,000**	**0,000**	SMU,174c	SMU,175	rc
igs0156	1,1	1,1	1,3	**0,012**	0,210	**0,000**	SMU,175	SMU,176	fw
igs0156	1,1	1,0	1,3	0,336	0,979	**0,004**	SMU,175	SMU,176	rc
igs0157	-1,1	1,2	1,0	0,108	**0,041**	0,758	SMU,176	SMU,177	fw
igs0157	-1,1	-1,1	-1,4	0,817	0,806	0,503	SMU,176	SMU,177	rc
igs0158	1,0	-1,1	1,6	0,549	0,426	**0,000**	SMU,178	SMU,179	fw
igs0158	-1,1	1,0	-1,5	0,636	0,962	0,140	SMU,178	SMU,179	rc
igs0160	-1,2	-1,0	1,0	**0,017**	0,946	0,876	SMU,180	SMU,181	fw
igs0160	-1,0	-1,1	-1,1	0,894	0,220	0,165	SMU,180	SMU,181	rc
igs0161	-1,0	-1,3	1,1	0,904	0,085	0,472	SMU,181	SMU,182	fw
igs0161	-1,2	-1,7	1,5	0,605	0,238	0,256	SMU,181	SMU,182	rc
igs0162	**2,2**	**3,6**	1,4	**0,000**	**0,000**	**0,016**	SMU,183	SMU,184	fw
igs0162	**2,3**	-2,2	-1,1	**0,001**	**0,004**	0,614	SMU,183	SMU,184	rc
igs0163	-1,0	1,2	1,5	0,756	0,256	0,139	SMU,185	SMU,186	fw
igs0163	1,2	-1,4	1,6	0,756	0,494	0,395	SMU,185	SMU,186	rc
igs0164	-1,5	-1,4	1,5	0,224	0,191	0,252	SMU,186	SMU,187c	fw
igs0164	-1,1	1,0	-1,3	0,832	0,920	0,396	SMU,186	SMU,187c	rc
igs0165	1,7	1,0	-1,1	**0,000**	0,829	0,533	SMU,188c	SMU,189	fw

igs0165	1,1	1,3	1,3	0,596	0,481	0,236	SMU,188c	SMU,189	rc
igs0166	1,1	-1,1	1,8	**0,013**	0,288	**0,000**	SMU,189	SMUr04	fw
igs0166	**2,1**	1,1	1,8	**0,000**	0,233	**0,000**	SMU,189	SMUr04	rc
igs0167	1,2	1,3	1,1	**0,013**	0,121	0,320	SMUr04	SMUt21	fw
igs0167	**2,3**	1,1	1,6	**0,000**	**0,033**	**0,000**	SMUr04	SMUt21	rc
igs0168	1,0	-1,1	1,4	0,757	0,567	**0,001**	SMUt21	SMUr05	fw
igs0168	1,4	-1,1	1,5	**0,000**	0,104	**0,000**	SMUt21	SMUr05	rc
igs0169	1,0	1,1	-1,1	0,863	0,196	0,130	SMUr05	SMUr06	fw
igs0169	**2,0**	1,1	1,6	**0,000**	0,350	**0,000**	SMUr05	SMUr06	rc
igs0171	1,0	-1,1	1,0	0,373	0,384	0,235	SMUt23	SMUt24	fw
igs0171	1,7	1,0	1,8	**0,003**	0,910	**0,001**	SMUt23	SMUt24	rc
igs0181	-1,5	-1,2	1,1	**0,000**	0,115	0,511	SMUt33	SMU,191c	fw
igs0181	1,7	-1,4	1,1	0,644	0,782	0,933	SMUt33	SMU,191c	rc
igs0184	-1,1	1,1	-1,2	0,714	0,757	0,581	SMU,194c	SMU,195c	fw
igs0184	-1,2	1,1	**2,3**	**0,020**	0,564	**0,000**	SMU,194c	SMU,195c	rc
igs0188	1,3	1,4	1,1	**0,000**	**0,001**	0,239	SMU,199c	SMU,200c	fw
igs0188	1,0	1,0	-1,2	**0,010**	0,600	**0,004**	SMU,199c	SMU,200c	rc
igs0191	1,0	-1,1	1,3	0,853	0,775	0,159	SMU,202c	SMU,204c	fw
igs0191	-1,5	-1,4	1,2	**0,000**	**0,016**	0,158	SMU,202c	SMU,204c	rc
igs0192	-1,9	-1,3	-1,7	0,116	0,375	0,094	SMU,204c	SMU,205c	fw
igs0192	1,3	-1,1	1,6	**0,000**	**0,015**	**0,000**	SMU,204c	SMU,205c	rc
igs0193	-1,4	-1,1	-1,2	0,198	0,820	0,471	SMU,205c	SMU,206c	fw
igs0193	-1,1	-1,5	1,3	0,286	0,144	0,131	SMU,205c	SMU,206c	rc
igs0194	1,0	1,2	-1,0	0,815	0,364	0,852	SMU,207c	SMU,208c	fw
igs0194	1,3	1,1	1,0	0,166	0,627	0,804	SMU,207c	SMU,208c	rc
igs0197	-1,2	1,0	-1,2	0,311	0,871	0,405	SMU,210c	SMU,211c	fw
igs0197	-1,3	-1,5	1,1	**0,003**	**0,047**	0,256	SMU,210c	SMU,211c	rc
igs0200	1,0	-1,1	-1,6	0,680	0,418	**0,022**	SMU,213c	SMU,214c	fw
igs0200	-1,2	-1,5	-1,3	**0,002**	**0,001**	**0,000**	SMU,213c	SMU,214c	rc
igs0202	1,4	-1,0	1,3	0,446	0,972	0,542	SMU,215c	SMU,216c	fw
igs0202	1,5	-1,7	1,3	**0,024**	**0,000**	0,220	SMU,215c	SMU,216c	rc
igs0203	1,5	-1,7	**2,6**	0,444	0,440	0,069	SMU,216c	SMU,217c	fw
igs0203	**2,3**	1,1	-1,4	**0,000**	0,061	0,048	SMU,216c	SMU,217c	rc
igs0204	1,2	-1,1	1,4	0,475	0,743	0,327	SMU,217c	SMU,218	fw
igs0204	1,1	-1,4	1,6	0,562	0,130	0,103	SMU,217c	SMU,218	rc
igs0206	1,5	-1,0	1,2	0,388	0,932	0,779	SMU,219	SMU,220c	fw
igs0206	-1,1	-1,1	1,2	0,827	0,786	0,571	SMU,219	SMU,220c	rc
igs0207	1,3	1,3	1,5	0,356	0,340	0,073	SMU,220c	SMU,221c	fw
igs0207	1,1	-1,2	-1,1	0,498	0,291	0,722	SMU,220c	SMU,221c	rc
igs0208	1,0	-1,3	-1,0	0,946	**0,020**	0,781	SMU,221c	SMU,222c	fw
igs0208	-1,0	1,1	-1,0	0,823	0,204	0,751	SMU,221c	SMU,222c	rc
igs0209	-1,2	-1,3	-1,4	0,559	0,225	0,101	SMU,222c	SMU,223c	fw
igs0209	1,4	-1,3	-1,0	**0,000**	0,054	0,897	SMU,222c	SMU,223c	rc
igs0210	-1,4	-1,7	1,4	**0,002**	**0,001**	**0,007**	SMU,223c	SMU,224c	fw
igs0210	-1,2	-1,2	1,1	**0,034**	0,182	0,398	SMU,223c	SMU,224c	rc
igs0211	-1,4	-1,7	-1,3	**0,029**	**0,001**	0,247	SMU,225c	SMU,226c	fw
igs0211	1,1	-1,0	-1,1	0,745	0,890	0,880	SMU,225c	SMU,226c	rc
igs0212	1,1	-1,2	1,4	0,462	**0,013**	**0,000**	SMU,226c	SMU,227c	fw
igs0212	-1,1	-1,0	-1,1	0,672	0,934	0,611	SMU,226c	SMU,227c	rc
igs0213	1,0	-1,2	1,1	0,884	0,293	0,792	SMU,227c	SMU,228	fw
igs0213	1,2	-1,0	-1,1	0,269	0,816	0,607	SMU,227c	SMU,228	rc
igs0214	1,3	-1,2	-1,2	**0,030**	**0,002**	**0,018**	SMU,229	SMU,231	fw
igs0214	1,0	-1,2	-1,5	0,964	0,804	0,521	SMU,229	SMU,231	rc
igs0215	1,1	1,2	1,6	0,194	**0,027**	**0,001**	SMU,232	SMU,233	fw
igs0215	-1,3	-2,2	1,4	0,339	**0,032**	0,307	SMU,232	SMU,233	rc
igs0216	-1,0	1,2	1,0	0,855	0,252	0,678	SMU,233	SMU,234	fw
igs0216	-1,3	1,0	1,1	0,311	0,975	0,670	SMU,233	SMU,234	rc
igs0217	-1,1	-1,3	-1,5	**0,000**	**0,009**	**0,003**	SMU,234	SMU,235	fw
igs0217	-1,1	-1,4	1,3	0,160	**0,005**	0,068	SMU,234	SMU,235	rc
igs0218	1,1	-1,1	1,2	0,853	0,630	0,485	SMU,235	SMU,236c	fw
igs0218	1,1	1,3	-1,1	0,889	0,526	0,801	SMU,235	SMU,236c	rc
igs0220	-1,2	-1,1	-1,4	**0,020**	0,311	**0,003**	SMU,238c	SMU,239c	fw
igs0220	-1,0	-1,2	-1,3	0,949	0,116	**0,001**	SMU,238c	SMU,239c	rc
igs0221	1,1	-1,1	-1,3	0,640	0,366	**0,031**	SMU,239c	SMU,241c	fw
igs0221	-1,1	-1,0	-1,2	0,291	0,964	0,200	SMU,239c	SMU,241c	rc
igs0222	1,2	-1,0	1,0	**0,000**	0,864	0,792	SMU,242c	SMU,243	fw

igs0222	-1,0	1,1	-1,3	0,939	0,758	0,170	SMU,242c	SMU,243	rc
igs0223	-1,0	-1,2	1,2	0,891	0,180	0,477	SMU,243	SMU,244	fw
igs0223	-1,1	-1,4	-2,5	0,272	**0,030**	**0,001**	SMU,243	SMU,244	rc
igs0224	-1,2	-1,1	1,2	**0,042**	0,482	0,274	SMU,244	SMU,245	fw
igs0224	-1,5	-1,4	1,2	0,446	0,546	0,730	SMU,244	SMU,245	rc
igs0226	1,2	1,2	**2,1**	**0,015**	**0,009**	**0,000**	SMU,246	SMU,247	fw
igs0226	-1,6	-1,4	-1,4	**0,019**	**0,027**	0,072	SMU,246	SMU,247	rc
igs0227	1,5	-1,1	1,3	**0,025**	0,523	0,286	SMU,247	SMU,248	fw
igs0227	-1,1	-1,0	-2,5	0,190	0,651	**0,000**	SMU,247	SMU,248	rc
igs0230	1,1	1,0	-1,4	0,641	0,918	**0,009**	SMU,251	SMU,252	fw
igs0230	-1,0	-1,1	-1,7	0,921	0,712	**0,008**	SMU,251	SMU,252	rc
igs0231	-1,2	-1,2	1,0	0,599	0,661	0,896	SMU,252	SMU,253	fw
igs0231	-1,1	-1,1	1,2	0,695	0,526	0,275	SMU,252	SMU,253	rc
igs0232	1,2	1,1	1,2	**0,044**	0,090	**0,018**	SMU,253	SMU,255	fw
igs0232	1,0	-1,2	-1,2	0,898	0,596	0,437	SMU,253	SMU,255	rc
igs0233	-1,2	1,0	-1,0	0,178	0,864	0,877	SMU,255	SMU,256	fw
igs0233	-1,2	1,0	-1,1	0,663	0,949	0,876	SMU,255	SMU,256	rc
igs0236	1,3	-1,1	1,1	**0,000**	0,639	0,424	SMU,259	SMU,260	fw
igs0236	1,1	-1,1	-1,1	0,579	0,759	0,722	SMU,259	SMU,260	rc
igs0237	-1,4	-1,4	1,0	**0,002**	**0,000**	0,582	SMU,260	SMU,261c	fw
igs0237	-1,4	-1,3	-1,6	0,124	0,298	**0,026**	SMU,260	SMU,261c	rc
igs0238	-1,0	-1,5	-1,4	0,883	**0,042**	**0,029**	SMU,261c	SMU,262	fw
igs0238	1,2	-1,0	1,4	**0,003**	0,938	0,074	SMU,261c	SMU,262	rc
igs0239	-1,4	-1,1	1,2	0,192	0,662	0,198	SMU,262	SMU,263	fw
igs0239	-1,2	1,1	1,1	0,309	0,371	0,144	SMU,262	SMU,263	rc
igs0241	1,2	-1,2	1,7	0,130	**0,040**	**0,000**	SMU,264	SMU,265	fw
igs0241	-1,1	1,3	-1,1	**0,008**	**0,001**	**0,009**	SMU,264	SMU,265	rc
igs0242	-1,1	-1,0	1,1	0,705	0,824	0,733	SMU,265	SMU,267c	fw
igs0242	-1,1	1,0	1,0	0,519	0,764	0,821	SMU,265	SMU,267c	rc
igs0243	-1,1	-1,0	1,1	0,612	0,749	0,506	SMU,267c	SMU,268	fw
igs0243	-1,1	1,0	1,0	0,922	0,978	0,972	SMU,267c	SMU,268	rc
igs0244	-1,0	-1,2	1,1	0,432	**0,034**	0,131	SMU,268	SMU,270	fw
igs0244	-1,3	1,0	-1,0	0,238	0,857	0,848	SMU,268	SMU,270	rc
igs0245	-2,3	-1,6	1,4	**0,000**	**0,007**	**0,010**	SMU,270	SMU,271	fw
igs0245	-1,1	-1,3	-2,4	0,828	0,371	**0,023**	SMU,270	SMU,271	rc
igs0246	-1,2	1,1	1,5	0,061	0,203	**0,001**	SMU,271	SMU,272	fw
igs0246	1,0	-1,3	-2,5	0,922	**0,002**	**0,000**	SMU,271	SMU,272	rc
igs0247	-1,0	-1,1	1,2	0,603	0,152	0,138	SMU,272	SMU,273	fw
igs0247	1,0	-1,4	-2,4	0,752	**0,010**	**0,000**	SMU,272	SMU,273	rc
igs0250	-1,1	1,2	-1,1	0,061	0,341	0,508	SMU,275	SMU,276c	fw
igs0250	1,2	-1,3	-1,1	0,681	0,535	0,794	SMU,275	SMU,276c	rc
igs0251	1,2	-1,3	-1,1	0,223	**0,023**	0,353	SMU,276c	SMU,277	fw
igs0251	1,4	-1,2	-2,4	0,428	0,671	0,094	SMU,276c	SMU,277	rc
igs0252	-1,1	-1,1	-1,2	0,315	0,082	**0,001**	SMU,277	SMU,278	fw
igs0252	1,2	-1,3	-1,0	0,052	**0,000**	0,785	SMU,277	SMU,278	rc
igs0253	1,0	1,0	1,1	0,538	0,799	0,234	SMU,278	SMU,279	fw
igs0253	1,4	-1,1	1,1	0,229	0,711	0,725	SMU,278	SMU,279	rc
igs0254	-1,0	-1,6	-1,3	0,980	**0,004**	**0,033**	SMU,279	SMU,281	fw
igs0254	1,2	-2,0	1,2	0,208	**0,000**	0,375	SMU,279	SMU,281	rc
igs0255	1,0	1,0	-1,1	0,398	0,642	0,284	SMU,281	SMU,283	fw
igs0255	1,1	-1,3	-1,0	0,498	0,105	0,838	SMU,281	SMU,283	rc
igs0257	1,2	-1,0	1,5	**0,012**	0,861	**0,001**	SMU,284	SMU,285	fw
igs0257	-1,1	-1,6	-1,1	0,894	0,342	0,894	SMU,284	SMU,285	rc
igs0258	-1,0	-1,0	1,3	0,897	0,913	**0,010**	SMU,285	SMU,286	fw
igs0258	-1,1	-1,0	-1,1	0,673	0,922	0,708	SMU,285	SMU,286	rc
igs0260	1,1	1,2	-1,1	**0,005**	0,127	0,230	SMU,287	SMU,289	fw
igs0260	1,1	-1,2	1,3	0,703	0,607	0,362	SMU,287	SMU,289	rc
igs0261	1,0	-1,0	-1,1	0,768	0,655	0,085	SMU,289	SMU,290	fw
igs0261	1,1	-1,1	-1,0	0,578	0,533	0,866	SMU,289	SMU,290	rc
igs0262	1,1	1,0	-1,2	0,146	0,680	**0,035**	SMU,290	SMU,291	fw
igs0262	1,1	-1,1	1,3	0,457	0,614	0,108	SMU,290	SMU,291	rc
igs0263	1,1	1,1	-1,1	**0,000**	**0,024**	0,154	SMU,291	SMU,292	fw
igs0263	-1,3	-1,4	-1,1	**0,003**	**0,003**	0,328	SMU,291	SMU,292	rc
igs0264	-1,1	1,3	1,1	0,057	**0,000**	0,506	SMU,292	SMU,293	fw
igs0264	-1,2	1,2	-1,2	**0,002**	**0,047**	0,075	SMU,292	SMU,293	rc
igs0265	1,1	-1,0	1,4	0,128	**0,006**	**0,002**	SMU,293	SMU,294	fw

igs0265	-1,3	-1,2	-1,6	**0,000**	**0,000**	**0,000**	SMU,293	SMU,294	rc
igs0267	1,1	1,3	-1,1	**0,003**	**0,035**	0,600	SMU,296	SMU,297	fw
igs0267	-1,1	1,1	1,0	0,709	0,740	0,829	SMU,296	SMU,297	rc
igs0268	1,2	-1,1	-1,1	0,117	**0,001**	0,097	SMU,297	SMU,298	fw
igs0268	-1,7	-1,2	-1,7	**0,000**	0,068	**0,001**	SMU,297	SMU,298	rc
igs0269	1,1	-1,6	1,1	0,846	0,265	0,826	SMU,298	SMU,299c	fw
igs0269	-1,1	-1,5	1,2	0,699	0,171	0,490	SMU,298	SMU,299c	rc
igs0270	-1,1	1,1	-1,1	0,720	0,572	0,737	SMU,299c	SMU,300	fw
igs0270	-1,1	-1,1	1,0	0,792	0,833	0,955	SMU,299c	SMU,300	rc
igs0271	1,1	-1,5	1,2	0,430	**0,000**	0,111	SMU,301	SMU,302	fw
igs0271	-1,2	-1,1	-1,3	0,397	0,621	0,243	SMU,301	SMU,302	rc
igs0272	1,6	1,5	-1,3	**0,018**	**0,021**	0,385	SMU,302	SMU,303	fw
igs0272	-1,5	-1,7	1,2	0,363	0,196	0,758	SMU,302	SMU,303	rc
igs0273	-1,3	-1,2	1,2	**0,000**	**0,012**	**0,038**	SMU,305	SMU,307	fw
igs0273	1,1	-1,1	1,1	0,629	0,769	0,844	SMU,305	SMU,307	rc
igs0274	1,0	-1,1	1,1	0,882	0,092	0,515	SMU,307	SMU,308	fw
igs0274	-1,3	1,1	-1,1	0,412	0,848	0,683	SMU,307	SMU,308	rc
igs0276	-1,0	-1,3	1,4	0,692	**0,006**	0,118	SMU,310	SMU,311	fw
igs0276	-1,0	1,3	-1,3	0,941	0,406	0,248	SMU,310	SMU,311	rc
igs0277	1,0	-1,1	1,1	0,756	0,342	0,148	SMU,311	SMU,312	fw
igs0277	-1,4	1,2	-1,1	0,292	0,556	0,755	SMU,311	SMU,312	rc
igs0278	-1,1	**2,1**	-1,1	0,134	**0,000**	0,527	SMU,312	SMU,313	fw
igs0278	-1,2	1,4	-1,7	**0,038**	**0,005**	**0,004**	SMU,312	SMU,313	rc
igs0279	1,1	-1,2	-1,1	0,104	0,050	0,267	SMU,313	SMU,314	fw
igs0279	-1,2	1,1	1,1	**0,000**	0,178	0,383	SMU,313	SMU,314	rc
igs0280	-1,1	-1,2	-1,3	0,123	**0,000**	**0,000**	SMU,314	SMU,317	fw
igs0280	-1,0	-1,0	1,6	0,936	0,881	**0,001**	SMU,314	SMU,317	rc
igs0281	-1,0	-1,1	-1,0	0,977	0,713	0,932	SMU,317	SMU,318	fw
igs0281	-1,1	-1,1	1,0	0,539	0,358	0,737	SMU,317	SMU,318	rc
igs0282	-1,3	-1,0	1,2	0,604	0,949	0,640	SMU,318	SMU,320	fw
igs0282	-1,2	1,0	-1,3	0,277	0,959	0,374	SMU,318	SMU,320	rc
igs0285	-1,1	-1,2	1,2	0,766	0,407	0,434	SMU,323	SMU,325	fw
igs0285	1,0	-1,0	-1,2	0,810	0,983	0,384	SMU,323	SMU,325	rc
igs0287	1,3	-1,6	-1,0	**0,000**	**0,000**	0,476	SMU,327	SMU,328	fw
igs0287	1,4	**2,3**	1,2	0,582	0,138	0,787	SMU,327	SMU,328	rc
igs0288	1,5	1,1	1,3	**0,001**	0,338	0,275	SMU,328	SMU,329	fw
igs0288	-1,3	-1,2	-1,2	0,663	0,734	0,737	SMU,328	SMU,329	rc
igs0289	1,0	1,0	1,6	0,544	0,857	**0,000**	SMU,329	SMU,330	fw
igs0289	-1,4	1,1	-1,5	0,168	0,779	0,086	SMU,329	SMU,330	rc
igs0290	1,8	1,4	1,2	**0,000**	0,055	0,273	SMU,330	SMU,331	fw
igs0290	1,1	-1,3	-1,2	0,808	0,481	0,708	SMU,330	SMU,331	rc
igs0291	1,3	-2,8	-1,2	**0,000**	**0,000**	**0,043**	SMU,331	SMU,332	fw
igs0291	-1,1	-1,1	1,2	0,098	0,134	0,109	SMU,331	SMU,332	rc
igs0292	1,2	-1,2	-1,0	0,052	0,416	0,905	SMU,332	SMU,333	fw
igs0292	-1,1	1,0	-1,7	0,856	0,951	0,189	SMU,332	SMU,333	rc
igs0293	1,2	-1,3	-1,8	0,589	0,252	**0,015**	SMU,333	SMU,334	fw
igs0293	-1,2	1,2	1,5	0,514	0,331	0,219	SMU,333	SMU,334	rc
igs0294	-1,2	-1,0	1,1	**0,001**	0,572	0,186	SMU,334	SMU,335	fw
igs0294	1,2	-1,1	-1,6	0,165	0,469	**0,012**	SMU,334	SMU,335	rc
igs0295	1,2	-1,4	1,2	**0,002**	**0,002**	0,133	SMU,335	SMU,336	fw
igs0295	-1,3	**2,0**	-1,1	0,462	0,057	0,871	SMU,335	SMU,336	rc
igs0297	1,0	1,3	-1,2	0,540	**0,000**	**0,000**	SMU,338	SMU,339	fw
igs0297	1,1	-1,3	-1,3	0,733	0,283	0,388	SMU,338	SMU,339	rc
igs0298	1,3	1,0	-1,3	**0,013**	0,721	0,116	SMU,339	SMU,340	fw
igs0298	1,2	-1,3	1,2	0,739	0,588	0,707	SMU,339	SMU,340	rc
igs0299	-1,1	1,4	1,0	0,653	**0,031**	0,905	SMU,340	SMU,341	fw
igs0299	1,7	1,4	-1,1	**0,011**	0,173	0,714	SMU,340	SMU,341	rc
igs0300	-1,2	1,7	1,1	0,227	**0,002**	0,479	SMU,341	SMU,342	fw
igs0300	1,3	-1,1	-1,1	0,129	0,750	0,480	SMU,341	SMU,342	rc
igs0301	-1,4	1,1	1,3	**0,025**	0,382	**0,016**	SMU,342	SMU,343	fw
igs0301	1,2	-1,0	-1,5	**0,015**	0,989	**0,000**	SMU,342	SMU,343	rc
igs0302	-1,2	1,5	1,1	0,282	**0,009**	0,416	SMU,343	SMU,344	fw
igs0302	1,2	1,0	-1,2	0,089	0,896	0,435	SMU,343	SMU,344	rc
igs0304	1,0	1,2	1,0	0,847	0,395	0,902	SMU,345c	SMU,346	fw
igs0304	-1,3	-2,8	1,1	0,173	**0,000**	0,500	SMU,345c	SMU,346	rc
igs0305	-1,3	1,0	-1,4	**0,005**	0,410	**0,012**	SMU,346	SMU,348	fw

igs0305	-1,3	1,0	-1,3	0,312	0,957	0,392	SMU,346	SMU,348	rc
igs0308	1,4	-2,0	-1,7	0,499	0,073	0,252	SMU,350	SMUt34	fw
igs0308	-1,1	-1,7	-1,4	0,485	**0,000**	0,102	SMU,350	SMUt34	rc
igs0309	1,2	-1,0	**2,0**	0,659	0,936	0,167	SMUt34	SMUt35	fw
igs0309	1,2	1,1	-1,4	0,123	0,619	**0,015**	SMUt34	SMUt35	rc
igs0310	1,3	-1,4	-1,3	0,708	0,657	0,674	SMUt35	SMU,351	fw
igs0310	1,4	-1,5	-1,3	0,649	0,594	0,690	SMUt35	SMU,351	rc
igs0313	-1,9	-1,9	-1,0	**0,000**	**0,003**	0,792	SMU,355	SMU,356	fw
igs0313	-1,1	**2,6**	**2,9**	0,690	**0,002**	**0,002**	SMU,355	SMU,356	rc
igs0314	1,3	-1,1	-1,5	**0,019**	0,680	**0,001**	SMU,356	SMU,357	fw
igs0314	1,1	1,9	-1,1	0,829	0,057	0,797	SMU,356	SMU,357	rc
igs0316	-1,4	-1,4	1,1	**0,004**	**0,028**	0,248	SMU,358	SMU,359	fw
igs0316	1,0	-1,2	1,1	0,812	0,253	0,742	SMU,358	SMU,359	rc
igs0317	1,3	-1,0	1,4	**0,028**	0,850	0,094	SMU,359	SMU,360	fw
igs0317	-1,2	-1,2	1,1	0,652	0,646	0,853	SMU,359	SMU,360	rc
igs0318	-1,2	-1,3	-1,1	0,068	0,050	0,531	SMU,360	SMU,361	fw
igs0318	-1,1	-1,4	1,3	0,767	0,131	0,236	SMU,360	SMU,361	rc
igs0319	1,0	-1,1	-1,0	0,612	0,187	0,859	SMU,361	SMU,362	fw
igs0319	1,0	1,0	-1,2	0,880	0,919	0,657	SMU,361	SMU,362	rc
igs0320	1,4	1,1	-1,2	**0,000**	0,333	0,559	SMU,362	SMU,363	fw
igs0320	1,0	1,2	-1,0	0,965	0,754	0,962	SMU,362	SMU,363	rc
igs0321	1,3	-1,5	1,6	**0,000**	**0,000**	**0,000**	SMU,363	SMU,364	fw
igs0321	1,5	-1,0	1,5	**0,002**	0,484	**0,002**	SMU,363	SMU,364	rc
igs0322	-1,3	-1,2	-1,7	**0,001**	**0,045**	**0,000**	SMU,364	SMU,365	fw
igs0322	1,1	-1,3	1,2	0,849	0,634	0,690	SMU,364	SMU,365	rc
igs0324	-1,1	1,4	-1,3	0,361	**0,005**	**0,016**	SMU,366	SMU,367	fw
igs0324	1,0	-1,4	-2,0	0,953	0,325	0,047	SMU,366	SMU,367	rc
igs0325	-1,3	-1,1	-1,1	0,619	0,828	0,858	SMU,367	SMU,368c	fw
igs0325	-1,0	-1,4	1,1	0,900	0,147	0,658	SMU,367	SMU,368c	rc
igs0327	1,1	1,0	-1,2	0,539	0,776	0,346	SMU,369c	SMU,370	fw
igs0327	1,0	-1,0	1,3	0,861	0,943	**0,003**	SMU,369c	SMU,370	rc
igs0328	-1,0	-1,3	-1,4	0,939	**0,000**	**0,000**	SMU,371	SMU,372	fw
igs0328	**2,3**	-1,3	-3,7	0,055	0,403	**0,000**	SMU,371	SMU,372	rc
igs0331	-1,1	-1,3	1,3	0,055	**0,004**	**0,001**	SMU,376	SMU,378	fw
igs0331	1,3	1,3	-1,1	0,224	0,197	0,617	SMU,376	SMU,378	rc
igs0332	-1,0	-1,1	-1,2	0,895	0,843	0,571	SMU,379	SMU,381c	fw
igs0332	-1,3	-1,0	1,2	0,074	0,949	0,286	SMU,379	SMU,381c	rc
igs0333	1,2	-1,0	-1,0	0,331	0,847	0,820	SMU,381c	SMU,382c	fw
igs0333	1,0	-1,2	1,3	0,578	**0,007**	**0,000**	SMU,381c	SMU,382c	rc
igs0334	1,0	1,1	-1,1	0,957	0,662	0,206	SMU,382c	SMU,383c	fw
igs0334	1,1	-1,2	-1,2	0,357	**0,007**	0,058	SMU,382c	SMU,383c	rc
igs0335	1,0	-1,2	1,5	0,423	**0,029**	**0,009**	SMU,383c	SMU,384	fw
igs0335	1,3	1,8	**2,5**	0,089	**0,009**	**0,001**	SMU,383c	SMU,384	rc
igs0338	-1,5	**2,5**	1,2	**0,004**	**0,000**	**0,016**	SMU,387	SMU,388	fw
igs0338	1,4	1,6	1,2	0,170	0,136	0,302	SMU,387	SMU,388	rc
igs0339	1,3	1,1	-1,1	0,618	0,883	0,893	SMU,390	SMU,391c	fw
igs0339	**2,0**	1,1	-1,7	**0,011**	0,762	0,077	SMU,390	SMU,391c	rc
igs0340	-1,2	-1,0	1,5	0,577	0,970	0,249	SMU,391c	SMU,392c	fw
igs0340	-1,2	-1,0	-1,5	0,237	0,920	**0,000**	SMU,391c	SMU,392c	rc
igs0341	1,3	-1,2	1,2	0,188	0,533	0,419	SMU,392c	SMU,393	fw
igs0341	1,1	1,1	-1,1	0,867	0,759	0,845	SMU,392c	SMU,393	rc
igs0342	-1,2	-1,7	1,4	0,458	0,077	0,343	SMU,393	SMU,394c	fw
igs0342	-1,1	-1,5	-1,1	0,875	0,462	0,887	SMU,393	SMU,394c	rc
igs0343	-1,1	-1,7	1,2	0,661	**0,003**	0,199	SMU,394c	SMU,395	fw
igs0343	-1,1	-1,1	-1,1	0,454	**0,013**	0,137	SMU,394c	SMU,395	rc
igs0344	**3,2**	-2,2	1,8	**0,000**	**0,000**	**0,000**	SMU,395	SMU,396	fw
igs0344	**2,5**	-1,4	-2,9	0,131	0,543	0,104	SMU,395	SMU,396	rc
igs0345	1,3	-1,4	1,0	**0,026**	**0,004**	0,838	SMU,396	SMU,399	fw
igs0345	1,2	-1,4	1,6	0,692	0,407	0,322	SMU,396	SMU,399	rc
igs0346	-1,7	-1,3	-1,2	**0,039**	0,238	0,490	SMU,400	SMU,401c	fw
igs0346	1,2	**2,3**	-1,1	0,550	**0,020**	0,895	SMU,400	SMU,401c	rc
igs0347	-1,3	-1,0	-1,4	0,527	0,993	0,577	SMU,401c	SMU,402	fw
igs0347	-1,1	1,6	1,6	0,753	0,179	0,232	SMU,401c	SMU,402	rc
igs0348	-1,0	1,1	1,4	0,911	0,796	0,460	SMU,402	SMU,403	fw
igs0348	1,1	1,3	-1,2	0,623	0,140	0,197	SMU,402	SMU,403	rc
igs0351	1,0	1,0	1,0	0,803	0,772	0,822	SMU,405c	SMU,406c	fw

igs0351	-1,1	-1,8	1,8	0,628	**0,008**	**0,000**	SMU,405c	SMU,406c	rc
igs0352	1,0	1,0	-1,3	0,591	0,913	**0,031**	SMU,406c	SMU,407	fw
igs0352	-1,1	1,8	-1,1	0,416	**0,000**	0,261	SMU,406c	SMU,407	rc
igs0353	1,3	-1,1	-1,0	**0,001**	0,598	0,873	SMU,407	SMU,408	fw
igs0353	-2,7	-2,3	1,2	**0,000**	**0,002**	0,090	SMU,407	SMU,408	rc
igs0354	1,8	1,1	1,2	**0,000**	0,618	0,081	SMU,408	SMU,409	fw
igs0354	-1,1	-1,0	1,0	0,380	0,956	0,889	SMU,408	SMU,409	rc
igs0355	-1,0	1,0	1,2	0,906	0,452	**0,001**	SMU,409	SMU,410	fw
igs0355	1,0	1,1	-1,3	0,858	0,686	0,144	SMU,409	SMU,410	rc
igs0356	-1,3	-1,4	1,3	0,199	0,142	0,258	SMU,410	SMU,411c	fw
igs0356	-1,3	-1,7	1,4	0,181	**0,003**	0,127	SMU,410	SMU,411c	rc
igs0357	-1,1	1,0	1,6	0,797	0,944	0,079	SMU,412c	SMU,413	fw
igs0357	-1,2	1,0	-1,2	0,595	0,967	0,596	SMU,412c	SMU,413	rc
igs0359	1,5	-1,3	**2,1**	**0,000**	**0,000**	**0,000**	SMU,414	SMU,415	fw
igs0359	1,7	1,2	-2,3	0,180	0,695	**0,045**	SMU,414	SMU,415	rc
igs0361	1,2	-1,2	-1,2	**0,000**	**0,017**	0,051	SMU,416	SMUt36	fw
igs0361	1,1	-1,1	-1,1	0,745	0,698	0,718	SMU,416	SMUt36	rc
igs0362	1,1	-1,2	-1,3	0,680	0,522	0,175	SMUt36	SMU,417	fw
igs0362	-1,0	1,2	-1,4	0,927	0,665	0,421	SMUt36	SMU,417	rc
igs0363	-1,2	-1,3	1,3	0,116	**0,000**	**0,001**	SMU,417	SMU,418	fw
igs0363	1,3	1,1	1,1	0,094	0,589	0,465	SMU,417	SMU,418	rc
igs0366	1,1	-1,1	1,4	0,226	0,385	**0,011**	SMU,421	SMU,422	fw
igs0366	1,6	1,0	-1,2	**0,000**	0,848	0,398	SMU,421	SMU,422	rc
igs0367	1,0	-1,2	-1,3	0,857	**0,002**	**0,002**	SMU,422	SMU,423	fw
igs0367	1,5	1,7	1,7	0,098	0,053	**0,043**	SMU,422	SMU,423	rc
igs0368	1,0	-1,1	1,2	0,893	0,242	**0,003**	SMU,423	SMU,424	fw
igs0368	**9,1**	**20,1**	**18,5**	**0,000**	**0,000**	**0,000**	SMU,423	SMU,424	rc
igs0370	1,1	-1,2	1,1	0,391	0,190	0,144	SMU,427	SMU,428	fw
igs0370	1,5	1,8	1,6	0,154	0,069	0,090	SMU,427	SMU,428	rc
igs0371	-1,3	-1,0	1,6	**0,006**	0,479	**0,000**	SMU,428	SMU,429c	fw
igs0371	-1,1	-1,0	-1,7	0,436	0,620	**0,000**	SMU,428	SMU,429c	rc
igs0372	1,2	1,2	-1,0	0,306	0,188	0,867	SMU,429c	SMU,431	fw
igs0372	1,1	-1,0	1,0	0,513	0,610	0,782	SMU,429c	SMU,431	rc
igs0375	-1,0	-1,4	1,1	0,813	**0,000**	0,142	SMU,434	SMU,435	fw
igs0375	-1,1	-1,1	-1,4	0,419	0,538	**0,010**	SMU,434	SMU,435	rc
igs0376	1,4	-1,4	1,1	**0,000**	**0,002**	**0,028**	SMU,435	SMU,436c	fw
igs0376	-1,0	-1,1	-1,7	0,959	0,863	0,185	SMU,435	SMU,436c	rc
igs0377	-1,0	-1,1	-1,1	0,990	0,587	0,419	SMU,436c	SMU,438c	fw
igs0377	-1,1	1,1	1,3	0,258	0,286	**0,009**	SMU,436c	SMU,438c	rc
igs0378	-1,0	-1,0	-1,5	0,944	0,876	0,128	SMU,438c	SMU,439	fw
igs0378	-1,0	1,6	-1,2	0,774	**0,014**	0,104	SMU,438c	SMU,439	rc
igs0379	-1,3	-1,2	-1,1	**0,005**	**0,000**	0,321	SMU,439	SMU,440	fw
igs0379	-1,1	-2,2	-1,4	0,323	**0,000**	**0,002**	SMU,439	SMU,440	rc
igs0380	-1,0	1,2	1,1	0,552	0,115	0,687	SMU,442	SMU,444	fw
igs0380	1,4	-1,2	-1,5	0,157	0,455	0,067	SMU,442	SMU,444	rc
igs0381	-1,4	-1,1	1,1	**0,000**	0,464	0,614	SMU,444	SMU,445	fw
igs0381	-1,4	-1,0	-1,2	**0,019**	0,840	0,358	SMU,444	SMU,445	rc
igs0383	-1,5	1,2	-1,2	**0,000**	0,160	0,208	SMU,446	SMU,447	fw
igs0383	-1,1	-2,0	1,6	0,762	**0,008**	0,109	SMU,446	SMU,447	rc
igs0384	-1,3	-1,1	-1,1	**0,002**	0,568	0,484	SMU,447	SMU,448	fw
igs0384	1,2	-1,6	1,3	0,656	0,342	0,586	SMU,447	SMU,448	rc
igs0385	-1,0	1,8	1,0	0,483	**0,000**	0,546	SMU,448	SMU,449	fw
igs0385	-1,2	-1,2	-1,0	0,224	0,426	0,902	SMU,448	SMU,449	rc
igs0387	1,0	-1,2	1,3	0,860	**0,044**	0,079	SMU,450	SMU,451	fw
igs0387	-1,1	1,1	-1,4	0,723	0,701	**0,029**	SMU,450	SMU,451	rc
igs0388	1,1	**2,1**	-1,8	0,264	**0,000**	**0,001**	SMU,451	SMU,453	fw
igs0388	1,1	1,5	-1,3	0,540	**0,002**	**0,019**	SMU,451	SMU,453	rc
igs0392	-1,2	-2,1	1,3	0,063	**0,002**	0,138	SMU,456	SMU,457	fw
igs0392	-1,2	-1,2	-1,2	0,655	0,724	0,706	SMU,456	SMU,457	rc
igs0393	-1,1	-1,4	-1,4	0,472	**0,002**	**0,000**	SMU,457	SMU,458	fw
igs0393	-1,5	-1,4	1,2	**0,000**	**0,003**	**0,034**	SMU,457	SMU,458	rc
igs0394	1,0	-1,9	1,1	0,531	**0,000**	0,345	SMU,458	SMU,459	fw
igs0394	-1,0	-1,2	1,0	0,793	0,184	0,891	SMU,458	SMU,459	rc
igs0396	-1,7	-1,5	-1,1	**0,002**	**0,004**	0,325	SMU,461	SMU,462	fw
igs0396	-2,9	-2,0	**2,3**	0,158	0,248	0,283	SMU,461	SMU,462	rc
igs0397	1,0	-1,7	1,1	0,769	**0,004**	0,138	SMU,462	SMU,463	fw

igs0397	1,9	1,3	-1,9	0,241	0,654	0,250	SMU,462	SMU,463	rc
igs0398	-1,1	-1,2	-1,2	**0,032**	**0,034**	**0,004**	SMU,463	SMU,464	fw
igs0398	-1,2	-1,3	1,1	0,499	0,212	0,572	SMU,463	SMU,464	rc
igs0399	-1,1	-1,2	1,2	0,820	0,507	0,517	SMU,465	SMU,466	fw
igs0399	-1,0	-1,3	-1,3	0,866	0,320	0,103	SMU,465	SMU,466	rc
igs0400	-1,2	-1,1	-1,0	0,616	0,888	0,932	SMU,466	SMU,467	fw
igs0400	-1,2	-1,2	-1,1	0,441	0,389	0,720	SMU,466	SMU,467	rc
igs0401	-1,1	-1,2	1,3	0,680	0,456	0,415	SMU,467	SMU,469	fw
igs0401	1,3	-1,5	**2,0**	**0,040**	**0,010**	0,054	SMU,467	SMU,469	rc
igs0402	**2,3**	1,9	1,2	0,193	0,396	0,803	SMU,469	SMU,470	fw
igs0402	1,3	1,7	-1,4	**0,021**	**0,000**	**0,000**	SMU,469	SMU,470	rc
igs0403	-1,0	-1,0	1,3	0,671	0,971	0,092	SMU,470	SMU,471	fw
igs0403	-1,2	1,1	-1,0	0,812	0,864	0,959	SMU,470	SMU,471	rc
igs0404	1,0	-1,1	1,1	0,959	**0,028**	0,068	SMU,471	SMU,472	fw
igs0404	2,0	1,0	1,5	**0,001**	0,844	0,104	SMU,471	SMU,472	rc
igs0405	1,2	-1,1	1,3	0,071	0,176	**0,011**	SMU,472	SMU,473	fw
igs0405	1,7	1,1	1,1	**0,038**	0,765	0,666	SMU,472	SMU,473	rc
igs0406	1,0	-1,4	1,1	0,964	0,508	0,884	SMU,473	SMU,474	fw
igs0406	-1,6	-1,1	-1,9	**0,037**	0,570	**0,024**	SMU,473	SMU,474	rc
igs0407	-1,1	-1,6	1,2	0,616	0,225	0,289	SMU,474	SMU,475	fw
igs0407	-1,0	-1,3	-1,2	0,993	0,494	0,430	SMU,474	SMU,475	rc
igs0408	-1,2	-1,0	-1,3	**0,020**	0,867	**0,005**	SMU,475	SMU,478	fw
igs0408	1,2	-1,1	-1,3	0,336	0,745	0,162	SMU,475	SMU,478	rc
igs0410	1,2	1,6	1,0	**0,021**	**0,000**	0,591	SMU,479	SMU,480	fw
igs0410	-1,3	-1,2	-1,3	0,574	0,682	0,535	SMU,479	SMU,480	rc
igs0411	1,0	1,3	-1,1	0,783	**0,000**	**0,011**	SMU,480	SMU,481	fw
igs0411	1,2	-1,2	1,1	0,608	0,587	0,670	SMU,480	SMU,481	rc
igs0412	-1,0	1,2	1,3	0,244	**0,000**	**0,000**	SMU,482	SMU,483	fw
igs0412	1,7	1,2	1,3	**0,003**	**0,046**	**0,013**	SMU,482	SMU,483	rc
igs0413	-1,1	-1,2	-1,2	0,073	0,247	0,106	SMU,484	SMU,485	fw
igs0413	-1,4	1,0	1,2	0,159	0,992	0,421	SMU,484	SMU,485	rc
igs0414	-1,3	1,0	1,7	**0,007**	0,572	**0,001**	SMU,487	SMU,488	fw
igs0414	-1,0	1,3	-1,4	0,723	**0,041**	**0,012**	SMU,487	SMU,488	rc
igs0415	1,2	-1,6	1,8	**0,043**	**0,001**	**0,000**	SMU,489	SMU,490	fw
igs0415	-1,3	-1,8	1,0	0,591	0,254	0,959	SMU,489	SMU,490	rc
igs0416	1,1	1,3	-1,2	0,070	**0,012**	0,088	SMU,490	SMU,491	fw
igs0416	1,8	-1,4	-1,1	**0,000**	0,081	0,650	SMU,490	SMU,491	rc
igs0417	1,0	1,3	1,2	0,727	0,077	0,212	SMU,491	SMU,493	fw
igs0417	1,3	-1,2	-1,4	0,327	0,522	**0,014**	SMU,491	SMU,493	rc
igs0419	1,1	1,1	1,0	**0,033**	0,067	0,434	SMU,494	SMU,495	fw
igs0419	1,1	1,1	-1,4	0,638	0,721	**0,009**	SMU,494	SMU,495	rc
igs0420	-1,2	1,1	1,4	0,427	0,611	0,233	SMU,495	SMU,496	fw
igs0420	-1,1	-1,1	-1,5	0,700	0,565	**0,000**	SMU,495	SMU,496	rc
igs0421	1,3	-1,8	1,2	**0,004**	**0,001**	0,380	SMU,496	SMU,497c	fw
igs0421	1,2	1,1	1,2	0,060	0,097	**0,000**	SMU,496	SMU,497c	rc
igs0422	-1,0	-1,0	-1,2	0,916	0,968	0,116	SMU,497c	SMU,498	fw
igs0422	-1,1	1,7	-1,1	0,082	0,212	0,190	SMU,497c	SMU,498	rc
igs0423	1,2	-1,0	1,5	0,071	0,989	**0,006**	SMU,499	SMU,500	fw
igs0423	1,0	**7,0**	-1,5	0,962	0,063	0,686	SMU,499	SMU,500	rc
igs0424	-1,0	-1,0	1,1	0,706	0,758	**0,004**	SMU,500	SMU,501	fw
igs0424	-1,6	1,7	-1,3	0,310	0,295	0,537	SMU,500	SMU,501	rc
igs0426	1,6	1,5	1,8	**0,004**	**0,000**	**0,006**	SMU,502	SMU,503c	fw
igs0426	-1,9	1,2	1,5	**0,003**	0,233	**0,043**	SMU,502	SMU,503c	rc
igs0427	-1,1	-1,6	1,3	0,317	**0,007**	**0,013**	SMU,503c	SMU,504	fw
igs0427	1,0	-1,2	1,4	0,976	0,348	**0,008**	SMU,503c	SMU,504	rc
igs0428	1,2	-1,3	-1,1	0,200	**0,038**	0,677	SMU,506	SMU,507	fw
igs0428	1,0	**5,4**	1,0	0,894	**0,000**	0,837	SMU,506	SMU,507	rc
igs0430	1,2	1,5	1,9	**0,000**	**0,000**	**0,000**	SMU,508	SMU,509	fw
igs0430	-1,6	1,8	-1,4	**0,000**	**0,037**	0,076	SMU,508	SMU,509	rc
igs0431	-1,4	-1,2	-1,2	0,482	0,570	0,644	SMU,509	SMU,510c	fw
igs0431	1,6	-1,6	1,4	**0,001**	0,071	0,087	SMU,509	SMU,510c	rc
igs0432	-1,3	1,2	-1,8	0,089	0,278	**0,000**	SMU,510c	SMU,512c	fw
igs0432	-1,2	-1,3	-1,0	0,448	0,464	0,995	SMU,510c	SMU,512c	rc
igs0433	-1,2	1,1	-1,2	**0,000**	0,176	0,117	SMU,512c	SMU,513	fw
igs0433	-1,0	-1,4	-1,2	0,975	**0,034**	0,314	SMU,512c	SMU,513	rc
igs0434	1,1	-1,1	1,1	0,878	0,776	0,744	SMU,513	SMU,514	fw

igs0434	-1,1	-1,4	-1,2	0,356	**0,025**	0,192	SMU,513	SMU,514	rc
igs0435	1,2	-1,2	1,4	**0,037**	0,447	**0,008**	SMU,514	SMU,515	fw
igs0435	-1,2	1,0	1,0	0,486	0,769	0,718	SMU,514	SMU,515	rc
igs0436	1,1	1,2	1,0	0,299	**0,009**	0,935	SMU,515	SMU,516	fw
igs0436	-1,1	1,2	-1,1	0,661	**0,039**	0,264	SMU,515	SMU,516	rc
igs0437	-1,4	-1,0	**2,4**	**0,013**	0,539	**0,000**	SMU,518	SMU,520	fw
igs0437	-1,2	-1,1	**2,2**	0,422	0,793	0,048	SMU,518	SMU,520	rc
igs0438	-1,1	-1,2	1,1	0,057	0,274	0,511	SMU,523	SMU,524	fw
igs0438	-1,1	-1,2	1,1	0,613	0,232	0,511	SMU,523	SMU,524	rc
igs0439	-2,3	-1,6	1,7	**0,021**	0,151	**0,013**	SMU,525	SMU,526c	fw
igs0439	-2,3	1,4	1,1	**0,010**	0,090	0,668	SMU,525	SMU,526c	rc
igs0440	-1,2	1,1	-1,0	0,257	0,668	0,763	SMU,526c	SMU,527	fw
igs0440	-1,3	1,0	-1,2	0,070	0,922	0,260	SMU,526c	SMU,527	rc
igs0441	-1,1	-1,4	1,1	0,705	0,154	0,562	SMU,527	SMU,528c	fw
igs0441	1,1	-1,2	-1,3	0,686	0,575	0,236	SMU,527	SMU,528c	rc
igs0442	-1,9	-1,7	-1,1	**0,016**	**0,027**	0,708	SMU,529	SMU,530c	fw
igs0442	-1,2	-1,3	1,3	**0,042**	**0,021**	**0,033**	SMU,529	SMU,530c	rc
igs0443	-1,0	**3,5**	1,1	0,517	**0,000**	0,257	SMU,530c	SMU,531	fw
igs0443	-1,0	1,1	1,4	0,912	0,856	0,396	SMU,530c	SMU,531	rc
igs0447	1,2	**20,3**	-1,1	**0,001**	**0,000**	0,161	SMU,538	SMU,539c	fw
igs0447	1,1	-1,2	-1,2	0,691	0,064	0,109	SMU,538	SMU,539c	rc
igs0448	1,2	-1,1	1,8	0,151	0,146	**0,014**	SMU,539c	SMU,540	fw
igs0448	-1,2	-2,1	1,3	0,774	0,136	0,669	SMU,539c	SMU,540	rc
igs0449	1,2	-1,0	1,0	**0,000**	0,441	0,996	SMU,540	SMU,541	fw
igs0449	1,1	-1,2	-1,1	0,823	0,717	0,835	SMU,540	SMU,541	rc
igs0451	-1,2	-1,2	-1,3	**0,006**	0,051	**0,008**	SMU,543	SMU,545	fw
igs0451	1,2	1,0	-1,6	**0,023**	0,187	**0,030**	SMU,543	SMU,545	rc
igs0453	1,2	1,1	1,1	0,178	0,689	0,728	SMU,546	SMU,547	fw
igs0453	-1,1	-1,1	-1,5	0,706	0,815	0,305	SMU,546	SMU,547	rc
igs0454	-1,1	-2,8	1,4	0,437	**0,000**	0,066	SMU,547	SMU,548	fw
igs0454	-1,0	-1,0	-1,4	0,994	0,945	0,289	SMU,547	SMU,548	rc
igs0456	1,2	-1,0	-1,6	**0,023**	0,882	**0,000**	SMU,550	SMU,551	fw
igs0456	1,0	1,2	1,1	0,999	0,765	0,865	SMU,550	SMU,551	rc
igs0462	-1,1	-1,1	-1,0	0,182	0,572	0,959	SMU,557	SMU,558	fw
igs0462	-1,0	-1,1	-1,2	0,921	0,744	0,633	SMU,557	SMU,558	rc
igs0463	1,1	-1,1	1,1	0,687	0,788	0,624	SMU,558	SMU,560c	fw
igs0463	-1,0	-1,2	-1,0	0,883	0,546	0,982	SMU,558	SMU,560c	rc
igs0464	1,2	-1,1	1,4	0,192	0,832	0,245	SMU,560c	SMU,561c	fw
igs0464	1,5	1,9	**2,6**	**0,006**	**0,001**	**0,000**	SMU,560c	SMU,561c	rc
igs0465	1,1	1,1	-1,0	0,714	0,902	0,951	SMU,561c	SMU,562	fw
igs0465	1,8	1,5	1,6	**0,000**	**0,049**	**0,000**	SMU,561c	SMU,562	rc
igs0466	-1,0	-1,2	1,1	0,943	0,742	0,767	SMU,562	SMU,563	fw
igs0466	1,0	1,1	-1,1	0,843	0,662	0,335	SMU,562	SMU,563	rc
igs0467	1,3	-1,5	-1,0	0,056	**0,000**	0,903	SMU,563	SMU,564	fw
igs0467	-1,2	-1,9	-1,3	0,768	0,393	0,759	SMU,563	SMU,564	rc
igs0468	1,3	1,0	-1,2	**0,007**	0,769	**0,007**	SMU,564	SMU,565c	fw
igs0468	-1,2	-1,5	-1,5	0,423	**0,003**	**0,003**	SMU,564	SMU,565c	rc
igs0469	1,0	-1,7	-1,1	0,958	**0,006**	0,530	SMU,566c	SMU,567	fw
igs0469	1,1	-1,3	1,9	0,494	**0,013**	**0,000**	SMU,566c	SMU,567	rc
igs0470	-1,8	-1,8	1,3	**0,005**	**0,000**	0,124	SMU,568	SMU,569	fw
igs0470	-1,5	-1,2	-1,3	0,312	0,578	0,523	SMU,568	SMU,569	rc
igs0472	1,2	1,0	1,2	**0,002**	0,900	0,105	SMU,571	SMU,572	fw
igs0472	1,1	1,0	-1,1	0,634	0,936	0,751	SMU,571	SMU,572	rc
igs0473	-1,1	-1,0	1,4	0,624	0,776	**0,021**	SMU,573	SMU,574c	fw
igs0473	-1,2	-1,2	1,0	0,224	0,325	0,837	SMU,573	SMU,574c	rc
igs0475	-1,4	-1,0	-1,3	0,088	0,848	0,246	SMU,575c	SMU,576	fw
igs0475	1,0	1,1	-1,2	0,596	0,649	0,068	SMU,575c	SMU,576	rc
igs0476	1,1	1,0	-1,1	0,213	0,917	0,406	SMU,577	SMU,580	fw
igs0476	1,2	-1,1	-1,3	0,354	0,757	0,343	SMU,577	SMU,580	rc
igs0478	1,2	-1,4	-1,4	**0,005**	0,097	0,107	SMU,585	SMU,586	fw
igs0478	1,1	-1,4	1,1	0,725	0,182	0,555	SMU,585	SMU,586	rc
igs0479	-1,9	**2,4**	1,3	**0,000**	**0,000**	**0,041**	SMU,588	SMU,589	fw
igs0479	-2,0	-1,5	-1,0	0,363	0,623	0,994	SMU,588	SMU,589	rc
igs0480	-2,1	1,1	-1,6	**0,000**	0,390	**0,007**	SMU,589	SMU,590c	fw
igs0480	1,7	1,1	-1,3	0,208	0,915	0,506	SMU,589	SMU,590c	rc
igs0481	1,2	1,1	-1,2	0,342	0,141	0,200	SMU,590c	SMU,591c	fw

igs0481	1,4	-1,3	1,1	**0,010**	0,305	0,538	SMU,590c	SMU,591c	rc
igs0483	1,6	1,1	1,5	**0,000**	0,664	**0,010**	SMU,592c	SMU,593	fw
igs0483	1,4	1,2	1,4	0,254	0,375	0,045	SMU,592c	SMU,593	rc
igs0484	-1,2	-1,0	-2,3	0,288	0,601	**0,000**	SMU,593	SMU,594	fw
igs0484	-1,2	1,3	-1,4	0,468	0,391	0,199	SMU,593	SMU,594	rc
igs0485	-1,7	-1,6	-1,3	0,124	0,137	0,301	SMU,594	SMU,595	fw
igs0485	-1,0	-1,2	1,1	0,948	0,605	0,810	SMU,594	SMU,595	rc
igs0486	1,1	-1,4	1,4	0,250	**0,034**	**0,018**	SMU,595	SMU,596	fw
igs0486	1,3	1,0	1,2	0,413	0,944	0,574	SMU,595	SMU,596	rc
igs0487	1,2	-1,1	-1,0	0,153	0,564	0,989	SMU,596	SMU,597	fw
igs0487	1,1	-1,4	-1,2	0,838	0,535	0,651	SMU,596	SMU,597	rc
igs0489	-1,2	-1,1	1,7	0,155	0,204	**0,000**	SMU,598	SMU,599	fw
igs0489	1,2	-1,1	-1,2	0,621	0,839	0,625	SMU,598	SMU,599	rc
igs0490	-1,1	-1,2	-1,2	0,424	0,187	0,054	SMU,599	SMU,600c	fw
igs0490	1,7	-1,2	-2,3	0,116	0,627	**0,016**	SMU,599	SMU,600c	rc
igs0491	-1,1	-1,0	1,5	0,760	0,982	0,342	SMU,600c	SMU,602	fw
igs0491	1,1	-1,2	1,1	0,590	0,481	0,744	SMU,600c	SMU,602	rc
igs0492	1,3	1,4	-1,6	0,084	**0,001**	**0,001**	SMU,602	SMU,603	fw
igs0492	-1,6	1,3	-2,2	**0,002**	0,301	**0,002**	SMU,602	SMU,603	rc
igs0493	1,9	1,2	1,5	**0,000**	0,464	0,157	SMU,603	SMU,604	fw
igs0493	1,2	1,1	1,9	0,448	0,826	0,051	SMU,603	SMU,604	rc
igs0496	1,1	-1,3	1,7	0,144	**0,031**	**0,000**	SMU,606	SMU,607	fw
igs0496	-1,2	-1,3	1,5	0,426	0,167	0,128	SMU,606	SMU,607	rc
igs0497	1,5	1,1	-1,1	**0,000**	0,084	0,214	SMU,607	SMU,608	fw
igs0497	1,2	1,6	1,1	0,527	**0,040**	0,640	SMU,607	SMU,608	rc
igs0498	1,1	-1,2	1,2	0,627	**0,046**	**0,024**	SMU,608	SMU,609	fw
igs0498	-1,3	-1,6	-1,4	0,099	**0,021**	0,142	SMU,608	SMU,609	rc
igs0499	1,4	-1,4	1,2	**0,000**	**0,002**	**0,021**	SMU,609	SMU,610	fw
igs0499	1,1	-1,1	1,0	0,802	0,797	0,969	SMU,609	SMU,610	rc
igs0500	-1,1	-1,3	-1,1	0,200	**0,000**	0,520	SMU,610	SMU,611	fw
igs0500	-1,4	1,3	1,3	0,530	0,640	0,686	SMU,610	SMU,611	rc
igs0501	1,1	-1,2	-1,0	0,185	**0,009**	0,951	SMU,611	SMU,613	fw
igs0501	-1,1	-1,1	-1,1	0,686	0,656	0,822	SMU,611	SMU,613	rc
igs0502	-1,0	-1,5	-1,5	0,798	**0,026**	**0,008**	SMU,613	SMU,614	fw
igs0502	1,0	1,2	-1,5	0,843	0,462	**0,032**	SMU,613	SMU,614	rc
igs0503	-1,1	-1,1	1,1	**0,001**	0,162	0,066	SMU,614	SMU,616	fw
igs0503	1,1	1,1	-1,2	0,633	0,653	0,395	SMU,614	SMU,616	rc
igs0504	1,0	-1,1	-1,2	0,747	0,368	**0,014**	SMU,616	SMU,618	fw
igs0504	-1,3	-2,3	-1,1	0,390	**0,017**	0,678	SMU,616	SMU,618	rc
igs0505	-1,0	-1,0	1,1	0,750	0,798	0,446	SMU,618	SMU,620	fw
igs0505	-1,0	-1,0	-1,2	0,873	0,910	0,490	SMU,618	SMU,620	rc
igs0506	-1,1	-1,4	1,1	0,262	**0,007**	0,371	SMU,620	SMU,621c	fw
igs0506	-1,3	1,1	-1,3	**0,005**	0,246	**0,030**	SMU,620	SMU,621c	rc
igs0507	1,5	1,1	-1,5	**0,043**	0,334	**0,004**	SMU,621c	SMU,622c	fw
igs0507	1,8	1,2	1,2	**0,020**	0,207	0,157	SMU,621c	SMU,622c	rc
igs0508	1,5	-1,1	1,1	**0,037**	0,692	0,440	SMU,622c	SMU,623c	fw
igs0508	1,3	1,3	-1,5	0,082	**0,049**	**0,018**	SMU,622c	SMU,623c	rc
igs0509	1,2	-1,6	-1,3	0,445	**0,028**	0,425	SMU,623c	SMU,624	fw
igs0509	1,5	-1,3	1,9	0,087	0,091	**0,001**	SMU,623c	SMU,624	rc
igs0510	-1,2	-2,4	1,0	0,099	**0,000**	0,846	SMU,624	SMU,625	fw
igs0510	-1,3	1,5	-1,3	0,517	0,374	0,477	SMU,624	SMU,625	rc
igs0511	1,4	-1,1	-1,3	**0,001**	0,225	**0,020**	SMU,626	SMU,627	fw
igs0511	1,1	**8,3**	1,3	0,430	**0,000**	**0,041**	SMU,626	SMU,627	rc
igs0512	1,0	-1,2	**2,5**	0,873	0,314	**0,000**	SMU,627	SMU,628	fw
igs0512	1,0	1,6	-1,0	0,912	0,353	0,952	SMU,627	SMU,628	rc
igs0513	-1,2	-1,5	1,1	**0,020**	**0,002**	0,056	SMU,628	SMU,629	fw
igs0513	1,3	1,1	1,1	0,316	0,607	0,766	SMU,628	SMU,629	rc
igs0514	-1,1	-1,1	-1,0	0,188	**0,034**	0,870	SMU,629	SMU,630	fw
igs0514	-1,0	-1,5	-1,2	0,865	0,117	0,438	SMU,629	SMU,630	rc
igs0515	1,0	1,3	1,2	0,733	**0,022**	0,099	SMU,630	SMU,631	fw
igs0515	-1,1	-1,2	1,1	0,843	0,627	0,740	SMU,630	SMU,631	rc
igs0516	1,1	-1,4	-1,1	**0,047**	**0,008**	0,506	SMU,631	SMU,632	fw
igs0516	-1,9	1,2	1,0	0,100	0,472	0,960	SMU,631	SMU,632	rc
igs0517	1,1	-1,6	1,6	0,352	**0,001**	**0,000**	SMU,632	SMU,633	fw
igs0517	1,2	-1,7	-1,2	0,565	0,052	0,625	SMU,632	SMU,633	rc
igs0518	-1,3	1,3	-1,1	0,448	0,369	0,862	SMU,633	SMU,634	fw

igs0518	-1,2	-1,1	-1,3	0,432	0,664	0,222	SMU,633	SMU,634	rc
igs0519	-2,0	-1,3	1,9	**0,037**	0,269	**0,022**	SMU,634	SMU,635	fw
igs0519	-1,8	1,5	1,4	**0,014**	0,251	0,155	SMU,634	SMU,635	rc
igs0520	1,1	1,2	1,5	0,645	0,161	**0,013**	SMU,635	SMU,636	fw
igs0520	1,2	1,2	1,1	0,753	0,699	0,915	SMU,635	SMU,636	rc
igs0521	-1,8	1,2	-1,4	**0,000**	0,274	**0,024**	SMU,636	SMU,637c	fw
igs0521	-1,3	-1,2	-2,4	0,306	0,460	**0,006**	SMU,636	SMU,637c	rc
igs0522	1,1	-1,0	-1,2	0,284	0,852	0,147	SMU,637c	SMU,638	fw
igs0522	-1,1	-1,0	1,0	0,463	0,841	0,994	SMU,637c	SMU,638	rc
igs0524	1,3	1,0	-1,9	**0,000**	0,662	**0,000**	SMU,639	SMU,640c	fw
igs0524	-1,2	-1,1	1,5	0,262	0,272	**0,001**	SMU,639	SMU,640c	rc
igs0525	-1,4	-1,5	1,6	0,072	**0,035**	**0,023**	SMU,640c	SMU,641	fw
igs0525	1,1	1,2	-1,2	0,769	0,653	0,539	SMU,640c	SMU,641	rc
igs0526	-1,1	1,7	-1,1	0,528	**0,000**	0,241	SMU,641	SMU,642	fw
igs0526	1,0	1,0	1,5	0,965	0,546	**0,000**	SMU,641	SMU,642	rc
igs0527	-1,5	1,0	**3,3**	**0,023**	0,821	**0,000**	SMU,642	SMU,643	fw
igs0527	-1,3	1,1	1,4	0,324	0,655	**0,037**	SMU,642	SMU,643	rc
igs0528	-1,1	1,2	1,0	0,180	0,127	0,629	SMU,643	SMU,644	fw
igs0528	-1,0	**3,5**	-1,2	0,865	**0,001**	0,345	SMU,643	SMU,644	rc
igs0530	-1,1	-1,2	1,2	0,097	0,076	**0,003**	SMU,645	SMU,646	fw
igs0530	1,1	**7,8**	-1,3	0,662	**0,000**	0,137	SMU,645	SMU,646	rc
igs0531	-1,0	1,6	-1,0	0,518	**0,000**	0,951	SMU,646	SMU,647	fw
igs0531	-1,5	**3,0**	-1,4	**0,023**	**0,007**	0,079	SMU,646	SMU,647	rc
igs0532	1,1	1,1	1,2	0,315	0,166	0,200	SMU,647	SMU,648	fw
igs0532	-1,4	-1,2	-1,3	0,531	0,757	0,587	SMU,647	SMU,648	rc
igs0533	1,4	1,4	-1,1	**0,000**	**0,002**	0,087	SMU,648	SMU,649	fw
igs0533	1,1	-1,1	-1,1	0,781	0,744	0,799	SMU,648	SMU,649	rc
igs0534	1,0	-1,2	1,0	0,266	0,060	0,733	SMU,649	SMU,650	fw
igs0534	-1,2	-1,5	-1,1	0,383	0,056	0,538	SMU,649	SMU,650	rc
igs0535	1,3	1,8	1,0	0,221	**0,001**	0,804	SMU,650	SMU,651c	fw
igs0535	1,0	-1,0	-1,1	0,925	0,961	0,551	SMU,650	SMU,651c	rc
igs0537	1,4	-1,2	-1,1	**0,003**	0,122	0,222	SMU,653c	SMU,654	fw
igs0537	1,2	-1,5	-1,4	0,073	**0,002**	0,076	SMU,653c	SMU,654	rc
igs0539	1,1	-1,0	1,1	0,081	0,854	0,508	SMU,657	SMU,658	fw
igs0539	1,2	-1,1	-1,3	**0,003**	0,278	**0,001**	SMU,657	SMU,658	rc
igs0540	1,2	1,2	-1,8	**0,032**	0,071	**0,003**	SMU,658	SMU,659	fw
igs0540	-1,4	-1,5	-1,1	0,061	**0,038**	0,471	SMU,658	SMU,659	rc
igs0541	1,4	-1,2	1,6	**0,002**	0,563	**0,001**	SMU,660	SMU,661	fw
igs0541	1,1	1,1	-1,1	0,386	0,153	0,443	SMU,660	SMU,661	rc
igs0542	-1,0	1,4	1,2	0,987	**0,001**	0,105	SMU,661	SMU,662	fw
igs0542	1,1	1,2	-1,3	0,682	0,482	0,445	SMU,661	SMU,662	rc
igs0543	1,2	1,9	1,5	0,332	**0,000**	0,171	SMU,662	SMU,663	fw
igs0543	1,2	-1,0	1,2	**0,000**	0,962	0,066	SMU,662	SMU,663	rc
igs0544	-1,3	-1,1	1,1	**0,004**	0,543	0,195	SMU,663	SMU,664	fw
igs0544	1,0	1,2	-1,0	0,965	0,104	0,720	SMU,663	SMU,664	rc
igs0545	-1,3	1,2	1,1	0,162	0,134	0,248	SMU,664	SMU,665	fw
igs0545	1,1	1,2	-1,2	**0,001**	**0,012**	**0,000**	SMU,664	SMU,665	rc
igs0546	-1,5	1,1	-1,1	**0,000**	0,657	0,686	SMU,665	SMU,666	fw
igs0546	-1,2	-1,3	1,4	0,168	0,116	0,106	SMU,665	SMU,666	rc
igs0547	-1,9	1,3	1,1	0,232	0,529	0,808	SMU,666	SMU,667	fw
igs0547	1,2	-1,3	-1,5	**0,042**	0,227	**0,013**	SMU,666	SMU,667	rc
igs0548	1,2	-1,3	1,2	0,375	0,090	0,222	SMU,667	SMU,668c	fw
igs0548	-1,1	1,0	-1,3	0,186	0,991	**0,032**	SMU,667	SMU,668c	rc
igs0549	1,0	-1,2	-1,0	0,982	0,444	0,897	SMU,668c	SMU,669c	fw
igs0549	-1,4	1,3	-1,0	**0,017**	0,067	0,942	SMU,668c	SMU,669c	rc
igs0550	1,2	-1,1	**2,2**	0,070	0,353	**0,000**	SMU,669c	SMU,670	fw
igs0550	1,3	-1,3	-1,2	0,231	0,248	0,233	SMU,669c	SMU,670	rc
igs0553	1,0	-1,1	1,0	0,983	0,587	0,932	SMU,672	SMU,673	fw
igs0553	-1,1	-1,2	1,0	0,439	0,377	0,970	SMU,672	SMU,673	rc
igs0554	1,2	-1,6	1,9	**0,002**	**0,009**	**0,000**	SMU,673	SMU,674	fw
igs0554	-1,1	-1,4	-1,3	0,807	0,540	0,695	SMU,673	SMU,674	rc
igs0556	1,1	-1,2	1,1	0,570	0,441	0,588	SMU,675	SMU,676	fw
igs0556	-1,6	-1,4	-1,4	0,383	0,582	0,535	SMU,675	SMU,676	rc
igs0557	1,0	-1,2	1,1	0,454	**0,019**	0,335	SMU,676	SMU,677	fw
igs0557	-1,3	1,1	-1,2	0,154	0,571	0,467	SMU,676	SMU,677	rc
igs0559	-4,2	1,5	-1,3	**0,000**	0,110	0,072	SMU,679	SMU,680	fw

igs0559	1,2	2,0	1,9	0,437	**0,041**	0,077	SMU,679	SMU,680	rc
igs0560	1,0	-1,0	1,3	0,398	0,874	**0,001**	SMU,680	SMU,681	fw
igs0560	1,2	-1,1	-1,3	0,112	0,082	**0,012**	SMU,680	SMU,681	rc
igs0561	-1,0	1,2	-1,1	0,921	0,308	0,397	SMU,681	SMU,682	fw
igs0561	1,0	-1,2	-1,6	0,794	0,179	**0,000**	SMU,681	SMU,682	rc
igs0564	1,2	1,0	-1,1	**0,025**	0,807	0,166	SMU,685	SMU,687c	fw
igs0564	-1,1	-1,1	-1,1	0,534	0,394	0,534	SMU,685	SMU,687c	rc
igs0565	1,7	1,0	1,5	**0,003**	0,729	**0,006**	SMU,687c	SMU,688	fw
igs0565	1,4	-1,0	1,4	**0,026**	0,924	0,059	SMU,687c	SMU,688	rc
igs0566	1,1	1,0	1,4	0,187	0,708	0,066	SMU,688	SMU,689	fw
igs0566	-1,1	-1,1	-1,2	0,357	0,673	0,154	SMU,688	SMU,689	rc
igs0568	1,3	2,0	-1,0	**0,001**	**0,001**	0,830	SMU,690	SMU,691	fw
igs0568	1,1	1,1	1,0	0,904	0,894	0,961	SMU,690	SMU,691	rc
igs0569	1,3	1,1	1,5	**0,012**	0,357	**0,000**	SMU,691	SMU,692	fw
igs0569	1,6	-1,2	-1,1	0,367	0,702	0,796	SMU,691	SMU,692	rc
igs0570	-1,1	1,9	**3,0**	0,650	**0,009**	**0,001**	SMU,694c	SMU,695	fw
igs0570	1,0	-1,0	1,3	0,928	0,984	0,472	SMU,694c	SMU,695	rc
igs0572	-1,1	-1,2	1,1	**0,012**	**0,000**	0,080	SMU,696	SMU,697	fw
igs0572	-1,0	-1,3	-1,0	0,921	0,617	0,974	SMU,696	SMU,697	rc
igs0573	1,1	1,3	-1,2	**0,017**	0,102	0,119	SMU,697	SMU,698	fw
igs0573	-1,4	-1,8	**2,7**	**0,001**	**0,001**	**0,000**	SMU,697	SMU,698	rc
igs0574	1,1	-1,3	1,5	0,415	0,058	**0,001**	SMU,698	SMU,699	fw
igs0574	1,3	-1,4	-1,2	0,262	0,119	0,381	SMU,698	SMU,699	rc
igs0575	1,2	-1,4	1,1	0,386	0,283	0,781	SMU,699	SMU,700c	fw
igs0575	-1,5	-1,9	1,1	0,389	0,236	0,845	SMU,699	SMU,700c	rc
igs0578	-1,9	1,6	**3,3**	**0,033**	0,271	**0,015**	SMU,703c	SMU,704c	fw
igs0578	1,8	1,5	-1,5	**0,001**	**0,042**	**0,000**	SMU,703c	SMU,704c	rc
igs0579	-1,2	-1,2	-1,8	**0,033**	0,064	**0,006**	SMU,704c	SMU,706c	fw
igs0579	1,3	1,1	1,4	**0,011**	0,292	**0,012**	SMU,704c	SMU,706c	rc
igs0580	1,2	1,2	-1,3	0,710	0,672	0,646	SMU,706c	SMU,707c	fw
igs0580	-1,3	1,3	1,1	**0,000**	**0,000**	0,434	SMU,706c	SMU,707c	rc
igs0581	1,2	1,2	1,3	0,099	0,252	0,053	SMU,707c	SMU,709	fw
igs0581	-1,3	1,7	-1,2	0,215	**0,007**	0,249	SMU,707c	SMU,709	rc
igs0582	-1,4	-1,1	1,3	**0,003**	0,888	0,326	SMU,711	SMU,712	fw
igs0582	-1,2	-1,5	-1,2	0,644	0,323	0,675	SMU,711	SMU,712	rc
igs0583	1,0	1,0	-1,0	0,855	0,815	0,678	SMU,712	SMU,713	fw
igs0583	-1,3	-1,6	-1,1	0,408	0,081	0,539	SMU,712	SMU,713	rc
igs0584	-1,1	1,4	-1,5	0,427	**0,010**	**0,000**	SMU,713	SMU,714	fw
igs0584	-1,4	-1,3	-1,7	0,557	0,702	0,477	SMU,713	SMU,714	rc
igs0585	1,1	-1,1	-1,5	0,297	0,613	**0,001**	SMU,714	SMU,715	fw
igs0585	-1,2	1,0	-1,0	0,549	0,970	0,999	SMU,714	SMU,715	rc
igs0586	-1,4	-1,3	1,2	0,151	0,249	0,248	SMU,715	SMU,716	fw
igs0586	1,1	-1,7	1,1	0,632	0,050	0,690	SMU,715	SMU,716	rc
igs0588	-1,1	1,1	-1,1	0,764	0,615	0,715	SMU,718c	SMU,719c	fw
igs0588	-1,3	-1,0	1,0	0,143	0,941	0,913	SMU,718c	SMU,719c	rc
igs0589	-1,5	1,0	-2,6	**0,021**	0,905	**0,000**	SMU,719c	SMU,720	fw
igs0589	**2,1**	-1,1	-1,2	**0,000**	0,795	0,380	SMU,719c	SMU,720	rc
igs0590	1,4	1,0	1,7	**0,000**	0,658	**0,002**	SMU,720	SMU,721	fw
igs0590	-1,2	-1,6	-1,4	0,082	**0,001**	**0,000**	SMU,720	SMU,721	rc
igs0591	-1,0	-1,1	1,6	0,944	0,512	**0,001**	SMU,721	SMU,722	fw
igs0591	-1,4	-1,2	-2,1	**0,018**	0,112	**0,000**	SMU,721	SMU,722	rc
igs0592	-1,0	1,1	-1,2	0,633	0,078	**0,018**	SMU,722	SMU,723	fw
igs0592	1,3	1,4	1,0	0,587	0,436	0,969	SMU,722	SMU,723	rc
igs0593	-1,2	-1,1	1,3	**0,028**	0,507	**0,017**	SMU,723	SMU,724	fw
igs0593	1,3	1,1	-1,0	0,604	0,720	0,983	SMU,723	SMU,724	rc
igs0594	-1,1	1,1	-1,2	0,413	0,565	0,135	SMU,724	SMU,725c	fw
igs0594	1,0	-1,1	1,3	0,691	0,389	**0,004**	SMU,724	SMU,725c	rc
igs0595	1,2	1,2	1,2	**0,000**	**0,000**	0,054	SMU,725c	SMU,727	fw
igs0595	1,1	1,3	-1,3	0,768	0,299	0,156	SMU,725c	SMU,727	rc
igs0596	1,0	-1,7	-1,2	0,790	**0,000**	0,249	SMU,728	SMU,730	fw
igs0596	1,0	1,1	1,3	0,975	0,518	0,109	SMU,728	SMU,730	rc
igs0598	1,0	1,0	1,1	0,730	0,398	0,301	SMU,732	SMU,734	fw
igs0598	-1,3	-1,1	-1,2	0,072	0,491	0,247	SMU,732	SMU,734	rc
igs0599	1,3	-1,3	1,4	**0,001**	0,124	**0,001**	SMU,734	SMU,735	fw
igs0599	1,2	-1,2	-1,3	0,254	0,382	0,138	SMU,734	SMU,735	rc
igs0600	1,0	-1,1	-1,3	0,939	0,599	0,384	SMU,735	SMU,737	fw

igs0600	-2,5	-1,4	-1,8	**0,000**	0,125	0,157	SMU,735	SMU,737	rc
igs0601	1,1	1,1	1,1	0,158	0,091	0,302	SMU,737	SMU,738	fw
igs0601	-1,1	1,1	-1,3	0,701	0,426	0,114	SMU,737	SMU,738	rc
igs0602	1,0	-1,1	1,0	0,988	0,533	0,889	SMU,738	SMU,739c	fw
igs0602	1,2	1,0	-1,2	0,441	0,910	0,197	SMU,738	SMU,739c	rc
igs0603	1,0	1,1	-1,0	0,571	0,386	0,859	SMU,739c	SMU,741	fw
igs0603	1,3	-1,0	-1,1	0,051	0,827	0,567	SMU,739c	SMU,741	rc
igs0604	1,2	1,1	1,4	0,544	0,559	0,280	SMU,741	SMU,742	fw
igs0604	1,3	-1,6	**2,4**	**0,019**	**0,028**	**0,000**	SMU,741	SMU,742	rc
igs0606	-1,1	-1,3	-1,1	0,354	**0,026**	0,678	SMU,744	SMU,745	fw
igs0606	-1,3	-1,1	1,0	0,560	0,783	0,965	SMU,744	SMU,745	rc
igs0607	1,2	-1,3	1,0	0,655	0,386	0,991	SMU,745	SMU,746c	fw
igs0607	-1,0	1,6	1,3	0,873	**0,001**	0,067	SMU,745	SMU,746c	rc
igs0608	-1,1	-1,1	-1,1	0,736	0,648	0,498	SMU,748	SMU,750c	fw
igs0608	1,0	-1,0	1,0	0,481	0,856	0,489	SMU,748	SMU,750c	rc
igs0609	1,1	1,1	-1,4	0,377	0,200	**0,002**	SMU,750c	SMU,751	fw
igs0609	1,2	-1,0	1,8	0,124	0,846	**0,000**	SMU,750c	SMU,751	rc
igs0610	-1,0	-1,7	**2,2**	0,939	**0,000**	**0,000**	SMU,752	SMU,753	fw
igs0610	1,3	-1,8	1,1	0,683	0,499	0,923	SMU,752	SMU,753	rc
igs0611	-1,1	-1,3	-1,2	0,669	0,157	0,126	SMU,753	SMU,754	fw
igs0611	1,0	1,6	-1,3	0,880	0,117	0,320	SMU,753	SMU,754	rc
igs0612	-1,4	-1,0	1,5	**0,001**	0,177	**0,004**	SMU,755	SMU,756	fw
igs0612	1,6	1,0	-1,8	**0,003**	0,400	**0,000**	SMU,755	SMU,756	rc
igs0613	-1,2	-1,1	1,3	0,575	0,797	0,490	SMU,757	SMU,758c	fw
igs0613	1,2	-1,4	-1,1	0,461	0,193	0,657	SMU,757	SMU,758c	rc
igs0614	1,6	-1,7	1,1	0,164	0,155	0,846	SMU,758c	SMU,759	fw
igs0614	-1,1	-1,9	1,2	0,673	**0,000**	0,376	SMU,758c	SMU,759	rc
igs0615	-1,0	-1,1	-1,2	0,526	**0,000**	**0,000**	SMU,759	SMU,761	fw
igs0615	1,1	-1,1	1,3	0,796	0,656	0,412	SMU,759	SMU,761	rc
igs0616	1,2	-1,1	1,0	0,149	0,748	0,969	SMU,761	SMU,764	fw
igs0616	-1,1	-1,1	1,2	0,794	0,890	0,789	SMU,761	SMU,764	rc
igs0618	-1,6	-1,1	1,1	**0,000**	0,306	0,488	SMU,765	SMU,766	fw
igs0618	1,3	-1,2	-1,2	0,645	0,768	0,776	SMU,765	SMU,766	rc
igs0619	-1,0	-1,6	-1,2	0,997	0,059	0,193	SMU,767	SMU,768c	fw
igs0619	1,0	-1,2	1,2	0,842	0,346	0,241	SMU,767	SMU,768c	rc
igs0620	1,1	-1,8	-1,2	0,499	**0,002**	0,082	SMU,768c	SMU,769	fw
igs0620	1,3	1,4	-2,2	0,365	0,343	**0,005**	SMU,768c	SMU,769	rc
igs0621	-1,7	-1,5	-1,1	**0,020**	0,070	0,674	SMU,769	SMU,770c	fw
igs0621	1,2	**10,5**	1,5	0,716	**0,000**	0,332	SMU,769	SMU,770c	rc
igs0622	-1,3	-1,2	1,1	**0,022**	0,086	0,309	SMU,770c	SMU,771c	fw
igs0622	1,1	**3,9**	-1,3	0,103	**0,000**	**0,010**	SMU,770c	SMU,771c	rc
igs0623	-1,2	1,5	-1,3	0,173	**0,001**	0,070	SMU,771c	SMU,772	fw
igs0623	1,4	**2,3**	-1,0	**0,001**	**0,000**	0,728	SMU,771c	SMU,772	rc
igs0624	-1,1	-1,0	1,4	0,690	0,927	0,289	SMU,772	SMU,773c	fw
igs0624	1,2	1,1	-1,0	0,341	0,665	0,981	SMU,772	SMU,773c	rc
igs0625	-1,1	-1,1	1,3	0,245	0,292	0,055	SMU,773c	SMU,774	fw
igs0625	1,3	1,3	1,2	**0,007**	**0,003**	0,048	SMU,773c	SMU,774	rc
igs0626	-1,1	-1,0	1,3	0,725	0,925	0,324	SMU,774	SMU,775c	fw
igs0626	-1,2	-1,2	-1,4	0,600	0,657	0,307	SMU,774	SMU,775c	rc
igs0627	-1,1	-1,1	1,0	0,674	0,693	0,998	SMU,775c	SMU,776	fw
igs0627	-1,3	1,1	1,0	0,449	0,747	0,958	SMU,775c	SMU,776	rc
igs0631	-1,9	-1,1	1,4	**0,000**	0,476	**0,000**	SMU,780	SMU,781	fw
igs0631	-1,1	-1,1	-1,3	0,660	0,802	0,271	SMU,780	SMU,781	rc
igs0633	1,0	1,5	1,0	0,606	**0,007**	0,858	SMU,782	SMU,784	fw
igs0633	1,1	-1,4	1,3	0,908	0,587	0,639	SMU,782	SMU,784	rc
igs0634	-1,6	1,4	1,4	**0,026**	**0,000**	**0,008**	SMU,786	SMU,787	fw
igs0634	1,1	-1,3	-1,2	0,727	0,123	0,118	SMU,786	SMU,787	rc
igs0635	-1,3	-1,1	1,0	**0,000**	0,056	0,614	SMU,787	SMU,788	fw
igs0635	-1,2	-1,1	-1,4	0,401	0,725	0,075	SMU,787	SMU,788	rc
igs0636	-1,2	-1,2	1,0	**0,000**	**0,019**	0,619	SMU,788	SMU,789	fw
igs0636	-1,1	-1,1	-1,5	0,605	0,581	0,064	SMU,788	SMU,789	rc
igs0637	-1,0	1,1	1,1	0,845	0,316	0,718	SMU,789	SMU,790	fw
igs0637	1,1	-1,0	-1,1	0,495	0,808	0,497	SMU,789	SMU,790	rc
igs0638	-1,0	-1,1	1,1	0,283	0,232	0,157	SMU,791c	SMU,793	fw
igs0638	1,4	-1,1	1,0	0,146	0,611	0,939	SMU,791c	SMU,793	rc
igs0639	1,1	-1,5	1,3	**0,005**	**0,001**	**0,000**	SMU,793	SMU,794	fw

igs0639	1,0	-1,1	-2,2	0,905	0,260	**0,000**	SMU,793	SMU,794	rc
igs0640	-1,1	1,1	1,6	**0,023**	0,154	**0,003**	SMU,794	SMU,795	fw
igs0640	-1,2	-1,0	1,0	0,348	0,676	0,864	SMU,794	SMU,795	rc
igs0641	-2,3	-1,8	1,1	**0,000**	**0,001**	0,663	SMU,795	SMU,796	fw
igs0641	-1,1	-1,1	-1,2	0,654	0,804	0,363	SMU,795	SMU,796	rc
igs0642	1,4	-1,0	**2,7**	**0,001**	0,401	**0,000**	SMU,796	SMU,797	fw
igs0642	**2,7**	1,1	1,3	**0,000**	0,466	0,133	SMU,796	SMU,797	rc
igs0643	1,1	1,2	-1,3	**0,001**	**0,035**	**0,001**	SMU,797	SMU,798c	fw
igs0643	-1,2	-1,9	-1,6	0,362	**0,004**	**0,010**	SMU,797	SMU,798c	rc
igs0644	-1,0	1,1	-1,3	0,715	0,137	**0,014**	SMU,798c	SMU,799c	fw
igs0644	-1,2	-1,2	1,1	0,146	0,145	0,373	SMU,798c	SMU,799c	rc
igs0645	-1,5	-1,1	1,4	**0,000**	0,093	**0,001**	SMU,799c	SMU,800	fw
igs0645	1,2	-1,1	-1,6	0,428	0,564	0,155	SMU,799c	SMU,800	rc
igs0646	-1,1	1,1	1,0	0,521	0,181	0,307	SMU,800	SMU,801	fw
igs0646	-1,1	-1,1	-2,1	0,680	0,652	**0,026**	SMU,800	SMU,801	rc
igs0647	1,0	1,1	1,2	0,665	0,217	0,059	SMU,801	SMU,802	fw
igs0647	-1,0	-1,4	-1,0	0,980	0,063	0,990	SMU,801	SMU,802	rc
igs0648	-1,1	-1,3	1,0	**0,008**	**0,020**	0,824	SMU,802	SMU,803c	fw
igs0648	-1,2	-1,1	-1,0	0,713	0,853	0,936	SMU,802	SMU,803c	rc
igs0649	-1,1	1,0	1,0	0,523	0,957	0,938	SMU,803c	SMU,804	fw
igs0649	-1,0	1,1	1,3	0,914	0,532	**0,039**	SMU,803c	SMU,804	rc
igs0650	-1,4	-1,4	1,5	0,161	0,236	0,161	SMU,804	SMU,805c	fw
igs0650	-1,0	1,1	1,3	0,900	0,474	0,060	SMU,804	SMU,805c	rc
igs0651	1,1	1,3	**2,1**	0,829	0,247	**0,021**	SMU,806c	SMU,807	fw
igs0651	-1,4	1,4	1,2	0,083	**0,047**	0,307	SMU,806c	SMU,807	rc
igs0652	1,3	-1,0	-1,2	**0,000**	0,783	0,141	SMU,807	SMU,809	fw
igs0652	1,2	-1,1	-1,6	0,653	0,875	0,278	SMU,807	SMU,809	rc
igs0653	-1,0	1,2	-1,0	0,932	**0,011**	0,735	SMU,809	SMU,811	fw
igs0653	1,3	-1,0	-1,3	**0,009**	0,663	**0,032**	SMU,809	SMU,811	rc
igs0654	-1,0	-1,4	-1,5	0,636	**0,000**	**0,001**	SMU,812	SMU,813	fw
igs0654	1,0	-1,2	1,2	0,998	0,324	0,282	SMU,812	SMU,813	rc
igs0655	1,0	1,0	-1,2	0,950	0,989	0,647	SMU,813	SMU,814	fw
igs0655	-1,1	1,3	-1,3	0,535	**0,006**	0,121	SMU,813	SMU,814	rc
igs0656	-1,2	-2,2	-1,2	0,301	**0,000**	0,211	SMU,814	SMU,815	fw
igs0656	1,2	1,0	**2,5**	0,311	0,871	**0,000**	SMU,814	SMU,815	rc
igs0657	1,0	-1,0	-1,4	0,858	0,898	**0,000**	SMU,815	SMU,816	fw
igs0657	-1,0	-1,4	-1,0	0,961	0,233	0,945	SMU,815	SMU,816	rc
igs0658	1,0	-1,2	1,0	0,542	0,146	0,840	SMU,816	SMU,817	fw
igs0658	1,1	-1,1	-2,9	0,051	**0,041**	**0,014**	SMU,816	SMU,817	rc
igs0659	-1,2	-1,0	1,6	0,084	0,877	**0,000**	SMU,817	SMU,818	fw
igs0659	1,3	-1,1	1,5	0,597	0,811	0,454	SMU,817	SMU,818	rc
igs0660	1,5	-1,4	1,4	0,556	0,595	0,675	SMU,818	SMU,819	fw
igs0660	-1,1	1,0	-1,1	0,670	0,904	0,745	SMU,818	SMU,819	rc
igs0661	-1,5	1,0	1,1	0,160	0,967	0,856	SMU,819	SMU,820	fw
igs0661	-1,1	-1,2	1,2	0,755	0,685	0,623	SMU,819	SMU,820	rc
igs0662	1,0	-1,2	-1,1	0,693	0,228	0,627	SMU,820	SMU,821	fw
igs0662	1,3	-1,3	1,4	0,631	0,661	0,506	SMU,820	SMU,821	rc
igs0665	-1,2	-1,3	1,4	0,138	**0,013**	0,049	SMU,823	SMU,824	fw
igs0665	1,6	1,2	-1,0	0,247	0,488	0,901	SMU,823	SMU,824	rc
igs0666	-1,1	1,1	1,4	0,218	0,633	0,069	SMU,824	SMU,825	fw
igs0666	1,4	1,2	-1,2	0,474	0,727	0,691	SMU,824	SMU,825	rc
igs0668	**4,2**	-1,0	**3,0**	**0,000**	0,928	**0,000**	SMU,831	SMU,832	fw
igs0668	-1,4	-1,7	-1,2	0,294	0,114	0,414	SMU,831	SMU,832	rc
igs0670	1,3	-1,3	1,4	**0,000**	**0,018**	**0,028**	SMU,833	SMU,834	fw
igs0670	-1,4	-1,5	1,0	0,275	0,317	0,835	SMU,833	SMU,834	rc
igs0671	1,2	1,3	-1,2	**0,000**	**0,001**	**0,000**	SMU,834	SMU,835	fw
igs0671	1,1	1,3	-1,5	0,819	0,521	0,419	SMU,834	SMU,835	rc
igs0672	-1,0	-1,2	1,5	0,727	0,120	**0,000**	SMU,835	SMU,836	fw
igs0672	1,8	1,7	1,1	0,131	0,151	0,845	SMU,835	SMU,836	rc
igs0673	1,1	1,1	-1,1	**0,000**	0,064	0,514	SMU,836	SMU,837	fw
igs0673	1,6	**27,6**	1,1	0,166	**0,000**	0,841	SMU,836	SMU,837	rc
igs0674	-1,0	-1,2	1,4	0,729	0,059	0,047	SMU,837	SMU,838	fw
igs0674	-1,1	**8,3**	1,5	0,860	**0,000**	0,373	SMU,837	SMU,838	rc
igs0676	1,1	1,0	1,1	0,491	0,969	0,581	SMU,839	SMU,840c	fw
igs0676	1,1	1,6	-1,4	0,497	**0,000**	**0,017**	SMU,839	SMU,840c	rc
igs0677	1,2	1,2	-1,1	0,558	0,644	0,838	SMU,840c	SMU,841	fw

igs0677	1,8	1,2	**2,1**	**0,003**	0,388	**0,001**	SMU,840c	SMU,841	rc
igs0678	1,1	1,1	1,2	0,527	0,654	0,126	SMU,841	SMU,842	fw
igs0678	-1,0	-1,0	-1,5	0,942	0,964	0,503	SMU,841	SMU,842	rc
igs0679	1,2	1,5	1,1	**0,014**	**0,000**	0,374	SMU,842	SMU,843	fw
igs0679	1,2	-1,3	1,3	0,563	0,513	0,494	SMU,842	SMU,843	rc
igs0680	1,9	-1,2	1,4	**0,000**	0,051	**0,000**	SMU,843	SMU,844	fw
igs0680	1,4	-1,2	-1,3	**0,011**	0,059	**0,011**	SMU,843	SMU,844	rc
igs0681	1,0	1,5	**2,1**	0,872	**0,000**	**0,000**	SMU,844	SMU,845	fw
igs0681	1,4	1,2	-1,7	**0,003**	0,084	**0,000**	SMU,844	SMU,845	rc
igs0682	-1,8	-2,0	-1,0	**0,000**	**0,000**	0,747	SMU,845	SMU,847c	fw
igs0682	1,1	1,0	1,7	0,797	0,952	0,347	SMU,845	SMU,847c	rc
igs0684	-1,0	-1,3	1,5	0,687	0,061	**0,010**	SMU,849	SMU,850	fw
igs0684	-1,6	-1,5	1,0	0,245	0,299	0,971	SMU,849	SMU,850	rc
igs0685	-1,0	-1,0	1,1	0,872	0,606	0,151	SMU,851	SMU,852	fw
igs0685	-1,0	-1,2	-1,2	0,971	0,059	0,107	SMU,851	SMU,852	rc
igs0687	1,3	-1,3	1,4	**0,000**	**0,000**	**0,000**	SMU,854	SMU,855	fw
igs0687	1,0	-1,6	-1,1	0,954	**0,047**	0,706	SMU,854	SMU,855	rc
igs0688	-1,0	-1,2	1,2	0,742	**0,001**	0,300	SMU,855	SMU,856	fw
igs0688	1,0	-1,0	-1,3	0,939	0,921	0,506	SMU,855	SMU,856	rc
igs0690	1,3	-1,2	1,6	**0,000**	**0,035**	**0,000**	SMU,858	SMU,859	fw
igs0690	-1,8	-1,6	-1,8	**0,000**	**0,002**	**0,000**	SMU,858	SMU,859	rc
igs0691	-1,3	-1,2	1,1	0,125	0,305	0,515	SMU,859	SMU,860	fw
igs0691	1,1	1,0	-1,4	0,679	0,835	**0,016**	SMU,859	SMU,860	rc
igs0692	-1,3	-1,8	1,0	**0,000**	**0,000**	0,886	SMU,860	SMU,862	fw
igs0692	1,3	-1,1	1,0	0,383	0,765	0,937	SMU,860	SMU,862	rc
igs0695	1,0	1,2	1,2	0,891	**0,011**	0,058	SMU,864	SMU,865	fw
igs0695	-1,5	1,0	-1,1	0,100	0,927	0,700	SMU,864	SMU,865	rc
igs0697	1,4	1,7	-1,6	**0,000**	**0,000**	**0,000**	SMU,866	SMU,867	fw
igs0697	-1,3	-1,9	-2,1	0,442	0,099	0,083	SMU,866	SMU,867	rc
igs0698	-1,0	-1,0	-1,1	0,908	0,849	0,334	SMU,868	SMU,869	fw
igs0698	-1,1	-1,2	-1,1	0,580	0,498	0,541	SMU,868	SMU,869	rc
igs0699	1,2	-1,3	1,6	0,065	0,096	0,076	SMU,869	SMU,870	fw
igs0699	-1,2	-1,2	**2,1**	0,662	0,575	**0,008**	SMU,869	SMU,870	rc
igs0700	1,2	1,4	**2,2**	**0,037**	**0,002**	**0,000**	SMU,872	SMU,873	fw
igs0700	-1,0	-1,3	1,6	0,848	0,318	0,110	SMU,872	SMU,873	rc
igs0702	1,2	-1,6	1,4	**0,007**	**0,041**	**0,031**	SMU,874	SMU,875c	fw
igs0702	-1,2	1,1	1,1	0,093	0,622	0,334	SMU,874	SMU,875c	rc
igs0703	-1,2	1,0	1,1	**0,000**	0,586	0,165	SMU,875c	SMU,876	fw
igs0703	-1,0	**2,2**	-1,1	0,961	**0,000**	0,281	SMU,875c	SMU,876	rc
igs0704	-1,7	1,1	1,9	**0,000**	0,189	**0,000**	SMU,876	SMU,877	fw
igs0704	-1,0	1,5	-1,2	0,924	0,452	0,730	SMU,876	SMU,877	rc
igs0708	-1,1	-1,1	-1,0	**0,010**	0,386	0,534	SMU,880	SMU,881	fw
igs0708	-1,0	1,2	1,3	0,857	0,226	**0,028**	SMU,880	SMU,881	rc
igs0710	-1,2	1,4	-1,9	0,061	**0,004**	**0,000**	SMU,882	SMU,883	fw
igs0710	-1,4	1,2	-1,1	0,374	0,698	0,848	SMU,882	SMU,883	rc
igs0711	-1,2	1,2	-1,1	0,175	0,255	0,379	SMU,883	SMU,885	fw
igs0711	1,3	1,3	1,1	0,442	0,587	0,771	SMU,883	SMU,885	rc
igs0712	-1,5	1,9	**2,1**	**0,036**	**0,006**	**0,001**	SMU,885	SMU,886	fw
igs0712	-1,6	-1,0	-3,5	**0,015**	0,971	**0,000**	SMU,885	SMU,886	rc
igs0714	-1,0	-1,2	-1,1	0,592	**0,001**	0,072	SMU,887	SMU,888	fw
igs0714	1,3	1,1	-1,5	**0,000**	0,236	**0,000**	SMU,887	SMU,888	rc
igs0715	1,2	1,4	1,4	**0,006**	**0,001**	**0,005**	SMU,888	SMU,889	fw
igs0715	-1,0	1,0	-1,3	0,965	0,932	0,245	SMU,888	SMU,889	rc
igs0716	-1,0	-1,0	-1,1	0,671	0,770	0,333	SMU,889	SMU,890	fw
igs0716	-1,2	1,0	1,4	0,576	0,900	0,287	SMU,889	SMU,890	rc
igs0717	-1,3	-1,7	1,4	**0,000**	**0,000**	**0,000**	SMU,890	SMU,891	fw
igs0717	-1,0	1,2	1,3	0,827	0,590	0,405	SMU,890	SMU,891	rc
igs0718	1,4	-1,8	-1,0	**0,000**	**0,001**	0,402	SMU,892	SMU,893	fw
igs0718	1,1	-1,5	1,8	0,186	**0,013**	**0,002**	SMU,892	SMU,893	rc
igs0719	1,5	-1,3	1,3	0,112	0,147	**0,001**	SMU,893	SMU,895	fw
igs0719	1,0	-5,0	1,5	0,951	**0,000**	**0,000**	SMU,893	SMU,895	rc
igs0721	-1,3	-1,2	-1,0	**0,000**	0,148	0,901	SMU,897	SMU,898	fw
igs0721	-1,1	-1,1	1,6	0,868	0,674	0,129	SMU,897	SMU,898	rc
igs0725	-1,1	-1,4	-1,1	**0,009**	**0,000**	0,320	SMU,902	SMU,905	fw
igs0725	1,0	1,0	-1,3	0,959	0,954	0,150	SMU,902	SMU,905	rc
igs0727	-1,3	-1,0	1,0	**0,020**	0,651	0,778	SMU,906	SMU,909	fw

igs0727	-1,3	1,4	-1,9	0,121	0,282	**0,007**	SMU,906	SMU,909	rc
igs0728	1,2	1,3	-1,1	**0,022**	**0,039**	0,456	SMU,909	SMU,910	fw
igs0728	-1,0	-1,2	-1,1	0,947	0,477	0,745	SMU,909	SMU,910	rc
igs0729	-1,3	1,2	-1,2	0,286	0,620	0,628	SMU,910	SMU,911c	fw
igs0729	-1,1	1,1	-1,0	0,707	0,838	0,948	SMU,910	SMU,911c	rc
igs0730	-1,0	1,2	1,3	0,882	0,058	**0,005**	SMU,911c	SMU,913	fw
igs0730	1,1	-1,0	-1,2	0,568	0,980	0,487	SMU,911c	SMU,913	rc
igs0731	1,5	-3,0	-1,2	**0,004**	**0,000**	0,294	SMU,913	SMU,914c	fw
igs0731	-1,1	-1,6	1,4	0,828	0,104	0,362	SMU,913	SMU,914c	rc
igs0732	-1,1	-1,3	1,3	0,559	0,284	0,129	SMU,914c	SMU,915c	fw
igs0732	-1,3	-1,1	-1,3	0,263	0,545	0,135	SMU,914c	SMU,915c	rc
igs0734	1,2	-1,2	-1,1	0,438	0,476	0,658	SMU,919c	SMU,921	fw
igs0734	-1,0	1,1	-1,0	0,738	0,396	0,949	SMU,919c	SMU,921	rc
igs0736	1,0	-1,2	1,2	0,792	**0,030**	0,448	SMU,923	SMU,924	fw
igs0736	1,4	-1,1	-1,1	0,290	0,719	0,644	SMU,923	SMU,924	rc
igs0737	-1,5	1,0	1,2	**0,000**	0,887	0,204	SMU,924	SMU,925	fw
igs0737	1,0	-1,1	1,3	0,833	0,653	0,228	SMU,924	SMU,925	rc
igs0738	-1,3	-1,2	-1,1	**0,044**	**0,043**	0,253	SMU,925	SMU,926	fw
igs0738	**3,6**	**9,6**	**5,9**	**0,000**	**0,000**	**0,000**	SMU,925	SMU,926	rc
igs0739	1,4	1,5	-1,4	0,581	0,508	0,593	SMU,928	SMU,929c	fw
igs0739	**4,3**	-1,1	**3,7**	**0,003**	0,815	**0,002**	SMU,928	SMU,929c	rc
igs0740	-1,3	1,4	-1,4	0,452	0,320	0,291	SMU,929c	SMU,930c	fw
igs0740	1,2	-1,3	-1,1	**0,000**	**0,024**	0,781	SMU,929c	SMU,930c	rc
igs0741	-1,4	1,0	1,1	**0,023**	0,935	0,440	SMU,930c	SMU,932	fw
igs0741	-1,0	1,2	-1,1	0,883	0,568	0,630	SMU,930c	SMU,932	rc
igs0746	1,6	-1,0	-1,2	**0,000**	0,640	0,170	SMU,936	SMU,937	fw
igs0746	1,1	1,1	1,3	0,755	0,659	0,356	SMU,936	SMU,937	rc
igs0749	-1,3	1,2	1,8	0,580	0,746	0,312	SMU,939	SMU,940c	fw
igs0749	-1,2	1,5	-1,8	0,692	0,271	0,064	SMU,939	SMU,940c	rc
igs0750	1,2	1,0	1,2	0,736	0,969	0,799	SMU,941c	SMU,942	fw
igs0750	-1,1	-1,4	1,1	0,327	**0,015**	0,502	SMU,941c	SMU,942	rc
igs0751	1,5	1,4	-1,1	0,089	0,396	0,597	SMU,943c	SMU,944	fw
igs0751	1,0	-1,4	-1,2	0,896	0,180	0,318	SMU,943c	SMU,944	rc
igs0752	1,3	-1,9	1,0	**0,001**	**0,000**	0,948	SMU,944	SMU,946	fw
igs0752	-1,2	1,2	1,9	0,645	0,446	**0,012**	SMU,944	SMU,946	rc
igs0753	**2,1**	-2,1	1,7	**0,000**	**0,000**	**0,002**	SMU,946	SMU,947	fw
igs0753	1,4	2,0	1,4	0,317	**0,031**	0,321	SMU,946	SMU,947	rc
igs0757	-1,1	-1,2	1,0	0,263	**0,004**	0,852	SMU,950	SMU,951	fw
igs0757	1,1	-1,2	-1,2	0,714	0,531	0,441	SMU,950	SMU,951	rc
igs0759	-1,5	-1,1	-1,1	**0,036**	0,235	0,614	SMU,952	SMU,953c	fw
igs0759	1,0	-1,1	-1,6	0,901	0,566	**0,002**	SMU,952	SMU,953c	rc
igs0760	1,5	1,1	1,2	0,175	0,401	0,352	SMU,953c	SMU,954	fw
igs0760	1,2	1,0	**2,1**	0,376	0,974	**0,000**	SMU,953c	SMU,954	rc
igs0761	1,2	-1,2	-1,6	0,818	0,707	0,406	SMU,955	SMU,956	fw
igs0761	-1,1	-1,6	-2,1	0,251	0,131	**0,005**	SMU,955	SMU,956	rc
igs0762	-1,6	1,1	1,3	**0,007**	0,478	0,053	SMU,956	SMU,957	fw
igs0762	-1,5	-1,1	1,5	0,472	0,832	0,546	SMU,956	SMU,957	rc
igs0764	-1,2	1,1	-1,1	0,089	0,509	0,541	SMU,960	SMU,961	fw
igs0764	-1,3	-1,1	1,3	0,545	0,912	0,581	SMU,960	SMU,961	rc
igs0766	-1,0	1,1	-1,0	0,846	0,651	0,994	SMU,962	SMU,963c	fw
igs0766	1,0	-1,1	-1,1	0,883	0,300	0,595	SMU,962	SMU,963c	rc
igs0767	1,0	1,3	-1,2	0,861	**0,009**	0,061	SMU,963c	SMU,965	fw
igs0767	-1,1	-1,3	-1,2	0,713	0,572	0,590	SMU,963c	SMU,965	rc
igs0769	1,4	-1,1	-1,2	**0,004**	0,698	0,066	SMU,966	SMU,967	fw
igs0769	1,1	-1,1	-1,3	0,566	0,582	0,217	SMU,966	SMU,967	rc
igs0773	1,0	1,6	-1,1	0,926	0,167	0,854	SMU,971	SMU,972	fw
igs0773	-1,3	-1,6	-1,2	0,322	**0,028**	0,486	SMU,971	SMU,972	rc
igs0775	-1,4	-1,1	-1,2	**0,006**	0,509	0,470	SMU,976	SMU,977	fw
igs0775	-1,6	1,1	-1,1	0,168	0,581	0,730	SMU,976	SMU,977	rc
igs0776	-1,3	-1,2	-1,1	**0,000**	0,060	0,522	SMU,977	SMU,980	fw
igs0776	1,0	-1,1	-1,1	0,971	0,700	0,746	SMU,977	SMU,980	rc
igs0777	-1,2	1,0	1,2	0,126	0,945	0,262	SMU,980	SMU,981	fw
igs0777	1,1	1,1	-1,6	0,741	0,844	**0,031**	SMU,980	SMU,981	rc
igs0778	1,3	1,1	1,4	**0,000**	0,095	**0,017**	SMU,981	SMU,982	fw
igs0778	1,3	1,2	1,2	**0,006**	0,063	**0,002**	SMU,981	SMU,982	rc
igs0779	-1,2	-1,1	1,0	0,425	0,537	0,827	SMU,982	SMU,983	fw

igs0779	1,1	-1,1	-1,1	0,284	0,495	0,571	SMU,982	SMU,983	rc
igs0780	1,5	1,0	1,6	**0,000**	0,927	**0,004**	SMU,983	SMU,984	fw
igs0780	1,0	1,3	1,0	0,896	**0,024**	0,859	SMU,983	SMU,984	rc
igs0781	1,5	-1,5	-1,5	**0,000**	**0,000**	0,066	SMU,984	SMU,985	fw
igs0781	-1,4	-1,1	-1,5	0,099	0,358	0,135	SMU,984	SMU,985	rc
igs0782	-1,5	-1,3	-1,1	**0,000**	0,169	0,560	SMU,985	SMU,986c	fw
igs0782	1,1	-1,2	-1,5	0,806	0,544	0,213	SMU,985	SMU,986c	rc
igs0783	1,2	1,2	1,7	0,122	0,278	**0,011**	SMU,986c	SMU,987	fw
igs0783	1,6	-2,2	**2,5**	**0,005**	**0,000**	**0,000**	SMU,986c	SMU,987	rc
igs0784	1,4	-1,2	-1,1	**0,000**	**0,025**	0,728	SMU,987	SMU,988	fw
igs0784	-1,1	-1,7	-1,1	0,614	**0,009**	0,766	SMU,987	SMU,988	rc
igs0785	-1,1	1,0	1,1	0,553	0,628	0,604	SMU,988	SMU,989	fw
igs0785	1,1	-1,0	1,7	0,924	0,990	0,367	SMU,988	SMU,989	rc
igs0786	-1,6	-1,1	1,2	**0,000**	0,382	**0,023**	SMU,989	SMU,990	fw
igs0786	1,2	1,0	-1,9	0,474	0,975	**0,015**	SMU,989	SMU,990	rc
igs0787	-1,2	1,2	1,3	**0,008**	0,056	**0,000**	SMU,990	SMU,991	fw
igs0787	-1,3	-1,1	-1,5	0,518	0,635	0,251	SMU,990	SMU,991	rc
igs0788	-1,2	-1,3	-1,1	**0,021**	**0,031**	0,191	SMU,991	SMU,992	fw
igs0788	1,3	-1,2	-1,0	0,569	0,738	0,966	SMU,991	SMU,992	rc
igs0789	1,1	1,2	1,3	**0,014**	**0,035**	0,102	SMU,992	SMU,993	fw
igs0789	-1,5	-1,2	1,3	**0,000**	0,084	**0,001**	SMU,992	SMU,993	rc
igs0790	1,2	-1,1	-1,7	0,149	0,534	**0,014**	SMU,994	SMU,995	fw
igs0790	1,1	-1,2	-1,2	0,877	0,733	0,795	SMU,994	SMU,995	rc
igs0791	-1,8	-1,9	1,8	**0,000**	**0,000**	**0,001**	SMU,997	SMU,998	fw
igs0791	-1,5	-1,7	-1,0	**0,002**	**0,001**	0,785	SMU,997	SMU,998	rc
igs0792	-1,3	1,2	-1,5	**0,036**	0,518	**0,015**	SMU,998	SMU,999	fw
igs0792	1,1	1,0	1,1	0,772	0,814	0,208	SMU,998	SMU,999	rc
igs0793	1,4	1,5	-1,2	**0,001**	**0,002**	**0,025**	SMU,1000	SMU,1001	fw
igs0793	-1,1	**3,2**	1,5	0,518	**0,000**	0,056	SMU,1000	SMU,1001	rc
igs0794	-2,9	-1,5	**2,4**	**0,000**	0,199	**0,000**	SMU,1001	SMU,1002	fw
igs0794	1,1	**4,1**	1,3	0,913	**0,029**	0,712	SMU,1001	SMU,1002	rc
igs0795	1,1	1,0	-1,1	0,508	0,987	0,683	SMU,1002	SMU,1003	fw
igs0795	1,1	**2,4**	-1,1	0,556	**0,001**	0,760	SMU,1002	SMU,1003	rc
igs0796	1,3	-1,1	1,1	**0,000**	0,252	0,233	SMU,1003	SMU,1004	fw
igs0796	1,0	**2,8**	1,2	0,903	**0,012**	0,560	SMU,1003	SMU,1004	rc
igs0797	**3,3**	-1,4	-1,2	**0,000**	**0,022**	0,198	SMU,1004	SMU,1005	fw
igs0797	1,2	-1,5	-1,4	0,732	0,270	0,419	SMU,1004	SMU,1005	rc
igs0798	-1,4	-1,2	1,5	**0,005**	0,103	**0,001**	SMU,1005	SMU,1006	fw
igs0798	-1,5	-1,6	1,0	**0,001**	**0,001**	0,791	SMU,1005	SMU,1006	rc
igs0800	1,4	-1,0	1,9	**0,000**	0,858	**0,000**	SMU,1007	SMU,1008	fw
igs0800	1,8	-1,9	-1,2	**0,042**	**0,005**	0,196	SMU,1007	SMU,1008	rc
igs0801	-1,2	1,3	-1,1	0,060	**0,000**	0,104	SMU,1009	SMU,1010	fw
igs0801	-1,4	-1,4	1,4	0,234	0,171	0,180	SMU,1009	SMU,1010	rc
igs0802	1,4	1,3	-1,2	**0,000**	**0,004**	0,089	SMU,1010	SMU,1011	fw
igs0802	1,3	-1,1	1,1	0,205	0,747	0,453	SMU,1010	SMU,1011	rc
igs0804	-1,1	-1,2	-1,0	0,417	0,115	0,771	SMU,1012c	SMU,1013c	fw
igs0804	-1,3	-1,2	1,7	**0,015**	0,056	**0,000**	SMU,1012c	SMU,1013c	rc
igs0805	-1,6	1,0	1,2	**0,000**	0,741	0,367	SMU,1013c	SMU,1014	fw
igs0805	1,1	1,3	-1,2	0,535	0,153	0,300	SMU,1013c	SMU,1014	rc
igs0808	-1,4	-1,1	1,3	**0,000**	**0,026**	**0,000**	SMU,1017	SMU,1018	fw
igs0808	-1,2	-1,7	1,2	0,155	**0,000**	**0,012**	SMU,1017	SMU,1018	rc
igs0809	-1,1	1,0	-1,2	0,723	0,953	0,325	SMU,1018	SMU,1019	fw
igs0809	1,2	1,1	-1,0	0,604	0,700	0,928	SMU,1018	SMU,1019	rc
igs0810	-1,2	-1,0	-1,0	**0,001**	0,621	**0,016**	SMU,1021	SMU,1022	fw
igs0810	1,6	1,1	-1,4	**0,001**	0,103	**0,000**	SMU,1021	SMU,1022	rc
igs0812	1,5	1,5	1,1	**0,000**	**0,002**	0,188	SMU,1023	SMU,1024c	fw
igs0812	1,0	-1,3	1,3	0,956	0,738	0,743	SMU,1023	SMU,1024c	rc
igs0813	-1,1	1,2	-1,7	0,609	0,263	**0,002**	SMU,1024c	SMU,1025	fw
igs0813	1,4	1,5	1,8	**0,042**	**0,002**	**0,000**	SMU,1024c	SMU,1025	rc
igs0814	1,3	-1,4	1,6	**0,000**	**0,000**	**0,000**	SMU,1025	SMU,1026	fw
igs0814	-1,1	-1,0	-1,4	0,575	0,825	0,300	SMU,1025	SMU,1026	rc
igs0815	-1,1	1,2	1,3	0,304	**0,042**	**0,004**	SMU,1026	SMU,1027	fw
igs0815	-1,3	1,2	1,2	0,381	0,263	0,317	SMU,1026	SMU,1027	rc
igs0816	1,3	1,2	1,9	**0,004**	**0,035**	**0,001**	SMU,1027	SMU,1028	fw
igs0816	-1,4	1,1	1,4	0,065	0,402	0,077	SMU,1027	SMU,1028	rc
igs0817	-1,1	-1,2	-1,0	**0,042**	**0,000**	0,589	SMU,1028	SMU,1029	fw

igs0817	-1,1	-1,2	1,4	0,577	0,369	0,060	SMU,1028	SMU,1029	rc
igs0818	1,1	-1,0	1,1	0,432	0,862	0,188	SMU,1029	SMU,1030	fw
igs0818	1,0	1,1	1,0	0,322	**0,025**	0,550	SMU,1029	SMU,1030	rc
igs0819	-1,1	-1,2	1,2	**0,023**	**0,037**	**0,003**	SMU,1030	SMU,1031	fw
igs0819	-1,1	1,0	1,1	**0,009**	0,991	0,136	SMU,1030	SMU,1031	rc
igs0820	-1,4	-1,3	1,2	**0,009**	**0,000**	**0,034**	SMU,1031	SMU,1032	fw
igs0820	1,1	-1,5	1,2	0,301	**0,035**	0,164	SMU,1031	SMU,1032	rc
igs0821	1,2	-1,3	-1,1	0,246	0,081	0,386	SMU,1032	SMU,1034c	fw
igs0821	1,1	-1,3	-1,1	0,688	0,093	0,401	SMU,1032	SMU,1034c	rc
igs0822	1,8	-1,9	-1,3	**0,040**	**0,029**	0,535	SMU,1034c	SMU,1035	fw
igs0822	-1,3	-1,1	-1,2	**0,005**	**0,000**	**0,002**	SMU,1034c	SMU,1035	rc
igs0824	-1,7	-1,9	1,4	0,247	0,173	0,435	SMU,1036	SMU,1037c	fw
igs0824	1,6	-1,7	1,4	**0,007**	**0,037**	0,159	SMU,1036	SMU,1037c	rc
igs0825	1,0	1,0	-1,5	0,774	0,862	**0,002**	SMU,1038c	SMU,1039c	fw
igs0825	1,1	-1,3	1,6	0,316	0,062	**0,000**	SMU,1038c	SMU,1039c	rc
igs0827	1,1	1,6	2,0	0,749	0,173	**0,031**	SMU,1040c	SMU,1041	fw
igs0827	1,3	1,7	1,4	0,140	**0,036**	0,235	SMU,1040c	SMU,1041	rc
igs0828	-1,8	-1,9	1,1	**0,026**	0,052	0,616	SMU,1042	SMU,1043c	fw
igs0828	1,3	-1,1	**3,7**	**0,001**	0,583	**0,000**	SMU,1042	SMU,1043c	rc
igs0830	1,3	1,2	1,3	0,394	0,593	0,494	SMU,1046c	SMU,1047c	fw
igs0830	1,0	-1,1	1,2	0,859	0,553	0,453	SMU,1046c	SMU,1047c	rc
igs0831	1,3	**2,5**	1,1	**0,000**	**0,000**	0,089	SMU,1048	SMU,1050	fw
igs0831	1,1	-1,3	1,6	0,694	0,434	0,190	SMU,1048	SMU,1050	rc
igs0832	-1,0	**5,9**	-1,1	0,972	**0,000**	0,608	SMU,1050	SMU,1051	fw
igs0832	1,2	1,1	1,5	**0,013**	0,154	**0,000**	SMU,1050	SMU,1051	rc
igs0834	-1,1	**6,0**	1,3	0,417	**0,000**	0,087	SMU,1052	SMU,1053	fw
igs0834	-1,0	-1,2	1,1	0,931	0,490	0,659	SMU,1052	SMU,1053	rc
igs0836	1,1	-1,4	-1,4	0,551	**0,007**	0,058	SMU,1056	SMU,1057	fw
igs0836	-1,7	1,7	-4,6	**0,001**	**0,015**	**0,002**	SMU,1056	SMU,1057	rc
igs0837	-1,2	-1,7	-1,1	0,494	**0,017**	0,670	SMU,1058	SMU,1059	fw
igs0837	-1,0	-2,6	1,3	0,913	**0,000**	0,053	SMU,1058	SMU,1059	rc
igs0839	1,0	-1,1	1,0	0,892	0,669	0,850	SMU,1060	SMU,1061	fw
igs0839	-1,1	-1,1	1,6	0,365	0,362	**0,000**	SMU,1060	SMU,1061	rc
igs0840	1,4	-1,0	1,2	0,484	0,952	0,631	SMU,1061	SMU,1062	fw
igs0840	-1,1	-1,2	1,2	0,412	0,133	**0,017**	SMU,1061	SMU,1062	rc
igs0842	1,0	-1,3	-1,1	0,746	**0,045**	0,503	SMU,1063	SMU,1064c	fw
igs0842	-1,1	-1,3	-1,2	0,488	0,173	0,191	SMU,1063	SMU,1064c	rc
igs0844	1,3	-1,0	1,1	0,206	0,972	0,707	SMU,1065c	SMU,1066	fw
igs0844	1,3	-1,2	1,1	0,429	0,644	0,721	SMU,1065c	SMU,1066	rc
igs0845	-1,1	-1,4	-1,3	0,876	0,273	0,495	SMU,1066	SMU,1067c	fw
igs0845	1,1	-1,2	1,2	0,871	0,738	0,788	SMU,1066	SMU,1067c	rc
igs0847	-1,1	1,0	-1,1	0,546	0,913	0,662	SMU,1070c	SMU,1071c	fw
igs0847	-1,1	-1,1	-1,3	0,547	0,369	0,344	SMU,1070c	SMU,1071c	rc
igs0848	-1,1	-2,0	1,6	**0,006**	**0,000**	**0,007**	SMU,1071c	SMU,1072c	fw
igs0848	1,8	-1,5	**4,3**	**0,000**	**0,000**	**0,000**	SMU,1071c	SMU,1072c	rc
igs0849	-1,1	1,1	1,5	0,822	0,863	0,278	SMU,1072c	SMU,1073	fw
igs0849	-1,0	**2,1**	-1,8	0,955	**0,003**	**0,004**	SMU,1072c	SMU,1073	rc
igs0850	-1,4	1,4	-1,2	0,183	0,228	0,496	SMU,1073	SMU,1074	fw
igs0850	**2,8**	1,4	1,7	**0,000**	0,370	**0,023**	SMU,1073	SMU,1074	rc
igs0851	1,4	-1,0	1,1	**0,005**	0,913	0,289	SMU,1075	SMU,1076	fw
igs0851	1,4	-1,8	1,4	**0,001**	**0,004**	**0,000**	SMU,1075	SMU,1076	rc
igs0852	-1,1	-1,2	1,0	0,273	0,169	0,930	SMU,1076	SMU,1077	fw
igs0852	-1,1	-1,5	-1,2	0,648	0,396	0,529	SMU,1076	SMU,1077	rc
igs0853	1,6	-1,5	1,0	0,161	0,275	0,959	SMU,1077	SMU,1078c	fw
igs0853	-1,3	1,1	1,3	0,272	0,672	0,344	SMU,1077	SMU,1078c	rc
igs0857	-1,0	-1,4	-1,1	0,905	**0,000**	0,377	SMU,1085	SMU,1086	fw
igs0857	1,2	-1,0	1,5	0,066	0,768	**0,012**	SMU,1085	SMU,1086	rc
igs0858	1,0	1,7	1,0	0,936	0,271	0,993	SMU,1086	SMU,1087	fw
igs0858	-1,0	-1,9	-1,4	0,921	**0,001**	0,181	SMU,1086	SMU,1087	rc
igs0859	-1,6	-1,1	1,0	**0,001**	0,434	0,774	SMU,1087	SMU,1088	fw
igs0859	1,1	-1,2	-1,6	0,881	0,736	0,434	SMU,1087	SMU,1088	rc
igs0862	-1,3	1,1	1,6	**0,026**	0,406	**0,020**	SMU,1090	SMU,1091	fw
igs0862	1,7	-1,3	1,5	**0,007**	0,315	0,081	SMU,1090	SMU,1091	rc
igs0863	1,2	1,2	-1,1	0,051	0,317	0,775	SMU,1091	SMU,1093	fw
igs0863	1,1	1,0	1,3	0,823	0,974	0,540	SMU,1091	SMU,1093	rc
igs0865	1,3	-1,5	1,6	0,524	0,359	0,281	SMU,1094	SMU,1095	fw

igs0865	-1,3	-1,1	1,4	0,133	**0,001**	**0,000**	SMU,1094	SMU,1095	rc
igs0866	-1,0	-1,1	-1,2	0,722	0,538	0,332	SMU,1098c	SMU,1100c	fw
igs0866	-1,2	-1,2	1,0	0,081	0,231	0,936	SMU,1098c	SMU,1100c	rc
igs0867	-1,1	1,3	-1,1	0,590	0,248	0,574	SMU,1100c	SMU,1102	fw
igs0867	-1,2	-1,1	-1,0	**0,026**	0,593	0,952	SMU,1100c	SMU,1102	rc
igs0868	-1,1	-1,0	1,0	0,489	0,853	0,839	SMU,1102	SMU,1104c	fw
igs0868	1,3	-1,2	1,1	**0,002**	0,073	0,528	SMU,1102	SMU,1104c	rc
igs0870	-1,1	1,0	1,3	0,550	0,823	0,051	SMU,1106c	SMU,1107c	fw
igs0870	1,1	-1,0	1,1	0,407	0,896	0,297	SMU,1106c	SMU,1107c	rc
igs0871	-1,0	-1,2	-1,2	0,812	0,158	0,364	SMU,1107c	SMU,1108c	fw
igs0871	1,2	-1,0	1,2	0,235	0,891	0,526	SMU,1107c	SMU,1108c	rc
igs0872	1,0	1,3	-1,0	0,919	0,386	0,917	SMU,1108c	SMU,1109c	fw
igs0872	-1,2	-1,3	-1,3	0,405	0,294	0,429	SMU,1108c	SMU,1109c	rc
igs0873	-1,4	1,4	1,2	0,103	0,220	0,311	SMU,1109c	SMU,1111c	fw
igs0873	-1,1	-1,0	1,1	0,346	0,892	0,465	SMU,1109c	SMU,1111c	rc
igs0874	1,4	1,1	-1,3	0,281	0,564	0,348	SMU,1111c	SMU,1112c	fw
igs0874	1,1	-1,2	1,4	0,093	0,203	**0,015**	SMU,1111c	SMU,1112c	rc
igs0875	1,3	1,2	-1,1	0,342	0,459	0,842	SMU,1112c	SMU,1113	fw
igs0875	-1,3	-1,2	1,3	**0,003**	0,052	**0,000**	SMU,1112c	SMU,1113	rc
igs0877	1,2	1,5	-1,2	0,484	0,190	0,357	SMU,1114	SMU,1115	fw
igs0877	-1,1	1,3	-1,4	0,751	0,537	0,427	SMU,1114	SMU,1115	rc
igs0878	1,7	-1,1	1,6	0,346	0,873	0,372	SMU,1115	SMU,1116c	fw
igs0878	-1,2	1,4	1,4	0,716	0,550	0,566	SMU,1115	SMU,1116c	rc
igs0879	1,4	-1,4	**2,0**	0,452	0,464	0,085	SMU,1116c	SMU,1117	fw
igs0879	1,6	-1,2	1,7	**0,000**	0,094	**0,000**	SMU,1116c	SMU,1117	rc
igs0880	1,1	1,0	-1,3	0,776	0,885	0,331	SMU,1117	SMU,1118c	fw
igs0880	1,0	-1,5	-1,1	0,812	**0,000**	0,601	SMU,1117	SMU,1118c	rc
igs0882	-1,1	-1,3	1,1	0,890	0,512	0,820	SMU,1120	SMU,1121c	fw
igs0882	1,4	-1,1	1,3	**0,003**	0,707	**0,013**	SMU,1120	SMU,1121c	rc
igs0883	1,5	-1,4	-1,0	0,405	0,510	0,928	SMU,1121c	SMU,1122	fw
igs0883	**3,0**	-1,1	1,6	**0,000**	0,304	**0,041**	SMU,1121c	SMU,1122	rc
igs0885	-1,1	-1,3	-1,5	0,571	0,178	0,082	SMU,1124	SMU,1125c	fw
igs0885	1,0	-1,9	-1,2	0,412	**0,005**	**0,006**	SMU,1124	SMU,1125c	rc
igs0886	-1,2	1,1	1,5	0,546	0,809	0,347	SMU,1125c	SMU,1126	fw
igs0886	-1,1	1,1	1,2	0,515	0,296	0,426	SMU,1125c	SMU,1126	rc
igs0887	-1,0	-1,8	1,9	0,924	**0,001**	**0,000**	SMU,1126	SMU,1127	fw
igs0887	-2,1	-1,1	-1,2	0,285	0,879	0,785	SMU,1126	SMU,1127	rc
igs0888	-1,1	-1,6	1,5	0,653	**0,028**	0,064	SMU,1127	SMU,1128	fw
igs0888	1,3	-1,3	1,5	0,474	0,533	0,372	SMU,1127	SMU,1128	rc
igs0889	-1,3	-1,9	1,6	0,057	**0,001**	0,055	SMU,1129	SMU,1131c	fw
igs0889	1,2	1,6	1,4	**0,007**	**0,002**	0,237	SMU,1129	SMU,1131c	rc
igs0890	-1,2	-1,1	1,1	0,119	0,614	0,626	SMU,1131c	SMU,1132	fw
igs0890	-1,4	1,3	-1,3	**0,001**	**0,005**	**0,021**	SMU,1131c	SMU,1132	rc
igs0891	1,6	1,3	1,4	0,251	0,591	0,466	SMU,1132	SMU,1133	fw
igs0891	-1,0	**2,2**	1,3	0,801	**0,019**	0,100	SMU,1132	SMU,1133	rc
igs0892	1,2	-1,3	-1,2	**0,001**	**0,000**	**0,009**	SMU,1133	SMU,1134c	fw
igs0892	-2,5	-2,9	-1,9	**0,000**	**0,000**	**0,001**	SMU,1133	SMU,1134c	rc
igs0895	-1,2	-1,1	-1,5	0,485	0,451	0,070	SMU,1137	SMU,1138	fw
igs0895	-1,5	-1,2	1,1	**0,011**	**0,000**	0,240	SMU,1137	SMU,1138	rc
igs0896	1,3	-1,2	1,7	0,621	0,783	0,381	SMU,1138	SMU,1139c	fw
igs0896	1,0	-1,3	2,0	0,878	0,245	**0,000**	SMU,1138	SMU,1139c	rc
igs0897	-2,1	-2,0	-1,2	0,109	0,088	0,676	SMU,1139c	SMU,1140c	fw
igs0897	-1,1	-1,7	1,6	0,155	**0,001**	**0,000**	SMU,1139c	SMU,1140c	rc
igs0898	-1,2	-1,5	-1,1	0,578	0,129	0,619	SMU,1140c	SMU,1141c	fw
igs0898	-1,1	-1,4	-1,2	0,303	**0,016**	0,071	SMU,1140c	SMU,1141c	rc
igs0899	-1,0	-1,1	-1,9	0,917	0,783	**0,018**	SMU,1142c	SMU,1143c	fw
igs0899	-1,3	-1,0	1,2	**0,000**	0,802	0,060	SMU,1142c	SMU,1143c	rc
igs0900	1,3	-1,3	-1,0	0,276	0,261	0,999	SMU,1143c	SMU,1144	fw
igs0900	1,4	1,3	2,0	**0,002**	**0,008**	**0,003**	SMU,1143c	SMU,1144	rc
igs0901	1,0	1,6	-1,1	0,977	0,055	0,690	SMU,1144	SMU,1145c	fw
igs0901	-1,3	-1,0	-1,4	**0,011**	0,896	0,129	SMU,1144	SMU,1145c	rc
igs0903	1,1	1,7	-1,2	0,434	**0,000**	0,184	SMU,1147c	SMU,1148	fw
igs0903	-1,1	-1,2	1,1	0,208	**0,019**	0,100	SMU,1147c	SMU,1148	rc
igs0904	-1,1	1,0	-1,2	0,754	0,981	0,752	SMU,1150	SMU,1151c	fw
igs0904	-1,1	-1,0	1,1	0,497	0,601	0,542	SMU,1150	SMU,1151c	rc
igs0905	-1,3	-1,5	1,4	**0,044**	**0,004**	**0,005**	SMU,1152c	SMU,1153c	fw

igs0905	1,2	-1,1	1,2	0,121	0,130	0,203	SMU,1152c	SMU,1153c	rc
igs0906	-1,0	1,1	-1,0	0,876	0,752	0,854	SMU,1153c	SMU,1154c	fw
igs0906	1,0	1,1	1,5	0,458	0,208	**0,000**	SMU,1153c	SMU,1154c	rc
igs0907	-1,2	-1,2	1,1	0,604	0,507	0,775	SMU,1156c	SMU,1157c	fw
igs0907	1,1	1,1	1,1	0,131	0,292	**0,002**	SMU,1156c	SMU,1157c	rc
igs0909	-1,1	-1,2	1,0	0,613	0,294	0,945	SMU,1158c	SMU,1159c	fw
igs0909	-1,1	1,0	1,1	**0,000**	0,665	0,197	SMU,1158c	SMU,1159c	rc
igs0910	1,5	-1,1	-1,1	**0,001**	0,125	0,442	SMU,1159c	SMU,1160c	fw
igs0910	1,2	1,1	1,8	**0,030**	**0,032**	**0,000**	SMU,1159c	SMU,1160c	rc
igs0911	-1,1	-1,0	1,3	0,553	0,683	0,099	SMU,1160c	SMU,1161c	fw
igs0911	-1,1	-1,1	-1,4	0,355	0,439	0,115	SMU,1160c	SMU,1161c	rc
igs0912	-1,1	-1,1	1,0	0,648	0,666	0,832	SMU,1161c	SMU,1163c	fw
igs0912	-1,2	-1,3	-1,0	**0,036**	0,052	0,797	SMU,1161c	SMU,1163c	rc
igs0914	-1,3	-1,2	-1,5	0,261	0,363	**0,007**	SMU,1165c	SMU,1166c	fw
igs0914	1,5	-1,7	-1,0	**0,000**	**0,000**	0,765	SMU,1165c	SMU,1166c	rc
igs0916	2,0	1,1	-1,9	**0,016**	0,815	**0,002**	SMU,1167c	SMU,1168	fw
igs0916	**2,6**	-1,0	1,1	**0,000**	0,700	0,619	SMU,1167c	SMU,1168	rc
igs0917	1,1	-1,0	1,0	0,584	0,795	0,955	SMU,1168	SMU,1169c	fw
igs0917	-1,0	-1,2	-1,2	0,998	0,373	0,253	SMU,1168	SMU,1169c	rc
igs0919	1,1	1,1	-1,1	0,869	0,854	0,843	SMU,1170	SMU,1171c	fw
igs0919	-1,3	1,1	1,1	**0,034**	0,733	0,465	SMU,1170	SMU,1171c	rc
igs0920	1,2	1,2	-1,5	0,513	0,513	0,186	SMU,1171c	SMU,1172c	fw
igs0920	1,1	-1,0	-1,2	0,246	0,670	**0,016**	SMU,1171c	SMU,1172c	rc
igs0922	-1,2	1,2	1,0	0,355	0,312	0,856	SMU,1173	SMU,1174	fw
igs0922	-1,1	-1,3	1,5	0,603	**0,008**	**0,000**	SMU,1173	SMU,1174	rc
igs0923	-1,0	1,2	1,1	0,915	0,565	0,806	SMU,1174	SMU,1175	fw
igs0923	1,2	-1,2	-1,0	0,564	0,471	0,925	SMU,1174	SMU,1175	rc
igs0924	1,2	-1,2	1,2	0,177	0,344	0,359	SMU,1175	SMU,1176	fw
igs0924	-1,4	-1,6	1,5	0,086	**0,009**	**0,029**	SMU,1175	SMU,1176	rc
igs0925	-1,8	1,1	1,2	0,232	0,836	0,593	SMU,1176	SMU,1177c	fw
igs0925	-1,6	-1,1	-1,2	**0,010**	0,625	0,096	SMU,1176	SMU,1177c	rc
igs0928	-1,3	-1,2	-1,3	0,397	0,449	0,316	SMU,1179c	SMU,1180	fw
igs0928	-1,3	-1,2	-1,4	0,117	0,394	0,132	SMU,1179c	SMU,1180	rc
igs0929	-1,1	-1,2	-1,1	0,374	0,365	0,594	SMU,1180	SMU,1182	fw
igs0929	-1,1	-1,0	1,3	0,195	0,385	**0,000**	SMU,1180	SMU,1182	rc
igs0932	-2,1	-1,1	1,1	**0,009**	0,224	0,172	SMU,1184c	SMU,1185	fw
igs0932	-1,1	-1,3	1,7	**0,043**	**0,000**	**0,000**	SMU,1184c	SMU,1185	rc
igs0933	-1,0	1,2	-1,3	0,870	0,152	0,085	SMU,1185	SMU,1187	fw
igs0933	1,0	-1,1	1,0	0,627	**0,042**	0,455	SMU,1185	SMU,1187	rc
igs0934	1,1	1,1	-1,2	0,866	0,927	0,843	SMU,1187	SMU,1188	fw
igs0934	-1,4	1,5	1,6	**0,000**	**0,001**	**0,001**	SMU,1187	SMU,1188	rc
igs0935	-2,3	1,4	-1,0	0,091	0,369	0,941	SMU,1188	SMU,1189c	fw
igs0935	1,2	1,5	-1,7	0,172	**0,002**	**0,000**	SMU,1188	SMU,1189c	rc
igs0936	-1,1	-1,1	1,3	0,701	0,726	0,097	SMU,1189c	SMU,1190	fw
igs0936	-1,4	-1,2	1,1	**0,000**	**0,008**	0,244	SMU,1189c	SMU,1190	rc
igs0937	1,3	1,1	1,1	0,188	0,668	0,826	SMU,1190	SMU,1191	fw
igs0937	1,4	-1,9	1,2	**0,000**	**0,000**	**0,016**	SMU,1190	SMU,1191	rc
igs0938	1,0	1,0	-1,7	0,945	0,978	0,486	SMU,1191	SMU,1192	fw
igs0938	1,1	-1,4	1,2	**0,041**	**0,001**	**0,000**	SMU,1191	SMU,1192	rc
igs0939	-1,2	1,2	1,0	0,697	0,462	0,869	SMU,1192	SMU,1193	fw
igs0939	-1,6	-1,3	1,2	0,122	**0,024**	0,267	SMU,1192	SMU,1193	rc
igs0941	-1,2	-1,3	-1,4	0,317	0,121	0,155	SMU,1195	SMU,1196c	fw
igs0941	**2,0**	-1,8	-1,3	**0,000**	**0,001**	0,098	SMU,1195	SMU,1196c	rc
igs0942	1,5	1,1	1,5	0,359	0,748	0,388	SMU,1196c	SMU,1197	fw
igs0942	1,1	1,1	1,2	0,142	0,360	**0,019**	SMU,1196c	SMU,1197	rc
igs0943	1,3	-1,1	1,3	0,494	0,797	0,533	SMU,1197	SMUt37	fw
igs0943	1,2	-1,2	1,1	**0,037**	0,325	0,287	SMU,1197	SMUt37	rc
igs0944	1,3	-1,4	1,0	0,713	0,615	0,970	SMUt37	SMU,1200	fw
igs0944	-1,5	-1,1	1,0	**0,000**	0,182	0,707	SMUt37	SMU,1200	rc
igs0945	-1,1	-1,5	1,0	0,730	0,124	0,993	SMU,1200	SMUt38	fw
igs0945	-1,1	1,3	-1,1	0,638	0,175	0,524	SMU,1200	SMUt38	rc
igs0946	1,2	1,1	-1,0	0,212	0,344	0,880	SMUt38	SMUt39	fw
igs0946	-1,2	-1,0	1,3	0,302	0,927	0,259	SMUt38	SMUt39	rc
igs0947	1,5	-1,1	-1,2	0,528	0,864	0,840	SMUt39	SMU,1201c	fw
igs0947	1,1	1,1	-1,5	0,397	0,481	**0,004**	SMUt39	SMU,1201c	rc
igs0948	-1,1	-1,1	-1,3	0,859	0,813	0,496	SMU,1201c	SMU,1203	fw

igs0948	1,0	-1,5	1,2	0,573	**0,000**	0,318	SMU,1201c	SMU,1203	rc
igs0949	-1,1	-1,3	-1,5	0,768	0,515	0,306	SMU,1203	SMU,1204	fw
igs0949	-1,3	-1,4	1,1	**0,000**	**0,000**	0,090	SMU,1203	SMU,1204	rc
igs0950	-1,3	-1,4	1,2	0,153	0,095	0,524	SMU,1204	SMU,1205c	fw
igs0950	-1,1	1,4	1,2	0,221	**0,010**	**0,013**	SMU,1204	SMU,1205c	rc
igs0951	1,1	-1,1	-1,1	**0,007**	**0,032**	0,165	SMU,1205c	SMU,1206c	fw
igs0951	1,2	-1,4	1,7	0,502	0,109	0,045	SMU,1205c	SMU,1206c	rc
igs0952	1,1	-1,1	-1,4	0,360	0,705	**0,017**	SMU,1206c	SMU,1207	fw
igs0952	-1,0	-1,3	1,7	0,710	**0,000**	**0,000**	SMU,1206c	SMU,1207	rc
igs0953	1,3	1,1	1,2	**0,002**	**0,026**	**0,028**	SMU,1207	SMU,1208c	fw
igs0953	1,0	-1,3	1,2	0,956	**0,001**	0,047	SMU,1207	SMU,1208c	rc
igs0954	1,0	-1,1	1,6	0,986	0,657	0,063	SMU,1208c	SMU,1209c	fw
igs0954	-1,2	1,2	-1,5	0,194	0,346	**0,017**	SMU,1208c	SMU,1209c	rc
igs0956	-1,5	1,5	-1,2	0,124	0,214	0,324	SMU,1210	SMU,1211	fw
igs0956	1,1	1,4	1,4	0,559	**0,008**	0,099	SMU,1210	SMU,1211	rc
igs0957	1,1	-1,4	-1,3	0,764	0,152	0,430	SMU,1211	SMU,1213c	fw
igs0957	-1,1	-1,3	-1,2	0,237	**0,016**	0,068	SMU,1211	SMU,1213c	rc
igs0958	-1,5	-1,4	-1,1	**0,001**	**0,012**	0,487	SMU,1213c	SMU,1214	fw
igs0958	-1,1	-1,0	1,8	0,809	0,922	**0,045**	SMU,1213c	SMU,1214	rc
igs0961	-1,0	-1,1	1,2	0,938	0,758	0,687	SMU,1216c	SMU,1217c	fw
igs0961	-1,3	-1,1	1,2	**0,040**	0,333	0,220	SMU,1216c	SMU,1217c	rc
igs0962	1,1	1,0	-1,2	0,669	0,939	0,348	SMU,1217c	SMU,1218	fw
igs0962	-1,2	-1,0	-1,2	**0,000**	0,794	**0,042**	SMU,1217c	SMU,1218	rc
igs0963	1,1	1,3	-1,4	0,505	0,061	**0,002**	SMU,1218	SMU,1219c	fw
igs0963	-1,1	1,1	1,3	0,373	0,119	**0,002**	SMU,1218	SMU,1219c	rc
igs0964	-1,0	1,2	-1,6	0,889	0,407	**0,024**	SMU,1219c	SMU,1220c	fw
igs0964	-1,1	1,0	-1,1	**0,000**	0,703	0,071	SMU,1219c	SMU,1220c	rc
igs0965	1,0	1,2	1,2	0,969	0,145	0,227	SMU,1220c	SMU,1221	fw
igs0965	1,1	1,2	-1,1	0,294	**0,015**	**0,016**	SMU,1220c	SMU,1221	rc
igs0966	-1,6	-1,1	-1,8	**0,014**	0,599	**0,000**	SMU,1221	SMU,1222	fw
igs0966	1,7	1,9	**2,2**	**0,000**	**0,000**	**0,000**	SMU,1221	SMU,1222	rc
igs0968	-1,0	1,1	1,1	0,972	0,790	0,858	SMU,1224	SMU,1225	fw
igs0968	1,1	1,0	1,4	0,148	0,664	**0,000**	SMU,1224	SMU,1225	rc
igs0969	1,3	1,3	-1,3	0,490	0,469	0,460	SMU,1225	SMU,1226c	fw
igs0969	1,1	-1,3	-1,1	0,897	0,425	0,700	SMU,1225	SMU,1226c	rc
igs0971	-1,1	-1,3	-1,3	0,787	0,167	0,213	SMU,1227	SMU,1228c	fw
igs0971	1,1	1,1	1,1	**0,000**	0,151	0,257	SMU,1227	SMU,1228c	rc
igs0974	-1,0	-1,2	-1,3	0,919	0,507	0,295	SMU,1230c	SMU,1231c	fw
igs0974	-1,1	1,1	1,1	0,081	0,343	**0,008**	SMU,1230c	SMU,1231c	rc
igs0975	-1,0	-1,2	-1,1	0,985	0,130	0,485	SMU,1231c	SMU,1232c	fw
igs0975	-1,1	-1,3	-1,1	0,096	**0,014**	0,395	SMU,1231c	SMU,1232c	rc
igs0977	-1,3	-1,3	-1,4	0,116	**0,020**	**0,008**	SMU,1233	SMU,1234	fw
igs0977	-1,0	-1,0	-1,1	0,719	0,582	0,464	SMU,1233	SMU,1234	rc
igs0978	-1,3	1,2	-1,4	0,579	0,757	0,475	SMU,1234	SMU,1235	fw
igs0978	1,0	1,2	1,0	0,889	**0,016**	0,826	SMU,1234	SMU,1235	rc
igs0979	-1,1	1,1	-1,5	0,825	0,777	0,091	SMU,1235	SMU,1236c	fw
igs0979	-1,1	1,0	1,5	0,057	0,240	**0,001**	SMU,1235	SMU,1236c	rc
igs0981	-1,1	-1,1	-1,0	0,826	0,690	0,965	SMU,1237c	SMU,1238c	fw
igs0981	-1,5	1,0	1,3	**0,005**	0,939	0,082	SMU,1237c	SMU,1238c	rc
igs0982	1,0	-1,1	-1,3	0,822	0,390	**0,002**	SMU,1238c	SMU,1239	fw
igs0982	**2,5**	1,3	1,2	**0,000**	0,166	**0,015**	SMU,1238c	SMU,1239	rc
igs0984	1,1	-1,3	-1,6	0,486	0,088	**0,003**	SMU,1240c	SMU,1241	fw
igs0984	1,2	-1,6	1,4	0,427	**0,033**	**0,001**	SMU,1240c	SMU,1241	rc
igs0985	1,1	-1,1	1,2	0,563	0,502	0,287	SMU,1241	SMU,1243	fw
igs0985	-1,1	-1,1	-1,6	0,214	**0,013**	**0,012**	SMU,1241	SMU,1243	rc
igs0986	1,3	1,1	-1,3	0,115	0,718	0,203	SMU,1243	SMU,1245c	fw
igs0986	-1,2	1,0	-1,4	**0,038**	0,943	**0,015**	SMU,1243	SMU,1245c	rc
igs0987	-1,0	1,0	-1,1	0,831	0,810	0,701	SMU,1245c	SMU,1246c	fw
igs0987	-1,3	1,1	-1,2	**0,027**	0,668	0,072	SMU,1245c	SMU,1246c	rc
igs0988	-1,8	-1,5	-1,1	**0,020**	0,091	0,696	SMU,1246c	SMU,1247	fw
igs0988	-1,4	1,9	-1,1	**0,001**	**0,000**	0,091	SMU,1246c	SMU,1247	rc
igs0989	-1,0	-1,2	-1,1	0,853	0,320	0,634	SMU,1247	SMU,1249c	fw
igs0989	-1,0	-1,1	-1,0	0,588	0,063	0,678	SMU,1247	SMU,1249c	rc
igs0990	1,2	-1,1	1,0	**0,000**	**0,032**	0,534	SMU,1249c	SMU,1250c	fw
igs0990	-1,5	1,2	1,3	**0,000**	**0,026**	**0,008**	SMU,1249c	SMU,1250c	rc
igs0991	1,2	-1,4	-1,2	0,613	0,416	0,598	SMU,1250c	SMU,1251	fw

igs0991	1,0	-1,5	-1,3	0,939	**0,012**	0,191	SMU,1250c	SMU,1251	rc
igs0992	1,1	1,1	-1,3	0,408	0,357	0,276	SMU,1251	SMU,1252	fw
igs0992	-1,1	-1,0	1,1	0,338	0,694	0,329	SMU,1251	SMU,1252	rc
igs0994	-1,1	-1,4	-1,6	0,751	0,370	0,206	SMU,1253c	SMU,1254	fw
igs0994	1,6	1,2	1,0	**0,000**	0,295	0,952	SMU,1253c	SMU,1254	rc
igs0995	-1,3	-1,3	-1,2	0,161	0,119	0,331	SMU,1255c	SMU,1256c	fw
igs0995	1,3	1,1	1,7	**0,009**	0,118	**0,000**	SMU,1255c	SMU,1256c	rc
igs0996	1,4	-1,1	-1,0	0,595	0,913	0,980	SMU,1257c	SMU,1258c	fw
igs0996	-1,1	1,1	-1,1	**0,018**	0,591	0,513	SMU,1257c	SMU,1258c	rc
igs0997	-1,1	-1,1	1,5	0,770	0,477	**0,000**	SMU,1258c	SMU,1259	fw
igs0997	1,1	1,4	-1,4	0,240	**0,000**	**0,003**	SMU,1258c	SMU,1259	rc
igs0998	1,0	-1,0	-1,1	0,839	0,805	0,567	SMU,1259	SMU,1260c	fw
igs0998	1,1	-1,0	-1,1	0,314	0,722	0,107	SMU,1259	SMU,1260c	rc
igs1000	1,1	-1,5	-1,3	**0,049**	**0,000**	**0,000**	SMU,1261c	SMU,1262c	fw
igs1000	-1,1	1,1	1,1	0,489	0,057	**0,008**	SMU,1261c	SMU,1262c	rc
igs1001	1,0	-1,7	-2,8	0,996	**0,008**	**0,000**	SMU,1262c	SMU,1263	fw
igs1001	1,1	1,1	1,4	0,540	0,781	0,234	SMU,1262c	SMU,1263	rc
igs1003	-1,4	-1,8	-1,6	**0,004**	**0,000**	**0,000**	SMU,1265	SMU,1266	fw
igs1003	1,1	1,0	-1,1	0,103	0,943	0,074	SMU,1265	SMU,1266	rc
igs1006	1,2	-1,2	-1,0	0,406	0,280	0,989	SMU,1273	SMU,1276c	fw
igs1006	1,0	1,1	1,0	0,301	0,325	0,527	SMU,1273	SMU,1276c	rc
igs1007	1,3	-1,1	-1,2	0,445	0,837	0,586	SMU,1276c	SMU,1277	fw
igs1007	1,4	1,1	1,6	**0,007**	0,495	**0,000**	SMU,1276c	SMU,1277	rc
igs1008	-1,0	1,2	-1,1	0,768	**0,043**	0,545	SMU,1278c	SMU,1279c	fw
igs1008	-1,0	-1,3	1,3	0,745	**0,012**	**0,000**	SMU,1278c	SMU,1279c	rc
igs1009	1,2	1,4	1,3	0,534	0,352	0,376	SMU,1279c	SMU,1280c	fw
igs1009	1,1	1,0	-1,3	0,302	0,955	**0,009**	SMU,1279c	SMU,1280c	rc
igs1010	-1,6	-1,0	1,9	**0,000**	0,291	**0,000**	SMU,1280c	SMU,1282	fw
igs1010	1,0	-1,7	1,2	0,642	**0,000**	0,045	SMU,1280c	SMU,1282	rc
igs1011	1,2	-1,1	1,1	0,802	0,884	0,878	SMU,1282	SMUt40	fw
igs1011	-1,2	-1,3	-1,1	0,436	**0,000**	0,049	SMU,1282	SMUt40	rc
igs1012	-1,3	1,1	-1,1	0,516	0,867	0,861	SMUt40	SMU,1284c	fw
igs1012	-1,1	-1,2	-1,1	0,320	**0,019**	0,502	SMUt40	SMU,1284c	rc
igs1014	-1,4	1,1	-1,0	0,254	0,777	0,892	SMU,1286c	SMU,1287	fw
igs1014	1,4	-1,3	1,4	**0,000**	**0,014**	**0,037**	SMU,1286c	SMU,1287	rc
igs1015	-1,3	-1,2	1,0	0,681	0,828	0,952	SMU,1287	SMU,1288	fw
igs1015	-1,4	-1,1	1,1	0,127	0,660	0,613	SMU,1287	SMU,1288	rc
igs1016	1,6	-1,2	1,2	0,565	0,794	0,819	SMU,1288	SMU,1289c	fw
igs1016	1,1	-1,7	1,0	0,145	**0,000**	0,624	SMU,1288	SMU,1289c	rc
igs1017	-1,0	1,4	1,2	0,948	0,334	0,639	SMU,1289c	SMU,1290c	fw
igs1017	2,0	1,2	1,5	**0,000**	0,165	**0,002**	SMU,1289c	SMU,1290c	rc
igs1018	-1,4	-1,5	-1,5	0,441	0,258	0,348	SMU,1291c	SMU,1292c	fw
igs1018	-1,4	1,3	1,5	**0,001**	**0,002**	**0,000**	SMU,1291c	SMU,1292c	rc
igs1019	1,2	1,1	-1,4	0,475	0,847	0,108	SMU,1292c	SMU,1293c	fw
igs1019	-1,1	-1,4	-1,2	0,109	**0,000**	0,048	SMU,1292c	SMU,1293c	rc
igs1020	1,1	-1,4	-1,7	0,773	0,468	0,247	SMU,1293c	SMU,1294	fw
igs1020	1,5	1,1	1,2	**0,000**	0,303	**0,001**	SMU,1293c	SMU,1294	rc
igs1021	1,1	-1,4	-1,2	0,306	0,129	0,332	SMU,1294	SMU,1295	fw
igs1021	-1,9	-1,0	-1,3	**0,000**	**0,036**	0,065	SMU,1294	SMU,1295	rc
igs1022	-1,1	1,3	-2,3	0,507	**0,008**	**0,001**	SMU,1295	SMU,1296	fw
igs1022	-1,4	-1,5	1,6	0,341	0,428	0,230	SMU,1295	SMU,1296	rc
igs1023	-1,1	-1,1	1,2	0,461	0,079	**0,022**	SMU,1296	SMU,1297	fw
igs1023	1,0	-1,2	-1,8	0,842	0,218	**0,000**	SMU,1296	SMU,1297	rc
igs1024	1,3	1,1	1,5	**0,004**	0,335	**0,000**	SMU,1297	SMU,1298	fw
igs1024	-1,5	-1,3	-1,1	0,491	0,687	0,835	SMU,1297	SMU,1298	rc
igs1025	1,5	-1,2	-1,0	0,513	0,717	0,965	SMU,1298	SMU,1299c	fw
igs1025	-1,2	1,7	1,8	0,802	0,529	0,421	SMU,1298	SMU,1299c	rc
igs1026	-1,1	-1,3	-2,0	0,771	0,222	**0,015**	SMU,1300c	SMU,1301c	fw
igs1026	1,9	1,3	-1,6	**0,002**	**0,007**	**0,001**	SMU,1300c	SMU,1301c	rc
igs1027	-1,5	1,4	-1,0	**0,031**	**0,025**	0,921	SMU,1301c	SMU,1302	fw
igs1027	-1,2	-1,1	1,1	0,162	0,673	0,181	SMU,1301c	SMU,1302	rc
igs1028	-1,2	-1,8	-1,2	0,578	**0,020**	0,497	SMU,1302	SMU,1303c	fw
igs1028	1,1	-1,1	1,1	0,374	0,584	0,497	SMU,1302	SMU,1303c	rc
igs1029	-1,1	-1,3	1,1	0,665	0,255	0,776	SMU,1303c	SMU,1304c	fw
igs1029	-1,1	-1,1	1,1	0,826	0,877	0,773	SMU,1303c	SMU,1304c	rc
igs1030	-1,0	-1,0	-1,8	0,901	0,756	**0,001**	SMU,1307c	SMU,1308	fw

igs1030	-1,0	1,0	-1,3	0,618	1,000	**0,010**	SMU,1307c	SMU,1308	rc
igs1031	-1,1	-1,7	-1,2	**0,040**	**0,000**	**0,009**	SMU,1308	SMU,1309c	fw
igs1031	-1,4	1,5	**2,3**	**0,047**	**0,043**	**0,007**	SMU,1308	SMU,1309c	rc
igs1032	-1,2	-1,3	-1,1	0,689	0,651	0,908	SMU,1309c	SMU,1310	fw
igs1032	1,2	1,3	1,0	**0,010**	**0,001**	0,356	SMU,1309c	SMU,1310	rc
igs1033	-1,2	-1,6	1,1	0,647	0,186	0,869	SMU,1310	SMU,1311	fw
igs1033	1,1	-1,6	-1,2	0,224	**0,000**	0,109	SMU,1310	SMU,1311	rc
igs1034	1,0	-1,1	1,6	0,890	0,695	**0,009**	SMU,1311	SMU,1312	fw
igs1034	-1,2	**2,0**	**2,4**	**0,029**	**0,001**	**0,000**	SMU,1311	SMU,1312	rc
igs1035	-1,1	-1,5	1,1	0,932	0,406	0,831	SMU,1312	SMU,1313c	fw
igs1035	-2,3	-1,2	1,4	**0,000**	0,144	**0,042**	SMU,1312	SMU,1313c	rc
igs1036	-1,1	-1,1	-1,0	0,362	0,323	0,977	SMU,1313c	SMU,1314	fw
igs1036	1,0	1,0	-1,2	0,895	0,934	0,577	SMU,1313c	SMU,1314	rc
igs1037	-1,3	-1,9	-1,2	0,360	**0,004**	0,384	SMU,1314	SMU,1315c	fw
igs1037	-1,1	-1,3	-1,3	0,800	0,537	0,568	SMU,1314	SMU,1315c	rc
igs1039	-1,3	-1,1	1,0	0,099	0,498	0,872	SMU,1316c	SMU,1317c	fw
igs1039	1,2	1,1	-1,1	0,260	0,080	0,336	SMU,1316c	SMU,1317c	rc
igs1040	-1,0	-1,1	-1,1	0,934	0,586	0,717	SMU,1317c	SMU,1319c	fw
igs1040	-1,2	-1,1	1,0	0,157	0,526	0,922	SMU,1317c	SMU,1319c	rc
igs1041	1,4	-1,0	-1,1	0,250	0,933	0,633	SMU,1319c	SMU,1321c	fw
igs1041	-1,0	1,5	-1,3	0,537	**0,000**	0,098	SMU,1319c	SMU,1321c	rc
igs1042	-1,1	-1,1	1,3	0,781	0,888	0,517	SMU,1321c	SMU,1322	fw
igs1042	1,5	1,6	1,0	**0,008**	**0,000**	0,983	SMU,1321c	SMU,1322	rc
igs1043	-1,1	1,7	1,3	0,911	0,229	0,548	SMU,1322	SMU,1323	fw
igs1043	-1,2	1,1	-1,0	0,320	0,736	0,904	SMU,1322	SMU,1323	rc
igs1044	1,0	1,1	-1,1	0,999	0,836	0,712	SMU,1323	SMU,1324	fw
igs1044	-1,3	-1,0	1,6	0,190	0,958	**0,034**	SMU,1323	SMU,1324	rc
igs1046	1,3	-1,0	-1,4	**0,006**	0,880	**0,003**	SMU,1326	SMU,1327c	fw
igs1046	-1,0	1,1	1,6	0,852	0,205	**0,000**	SMU,1326	SMU,1327c	rc
igs1047	1,1	-1,0	-1,0	0,503	0,885	0,855	SMU,1327c	SMU,1329c	fw
igs1047	1,1	1,1	-1,0	0,172	0,132	0,978	SMU,1327c	SMU,1329c	rc
igs1048	1,0	-1,2	-1,1	0,369	0,064	0,077	SMU,1329c	SMU,1330c	fw
igs1048	1,1	1,3	-1,4	0,450	0,198	**0,025**	SMU,1329c	SMU,1330c	rc
igs1049	-1,3	1,1	-1,1	**0,013**	0,077	0,711	SMU,1330c	SMU,1331c	fw
igs1049	1,0	1,0	-1,2	0,900	0,980	0,232	SMU,1330c	SMU,1331c	rc
igs1050	-1,0	1,0	-1,4	0,973	0,693	**0,006**	SMU,1331c	SMU,1332c	fw
igs1050	1,2	-1,1	1,7	**0,016**	0,285	**0,000**	SMU,1331c	SMU,1332c	rc
igs1051	1,0	1,0	1,2	0,825	0,914	0,393	SMU,1332c	SMU,1334	fw
igs1051	-1,2	-1,0	1,0	0,304	0,768	0,979	SMU,1332c	SMU,1334	rc
igs1052	-1,0	-1,2	1,5	0,641	0,247	**0,033**	SMU,1334	SMU,1335c	fw
igs1052	-1,7	-1,6	**2,3**	**0,004**	**0,018**	**0,001**	SMU,1334	SMU,1335c	rc
igs1054	**2,1**	1,2	**5,0**	**0,023**	0,221	**0,004**	SMU,1336	SMU,1337c	fw
igs1055	**2,0**	-2,4	-1,5	**0,047**	**0,039**	0,089	SMU,1337c	SMU,1338c	fw
igs1055	-1,6	-1,1	-1,4	**0,013**	0,349	**0,005**	SMU,1337c	SMU,1338c	rc
igs1056	-1,2	-1,2	1,1	0,324	0,712	0,816	SMU,1338c	SMU,1339	fw
igs1056	1,2	-1,4	-2,3	0,208	**0,004**	**0,000**	SMU,1338c	SMU,1339	rc
igs1057	-1,2	-1,5	**2,3**	0,120	**0,031**	**0,000**	SMU,1339	SMU,1340	fw
igs1057	-1,2	-2,2	1,2	0,592	**0,001**	0,382	SMU,1339	SMU,1340	rc
igs1059	-1,0	-2,6	-1,1	0,349	**0,000**	0,612	SMU,1342	SMU,1343c	fw
igs1059	**2,1**	-1,6	1,8	**0,000**	**0,001**	**0,002**	SMU,1342	SMU,1343c	rc
igs1060	-1,1	-3,7	1,1	0,610	**0,002**	0,807	SMU,1343c	SMU,1344c	fw
igs1060	-1,2	1,2	1,1	0,135	**0,046**	0,125	SMU,1343c	SMU,1344c	rc
igs1064	-1,1	1,0	**2,1**	0,648	0,915	**0,013**	SMU,1348c	SMU,1349	fw
igs1064	1,2	1,3	1,4	0,466	0,337	0,206	SMU,1348c	SMU,1349	rc
igs1065	-1,1	-1,4	1,3	0,456	0,055	**0,003**	SMU,1349	SMU,1351	fw
igs1065	1,4	1,3	-1,4	0,199	0,164	0,060	SMU,1349	SMU,1351	rc
igs1067	1,2	1,3	1,1	0,245	0,209	0,521	SMU,1353	SMU,1354c	fw
igs1067	-1,1	1,2	-1,1	0,360	0,175	0,481	SMU,1353	SMU,1354c	rc
igs1070	1,4	-1,1	-1,0	0,471	0,901	0,925	SMU,1356c	SMU,1357	fw
igs1070	1,0	1,0	1,5	0,945	0,519	**0,000**	SMU,1356c	SMU,1357	rc
igs1072	-1,0	-1,1	1,2	0,978	0,669	0,302	SMU,1358	SMU,1359	fw
igs1072	-1,3	-1,5	-1,0	0,291	0,097	0,883	SMU,1358	SMU,1359	rc
igs1073	-1,1	1,2	1,4	0,673	0,226	0,066	SMU,1359	SMU,1360c	fw
igs1073	1,4	-1,4	1,9	**0,000**	**0,014**	**0,000**	SMU,1359	SMU,1360c	rc
igs1074	1,1	1,2	1,3	0,365	0,194	**0,043**	SMU,1361c	SMU,1363c	fw
igs1074	1,1	1,1	1,1	0,117	0,285	0,329	SMU,1361c	SMU,1363c	rc

igs1075	-1,1	1,1	1,5	0,522	0,291	**0,000**	SMU,1363c	SMU,1365c	fw
igs1075	-1,3	-1,3	-1,2	**0,042**	0,082	0,301	SMU,1363c	SMU,1365c	rc
igs1078	-1,0	-1,5	1,3	0,895	**0,000**	**0,001**	SMU,1367c	SMU,1368	fw
igs1078	-1,1	1,0	-1,2	0,168	0,713	**0,006**	SMU,1367c	SMU,1368	rc
igs1079	-1,0	-1,0	-1,1	0,450	0,798	**0,003**	SMU,1368	SMU,1369	fw
igs1079	-1,1	1,3	-1,2	0,102	**0,001**	0,064	SMU,1368	SMU,1369	rc
igs1080	-1,1	1,1	1,0	0,622	0,464	0,806	SMU,1369	SMU,1370c	fw
igs1080	1,0	1,5	-1,2	0,903	**0,000**	**0,005**	SMU,1369	SMU,1370c	rc
igs1081	-1,1	1,1	-1,1	0,178	0,399	0,251	SMU,1370c	SMU,1372c	fw
igs1081	-1,0	**2,7**	-1,1	0,685	**0,000**	0,118	SMU,1370c	SMU,1372c	rc
igs1082	-1,2	-1,1	-1,4	**0,001**	0,206	**0,000**	SMU,1372c	SMU,1373c	fw
igs1082	1,1	1,5	1,4	0,165	**0,000**	**0,000**	SMU,1372c	SMU,1373c	rc
igs1083	-1,0	-1,2	-1,0	0,609	0,144	0,557	SMU,1373c	SMU,1374	fw
igs1083	1,1	**2,5**	1,0	0,111	**0,000**	0,595	SMU,1373c	SMU,1374	rc
igs1084	1,0	-1,1	-1,1	0,909	0,471	0,536	SMU,1374	SMU,1375c	fw
igs1084	-1,1	1,3	-1,2	0,123	**0,032**	**0,001**	SMU,1374	SMU,1375c	rc
igs1085	-1,8	-1,3	-1,8	**0,037**	0,315	0,117	SMU,1375c	SMU,1377c	fw
igs1085	1,1	-1,2	**2,6**	**0,010**	0,141	**0,000**	SMU,1375c	SMU,1377c	rc
igs1086	-1,2	-1,4	-1,7	0,766	0,562	0,441	SMU,1377c	SMU,1378	fw
igs1086	-1,6	1,6	**2,0**	**0,000**	**0,000**	**0,000**	SMU,1377c	SMU,1378	rc
igs1087	1,0	-1,1	-1,2	0,913	0,660	0,193	SMU,1378	SMU,1379	fw
igs1087	-1,2	1,1	1,1	0,324	0,658	0,579	SMU,1378	SMU,1379	rc
igs1088	-1,1	1,1	-1,1	0,378	0,166	0,595	SMU,1379	SMU,1381	fw
igs1088	1,0	-1,2	1,1	0,711	**0,029**	0,336	SMU,1379	SMU,1381	rc
igs1090	-1,2	1,4	-1,6	0,694	0,495	0,240	SMU,1382	SMU,1383	fw
igs1090	-1,3	-1,7	-1,1	0,061	**0,000**	0,099	SMU,1382	SMU,1383	rc
igs1092	-1,1	-1,2	1,2	0,853	0,704	0,706	SMU,1384	SMU,1386	fw
igs1092	1,1	-1,2	-1,0	**0,008**	**0,028**	0,743	SMU,1384	SMU,1386	rc
igs1093	-1,0	-1,1	-1,2	0,780	0,542	0,317	SMU,1386	SMU,1387	fw
igs1093	1,6	-1,9	1,1	**0,036**	**0,017**	0,722	SMU,1386	SMU,1387	rc
igs1094	-1,4	1,1	-1,1	0,125	0,670	0,650	SMU,1388	SMU,1389	fw
igs1094	1,1	1,0	-1,4	0,354	0,942	**0,029**	SMU,1388	SMU,1389	rc
igs1095	-1,2	1,6	1,1	0,181	**0,000**	0,463	SMU,1389	SMU,1390	fw
igs1095	-1,2	-1,1	1,1	0,196	0,386	0,475	SMU,1389	SMU,1390	rc
igs1096	1,1	-2,3	-1,3	0,713	**0,008**	0,059	SMU,1390	SMU,1391c	fw
igs1096	-2,0	-1,4	-2,7	**0,000**	**0,018**	**0,000**	SMU,1390	SMU,1391c	rc
igs1097	1,3	1,0	-1,0	0,625	0,957	0,987	SMU,1391c	SMU,1392c	fw
igs1097	-1,0	1,4	1,5	0,568	**0,015**	**0,003**	SMU,1391c	SMU,1392c	rc
igs1098	1,2	-1,3	-1,1	0,083	**0,007**	0,067	SMU,1392c	SMU,1393c	fw
igs1098	1,5	-1,1	1,9	**0,000**	0,575	**0,000**	SMU,1392c	SMU,1393c	rc
igs1099	1,4	-1,1	-1,2	0,130	0,476	0,386	SMU,1393c	SMU,1394	fw
igs1099	-1,1	1,2	-1,1	**0,025**	**0,031**	0,163	SMU,1393c	SMU,1394	rc
igs1100	-1,1	-1,1	1,1	0,642	0,389	0,109	SMU,1394	SMU,1395c	fw
igs1100	-1,2	1,5	-1,1	**0,009**	**0,000**	0,095	SMU,1394	SMU,1395c	rc
igs1101	1,2	1,2	-1,0	0,115	**0,031**	0,709	SMU,1395c	SMU,1396	fw
igs1101	1,0	1,5	1,1	0,436	**0,000**	0,245	SMU,1395c	SMU,1396	rc
igs1102	1,4	-1,3	1,1	0,180	0,285	0,590	SMU,1396	SMU,1397c	fw
igs1102	1,2	-1,7	1,3	**0,033**	**0,000**	0,068	SMU,1396	SMU,1397c	rc
igs1103	-1,1	-1,1	1,0	0,567	0,711	0,864	SMU,1397c	SMU,1398	fw
igs1103	-1,2	-1,1	1,3	0,530	0,740	0,382	SMU,1397c	SMU,1398	rc
igs1104	-1,3	-1,4	1,0	0,490	0,431	0,977	SMU,1398	SMU,1399	fw
igs1104	1,0	-1,0	1,0	0,840	0,932	0,921	SMU,1398	SMU,1399	rc
igs1105	-1,0	-1,3	-2,1	0,951	0,337	**0,029**	SMU,1399	SMU,1400c	fw
igs1105	-1,0	-1,3	-1,3	0,820	0,065	**0,001**	SMU,1399	SMU,1400c	rc
igs1106	-1,0	**4,0**	-1,4	0,998	**0,000**	0,299	SMU,1400c	SMU,1402c	fw
igs1106	-1,3	-1,4	-1,1	**0,002**	**0,000**	0,079	SMU,1400c	SMU,1402c	rc
igs1107	-1,4	-1,2	-1,2	**0,003**	0,097	0,197	SMU,1402c	SMU,1403c	fw
igs1107	1,3	1,1	1,3	**0,001**	0,475	**0,000**	SMU,1402c	SMU,1403c	rc
igs1109	1,0	-1,4	1,5	0,746	**0,000**	**0,000**	SMU,1405c	SMU,1406c	fw
igs1109	-1,0	1,1	1,2	0,968	0,798	0,767	SMU,1405c	SMU,1406c	rc
igs1110	1,4	-1,1	-1,3	**0,005**	0,171	**0,026**	SMU,1406c	SMU,1407c	fw
igs1110	1,2	-1,4	-2,2	0,677	0,440	0,115	SMU,1406c	SMU,1407c	rc
igs1111	-1,1	-1,6	1,1	0,540	0,071	0,658	SMU,1408c	SMU,1409c	fw
igs1111	-1,1	-1,9	1,1	0,206	**0,000**	0,376	SMU,1408c	SMU,1409c	rc
igs1112	1,3	1,1	-1,3	0,252	0,715	0,326	SMU,1409c	SMU,1410	fw
igs1112	-1,3	-1,1	1,0	**0,001**	0,228	0,979	SMU,1409c	SMU,1410	rc

igs1113	-1,1	1,1	1,2	0,421	0,284	0,168	SMU,1410	SMU,1411	fw
igs1113	-1,4	-1,3	-1,3	**0,017**	**0,001**	**0,035**	SMU,1410	SMU,1411	rc
igs1114	-1,1	-1,1	1,5	0,880	0,701	0,254	SMU,1411	SMU,1412c	fw
igs1114	1,5	-1,2	1,5	**0,017**	0,190	**0,015**	SMU,1411	SMU,1412c	rc
igs1115	1,2	1,4	-1,0	0,721	0,461	0,936	SMU,1412c	SMU,1414c	fw
igs1115	-1,0	1,3	-1,3	0,667	**0,020**	0,095	SMU,1412c	SMU,1414c	rc
igs1119	-1,3	-1,1	1,0	**0,000**	0,053	0,547	SMU,1418	SMU,1419	fw
igs1119	-1,0	1,0	-1,3	0,916	0,951	0,089	SMU,1418	SMU,1419	rc
igs1120	1,2	1,3	-1,2	0,132	0,093	0,177	SMU,1420	SMU,1421	fw
igs1120	1,2	1,1	-1,1	0,316	0,528	0,660	SMU,1420	SMU,1421	rc
igs1124	1,5	1,1	1,1	**0,003**	0,463	0,476	SMU,1424	SMU,1425	fw
igs1124	-1,1	-1,0	-1,3	0,365	0,868	0,060	SMU,1424	SMU,1425	rc
igs1125	-1,3	-1,6	1,5	0,503	0,381	0,302	SMU,1425	SMU,1426c	fw
igs1125	-1,4	-1,4	1,1	**0,027**	0,081	0,518	SMU,1425	SMU,1426c	rc
igs1126	1,4	-1,2	-1,1	0,422	0,715	0,759	SMU,1426c	SMU,1427c	fw
igs1126	-1,2	1,1	1,4	0,110	0,522	**0,024**	SMU,1426c	SMU,1427c	rc
igs1127	-1,7	-1,5	-1,5	0,164	0,328	0,250	SMU,1428c	SMU,1429	fw
igs1127	-1,4	-1,3	1,7	**0,002**	**0,024**	0,072	SMU,1428c	SMU,1429	rc
igs1128	1,1	1,3	-1,0	0,470	**0,006**	0,904	SMU,1430	SMU,1431c	fw
igs1128	1,1	1,1	-1,4	0,736	0,827	0,093	SMU,1430	SMU,1431c	rc
igs1129	-1,7	1,2	-1,4	0,131	0,683	0,518	SMU,1431c	SMU,1432c	fw
igs1129	-1,2	-1,3	-1,8	0,074	**0,000**	**0,000**	SMU,1431c	SMU,1432c	rc
igs1130	1,0	1,1	-1,1	0,883	0,725	0,467	SMU,1432c	SMU,1434c	fw
igs1130	1,1	-1,1	1,4	0,437	0,078	**0,000**	SMU,1432c	SMU,1434c	rc
igs1131	1,1	1,1	1,1	0,694	0,510	0,333	SMU,1434c	SMU,1435c	fw
igs1131	1,1	1,1	1,0	**0,009**	0,220	0,352	SMU,1434c	SMU,1435c	rc
igs1134	-1,0	1,2	-2,0	0,854	0,437	**0,001**	SMU,1437	SMU,1438c	fw
igs1134	1,2	-1,4	-1,2	0,146	0,056	0,142	SMU,1437	SMU,1438c	rc
igs1135	1,1	-1,1	1,1	0,600	0,712	0,528	SMU,1438c	SMU,1442c	fw
igs1135	-1,0	-1,1	-1,0	0,946	0,247	0,926	SMU,1438c	SMU,1442c	rc
igs1136	-1,2	-1,2	1,0	0,436	0,435	0,876	SMU,1442c	SMU,1443c	fw
igs1136	-2,1	-1,5	1,2	**0,000**	**0,012**	0,295	SMU,1442c	SMU,1443c	rc
igs1137	1,1	1,1	-1,1	0,745	0,700	0,389	SMU,1443c	SMU,1444c	fw
igs1137	1,0	1,0	1,1	0,860	0,865	0,647	SMU,1443c	SMU,1444c	rc
igs1138	-1,1	-1,0	-1,3	0,660	0,891	0,294	SMU,1444c	SMU,1445c	fw
igs1138	-1,1	1,4	-1,3	0,538	**0,000**	**0,003**	SMU,1444c	SMU,1445c	rc
igs1139	1,8	1,1	-1,5	**0,008**	0,315	**0,000**	SMU,1446c	SMU,1447c	fw
igs1139	1,0	1,0	-1,5	0,902	0,944	**0,008**	SMU,1446c	SMU,1447c	rc
igs1140	-1,1	1,2	-1,5	0,835	0,681	0,243	SMU,1447c	SMU,1449	fw
igs1140	-1,2	-1,4	-1,3	**0,037**	**0,000**	**0,002**	SMU,1447c	SMU,1449	rc
igs1141	1,2	1,0	-1,4	**0,039**	0,694	**0,000**	SMU,1449	SMU,1450	fw
igs1141	-1,2	-1,2	1,5	**0,001**	**0,002**	**0,000**	SMU,1449	SMU,1450	rc
igs1142	-1,1	-1,5	1,9	0,939	0,575	0,360	SMU,1450	SMU,1451	fw
igs1142	1,5	-1,4	1,2	0,251	0,345	0,545	SMU,1450	SMU,1451	rc
igs1144	-1,1	1,1	-1,9	0,904	0,894	0,248	SMU,1452	SMU,1453c	fw
igs1144	-1,2	1,4	-1,0	0,243	**0,000**	0,932	SMU,1452	SMU,1453c	rc
igs1145	1,1	1,1	-1,1	0,509	0,614	0,756	SMU,1453c	SMU,1454c	fw
igs1145	1,0	-1,2	-1,1	0,790	**0,033**	0,080	SMU,1453c	SMU,1454c	rc
igs1146	1,1	1,1	1,0	0,729	0,560	0,835	SMU,1454c	SMU,1455	fw
igs1146	1,3	1,1	1,0	**0,012**	0,325	0,477	SMU,1454c	SMU,1455	rc
igs1147	-1,1	-1,4	1,1	0,672	0,106	0,436	SMU,1456c	SMU,1457	fw
igs1147	-1,0	1,2	-1,4	0,737	0,084	**0,001**	SMU,1456c	SMU,1457	rc
igs1148	-1,1	-1,2	-1,3	0,675	0,285	0,098	SMU,1457	SMU,1459c	fw
igs1148	1,0	-1,0	1,2	0,302	0,567	**0,000**	SMU,1457	SMU,1459c	rc
igs1149	-1,2	-1,2	1,0	0,693	0,591	0,961	SMU,1459c	SMU,1460	fw
igs1149	1,2	-1,1	1,4	**0,023**	**0,009**	**0,000**	SMU,1459c	SMU,1460	rc
igs1150	-1,0	-1,2	-1,3	0,864	0,134	**0,001**	SMU,1460	SMU,1461	fw
igs1150	1,2	-1,5	1,3	**0,012**	**0,000**	**0,000**	SMU,1460	SMU,1461	rc
igs1151	-1,0	1,0	-1,3	0,931	0,980	0,552	SMU,1461	SMU,1462c	fw
igs1151	-1,3	-1,1	1,9	**0,000**	0,083	**0,000**	SMU,1461	SMU,1462c	rc
igs1152	**2,3**	-2,2	1,6	**0,013**	**0,007**	**0,035**	SMU,1462c	SMU,1463c	fw
igs1152	-1,1	**2,4**	-1,8	**0,009**	**0,000**	**0,002**	SMU,1462c	SMU,1463c	rc
igs1154	1,2	-1,0	1,2	0,349	0,976	0,528	SMU,1466	SMU,1467	fw
igs1154	1,4	**2,1**	-1,2	**0,004**	**0,000**	0,254	SMU,1466	SMU,1467	rc
igs1155	-1,1	-1,2	-1,1	0,381	0,088	0,560	SMU,1467	SMU,1470c	fw
igs1155	-1,0	-1,1	-1,0	0,423	**0,011**	0,901	SMU,1467	SMU,1470c	rc

igs1156	-1,2	-1,2	1,0	0,268	0,135	0,919	SMU,1470c	SMU,1471c	fw
igs1156	1,0	1,2	1,2	0,807	**0,006**	0,165	SMU,1470c	SMU,1471c	rc
igs1157	1,3	1,1	1,3	0,338	0,660	0,371	SMU,1471c	SMU,1472	fw
igs1157	1,6	**2,0**	-1,1	**0,001**	**0,000**	0,651	SMU,1471c	SMU,1472	rc
igs1158	1,3	1,1	-2,0	0,276	0,653	**0,028**	SMU,1474c	SMU,1475c	fw
igs1158	-1,0	-1,2	1,3	0,849	0,387	**0,021**	SMU,1474c	SMU,1475c	rc
igs1159	-1,1	-1,1	-1,9	0,831	0,909	0,231	SMU,1476c	SMU,1477	fw
igs1159	-1,3	-1,2	1,2	0,060	**0,017**	**0,007**	SMU,1476c	SMU,1477	rc
igs1160	-1,2	1,0	-1,2	0,204	0,797	0,085	SMU,1477	SMU,1479	fw
igs1160	1,7	1,2	-1,6	0,476	0,797	0,559	SMU,1477	SMU,1479	rc
igs1161	-1,1	-1,1	-1,2	0,639	0,826	0,463	SMU,1479	SMU,1480	fw
igs1161	-1,1	1,1	1,5	0,545	0,652	**0,039**	SMU,1479	SMU,1480	rc
igs1162	1,0	-1,3	-1,8	0,949	0,138	**0,022**	SMU,1480	SMU,1482c	fw
igs1162	1,0	-1,1	1,7	0,894	0,614	**0,000**	SMU,1480	SMU,1482c	rc
igs1163	1,1	-1,2	-1,1	0,807	0,463	0,484	SMU,1483c	SMU,1484c	fw
igs1163	-1,0	1,2	1,1	0,614	0,212	0,304	SMU,1483c	SMU,1484c	rc
igs1165	1,6	1,4	1,2	**0,000**	**0,010**	**0,016**	SMU,1486c	SMU,1487	fw
igs1165	1,2	**2,0**	1,1	**0,017**	**0,000**	0,303	SMU,1486c	SMU,1487	rc
igs1166	-1,1	-1,2	-1,1	0,859	0,083	0,473	SMU,1487	SMU,1488c	fw
igs1166	-1,1	1,3	-1,2	0,255	0,075	0,076	SMU,1487	SMU,1488c	rc
igs1168	1,2	-1,2	-1,3	0,493	0,380	0,097	SMU,1489	SMU,1490	fw
igs1168	1,0	1,2	-1,0	0,911	**0,040**	0,795	SMU,1489	SMU,1490	rc
igs1175	-1,1	-1,4	1,2	0,760	0,065	0,228	SMU,1496	SMU,1498	fw
igs1175	1,1	1,0	1,1	0,343	0,824	0,534	SMU,1496	SMU,1498	rc
igs1176	1,2	-1,7	-1,3	0,615	**0,029**	0,197	SMU,1498	SMU,1499	fw
igs1176	1,5	-1,8	-1,1	**0,018**	**0,000**	0,698	SMU,1498	SMU,1499	rc
igs1177	-1,0	1,0	-1,0	0,839	0,919	0,636	SMU,1500	SMU,1502c	fw
igs1177	-1,0	-1,1	1,1	0,909	0,379	0,260	SMU,1500	SMU,1502c	rc
igs1178	1,1	-1,0	-1,5	0,722	0,938	0,220	SMU,1502c	SMU,1504c	fw
igs1178	1,1	-1,1	-1,6	0,174	0,455	**0,000**	SMU,1502c	SMU,1504c	rc
igs1179	1,2	1,0	1,2	**0,001**	0,371	0,064	SMU,1504c	SMU,1505c	fw
igs1179	1,0	1,0	-1,1	0,799	0,560	0,170	SMU,1504c	SMU,1505c	rc
igs1180	1,2	1,0	1,0	0,288	0,987	0,859	SMU,1505c	SMU,1506c	fw
igs1180	1,1	1,0	-1,1	0,416	0,708	0,249	SMU,1505c	SMU,1506c	rc
igs1181	-1,4	-1,2	-1,0	0,369	0,684	0,948	SMU,1508c	SMU,1509	fw
igs1181	-1,2	1,1	-1,1	0,112	0,687	0,656	SMU,1508c	SMU,1509	rc
igs1182	1,1	1,1	1,5	0,682	0,679	0,045	SMU,1509	SMU,1510	fw
igs1182	1,0	1,0	-1,4	0,833	0,867	0,083	SMU,1509	SMU,1510	rc
igs1184	-1,0	-1,2	-1,4	0,907	0,103	**0,012**	SMU,1511c	SMU,1512	fw
igs1184	-1,2	-1,3	-1,3	**0,000**	**0,003**	**0,042**	SMU,1511c	SMU,1512	rc
igs1185	1,3	1,0	-1,1	0,275	0,859	0,849	SMU,1512	SMU,1513	fw
igs1185	-1,2	1,1	1,2	**0,000**	0,121	**0,008**	SMU,1512	SMU,1513	rc
igs1186	-1,0	-1,2	-1,7	0,973	0,499	0,118	SMU,1514	SMU,1515	fw
igs1186	1,3	1,1	1,0	**0,000**	0,245	0,771	SMU,1514	SMU,1515	rc
igs1187	1,1	1,4	-1,3	0,811	0,110	0,157	SMU,1517	SMU,1519	fw
igs1187	1,3	**2,2**	1,5	0,198	**0,000**	0,072	SMU,1517	SMU,1519	rc
igs1190	**2,5**	-1,0	1,6	**0,042**	0,927	0,120	SMU,1522	SMU,1523	fw
igs1190	-1,2	-1,8	1,3	0,735	0,268	0,560	SMU,1522	SMU,1523	rc
igs1191	-1,1	1,1	-1,1	0,121	0,291	0,302	SMU,1523	SMU,1524c	fw
igs1191	1,4	1,3	1,3	**0,014**	**0,006**	**0,000**	SMU,1523	SMU,1524c	rc
igs1193	1,5	1,1	1,1	0,442	0,804	0,761	SMU,1525	SMU,1526c	fw
igs1193	1,5	-1,1	-1,6	**0,049**	0,338	0,055	SMU,1525	SMU,1526c	rc
igs1194	-1,3	-1,1	-1,0	0,313	0,846	0,968	SMU,1526c	SMU,1527	fw
igs1194	-1,0	1,1	1,0	0,918	0,573	0,825	SMU,1526c	SMU,1527	rc
igs1200	-1,3	-1,3	-2,2	**0,012**	**0,011**	**0,000**	SMU,1533	SMU,1534	fw
igs1200	1,2	1,1	**2,0**	**0,000**	0,102	**0,000**	SMU,1533	SMU,1534	rc
igs1201	1,1	-1,2	-1,1	0,690	0,431	0,713	SMU,1534	SMU,1535	fw
igs1201	1,6	1,4	1,6	**0,000**	0,071	**0,000**	SMU,1534	SMU,1535	rc
igs1202	-1,4	-1,5	1,3	0,427	0,349	0,552	SMU,1535	SMU,1536	fw
igs1202	-1,0	-1,3	1,5	0,914	0,116	**0,043**	SMU,1535	SMU,1536	rc
igs1204	1,1	1,0	-1,3	0,724	0,809	0,170	SMU,1539	SMU,1541	fw
igs1204	-1,2	-1,1	-1,2	**0,009**	0,128	0,112	SMU,1539	SMU,1541	rc
igs1205	1,2	1,0	-1,2	0,335	0,887	0,129	SMU,1541	SMU,1542c	fw
igs1205	-2,0	-1,0	1,4	**0,000**	0,939	**0,000**	SMU,1541	SMU,1542c	rc
igs1207	1,1	-1,2	1,4	0,567	0,441	0,046	SMU,1543	SMU,1545c	fw
igs1207	1,3	-1,0	1,7	**0,017**	0,787	**0,000**	SMU,1543	SMU,1545c	rc

igs1208	1,1	-1,5	-1,7	0,878	0,468	0,509	SMU,1545c	SMU,1546	fw
igs1208	1,4	-1,0	-1,7	**0,044**	0,998	**0,007**	SMU,1545c	SMU,1546	rc
igs1209	1,1	-1,1	-1,1	0,062	0,234	**0,038**	SMU,1546	SMU,1547c	fw
igs1209	-1,0	1,0	-1,2	0,945	0,934	0,688	SMU,1546	SMU,1547c	rc
igs1210	-1,0	1,0	-1,2	0,668	0,963	**0,003**	SMU,1548c	SMU,1550c	fw
igs1210	-1,1	1,1	-1,2	0,065	0,144	**0,036**	SMU,1548c	SMU,1550c	rc
igs1211	1,2	1,0	-1,4	**0,001**	0,464	**0,001**	SMU,1551c	SMU,1552c	fw
igs1211	-1,3	-1,0	1,2	0,098	0,358	**0,030**	SMU,1551c	SMU,1552c	rc
igs1213	-1,4	-1,1	-1,0	0,336	0,729	0,993	SMU,1554c	SMU,1555c	fw
igs1213	-1,1	-1,1	-1,1	**0,010**	0,541	0,666	SMU,1554c	SMU,1555c	rc
igs1216	-1,0	1,0	1,0	0,836	0,759	0,908	SMU,1558c	SMU,1560	fw
igs1216	1,1	1,1	-1,1	0,280	0,688	0,256	SMU,1558c	SMU,1560	rc
igs1217	-1,2	-2,0	1,1	0,247	**0,007**	0,303	SMU,1560	SMU,1561	fw
igs1217	1,7	-1,0	-1,3	0,157	0,990	0,341	SMU,1560	SMU,1561	rc
igs1219	1,4	-1,1	-1,6	0,115	0,696	**0,043**	SMU,1562	SMU,1563	fw
igs1219	-1,0	-1,1	2,0	0,925	0,263	**0,000**	SMU,1562	SMU,1563	rc
igs1220	1,1	-1,1	-1,2	0,786	0,710	0,630	SMU,1563	SMU,1564	fw
igs1220	1,5	-1,0	-1,5	**0,000**	0,907	**0,000**	SMU,1563	SMU,1564	rc
igs1221	1,2	1,5	1,0	0,771	0,301	0,957	SMU,1565	SMU,1566	fw
igs1221	1,0	-1,8	1,3	0,684	**0,000**	**0,016**	SMU,1565	SMU,1566	rc
igs1222	-1,0	-1,2	1,1	0,937	0,341	0,593	SMU,1566	SMU,1568	fw
igs1222	-1,4	-1,3	1,2	**0,035**	0,081	0,319	SMU,1566	SMU,1568	rc
igs1223	1,3	1,1	-2,2	0,071	0,542	**0,003**	SMU,1568	SMU,1569	fw
igs1223	-1,2	1,1	1,7	**0,038**	0,519	**0,001**	SMU,1568	SMU,1569	rc
igs1225	-1,0	-1,1	1,4	0,978	0,912	0,598	SMU,1571	SMU,1572	fw
igs1225	-1,0	-1,0	1,8	0,884	0,805	**0,016**	SMU,1571	SMU,1572	rc
igs1226	1,4	-2,1	-1,0	0,423	0,075	0,938	SMU,1572	SMU,1573	fw
igs1226	-1,0	1,5	-1,6	0,766	**0,000**	**0,001**	SMU,1572	SMU,1573	rc
igs1227	-1,2	-1,2	1,1	0,391	0,505	0,824	SMU,1573	SMU,1574c	fw
igs1227	1,0	-1,0	1,1	0,452	0,983	0,335	SMU,1573	SMU,1574c	rc
igs1228	1,1	-1,1	1,1	0,573	0,570	0,631	SMU,1574c	SMU,1575c	fw
igs1228	-1,0	1,5	1,1	0,457	**0,000**	0,281	SMU,1574c	SMU,1575c	rc
igs1229	1,0	1,4	**2,5**	0,987	**0,001**	**0,001**	SMU,1576c	SMU,1577c	fw
igs1229	**2,0**	-2,9	**2,9**	**0,001**	**0,000**	**0,000**	SMU,1576c	SMU,1577c	rc
igs1230	1,2	-1,0	-1,5	0,133	0,794	**0,000**	SMU,1577c	SMU,1578	fw
igs1230	1,1	1,1	1,1	0,359	0,704	0,633	SMU,1577c	SMU,1578	rc
igs1231	-1,4	-1,1	**2,5**	0,348	0,676	**0,007**	SMU,1579	SMU,1581	fw
igs1231	2,0	-1,2	-3,1	**0,000**	0,233	**0,000**	SMU,1579	SMU,1581	rc
igs1232	1,2	1,3	1,5	**0,001**	**0,000**	**0,000**	SMU,1582c	SMU,1584c	fw
igs1232	-1,1	1,0	1,4	0,222	0,778	**0,009**	SMU,1582c	SMU,1584c	rc
igs1233	-1,2	-1,1	-1,4	0,127	0,413	**0,005**	SMU,1584c	SMU,1585c	fw
igs1233	-1,3	-1,4	1,1	**0,010**	**0,022**	0,441	SMU,1584c	SMU,1585c	rc
igs1234	1,1	-1,2	-1,5	0,798	0,663	0,453	SMU,1585c	SMU,1586	fw
igs1234	-1,2	-1,0	1,1	**0,013**	0,850	0,176	SMU,1585c	SMU,1586	rc
igs1235	1,1	-1,0	1,0	0,484	0,814	0,953	SMU,1587c	SMU,1588c	fw
igs1235	-1,0	-1,0	-1,1	0,642	0,620	**0,013**	SMU,1587c	SMU,1588c	rc
igs1237	-1,0	1,3	-1,5	0,794	0,053	**0,001**	SMU,1589c	SMU,1590	fw
igs1237	-1,0	-1,3	1,3	0,620	**0,000**	**0,001**	SMU,1589c	SMU,1590	rc
igs1238	-1,4	1,1	-1,3	**0,022**	0,684	0,162	SMU,1590	SMU,1591	fw
igs1238	-1,2	1,3	1,2	0,113	**0,000**	0,106	SMU,1590	SMU,1591	rc
igs1239	1,0	-1,1	1,3	0,959	0,900	0,690	SMU,1591	SMU,1592	fw
igs1239	-1,2	-2,1	-1,1	0,085	**0,000**	0,543	SMU,1591	SMU,1592	rc
igs1240	1,1	-1,4	1,3	0,894	0,294	0,372	SMU,1592	SMU,1593c	fw
igs1240	-1,3	-1,4	1,2	0,420	0,210	0,398	SMU,1592	SMU,1593c	rc
igs1241	-1,1	1,1	1,1	0,837	0,898	0,805	SMU,1593c	SMU,1595	fw
igs1241	-1,2	1,2	1,1	**0,004**	**0,014**	0,557	SMU,1593c	SMU,1595	rc
igs1242	1,0	-1,1	-1,3	0,929	0,814	0,500	SMU,1595	SMU,1596	fw
igs1242	-1,0	-1,1	-1,1	0,867	0,480	0,338	SMU,1595	SMU,1596	rc
igs1245	-1,1	1,1	-1,0	0,075	0,055	0,815	SMU,1599	SMU,1600	fw
igs1245	1,4	-1,1	1,5	**0,000**	0,110	**0,008**	SMU,1599	SMU,1600	rc
igs1246	-1,0	-1,1	-1,3	0,394	0,597	**0,014**	SMU,1600	SMU,1601	fw
igs1246	-1,1	-1,2	1,2	0,279	**0,012**	0,082	SMU,1600	SMU,1601	rc
igs1247	1,0	1,0	-1,1	0,688	0,726	0,362	SMU,1601	SMU,1602	fw
igs1247	-1,0	1,0	-1,1	0,870	0,908	0,666	SMU,1601	SMU,1602	rc
igs1248	-1,4	-1,4	-1,4	0,052	0,052	**0,019**	SMU,1602	SMU,1603	fw
igs1248	-1,8	-1,1	1,1	**0,001**	0,516	0,132	SMU,1602	SMU,1603	rc

igs1249	1,1	-1,1	1,0	0,309	0,311	0,733	SMU,1603	SMU,1604c	fw
igs1249	-1,0	1,4	1,0	0,940	**0,025**	0,713	SMU,1603	SMU,1604c	rc
igs1250	1,5	-1,2	1,3	0,245	0,581	0,265	SMU,1604c	SMU,1605	fw
igs1250	-1,1	-1,6	**2,5**	0,449	**0,000**	**0,000**	SMU,1604c	SMU,1605	rc
igs1251	-1,4	-1,3	-3,2	0,098	**0,034**	**0,006**	SMU,1605	SMU,1606	fw
igs1251	-1,0	1,1	1,0	0,856	0,322	0,815	SMU,1605	SMU,1606	rc
igs1252	1,1	-1,0	-1,2	0,803	0,868	0,512	SMU,1607	SMU,1609c	fw
igs1252	1,0	-1,2	-1,3	0,703	0,077	**0,001**	SMU,1607	SMU,1609c	rc
igs1253	1,3	1,3	-1,8	0,365	0,320	0,133	SMU,1609c	SMU,1610	fw
igs1253	1,0	-1,1	1,1	0,752	0,340	0,426	SMU,1609c	SMU,1610	rc
igs1254	1,1	1,1	-1,4	0,881	0,853	0,335	SMU,1611c	SMU,1612c	fw
igs1254	1,3	-2,1	1,0	**0,004**	**0,000**	0,951	SMU,1611c	SMU,1612c	rc
igs1255	1,6	1,4	-1,4	**0,011**	**0,014**	0,055	SMU,1614	SMU,1615c	fw
igs1255	-1,1	-1,0	1,1	0,060	0,934	0,309	SMU,1614	SMU,1615c	rc
igs1256	1,3	1,1	-1,5	**0,005**	0,203	**0,000**	SMU,1615c	SMU,1616c	fw
igs1256	-1,1	1,0	1,1	**0,037**	0,653	0,180	SMU,1615c	SMU,1616c	rc
igs1257	-1,3	-1,3	-1,3	0,446	0,381	0,373	SMU,1616c	SMU,1617	fw
igs1257	-1,1	-1,5	1,8	0,165	**0,000**	**0,000**	SMU,1616c	SMU,1617	rc
igs1259	1,0	1,1	-1,2	0,950	0,717	0,400	SMU,1619c	SMU,1620	fw
igs1259	-1,0	-1,2	1,1	0,411	**0,002**	0,069	SMU,1619c	SMU,1620	rc
igs1260	-1,5	1,3	-1,7	0,069	0,452	0,115	SMU,1620	SMU,1621c	fw
igs1260	1,4	1,1	1,5	**0,000**	**0,046**	**0,000**	SMU,1620	SMU,1621c	rc
igs1261	1,1	1,1	-1,0	0,575	0,370	0,812	SMU,1622	SMU,1623c	fw
igs1261	1,3	-1,3	-1,2	**0,000**	**0,039**	**0,004**	SMU,1622	SMU,1623c	rc
igs1262	-1,0	-1,3	-1,5	0,968	0,539	0,239	SMU,1623c	SMU,1624	fw
igs1262	-1,1	-1,1	1,6	0,220	0,487	**0,015**	SMU,1623c	SMU,1624	rc
igs1263	-1,1	-1,3	1,1	0,321	**0,046**	0,545	SMU,1624	SMU,1625	fw
igs1263	-1,2	-1,1	-1,1	**0,008**	**0,026**	**0,004**	SMU,1624	SMU,1625	rc
igs1264	1,3	-1,1	-1,9	0,663	0,848	0,253	SMU,1625	SMU,1626	fw
igs1264	**2,2**	-1,2	-1,2	**0,000**	0,544	0,202	SMU,1625	SMU,1626	rc
igs1265	1,6	1,0	1,5	**0,001**	0,645	**0,000**	SMU,1626	SMU,1627	fw
igs1265	-1,0	-1,0	1,2	0,907	0,833	**0,042**	SMU,1626	SMU,1627	rc
igs1266	1,1	1,1	1,2	0,929	0,958	0,866	SMU,1627	SMU,1628	fw
igs1266	-1,0	-1,4	2,0	0,594	0,069	**0,000**	SMU,1627	SMU,1628	rc
igs1267	1,1	-1,1	-1,1	0,503	0,514	0,328	SMU,1628	SMU,1629c	fw
igs1267	-1,2	-1,2	-1,4	0,466	0,327	**0,026**	SMU,1628	SMU,1629c	rc
igs1268	1,0	-2,4	1,1	0,968	0,056	0,762	SMU,1629c	SMU,1631	fw
igs1268	1,4	-1,3	1,1	**0,000**	**0,039**	0,686	SMU,1629c	SMU,1631	rc
igs1269	-1,7	-1,5	-1,2	**0,007**	**0,004**	0,309	SMU,1631	SMU,1632	fw
igs1269	-1,6	-1,4	1,2	**0,011**	0,287	0,393	SMU,1631	SMU,1632	rc
igs1273	-1,1	-1,5	-1,0	0,718	0,346	0,938	SMU,1635	SMU,1636c	fw
igs1273	-1,4	1,4	-1,0	**0,032**	**0,019**	0,960	SMU,1635	SMU,1636c	rc
igs1274	-1,5	-1,3	1,3	**0,007**	0,061	0,058	SMU,1636c	SMU,1637c	fw
igs1274	-1,1	-1,1	-1,1	0,616	0,560	0,352	SMU,1636c	SMU,1637c	rc
igs1275	-1,2	1,2	-1,3	0,711	0,559	0,399	SMU,1637c	SMU,1638c	fw
igs1275	-1,3	1,1	-1,3	**0,001**	0,183	0,122	SMU,1637c	SMU,1638c	rc
igs1276	1,2	-1,1	-1,2	0,707	0,776	0,655	SMU,1638c	SMU,1639	fw
igs1276	1,1	1,2	1,1	0,255	0,085	0,180	SMU,1638c	SMU,1639	rc
igs1277	-1,0	-1,0	-1,1	0,920	0,995	0,705	SMU,1639	SMU,1641c	fw
igs1277	1,1	-1,1	-1,0	0,192	**0,042**	0,402	SMU,1639	SMU,1641c	rc
igs1278	-1,4	-1,9	-1,6	0,700	0,459	0,593	SMU,1641c	SMU,1642c	fw
igs1278	1,4	-1,1	1,4	**0,000**	0,499	**0,000**	SMU,1641c	SMU,1642c	rc
igs1279	1,1	1,1	-1,0	0,622	0,457	0,858	SMU,1642c	SMU,1643c	fw
igs1279	1,1	-1,3	1,1	0,246	**0,002**	**0,017**	SMU,1642c	SMU,1643c	rc
igs1280	1,1	-1,2	1,1	0,528	0,287	0,579	SMU,1643c	SMU,1644c	fw
igs1280	1,1	1,1	1,1	**0,038**	**0,002**	0,206	SMU,1643c	SMU,1644c	rc
igs1281	1,5	-1,1	1,2	0,258	0,846	0,562	SMU,1644c	SMU,1645	fw
igs1281	1,2	-1,1	1,2	0,351	0,581	0,543	SMU,1644c	SMU,1645	rc
igs1282	-1,0	-1,2	1,0	0,751	0,124	0,937	SMU,1645	SMU,1646c	fw
igs1282	-1,7	-1,4	1,0	0,234	0,499	0,987	SMU,1645	SMU,1646c	rc
igs1283	-1,2	1,1	1,1	0,528	0,833	0,588	SMU,1647c	SMU,1648c	fw
igs1283	1,3	-1,4	1,7	**0,000**	**0,010**	**0,000**	SMU,1647c	SMU,1648c	rc
igs1284	-1,0	1,2	-1,5	0,888	0,097	**0,000**	SMU,1648c	SMU,1649	fw
igs1284	1,1	-1,0	-1,2	0,811	0,971	0,675	SMU,1648c	SMU,1649	rc
igs1285	1,0	-1,1	-1,4	0,656	**0,043**	**0,038**	SMU,1649	SMU,1650	fw
igs1285	-1,1	1,1	-1,0	0,612	0,779	0,883	SMU,1649	SMU,1650	rc

igs1286	-1,7	-1,1	-1,2	**0,047**	0,594	0,406	SMU,1650	SMU,1651	fw
igs1286	-1,1	1,0	1,1	0,080	0,912	0,631	SMU,1650	SMU,1651	rc
igs1287	-1,3	1,4	-1,5	**0,011**	**0,004**	**0,039**	SMU,1651	SMU,1652	fw
igs1287	1,2	-1,1	1,5	0,116	0,666	0,054	SMU,1651	SMU,1652	rc
igs1288	1,5	1,0	-1,7	0,240	0,980	0,184	SMU,1652	SMU,1653	fw
igs1288	-1,1	1,0	1,2	**0,037**	0,411	**0,005**	SMU,1652	SMU,1653	rc
igs1289	1,2	1,1	-1,9	0,435	0,799	**0,022**	SMU,1653	SMU,1654c	fw
igs1289	-1,2	-1,1	-1,0	0,092	0,511	0,785	SMU,1653	SMU,1654c	rc
igs1290	1,1	-1,2	-1,8	0,537	0,493	**0,036**	SMU,1654c	SMU,1655c	fw
igs1290	-1,1	-1,0	1,1	0,232	0,817	0,195	SMU,1654c	SMU,1655c	rc
igs1291	-1,1	-1,1	-1,2	0,344	0,383	0,077	SMU,1655c	SMU,1656	fw
igs1291	-1,2	1,2	-1,2	0,063	**0,029**	0,098	SMU,1655c	SMU,1656	rc
igs1292	1,3	1,3	-1,1	0,419	0,361	0,636	SMU,1656	SMU,1657c	fw
igs1292	1,0	-1,1	1,0	0,799	0,526	0,876	SMU,1656	SMU,1657c	rc
igs1294	1,0	-1,6	-1,2	0,854	**0,016**	0,269	SMU,1658	SMU,1659c	fw
igs1294	-1,1	-1,1	-1,0	**0,025**	0,291	0,938	SMU,1658	SMU,1659c	rc
igs1297	-1,5	-2,0	1,1	0,235	**0,044**	0,846	SMU,1663	SMU,1664c	fw
igs1297	1,5	1,3	-1,8	**0,009**	0,176	**0,001**	SMU,1663	SMU,1664c	rc
igs1298	-1,1	-1,4	-1,8	0,782	0,102	**0,002**	SMU,1664c	SMU,1665	fw
igs1298	1,2	-1,4	**2,3**	**0,007**	**0,019**	**0,000**	SMU,1664c	SMU,1665	rc
igs1300	1,3	-1,3	1,4	0,471	0,584	0,389	SMU,1668	SMU,1669	fw
igs1300	-1,4	1,1	1,7	**0,002**	0,286	**0,003**	SMU,1668	SMU,1669	rc
igs1301	1,1	-1,9	-1,2	0,682	**0,025**	0,410	SMU,1669	SMU,1670c	fw
igs1301	-1,4	-1,5	**2,1**	0,051	**0,014**	**0,001**	SMU,1669	SMU,1670c	rc
igs1303	1,0	1,2	-1,7	0,969	0,654	0,177	SMU,1671c	SMU,1672	fw
igs1303	-1,3	-1,3	1,5	**0,000**	**0,001**	**0,004**	SMU,1671c	SMU,1672	rc
igs1304	1,2	1,0	-1,0	0,457	0,995	0,880	SMU,1672	SMU,1673	fw
igs1304	-1,0	1,1	1,1	0,175	0,138	**0,007**	SMU,1672	SMU,1673	rc
igs1305	-1,0	1,1	1,0	0,899	0,339	0,791	SMU,1673	SMU,1674	fw
igs1305	1,0	-1,0	1,1	0,466	0,868	0,280	SMU,1673	SMU,1674	rc
igs1307	-1,2	1,0	-1,0	0,218	0,887	0,924	SMU,1675	SMU,1676c	fw
igs1307	1,1	1,0	-1,1	0,091	0,920	0,428	SMU,1675	SMU,1676c	rc
igs1308	1,3	-1,2	1,3	0,634	0,644	0,520	SMU,1676c	SMU,1677	fw
igs1308	1,3	-1,2	-1,5	**0,000**	0,350	**0,022**	SMU,1676c	SMU,1677	rc
igs1309	-1,2	-1,1	-1,2	0,420	0,544	0,408	SMU,1677	SMU,1678	fw
igs1309	1,1	-1,2	1,3	0,592	0,353	0,156	SMU,1677	SMU,1678	rc
igs1310	-1,5	1,4	-1,1	0,107	**0,046**	0,732	SMU,1678	SMU,1679c	fw
igs1310	-1,5	1,3	-1,4	**0,003**	**0,018**	**0,025**	SMU,1678	SMU,1679c	rc
igs1312	1,1	1,0	1,0	0,887	0,977	0,952	SMU,1681c	SMU,1682c	fw
igs1312	1,8	1,1	1,1	**0,002**	0,439	0,500	SMU,1681c	SMU,1682c	rc
igs1313	-1,3	-1,5	-2,2	0,198	**0,001**	**0,000**	SMU,1682c	SMU,1683c	fw
igs1313	-1,3	1,9	-1,2	**0,046**	**0,000**	**0,014**	SMU,1682c	SMU,1683c	rc
igs1314	1,3	1,1	-1,2	0,625	0,873	0,732	SMU,1683c	SMU,1685c	fw
igs1314	1,2	-1,2	-1,4	0,431	0,470	0,075	SMU,1683c	SMU,1685c	rc
igs1315	-1,2	-1,3	-1,1	0,662	0,446	0,804	SMU,1685c	SMU,1687	fw
igs1315	-1,4	-1,0	1,1	**0,005**	0,712	0,555	SMU,1685c	SMU,1687	rc
igs1316	-1,5	-1,1	-2,3	0,158	0,619	**0,004**	SMU,1687	SMU,1688	fw
igs1316	-1,2	-1,2	1,0	**0,033**	**0,031**	0,390	SMU,1687	SMU,1688	rc
igs1318	-1,0	-1,2	-1,2	0,867	0,333	0,387	SMU,1691	SMU,1692	fw
igs1318	1,1	-1,4	-1,1	**0,031**	**0,000**	0,338	SMU,1691	SMU,1692	rc
igs1319	**2,3**	1,2	-1,7	**0,027**	0,582	0,168	SMU,1692	SMU,1693	fw
igs1319	1,4	-1,2	1,1	**0,005**	0,169	0,399	SMU,1692	SMU,1693	rc
igs1320	-1,1	-1,2	-1,4	0,910	0,830	0,611	SMU,1693	SMU,1694c	fw
igs1320	1,4	1,2	1,5	**0,010**	**0,036**	**0,007**	SMU,1693	SMU,1694c	rc
igs1321	1,4	1,1	-1,0	0,549	0,823	0,979	SMU,1694c	SMU,1695	fw
igs1321	-1,0	-1,6	1,5	0,907	0,223	0,153	SMU,1694c	SMU,1695	rc
igs1322	-1,1	1,2	-3,3	0,739	**0,028**	**0,000**	SMU,1695	SMU,1697c	fw
igs1322	-1,6	1,0	-2,4	**0,001**	0,988	**0,000**	SMU,1695	SMU,1697c	rc
igs1323	-1,2	1,3	-1,1	0,124	0,057	0,249	SMU,1699c	SMU,1700c	fw
igs1323	-1,3	-1,9	-1,9	**0,002**	**0,000**	**0,000**	SMU,1699c	SMU,1700c	rc
igs1324	-1,1	1,1	1,2	0,787	0,639	0,622	SMU,1701c	SMU,1702c	fw
igs1324	1,4	-1,7	1,0	**0,001**	**0,000**	0,855	SMU,1701c	SMU,1702c	rc
igs1325	1,2	1,3	1,1	0,701	0,558	0,912	SMU,1703c	SMU,1704	fw
igs1325	-1,1	1,0	-1,1	0,307	0,883	0,427	SMU,1703c	SMU,1704	rc
igs1326	-1,3	-1,3	-1,2	0,343	0,251	0,187	SMU,1706	SMU,1707c	fw
igs1326	-1,2	-1,3	1,2	0,116	0,307	0,205	SMU,1706	SMU,1707c	rc

igs1327	1,3	1,0	1,2	0,562	0,920	0,710	SMU,1707c	SMU,1708	fw
igs1327	1,0	1,2	-1,1	0,974	0,215	0,761	SMU,1707c	SMU,1708	rc
igs1329	1,7	1,1	1,1	0,317	0,880	0,852	SMU,1709	SMU,1710c	fw
igs1329	-1,6	1,3	1,5	**0,034**	**0,040**	**0,004**	SMU,1709	SMU,1710c	rc
igs1330	-1,2	-1,3	-1,3	**0,036**	**0,029**	0,047	SMU,1718	SMU,1719c	fw
igs1330	1,2	-1,2	-1,0	**0,000**	0,150	0,862	SMU,1718	SMU,1719c	rc
igs1331	-1,4	-1,3	1,1	0,066	0,212	0,390	SMU,1719c	SMU,1721c	fw
igs1331	1,2	-1,1	-1,1	**0,048**	0,498	0,490	SMU,1719c	SMU,1721c	rc
igs1332	-1,0	-1,1	-1,4	0,900	0,675	0,255	SMU,1721c	SMU,1722c	fw
igs1332	-1,1	-1,1	1,0	0,421	0,236	0,932	SMU,1721c	SMU,1722c	rc
igs1334	-1,1	1,1	-1,1	0,691	0,778	0,787	SMU,1723c	SMU,1724c	fw
igs1334	-1,1	-1,2	1,7	**0,014**	**0,005**	**0,006**	SMU,1723c	SMU,1724c	rc
igs1335	**2,2**	-1,4	-1,0	**0,008**	**0,039**	0,999	SMU,1724c	SMU,1725	fw
igs1335	1,2	-1,2	-1,3	0,182	0,278	**0,031**	SMU,1724c	SMU,1725	rc
igs1336	1,3	-2,8	1,1	**0,019**	**0,000**	0,626	SMU,1725	SMU,1727	fw
igs1336	1,0	1,7	1,0	0,902	**0,029**	0,868	SMU,1725	SMU,1727	rc
igs1337	1,0	-1,1	-1,1	0,890	0,846	0,748	SMU,1727	SMU,1728	fw
igs1337	-1,5	-1,1	1,2	0,114	0,603	0,456	SMU,1727	SMU,1728	rc
igs1338	1,0	-1,3	1,5	0,875	0,170	**0,025**	SMU,1728	SMU,1729c	fw
igs1338	1,1	-1,0	-1,0	0,808	0,962	0,889	SMU,1728	SMU,1729c	rc
igs1339	1,1	-1,1	-1,1	0,543	0,505	0,632	SMU,1729c	SMU,1730c	fw
igs1339	-1,1	-1,1	1,1	0,431	0,398	0,596	SMU,1729c	SMU,1730c	rc
igs1340	-1,5	-1,6	-3,0	0,539	0,504	0,222	SMU,1730c	SMU,1731	fw
igs1340	1,1	1,0	-1,5	0,564	0,899	**0,018**	SMU,1730c	SMU,1731	rc
igs1342	**4,4**	-1,2	-2,1	**0,021**	0,742	0,181	SMU,1732c	SMU,1733c	fw
igs1342	-1,0	1,6	-1,7	0,849	0,072	0,094	SMU,1732c	SMU,1733c	rc
igs1343	-1,0	-1,0	-1,2	0,955	0,911	0,744	SMU,1733c	SMU,1734	fw
igs1343	1,2	1,3	1,3	0,367	0,167	**0,011**	SMU,1733c	SMU,1734	rc
igs1345	1,0	-1,4	-1,1	0,961	0,426	0,836	SMU,1736	SMU,1737	fw
igs1345	-1,1	-1,0	1,0	0,544	0,849	0,853	SMU,1736	SMU,1737	rc
igs1347	1,8	-1,0	-2,2	**0,010**	0,897	**0,003**	SMU,1739	SMU,1740	fw
igs1347	-1,1	-1,1	-1,1	0,391	0,602	0,314	SMU,1739	SMU,1740	rc
igs1350	1,7	-1,1	**2,2**	0,112	0,778	**0,024**	SMU,1742c	SMU,1743	fw
igs1350	1,4	-3,1	**2,5**	**0,001**	**0,000**	**0,000**	SMU,1742c	SMU,1743	rc
igs1351	-1,1	-1,5	-1,1	0,749	0,152	0,755	SMU,1743	SMU,1744	fw
igs1351	-2,0	1,2	1,6	**0,001**	0,066	**0,014**	SMU,1743	SMU,1744	rc
igs1352	-1,2	-1,1	1,0	0,610	0,759	0,958	SMU,1745c	SMU,1746c	fw
igs1352	1,1	-1,2	1,2	**0,001**	**0,002**	0,053	SMU,1745c	SMU,1746c	rc
igs1353	-1,1	-1,0	1,3	0,829	0,957	0,666	SMU,1746c	SMU,1747c	fw
igs1353	-1,4	1,0	1,1	**0,001**	0,843	0,310	SMU,1746c	SMU,1747c	rc
igs1354	-1,3	1,2	-1,5	0,511	0,755	0,274	SMU,1747c	SMU,1748	fw
igs1354	1,7	-1,6	1,2	0,069	**0,011**	0,578	SMU,1747c	SMU,1748	rc
igs1355	-1,1	-1,0	-1,2	0,762	0,925	0,771	SMU,1748	SMUt41	fw
igs1355	-1,3	-1,2	-1,3	**0,000**	**0,032**	**0,004**	SMU,1748	SMUt41	rc
igs1356	**2,3**	1,2	1,2	**0,000**	**0,000**	0,047	SMUt41	SMUt42	fw
igs1356	1,1	-1,0	1,2	**0,000**	0,514	**0,006**	SMUt41	SMUt42	rc
igs1362	**2,2**	1,2	1,3	**0,000**	**0,011**	**0,001**	SMUt47	SMUt48	fw
igs1362	1,0	-1,2	-1,1	0,266	**0,010**	**0,002**	SMUt47	SMUt48	rc
igs1363	**2,1**	-1,0	1,9	**0,000**	0,883	**0,000**	SMUt48	SMUt49	fw
igs1363	1,1	-1,1	-1,2	**0,046**	**0,035**	**0,030**	SMUt48	SMUt49	rc
igs1364	**2,1**	1,1	1,6	**0,000**	0,302	**0,000**	SMUr07	SMUr08	fw
igs1364	1,0	1,1	-1,1	0,694	0,244	0,156	SMUr07	SMUr08	rc
igs1365	1,5	-1,1	1,5	**0,000**	0,189	**0,000**	SMUr08	SMUt50	fw
igs1365	1,0	-1,0	1,5	0,933	0,954	**0,001**	SMUr08	SMUt50	rc
igs1366	**2,3**	1,1	1,5	**0,000**	0,076	**0,000**	SMUt50	SMUr09	fw
igs1366	1,2	1,3	1,1	**0,015**	0,099	0,386	SMUt50	SMUr09	rc
igs1367	1,1	1,0	1,1	0,522	0,977	0,723	SMUr09	SMU,1750c	fw
igs1367	1,1	1,0	1,1	0,471	0,785	0,331	SMUr09	SMU,1750c	rc
igs1368	1,2	1,1	1,1	0,609	0,764	0,709	SMU,1750c	SMU,1752c	fw
igs1368	1,1	1,0	-1,1	**0,012**	0,812	**0,036**	SMU,1750c	SMU,1752c	rc
igs1369	-1,4	-1,2	1,3	0,092	0,408	0,405	SMU,1752c	SMU,1753c	fw
igs1369	-1,1	-1,0	-1,0	0,408	0,948	0,975	SMU,1752c	SMU,1753c	rc
igs1371	1,4	1,2	1,5	**0,011**	0,118	**0,006**	SMU,1754c	SMU,1755c	fw
igs1371	-1,1	1,1	1,1	0,093	**0,042**	0,531	SMU,1754c	SMU,1755c	rc
igs1372	1,8	1,2	-1,2	**0,003**	0,232	0,053	SMU,1758c	SMU,1760c	fw
igs1372	1,1	-1,7	1,9	0,256	**0,002**	**0,014**	SMU,1758c	SMU,1760c	rc

igs1374	-1,3	-1,3	1,3	0,313	0,093	0,174	SMU,1763c	SMU,1764c	fw
igs1374	1,4	1,2	-1,0	0,056	0,168	0,822	SMU,1763c	SMU,1764c	rc
igs1375	1,4	-1,4	1,8	0,444	0,294	0,063	SMU,1764c	SMU,1765c	fw
igs1375	-1,1	1,6	1,5	0,214	**0,001**	**0,013**	SMU,1764c	SMU,1765c	rc
igs1376	1,2	-1,3	1,1	0,705	0,580	0,806	SMU,1765c	SMU,1766c	fw
igs1376	1,1	1,1	1,2	0,188	0,496	**0,025**	SMU,1765c	SMU,1766c	rc
igs1377	1,2	-1,4	1,0	0,093	0,066	0,885	SMU,1766c	SMU,1767c	fw
igs1377	1,3	-1,3	-1,1	**0,001**	**0,009**	0,165	SMU,1766c	SMU,1767c	rc
igs1378	-1,2	-1,5	1,1	0,430	**0,049**	0,632	SMU,1768c	SMU,1770	fw
igs1378	1,0	-1,0	1,1	0,753	0,793	0,266	SMU,1768c	SMU,1770	rc
igs1380	1,5	-1,0	-1,1	**0,020**	0,869	0,785	SMU,1773c	SMU,1774c	fw
igs1380	-1,1	1,0	1,1	0,304	0,293	0,114	SMU,1773c	SMU,1774c	rc
igs1381	-1,0	1,1	-1,8	0,952	0,799	0,118	SMU,1774c	SMUt51	fw
igs1381	1,2	-1,3	1,7	0,536	0,484	0,240	SMU,1774c	SMUt51	rc
igs1382	-1,1	-1,1	-1,1	0,674	0,788	0,564	SMUt51	SMU,1775c	fw
igs1382	1,2	-1,0	1,4	0,562	0,947	0,327	SMUt51	SMU,1775c	rc
igs1383	1,1	-1,0	-1,2	0,607	0,786	0,250	SMU,1775c	SMU,1776c	fw
igs1383	-1,1	-1,3	1,0	0,558	**0,000**	0,593	SMU,1775c	SMU,1776c	rc
igs1384	-1,1	-1,6	1,2	0,505	**0,001**	0,241	SMU,1776c	SMU,1777	fw
igs1384	-1,2	-1,3	1,2	0,164	0,073	0,138	SMU,1776c	SMU,1777	rc
igs1386	1,4	-1,2	1,4	**0,003**	0,415	0,171	SMU,1779c	SMU,1780	fw
igs1386	-1,1	1,2	-1,1	0,254	0,229	0,487	SMU,1779c	SMU,1780	rc
igs1387	-1,0	1,1	1,5	0,866	0,483	**0,001**	SMU,1780	SMU,1781	fw
igs1387	-1,2	-1,0	1,2	0,708	0,942	0,718	SMU,1780	SMU,1781	rc
igs1388	-1,1	1,2	1,3	0,874	0,246	**0,025**	SMU,1781	SMU,1782	fw
igs1388	-1,4	-1,3	1,4	0,222	0,181	0,167	SMU,1781	SMU,1782	rc
igs1389	1,3	1,0	-1,0	0,699	0,966	0,988	SMU,1782	SMUt52	fw
igs1389	-1,2	-1,3	-1,0	0,260	0,098	0,972	SMU,1782	SMUt52	rc
igs1393	**2,2**	1,2	1,3	**0,000**	**0,011**	**0,001**	SMUt55	SMUt56	fw
igs1393	1,0	-1,2	-1,1	0,260	**0,010**	**0,002**	SMUt55	SMUt56	rc
igs1394	**2,1**	-1,0	1,9	**0,000**	0,864	**0,000**	SMUt56	SMUt57	fw
igs1394	1,1	-1,1	-1,2	**0,047**	**0,035**	**0,030**	SMUt56	SMUt57	rc
igs1395	**2,1**	1,1	1,6	**0,000**	0,304	**0,000**	SMUr10	SMUr11	fw
igs1395	1,0	1,1	-1,1	0,696	0,245	0,155	SMUr10	SMUr11	rc
igs1396	1,5	-1,1	1,5	**0,000**	0,194	**0,000**	SMUr11	SMUt58	fw
igs1396	1,0	-1,0	1,5	0,932	0,953	**0,001**	SMUr11	SMUt58	rc
igs1397	**2,3**	1,1	1,5	**0,000**	0,081	**0,000**	SMUt58	SMUr12	fw
igs1397	1,2	1,3	1,1	**0,015**	0,099	0,385	SMUt58	SMUr12	rc
igs1398	1,2	1,1	1,1	0,669	0,931	0,868	SMUr12	SMU,1783	fw
igs1398	-1,0	1,0	1,2	0,970	0,674	**0,037**	SMUr12	SMU,1783	rc
igs1399	-1,1	-1,2	-1,0	0,431	0,122	0,732	SMU,1783	SMU,1784c	fw
igs1399	**2,9**	-1,5	-1,6	**0,003**	0,214	0,068	SMU,1783	SMU,1784c	rc
igs1402	-1,5	-1,8	1,8	0,286	0,074	0,174	SMU,1786	SMU,1787c	fw
igs1402	1,4	-1,2	1,8	0,122	0,340	**0,000**	SMU,1786	SMU,1787c	rc
igs1403	1,2	1,1	-1,2	0,278	0,740	0,260	SMU,1787c	SMU,1788c	fw
igs1403	-1,1	1,0	1,1	**0,029**	0,357	**0,027**	SMU,1787c	SMU,1788c	rc
igs1404	1,0	-1,1	-1,4	0,947	0,797	0,384	SMU,1788c	SMU,1789c	fw
igs1404	-1,0	-1,4	1,6	0,724	**0,039**	**0,003**	SMU,1788c	SMU,1789c	rc
igs1405	1,2	-1,2	1,2	0,632	0,634	0,600	SMU,1789c	SMU,1790c	fw
igs1405	1,3	1,1	-1,6	**0,000**	0,653	0,052	SMU,1789c	SMU,1790c	rc
igs1406	-1,4	-1,0	1,1	0,136	0,870	0,662	SMU,1790c	SMU,1791c	fw
igs1406	-1,1	-1,2	-1,2	0,562	0,438	0,202	SMU,1790c	SMU,1791c	rc
igs1407	-1,1	1,3	1,6	0,616	**0,036**	**0,000**	SMU,1791c	SMU,1792c	fw
igs1407	1,0	1,2	1,0	0,740	**0,028**	0,925	SMU,1791c	SMU,1792c	rc
igs1408	-1,1	-1,1	-1,0	0,099	0,101	0,796	SMU,1792c	SMU,1794c	fw
igs1408	-1,2	1,3	1,1	**0,023**	0,080	0,305	SMU,1792c	SMU,1794c	rc
igs1409	1,0	-1,2	-1,2	0,871	0,149	0,512	SMU,1794c	SMU,1795c	fw
igs1409	-1,1	-1,3	1,1	**0,000**	**0,000**	0,055	SMU,1794c	SMU,1795c	rc
igs1410	1,1	1,1	-1,2	0,817	0,738	0,334	SMU,1795c	SMU,1797c	fw
igs1410	-1,1	-1,0	-1,1	0,092	0,824	0,505	SMU,1795c	SMU,1797c	rc
igs1411	1,6	1,1	-1,8	**0,000**	0,113	**0,000**	SMU,1799	SMU,1800c	fw
igs1411	1,1	-1,1	1,0	0,446	0,657	0,869	SMU,1799	SMU,1800c	rc
igs1412	1,1	-1,1	1,2	0,181	0,214	0,096	SMU,1800c	SMU,1801c	fw
igs1412	1,3	-1,6	1,4	**0,001**	**0,000**	**0,003**	SMU,1800c	SMU,1801c	rc
igs1413	-1,3	-1,1	-1,1	0,318	0,725	0,595	SMU,1802c	SMU,1803c	fw
igs1413	-1,4	1,1	-2,1	**0,001**	0,619	**0,000**	SMU,1802c	SMU,1803c	rc

igs1415	-1,5	-1,2	-1,0	**0,000**	**0,013**	0,626	SMU,1804c	SMU,1805	fw
igs1415	-1,2	-1,1	-1,9	0,215	0,658	**0,004**	SMU,1804c	SMU,1805	rc
igs1416	1,7	-1,1	1,0	**0,010**	0,371	0,398	SMU,1806	SMU,1807c	fw
igs1416	-1,0	-1,4	1,3	1,000	**0,000**	**0,008**	SMU,1806	SMU,1807c	rc
igs1417	-1,1	-1,0	-1,5	0,672	0,844	**0,013**	SMU,1807c	SMU,1808c	fw
igs1417	-1,4	1,1	1,3	**0,000**	0,151	**0,000**	SMU,1807c	SMU,1808c	rc
igs1418	-1,1	-1,0	-1,3	0,640	0,826	0,071	SMU,1808c	SMU,1809	fw
igs1418	-1,0	1,1	-1,2	0,379	**0,036**	**0,000**	SMU,1808c	SMU,1809	rc
igs1420	-1,3	1,1	-1,4	0,557	0,868	0,452	SMU,1811	SMU,1812	fw
igs1420	1,0	-1,1	-1,7	0,595	0,581	**0,000**	SMU,1811	SMU,1812	rc
igs1421	-1,1	-1,3	-1,1	0,758	0,338	0,731	SMU,1812	SMU,1813	fw
igs1421	-1,1	1,1	-1,0	**0,038**	0,386	0,624	SMU,1812	SMU,1813	rc
igs1422	-1,1	-1,2	1,1	0,108	**0,011**	0,060	SMU,1813	SMU,1814	fw
igs1422	1,2	1,2	-1,1	**0,001**	**0,000**	0,208	SMU,1813	SMU,1814	rc
igs1424	-1,1	-1,1	-1,1	0,384	0,721	0,583	SMU,1815	SMU,1816c	fw
igs1424	1,0	1,1	-1,1	0,803	0,585	0,193	SMU,1815	SMU,1816c	rc
igs1425	1,2	-1,1	-1,2	0,087	0,701	0,276	SMU,1816c	SMU,1817c	fw
igs1425	-1,0	1,1	1,1	0,866	**0,000**	**0,011**	SMU,1816c	SMU,1817c	rc
igs1426	1,1	1,0	1,1	0,564	0,530	0,098	SMU,1817c	SMU,1818c	fw
igs1426	1,0	1,2	-1,1	0,690	**0,001**	**0,000**	SMU,1817c	SMU,1818c	rc
igs1427	1,2	-1,1	-1,0	0,571	0,722	0,903	SMU,1818c	SMU,1819	fw
igs1427	1,2	-1,5	-1,1	**0,000**	**0,000**	0,537	SMU,1818c	SMU,1819	rc
igs1428	-1,1	-1,2	-1,2	0,611	0,434	0,224	SMU,1821c	SMU,1822	fw
igs1428	1,7	-1,6	1,1	**0,000**	**0,007**	0,672	SMU,1821c	SMU,1822	rc
igs1429	1,0	-1,1	-1,3	0,878	0,583	0,200	SMU,1822	SMU,1823	fw
igs1429	1,4	1,0	1,0	**0,000**	0,725	0,645	SMU,1822	SMU,1823	rc
igs1431	1,3	-1,6	-1,2	0,494	0,284	0,665	SMU,1824c	SMU,1826	fw
igs1431	1,1	-1,1	-1,1	0,051	0,567	0,273	SMU,1824c	SMU,1826	rc
igs1432	-1,2	-1,2	1,2	0,499	0,397	0,415	SMU,1826	SMU,1827	fw
igs1432	1,1	-1,7	1,6	0,487	**0,000**	**0,003**	SMU,1826	SMU,1827	rc
igs1433	1,0	-1,0	-1,1	0,999	0,784	0,355	SMU,1827	SMU,1828	fw
igs1433	1,0	1,1	-1,5	0,964	0,912	0,609	SMU,1827	SMU,1828	rc
igs1434	1,1	1,0	-1,0	0,745	0,941	0,940	SMU,1828	SMU,1830c	fw
igs1434	1,0	1,1	-1,1	0,971	0,699	0,865	SMU,1828	SMU,1830c	rc
igs1435	1,6	**2,5**	1,1	0,067	**0,013**	0,724	SMU,1830c	SMU,1831	fw
igs1435	1,9	1,7	-1,3	**0,033**	0,183	0,443	SMU,1830c	SMU,1831	rc
igs1436	-1,3	-1,0	-1,2	0,215	0,925	0,358	SMU,1831	SMU,1832	fw
igs1436	-1,2	-1,4	-2,1	0,567	0,359	0,117	SMU,1831	SMU,1832	rc
igs1437	1,1	-1,5	1,3	0,537	0,073	**0,000**	SMU,1832	SMU,1833	fw
igs1437	-1,2	-1,0	-1,1	0,396	0,845	0,517	SMU,1832	SMU,1833	rc
igs1438	-1,3	1,2	1,1	0,132	0,549	0,556	SMU,1833	SMU,1834	fw
igs1438	-1,1	-1,3	1,4	0,314	**0,002**	**0,000**	SMU,1833	SMU,1834	rc
igs1439	1,1	-1,1	1,0	0,642	0,620	0,990	SMU,1835	SMU,1836	fw
igs1439	-1,1	-1,0	-1,0	0,139	0,897	0,628	SMU,1835	SMU,1836	rc
igs1442	-1,1	-1,1	-1,1	0,797	0,904	0,823	SMU,1838	SMU,1839	fw
igs1442	1,1	-1,2	-1,0	0,440	0,304	0,931	SMU,1838	SMU,1839	rc
igs1443	1,1	-1,9	-1,1	0,814	0,136	0,754	SMU,1839	SMU,1840	fw
igs1443	-1,2	1,2	-1,4	**0,002**	**0,026**	**0,000**	SMU,1839	SMU,1840	rc
igs1444	-1,7	-1,7	-1,1	0,094	0,105	0,634	SMU,1840	SMU,1841	fw
igs1444	-1,0	-1,3	1,0	0,661	0,050	0,912	SMU,1840	SMU,1841	rc
igs1445	1,0	-1,3	1,0	0,841	**0,044**	0,865	SMU,1841	SMU,1843	fw
igs1445	-1,0	-1,0	1,2	0,817	0,831	0,201	SMU,1841	SMU,1843	rc
igs1447	-1,2	-1,3	-1,6	0,703	0,543	0,190	SMU,1844	SMU,1845	fw
igs1447	-1,0	1,1	-1,4	1,000	0,553	0,051	SMU,1844	SMU,1845	rc
igs1448	1,1	-1,1	-1,7	0,435	0,626	**0,017**	SMU,1846c	SMU,1847	fw
igs1448	-1,1	1,1	1,0	0,384	0,410	0,749	SMU,1846c	SMU,1847	rc
igs1449	-1,2	-1,2	1,2	0,653	0,686	0,660	SMU,1847	SMU,1848	fw
igs1449	1,4	1,1	-1,6	0,085	0,646	0,082	SMU,1847	SMU,1848	rc
igs1450	-1,2	-1,1	-1,1	0,682	0,886	0,870	SMU,1848	SMU,1849	fw
igs1450	-1,8	1,3	1,1	0,157	0,585	0,718	SMU,1848	SMU,1849	rc
igs1451	-1,0	-1,3	-1,5	0,929	0,393	0,193	SMU,1849	SMU,1850	fw
igs1451	1,0	1,3	1,1	0,790	0,148	0,529	SMU,1849	SMU,1850	rc
igs1452	-1,1	1,2	-1,2	0,561	0,321	0,153	SMU,1851	SMU,1852	fw
igs1452	-1,2	-1,1	1,1	0,141	0,575	0,464	SMU,1851	SMU,1852	rc
igs1453	-1,0	**2,2**	-1,3	0,667	**0,000**	**0,012**	SMU,1852	SMU,1853	fw
igs1453	-1,3	-1,1	1,5	0,107	0,684	0,065	SMU,1852	SMU,1853	rc

igs1454	-1,0	-1,3	-1,0	0,885	0,259	0,955	SMU,1853	SMU,1854	fw
igs1454	-1,3	-1,6	1,4	0,190	**0,010**	0,142	SMU,1853	SMU,1854	rc
igs1456	1,1	-1,0	-1,3	0,731	0,964	0,519	SMU,1856c	SMU,1858	fw
igs1456	-1,2	-1,1	1,1	0,296	0,608	0,345	SMU,1856c	SMU,1858	rc
igs1457	1,8	1,0	1,5	**0,005**	0,921	0,087	SMU,1858	SMU,1859	fw
igs1457	-1,2	1,2	1,2	**0,000**	**0,015**	0,055	SMU,1858	SMU,1859	rc
igs1459	1,5	-1,1	1,1	0,693	0,939	0,923	SMU,1860	SMU,1861c	fw
igs1459	1,1	-1,1	-1,1	0,784	0,728	0,735	SMU,1860	SMU,1861c	rc
igs1460	-1,0	1,1	1,5	0,761	0,720	**0,013**	SMU,1861c	SMU,1862	fw
igs1460	-1,0	1,7	1,2	0,626	**0,001**	0,159	SMU,1861c	SMU,1862	rc
igs1461	-1,0	1,0	-1,1	0,960	0,944	0,784	SMU,1862	SMU,1865	fw
igs1461	1,0	1,0	1,2	0,772	0,720	0,080	SMU,1862	SMU,1865	rc
igs1462	1,1	-1,3	-1,1	0,654	**0,032**	0,615	SMU,1865	SMU,1867c	fw
igs1462	1,1	-1,0	1,6	0,791	0,846	0,056	SMU,1865	SMU,1867c	rc
igs1463	-1,9	-1,4	1,0	**0,024**	0,400	0,950	SMU,1867c	SMU,1869	fw
igs1463	-1,2	1,1	-1,1	**0,007**	0,582	0,342	SMU,1867c	SMU,1869	rc
igs1464	-1,0	-1,1	-1,1	0,990	0,884	0,935	SMU,1869	SMU,1870	fw
igs1464	-1,1	-1,0	-1,3	0,265	0,507	**0,000**	SMU,1869	SMU,1870	rc
igs1465	1,2	-1,8	1,5	0,777	0,352	0,424	SMU,1870	SMU,1871c	fw
igs1465	1,0	-1,3	1,0	0,893	**0,008**	0,513	SMU,1870	SMU,1871c	rc
igs1466	-1,1	-1,1	1,5	0,604	0,758	0,110	SMU,1872c	SMU,1873	fw
igs1466	-1,2	-1,2	1,5	**0,028**	0,129	**0,000**	SMU,1872c	SMU,1873	rc
igs1468	1,2	1,3	-1,2	0,078	**0,000**	**0,005**	SMU,1874	SMU,1875	fw
igs1468	-1,5	-1,3	-1,0	**0,001**	**0,006**	0,980	SMU,1874	SMU,1875	rc
igs1469	1,3	1,0	-1,0	0,080	0,593	0,615	SMU,1875	SMU,1876	fw
igs1469	1,7	-1,7	1,0	0,177	0,222	0,973	SMU,1875	SMU,1876	rc
igs1470	-1,0	1,1	-1,1	0,375	0,095	0,343	SMU,1876	SMU,1877	fw
igs1470	-1,1	-1,4	-1,0	0,555	0,071	0,996	SMU,1876	SMU,1877	rc
igs1471	-1,4	-1,3	-1,3	**0,000**	**0,002**	**0,000**	SMU,1877	SMU,1878	fw
igs1471	-1,8	-1,4	1,2	**0,000**	**0,003**	0,094	SMU,1877	SMU,1878	rc
igs1473	-1,1	-1,1	-1,3	0,732	0,367	0,114	SMU,1879	SMU,1881c	fw
igs1473	1,2	-1,3	1,2	0,153	0,238	0,333	SMU,1879	SMU,1881c	rc
igs1474	-1,1	-1,6	-1,6	0,679	**0,031**	**0,045**	SMU,1881c	SMU,1882c	fw
igs1474	-1,1	1,2	-1,2	0,311	0,110	**0,022**	SMU,1881c	SMU,1882c	rc
igs1475	1,4	-1,3	-1,1	**0,011**	0,066	0,656	SMU,1882c	SMU,1883	fw
igs1475	-1,0	1,3	1,2	0,948	0,109	0,372	SMU,1882c	SMU,1883	rc
igs1476	1,3	-1,5	-1,2	**0,011**	**0,015**	0,162	SMU,1883	SMU,1884c	fw
igs1476	-1,4	-1,3	1,1	0,403	0,368	0,663	SMU,1883	SMU,1884c	rc
igs1477	1,1	1,1	1,0	**0,029**	0,102	0,768	SMU,1884c	SMU,1886	fw
igs1477	1,0	-1,2	-1,1	0,876	0,479	0,785	SMU,1884c	SMU,1886	rc
igs1478	-1,1	-1,1	-1,0	0,128	0,507	0,716	SMU,1886	SMU,1888	fw
igs1478	-1,0	-1,1	-1,4	0,859	0,798	0,133	SMU,1886	SMU,1888	rc
igs1479	1,0	1,1	1,1	0,833	0,486	0,363	SMU,1888	SMU,1889c	fw
igs1479	1,3	-1,2	-1,0	**0,004**	**0,000**	0,758	SMU,1888	SMU,1889c	rc
igs1480	-1,1	1,4	1,0	0,401	**0,018**	0,876	SMU,1889c	SMU,1891c	fw
igs1480	1,0	1,7	-1,0	0,867	**0,000**	0,862	SMU,1889c	SMU,1891c	rc
igs1481	1,3	-1,4	-1,0	0,076	0,180	0,942	SMU,1892c	SMU,1893c	fw
igs1481	-1,1	-1,2	1,1	0,834	0,668	0,869	SMU,1892c	SMU,1893c	rc
igs1482	1,1	-1,3	-1,9	0,861	0,592	0,191	SMU,1894c	SMU,1895c	fw
igs1482	-1,4	-1,2	1,7	**0,033**	**0,000**	**0,002**	SMU,1894c	SMU,1895c	rc
igs1483	1,8	-2,3	-3,2	0,262	0,100	**0,028**	SMU,1895c	SMU,1896c	fw
igs1483	1,5	1,5	**2,3**	**0,004**	0,152	0,060	SMU,1895c	SMU,1896c	rc
igs1484	1,0	**2,0**	**2,1**	0,862	**0,000**	**0,000**	SMU,1896c	SMU,1897	fw
igs1484	-1,1	-1,0	1,1	0,569	0,968	0,280	SMU,1896c	SMU,1897	rc
igs1485	**2,5**	**5,2**	**3,8**	**0,000**	**0,000**	**0,000**	SMU,1897	SMU,1898	fw
igs1485	-1,1	1,2	-1,2	**0,024**	0,075	**0,017**	SMU,1897	SMU,1898	rc
igs1487	**2,4**	**2,3**	**2,8**	**0,019**	**0,020**	**0,006**	SMU,1900	SMU,1902c	fw
igs1487	-1,0	1,2	1,3	0,592	0,085	**0,000**	SMU,1900	SMU,1902c	rc
igs1488	**2,8**	**4,2**	**2,8**	0,148	0,052	0,153	SMU,1902c	SMU,1903c	fw
igs1488	1,6	1,0	2,0	**0,001**	0,831	**0,001**	SMU,1902c	SMU,1903c	rc
igs1489	**14,7**	**20,1**	**18,3**	**0,002**	**0,002**	**0,004**	SMU,1903c	SMU,1904c	fw
igs1489	-1,5	1,6	-1,1	**0,035**	**0,004**	0,545	SMU,1903c	SMU,1904c	rc
igs1490	**10,6**	**17,6**	**25,1**	**0,000**	**0,000**	**0,000**	SMU,1904c	SMU,1905c	fw
igs1490	1,0	-1,3	-1,1	0,552	**0,000**	0,544	SMU,1904c	SMU,1905c	rc
igs1491	**11,0**	**39,4**	**48,4**	**0,000**	**0,000**	**0,000**	SMU,1905c	SMU,1906c	fw
igs1491	-1,0	-1,2	-1,1	0,955	0,208	0,752	SMU,1905c	SMU,1906c	rc

igs1492	**17,8**	**49,0**	**47,8**	**0,000**	**0,000**	**0,000**	SMU,1906c	SMU,1907	fw
igs1492	-1,1	-1,2	1,5	**0,038**	**0,009**	**0,001**	SMU,1906c	SMU,1907	rc
igs1493	**2,6**	**13,4**	**11,3**	**0,003**	**0,000**	**0,000**	SMU,1907	SMU,1908c	fw
igs1493	1,3	1,2	1,1	**0,005**	0,281	0,755	SMU,1907	SMU,1908c	rc
igs1494	**12,1**	**21,4**	**42,1**	**0,000**	**0,000**	**0,000**	SMU,1908c	SMU,1909c	fw
igs1494	1,2	-1,1	1,1	**0,008**	0,391	0,436	SMU,1908c	SMU,1909c	rc
igs1495	**15,2**	**43,1**	**44,4**	**0,000**	**0,000**	**0,000**	SMU,1909c	SMU,1910c	fw
igs1495	-1,1	-1,3	1,1	0,770	**0,028**	**0,031**	SMU,1909c	SMU,1910c	rc
igs1496	**14,4**	**33,6**	**72,6**	**0,000**	**0,000**	**0,000**	SMU,1910c	SMU,1912c	fw
igs1496	-1,1	1,0	1,2	0,238	0,587	**0,029**	SMU,1910c	SMU,1912c	rc
igs1497	**11,9**	**22,9**	**91,2**	**0,000**	**0,000**	**0,000**	SMU,1912c	SMU,1913c	fw
igs1497	-1,2	-1,2	1,3	0,150	0,344	0,122	SMU,1912c	SMU,1913c	rc
igs1498	**12,3**	**12,5**	**20,4**	**0,000**	**0,000**	**0,000**	SMU,1913c	SMU,1914c	fw
igs1498	-1,6	-1,5	-1,2	**0,001**	**0,042**	0,254	SMU,1913c	SMU,1914c	rc
igs1499	**3,4**	**5,1**	**6,4**	**0,015**	**0,001**	**0,001**	SMU,1914c	SMU,1915	fw
igs1499	-1,2	-1,2	-1,4	**0,009**	0,177	0,052	SMU,1914c	SMU,1915	rc
igs1500	1,5	**2,8**	1,6	0,198	**0,023**	0,127	SMU,1915	SMU,1916	fw
igs1500	-1,0	-1,0	1,2	0,938	0,995	0,685	SMU,1915	SMU,1916	rc
igs1501	-1,0	1,5	1,3	0,898	**0,017**	**0,013**	SMU,1917	SMU,1918	fw
igs1501	1,0	-1,0	-1,1	0,661	0,635	**0,018**	SMU,1917	SMU,1918	rc
igs1502	1,4	1,1	-1,8	**0,007**	0,331	**0,000**	SMU,1918	SMU,1919	fw
igs1502	1,3	-1,2	1,1	0,280	0,165	0,523	SMU,1918	SMU,1919	rc
igs1503	-1,1	1,1	-2,1	0,446	0,365	**0,000**	SMU,1919	SMU,1920	fw
igs1503	-1,2	-1,1	-1,3	**0,024**	0,227	**0,004**	SMU,1919	SMU,1920	rc
igs1505	-1,3	-1,2	1,1	0,144	0,370	0,695	SMU,1923c	SMU,1924	fw
igs1505	1,1	-1,0	1,2	0,399	0,834	0,224	SMU,1923c	SMU,1924	rc
igs1506	-1,0	-1,1	1,1	0,848	0,645	0,716	SMU,1924	SMU,1925c	fw
igs1506	-1,1	-1,1	-1,2	0,149	0,419	**0,005**	SMU,1924	SMU,1925c	rc
igs1507	-1,3	1,5	1,4	0,302	**0,025**	**0,007**	SMU,1926	SMU,1927	fw
igs1507	1,7	**2,1**	-2,0	**0,000**	**0,000**	**0,000**	SMU,1926	SMU,1927	rc
igs1509	-1,2	-1,2	-1,2	0,683	0,645	0,605	SMU,1928	SMU,1929	fw
igs1509	1,0	-1,0	1,0	0,186	0,841	0,945	SMU,1928	SMU,1929	rc
igs1511	-1,3	-1,8	1,3	0,269	**0,045**	0,138	SMU,1930	SMU,1931	fw
igs1511	-1,4	-1,5	-3,9	0,153	0,195	**0,000**	SMU,1930	SMU,1931	rc
igs1512	-1,0	-1,1	1,0	0,867	0,791	0,923	SMU,1931	SMU,1933c	fw
igs1512	-1,0	-1,2	-1,5	0,798	0,365	**0,025**	SMU,1931	SMU,1933c	rc
igs1513	-1,0	1,1	-1,3	0,942	0,798	0,515	SMU,1934c	SMU,1935c	fw
igs1513	-1,7	1,5	1,3	**0,002**	0,097	0,155	SMU,1934c	SMU,1935c	rc
igs1515	1,1	-1,7	-1,9	0,684	**0,019**	**0,001**	SMU,1936c	SMU,1937	fw
igs1515	1,4	-1,4	-2,5	0,102	**0,000**	**0,000**	SMU,1936c	SMU,1937	rc
igs1516	1,0	1,1	-1,5	0,774	0,364	**0,018**	SMU,1937	SMU,1938c	fw
igs1516	1,3	-1,1	1,3	**0,000**	**0,019**	**0,000**	SMU,1937	SMU,1938c	rc
igs1518	-1,0	-1,5	-1,1	0,908	0,179	0,895	SMU,1940c	SMU,1941	fw
igs1518	-1,3	-1,2	-1,3	**0,000**	0,068	0,108	SMU,1940c	SMU,1941	rc
igs1519	1,1	-1,2	-1,1	0,707	0,506	0,769	SMU,1941	SMU,1942c	fw
igs1519	-1,1	1,0	1,5	0,242	0,806	**0,002**	SMU,1941	SMU,1942c	rc
igs1520	-1,2	-1,5	1,1	0,186	**0,022**	0,518	SMU,1942c	SMU,1943	fw
igs1520	-1,0	1,0	1,4	0,939	0,669	**0,001**	SMU,1942c	SMU,1943	rc
igs1521	-1,1	-1,2	1,1	0,599	0,259	0,251	SMU,1943	SMU,1945	fw
igs1521	-1,0	-1,1	1,1	0,961	0,436	0,454	SMU,1943	SMU,1945	rc
igs1522	1,1	-1,2	1,0	0,749	0,552	0,919	SMU,1946	SMU,1947	fw
igs1522	-1,1	1,0	1,1	0,409	0,737	0,207	SMU,1946	SMU,1947	rc
igs1523	1,1	-1,0	-2,1	0,402	0,977	**0,007**	SMU,1947	SMU,1948	fw
igs1523	-1,0	1,0	-1,1	0,751	0,932	0,095	SMU,1947	SMU,1948	rc
igs1524	1,0	-1,3	-1,4	0,848	0,056	0,075	SMU,1948	SMU,1949	fw
igs1524	-1,1	-1,4	1,0	0,140	**0,036**	0,437	SMU,1948	SMU,1949	rc
igs1525	1,7	-1,2	1,3	0,075	0,580	0,155	SMU,1949	SMU,1950	fw
igs1525	1,0	1,9	**2,3**	0,687	**0,000**	**0,000**	SMU,1949	SMU,1950	rc
igs1527	1,0	1,1	1,2	0,870	0,762	0,393	SMU,1951c	SMU,1954	fw
igs1527	-1,0	1,2	1,1	0,518	**0,035**	0,348	SMU,1951c	SMU,1954	rc
igs1528	1,1	-1,1	-1,2	0,696	0,668	0,580	SMU,1954	SMU,1955	fw
igs1528	1,4	-1,2	1,6	**0,030**	**0,006**	**0,000**	SMU,1954	SMU,1955	rc
igs1529	1,2	1,1	-1,2	0,808	0,904	0,844	SMU,1955	SMU,1956c	fw
igs1529	1,2	1,6	1,1	**0,014**	**0,000**	0,355	SMU,1955	SMU,1956c	rc
igs1532	-1,3	-1,0	1,2	0,191	0,811	0,388	SMU,1958c	SMU,1960c	fw
igs1532	-1,2	-1,1	1,0	**0,002**	0,146	0,579	SMU,1958c	SMU,1960c	rc

igs1534	1,1	1,3	-1,1	0,611	0,354	0,774	SMU,1961c	SMU,1963c	fw
igs1534	-1,1	-1,1	1,2	0,564	0,186	0,225	SMU,1961c	SMU,1963c	rc
igs1535	-1,3	**11,2**	-1,4	0,221	**0,000**	**0,034**	SMU,1966c	SMU,1967	fw
igs1535	-1,1	-1,2	1,2	0,143	**0,041**	**0,031**	SMU,1966c	SMU,1967	rc
igs1536	1,3	-1,1	1,3	0,216	0,900	0,175	SMU,1967	SMU,1968c	fw
igs1536	-1,7	1,0	-1,2	**0,000**	0,737	0,180	SMU,1967	SMU,1968c	rc
igs1537	1,0	1,6	-1,0	0,973	0,355	0,969	SMU,1969c	SMU,1970c	fw
igs1537	1,1	-1,3	-1,2	**0,014**	**0,042**	**0,016**	SMU,1969c	SMU,1970c	rc
igs1538	1,0	1,1	1,2	0,901	0,730	0,269	SMU,1972c	SMU,1973	fw
igs1538	1,1	1,1	-1,3	0,342	0,762	0,230	SMU,1972c	SMU,1973	rc
igs1539	-1,2	-1,0	**2,0**	0,653	0,897	**0,000**	SMU,1973	SMU,1974	fw
igs1539	-1,7	-1,3	-1,6	**0,032**	0,407	0,060	SMU,1973	SMU,1974	rc
igs1540	-1,1	1,3	-1,7	0,664	0,265	**0,002**	SMU,1974	SMU,1975c	fw
igs1540	-1,6	-1,5	1,5	0,054	0,089	0,052	SMU,1974	SMU,1975c	rc
igs1541	**3,1**	-1,2	-1,4	**0,002**	0,447	0,140	SMU,1975c	SMU,1976c	fw
igs1541	-1,3	1,1	-1,4	**0,026**	0,198	**0,001**	SMU,1975c	SMU,1976c	rc
igs1542	-1,2	**2,7**	1,1	0,422	**0,000**	0,785	SMU,1977c	SMU,1978	fw
igs1542	1,1	1,1	1,0	0,255	0,342	0,880	SMU,1977c	SMU,1978	rc
igs1543	-1,7	**7,4**	-1,1	**0,001**	**0,000**	0,573	SMU,1978	SMU,1979c	fw
igs1543	-1,4	-1,7	1,0	**0,000**	**0,002**	0,811	SMU,1978	SMU,1979c	rc
igs1544	1,2	**64,8**	-2,1	0,721	**0,000**	0,100	SMU,1979c	SMU,1980c	fw
igs1544	1,6	-1,1	1,0	**0,005**	0,183	0,138	SMU,1979c	SMU,1980c	rc
igs1545	-1,3	**18,5**	-1,7	0,319	**0,000**	**0,000**	SMU,1985	SMU,1987	fw
igs1545	1,0	-1,4	-1,7	0,778	**0,000**	**0,002**	SMU,1985	SMU,1987	rc
igs1546	1,2	1,0	1,3	0,624	0,999	0,128	SMU,1987	SMU,1988c	fw
igs1546	1,2	1,1	-1,0	**0,029**	0,497	0,882	SMU,1987	SMU,1988c	rc
igs1547	1,5	1,0	1,7	0,589	0,968	0,418	SMU,1988c	SMU,1989	fw
igs1547	-1,2	-1,1	1,8	0,089	0,192	**0,000**	SMU,1988c	SMU,1989	rc
igs1548	-1,4	-1,4	-1,1	**0,039**	0,068	0,632	SMU,1989	SMU,1990	fw
igs1548	1,4	-1,4	1,4	**0,000**	**0,016**	**0,000**	SMU,1989	SMU,1990	rc
igs1549	-1,1	1,2	1,2	0,888	0,698	0,663	SMU,1990	SMU,1991	fw
igs1549	1,0	-1,5	1,4	0,484	**0,015**	**0,006**	SMU,1990	SMU,1991	rc
igs1550	-1,1	-1,9	-1,1	0,652	**0,000**	0,270	SMU,1991	SMU,1992	fw
igs1550	1,5	1,4	1,2	**0,041**	0,147	0,571	SMU,1991	SMU,1992	rc
igs1551	-1,4	-1,2	-1,5	0,553	0,668	0,324	SMU,1992	SMU,1993	fw
igs1551	-1,1	-1,1	1,2	0,250	0,230	0,247	SMU,1992	SMU,1993	rc
igs1553	-1,0	1,6	-1,2	0,808	**0,000**	0,105	SMU,1995c	SMU,1996	fw
igs1553	-1,1	-1,1	1,1	0,191	0,203	0,171	SMU,1995c	SMU,1996	rc
igs1554	-1,5	**3,2**	1,3	0,359	**0,028**	0,213	SMU,1996	SMU,1997	fw
igs1554	1,3	1,3	**2,8**	**0,001**	0,251	**0,000**	SMU,1996	SMU,1997	rc
igs1555	1,1	1,1	-1,2	0,427	0,402	0,231	SMU,1997	SMUt59	fw
igs1555	-1,1	1,1	1,0	0,094	0,087	0,289	SMU,1997	SMUt59	rc
igs1557	**2,1**	1,1	1,6	**0,000**	0,313	**0,000**	SMUr13	SMUr14	fw
igs1557	1,0	1,1	-1,1	0,726	0,235	0,167	SMUr13	SMUr14	rc
igs1558	1,4	-1,1	1,5	**0,000**	0,151	**0,000**	SMUr14	SMUt60	fw
igs1558	1,0	-1,0	1,5	0,842	0,999	**0,001**	SMUr14	SMUt60	rc
igs1559	**2,3**	1,1	1,6	**0,000**	0,126	**0,000**	SMUt60	SMUr15	fw
igs1559	1,2	1,3	1,1	**0,017**	0,095	0,368	SMUt60	SMUr15	rc
igs1560	1,8	1,0	1,5	**0,010**	0,886	0,214	SMUr15	SMU,1999c	fw
igs1560	1,1	-1,2	1,6	0,696	0,312	**0,011**	SMUr15	SMU,1999c	rc
igs1561	1,1	-1,1	-1,3	0,871	0,879	0,773	SMU,1999c	SMU,2000	fw
igs1561	-1,2	-1,3	1,4	0,109	0,122	**0,018**	SMU,1999c	SMU,2000	rc
igs1563	1,2	-1,1	-1,5	0,079	0,143	**0,003**	SMU,2001	SMU,2002	fw
igs1563	-1,2	1,3	1,2	**0,027**	**0,003**	**0,002**	SMU,2001	SMU,2002	rc
igs1567	1,1	-1,4	1,4	0,860	0,694	0,616	SMU,2004	SMU,2005	fw
igs1567	-1,0	-1,3	1,0	0,513	**0,006**	0,699	SMU,2004	SMU,2005	rc
igs1568	1,1	-1,3	-1,3	0,901	0,703	0,729	SMU,2005	SMU,2006	fw
igs1568	1,3	-1,0	1,0	**0,034**	0,955	0,804	SMU,2005	SMU,2006	rc
igs1569	1,2	-1,2	-1,3	0,053	0,107	**0,013**	SMU,2007	SMU,2008	fw
igs1569	1,0	1,0	-1,2	0,673	0,585	**0,044**	SMU,2007	SMU,2008	rc
igs1572	1,5	-1,4	1,0	0,248	0,206	0,922	SMU,2010	SMU,2011	fw
igs1572	1,3	-1,4	1,6	**0,000**	**0,001**	**0,000**	SMU,2010	SMU,2011	rc
igs1573	-1,0	-1,5	1,3	0,879	**0,037**	0,249	SMU,2011	SMU,2012	fw
igs1573	1,4	1,2	1,2	**0,000**	**0,032**	**0,001**	SMU,2011	SMU,2012	rc
igs1574	1,2	-1,2	1,1	0,141	0,287	0,688	SMU,2012	SMU,2014	fw
igs1574	-1,1	-1,2	1,0	0,242	0,077	0,818	SMU,2012	SMU,2014	rc

igs1577	1,5	-1,1	1,2	0,109	0,790	0,403	SMU,2016	SMU,2017	fw
igs1577	1,1	1,1	1,0	0,092	0,466	0,407	SMU,2016	SMU,2017	rc
igs1578	-1,1	-1,4	-1,1	0,137	**0,002**	0,420	SMU,2017	SMU,2018	fw
igs1578	1,1	1,5	-1,1	0,077	**0,001**	0,182	SMU,2017	SMU,2018	rc
igs1585	-1,2	-1,2	1,4	0,693	0,726	0,612	SMU,2025	SMU,2026c	fw
igs1585	-1,1	1,7	1,0	0,356	**0,000**	0,638	SMU,2025	SMU,2026c	rc
igs1586	1,3	1,2	1,2	0,645	0,659	0,698	SMU,2026c	SMU,2027	fw
igs1586	-1,0	1,7	1,1	0,767	**0,000**	0,052	SMU,2026c	SMU,2027	rc
igs1587	-1,1	1,0	1,0	0,597	0,778	0,910	SMU,2027	SMU,2028	fw
igs1587	-1,0	1,0	1,1	0,893	0,914	0,730	SMU,2027	SMU,2028	rc
igs1588	1,2	1,0	-1,0	0,478	0,896	0,907	SMU,2028	SMU,2029	fw
igs1588	1,2	-1,2	1,4	0,055	**0,024**	**0,016**	SMU,2028	SMU,2029	rc
igs1589	-1,2	1,1	-1,2	0,637	0,825	0,597	SMU,2030	SMU,2031	fw
igs1589	-1,3	-1,1	1,1	**0,000**	0,067	0,204	SMU,2030	SMU,2031	rc
igs1590	-1,0	-1,2	-1,3	0,682	**0,048**	**0,026**	SMU,2031	SMU,2032	fw
igs1590	1,2	1,1	1,0	**0,013**	0,203	0,710	SMU,2031	SMU,2032	rc
igs1591	-1,2	-1,4	-1,1	0,678	0,550	0,932	SMU,2032	SMU,2033c	fw
igs1591	1,0	-1,0	1,2	0,564	0,917	**0,022**	SMU,2032	SMU,2033c	rc
igs1592	1,1	-1,2	-1,2	0,862	0,551	0,639	SMU,2033c	SMUt61	fw
igs1592	-1,2	1,2	1,4	0,219	0,224	0,104	SMU,2033c	SMUt61	rc
igs1593	-1,0	-1,0	1,3	0,861	0,794	0,085	SMUt61	SMU,2035	fw
igs1593	-1,2	-1,2	-1,1	0,396	0,458	0,637	SMUt61	SMU,2035	rc
igs1594	-1,4	1,3	1,3	0,452	0,538	0,461	SMU,2035	SMU,2036	fw
igs1594	-1,2	1,1	-1,4	0,455	0,434	**0,032**	SMU,2035	SMU,2036	rc
igs1595	-1,1	1,2	1,2	0,670	0,274	0,222	SMU,2036	SMU,2037	fw
igs1595	-1,1	-1,2	1,3	0,280	**0,038**	**0,000**	SMU,2036	SMU,2037	rc
igs1597	-1,0	-1,2	-1,1	0,986	0,635	0,853	SMU,2038	SMU,2040	fw
igs1597	-1,1	-1,1	-1,2	0,716	0,672	0,462	SMU,2038	SMU,2040	rc
igs1598	-1,0	-1,0	-1,1	0,894	0,980	0,662	SMU,2040	SMU,2042	fw
igs1598	1,0	1,1	1,2	0,938	0,113	0,068	SMU,2040	SMU,2042	rc
igs1599	-1,1	-1,4	-1,1	0,627	0,093	0,776	SMU,2042	SMU,2043c	fw
igs1599	1,0	1,0	1,1	0,895	0,743	0,177	SMU,2042	SMU,2043c	rc
igs1601	-1,2	-1,0	-1,3	0,718	0,921	0,572	SMU,2044	SMU,2046c	fw
igs1601	-1,2	-1,1	1,1	0,062	0,494	0,152	SMU,2044	SMU,2046c	rc
igs1602	-1,4	-1,1	-1,4	0,371	0,878	0,334	SMU,2046c	SMU,2047	fw
igs1602	-1,1	-1,4	-1,2	0,055	**0,001**	**0,000**	SMU,2046c	SMU,2047	rc
igs1603	-1,2	-1,2	-1,5	0,281	0,209	**0,014**	SMU,2047	SMU,2048	fw
igs1603	-1,0	-1,1	-1,1	0,925	0,457	0,504	SMU,2047	SMU,2048	rc
igs1604	-1,2	-1,1	-1,0	0,108	0,635	0,747	SMU,2048	SMU,2049c	fw
igs1604	1,2	1,0	1,1	**0,010**	0,775	0,411	SMU,2048	SMU,2049c	rc
igs1605	-1,0	1,1	-1,2	0,825	0,582	0,215	SMU,2050c	SMU,2052c	fw
igs1605	1,2	1,5	1,0	**0,003**	**0,001**	0,751	SMU,2050c	SMU,2052c	rc
igs1607	1,0	1,0	1,0	0,881	0,975	0,964	SMU,2054c	SMU,2055	fw
igs1607	-1,2	1,0	1,2	0,344	0,917	0,361	SMU,2054c	SMU,2055	rc
igs1608	1,2	-1,1	-1,2	**0,037**	0,256	**0,022**	SMU,2056	SMUt62	fw
igs1608	1,7	1,2	1,7	0,399	0,799	0,483	SMU,2056	SMUt62	rc
igs1609	-1,5	1,4	-1,3	0,139	0,107	0,291	SMUt62	SMU,2057c	fw
igs1609	-2,1	-1,6	1,1	0,257	0,516	0,902	SMUt62	SMU,2057c	rc
igs1610	-1,0	-1,1	1,1	0,956	0,844	0,861	SMU,2057c	SMU,2058	fw
igs1610	-1,2	-1,0	-1,2	0,436	0,919	0,345	SMU,2057c	SMU,2058	rc
igs1611	-1,3	1,1	-1,0	0,140	0,505	0,809	SMU,2058	SMU,2059c	fw
igs1611	-1,2	-1,2	1,2	0,563	0,467	0,304	SMU,2058	SMU,2059c	rc
igs1612	1,1	1,6	**2,8**	0,404	**0,000**	**0,000**	SMU,2059c	SMU,2060	fw
igs1612	**2,4**	-1,1	1,1	**0,001**	0,786	0,519	SMU,2059c	SMU,2060	rc
igs1613	-1,1	-1,3	**2,1**	0,052	**0,012**	**0,001**	SMU,2060	SMU,2061	fw
igs1613	1,3	-1,2	1,1	0,055	0,282	0,344	SMU,2060	SMU,2061	rc
igs1614	1,3	-1,1	-1,1	0,216	0,689	0,604	SMU,2061	SMU,2063	fw
igs1614	1,1	-1,0	-1,0	0,835	0,874	0,909	SMU,2061	SMU,2063	rc
igs1615	-1,6	-1,2	-1,2	0,139	0,627	0,504	SMU,2063	SMU,2064c	fw
igs1615	-1,7	-1,2	-1,1	**0,000**	0,243	0,442	SMU,2063	SMU,2064c	rc
igs1616	1,2	-1,1	-1,4	0,477	0,595	0,096	SMU,2064c	SMU,2065	fw
igs1616	-1,7	1,3	-1,2	**0,000**	**0,009**	0,077	SMU,2064c	SMU,2065	rc
igs1617	-1,2	-1,1	-2,5	0,155	0,180	**0,002**	SMU,2065	SMU,2066c	fw
igs1617	1,2	-1,0	1,8	0,127	0,907	**0,009**	SMU,2065	SMU,2066c	rc
igs1618	-1,1	-1,0	-1,2	0,514	0,906	0,098	SMU,2067	SMU,2069	fw
igs1618	1,0	-1,1	-1,1	0,244	0,126	0,074	SMU,2067	SMU,2069	rc

igs1619	-1,1	1,1	-1,1	0,468	**0,028**	**0,024**	SMU,2069	SMU,2070	fw
igs1619	-1,9	1,1	1,8	**0,000**	0,340	**0,001**	SMU,2069	SMU,2070	rc
igs1620	-1,1	-1,3	-1,3	0,590	0,118	0,120	SMU,2070	SMU,2071	fw
igs1620	-1,0	1,1	1,5	0,605	0,472	**0,001**	SMU,2070	SMU,2071	rc
igs1621	-1,2	-1,3	-1,7	0,265	0,123	**0,006**	SMU,2072c	SMU,2073c	fw
igs1621	-1,0	-1,0	1,3	0,530	0,163	**0,012**	SMU,2072c	SMU,2073c	rc
igs1622	-1,8	-1,5	-1,3	0,117	0,217	0,537	SMU,2073c	SMU,2074	fw
igs1622	-1,1	1,2	1,6	0,136	**0,010**	**0,002**	SMU,2073c	SMU,2074	rc
igs1623	1,6	-1,3	1,6	0,265	0,625	0,408	SMU,2074	SMU,2075c	fw
igs1623	-1,2	-1,4	1,4	**0,017**	**0,002**	**0,002**	SMU,2074	SMU,2075c	rc
igs1624	1,3	1,3	-2,3	0,576	0,722	0,146	SMU,2075c	SMU,2076c	fw
igs1624	1,2	-2,0	1,9	**0,018**	**0,002**	**0,022**	SMU,2075c	SMU,2076c	rc
igs1625	1,2	1,8	1,2	0,271	0,079	0,515	SMU,2076c	SMU,2077c	fw
igs1625	1,9	-1,6	-2,0	**0,003**	0,081	**0,015**	SMU,2076c	SMU,2077c	rc
igs1626	-1,3	1,7	-1,3	0,641	0,387	0,725	SMU,2077c	SMU,2078c	fw
igs1626	-1,4	-2,6	-2,9	**0,000**	**0,000**	**0,000**	SMU,2077c	SMU,2078c	rc
igs1627	1,0	1,7	-1,1	0,985	0,103	0,824	SMU,2079c	SMU,2080	fw
igs1627	-1,0	-1,5	1,0	0,216	**0,004**	0,858	SMU,2079c	SMU,2080	rc
igs1629	1,2	1,8	1,2	0,185	**0,001**	0,320	SMU,2081	SMU,2083c	fw
igs1629	-1,2	-1,0	1,3	**0,002**	0,593	**0,036**	SMU,2081	SMU,2083c	rc
igs1630	-1,1	1,8	1,1	0,711	**0,043**	0,733	SMU,2083c	SMU,2084c	fw
igs1630	1,1	-1,4	1,3	0,136	**0,001**	**0,036**	SMU,2083c	SMU,2084c	rc
igs1631	1,0	**3,1**	**2,0**	0,940	**0,000**	**0,000**	SMU,2084c	SMU,2085	fw
igs1631	**2,2**	-1,0	1,8	**0,000**	0,859	**0,000**	SMU,2084c	SMU,2085	rc
igs1632	1,2	**3,0**	1,1	0,691	0,096	0,776	SMU,2085	SMU,2086	fw
igs1632	-1,7	-1,5	1,4	**0,000**	**0,000**	0,048	SMU,2085	SMU,2086	rc
igs1633	1,0	1,1	1,1	0,942	0,852	0,739	SMU,2086	SMU,2087	fw
igs1633	-1,0	-1,3	1,5	0,765	**0,000**	**0,000**	SMU,2086	SMU,2087	rc
igs1634	1,1	-1,8	1,3	0,736	0,216	0,460	SMU,2087	SMU,2088	fw
igs1634	1,2	-1,2	1,8	**0,039**	0,295	**0,000**	SMU,2087	SMU,2088	rc
igs1635	-1,2	-1,2	-1,4	0,365	0,254	0,053	SMU,2088	SMU,2089	fw
igs1635	1,2	-1,2	1,0	**0,000**	**0,048**	0,897	SMU,2088	SMU,2089	rc
igs1636	1,2	1,0	-1,1	0,514	0,881	0,577	SMU,2089	SMU,2090c	fw
igs1636	1,0	1,1	-1,2	0,986	0,091	**0,005**	SMU,2089	SMU,2090c	rc
igs1637	1,2	1,2	1,3	0,125	0,170	0,079	SMU,2090c	SMU,2091c	fw
igs1637	-1,1	-1,1	-1,1	**0,034**	0,401	0,083	SMU,2090c	SMU,2091c	rc
igs1638	-1,3	-1,1	1,4	0,218	0,529	0,116	SMU,2093	SMU,2094c	fw
igs1638	1,1	1,1	1,2	0,392	0,229	0,089	SMU,2093	SMU,2094c	rc
igs1639	1,0	1,1	-1,0	0,994	0,566	0,734	SMU,2094c	SMU,2096c	fw
igs1639	1,0	1,0	1,0	0,563	0,805	0,751	SMU,2094c	SMU,2096c	rc
igs1640	-1,0	1,0	1,2	0,666	0,794	**0,011**	SMU,2096c	SMU,2097	fw
igs1640	1,1	-1,3	1,3	0,837	0,303	0,335	SMU,2096c	SMU,2097	rc
igs1641	1,9	1,3	1,6	**0,000**	0,056	**0,000**	SMU,2097	SMU,2098	fw
igs1641	-1,2	-1,2	-1,4	0,197	0,519	0,098	SMU,2097	SMU,2098	rc
igs1642	1,1	1,2	-1,5	0,820	0,768	0,459	SMU,2098	SMU,2099c	fw
igs1642	1,4	1,2	1,4	0,498	0,725	0,383	SMU,2098	SMU,2099c	rc
igs1643	-1,5	-1,7	1,5	**0,000**	**0,005**	**0,001**	SMU,2099c	SMU,2100c	fw
igs1643	1,2	-1,7	-1,2	**0,026**	**0,041**	0,115	SMU,2099c	SMU,2100c	rc
igs1644	-1,0	1,0	-1,1	0,978	0,987	0,874	SMU,2101	SMU,2102	fw
igs1644	1,0	-1,1	-1,1	0,502	0,096	0,542	SMU,2101	SMU,2102	rc
igs1645	-1,0	-1,6	-1,2	0,920	0,165	0,618	SMU,2102	SMU,2104	fw
igs1645	1,1	1,4	-1,2	0,253	**0,001**	**0,037**	SMU,2102	SMU,2104	rc
igs1646	-1,1	-1,2	1,6	**0,008**	**0,028**	**0,005**	SMU,2104	SMU,2104a	fw
igs1646	-1,7	-1,4	-1,1	0,470	0,664	0,885	SMU,2104	SMU,2104a	rc
igs1647	1,2	-1,0	-1,1	**0,008**	0,888	0,409	SMU,2104a	SMU,2105	fw
igs1647	-1,2	-1,3	1,3	0,509	0,151	0,255	SMU,2104a	SMU,2105	rc
igs1648	-1,0	-1,1	1,8	0,775	0,714	**0,000**	SMU,2105	SMU,2106c	fw
igs1648	-1,3	-1,4	1,4	0,077	0,072	0,183	SMU,2105	SMU,2106c	rc
igs1651	-1,0	-1,3	-1,4	0,998	0,471	0,260	SMU,2108c	SMU,2109	fw
igs1651	1,6	-1,3	-1,3	**0,018**	**0,000**	0,092	SMU,2108c	SMU,2109	rc
igs1652	-1,9	-2,1	-2,1	**0,000**	**0,000**	**0,000**	SMU,2109	SMU,2111c	fw
igs1652	-1,2	-1,9	-1,5	0,265	**0,001**	**0,041**	SMU,2109	SMU,2111c	rc
igs1653	1,2	-1,2	1,3	0,684	0,751	0,654	SMU,2111c	SMU,2112	fw
igs1653	1,1	-1,2	-1,3	**0,025**	**0,036**	**0,019**	SMU,2111c	SMU,2112	rc
igs1654	-1,0	1,4	1,1	0,793	0,065	0,560	SMU,2112	SMU,2113c	fw
igs1654	-1,1	-1,1	1,1	**0,037**	0,506	0,210	SMU,2112	SMU,2113c	rc

igs1655	1,2	-1,3	1,1	0,102	**0,000**	0,434	SMU,2113c	SMU,2114c	fw
igs1655	1,0	1,0	1,3	0,755	0,913	**0,000**	SMU,2113c	SMU,2114c	rc
igs1656	1,5	-1,3	-1,1	0,296	0,462	0,668	SMU,2114c	SMU,2115	fw
igs1656	-1,3	1,2	1,4	**0,005**	0,222	**0,000**	SMU,2114c	SMU,2115	rc
igs1657	1,0	-1,0	-1,1	0,613	0,743	0,425	SMU,2115	SMU,2116	fw
igs1657	1,1	1,3	1,0	0,654	0,335	0,918	SMU,2115	SMU,2116	rc
igs1660	-1,1	-1,2	1,1	0,631	0,137	0,373	SMU,2119	SMU,2120c	fw
igs1660	-1,2	1,2	-1,2	0,753	0,751	0,832	SMU,2119	SMU,2120c	rc
igs1662	1,3	1,6	1,1	0,368	0,060	0,854	SMU,2121c	SMU,2123	fw
igs1662	-1,3	1,1	-1,1	**0,019**	0,099	0,209	SMU,2121c	SMU,2123	rc
igs1663	-1,7	-2,2	1,8	0,069	**0,000**	**0,004**	SMU,2124	SMU,2125	fw
igs1663	-1,0	-1,3	1,1	0,948	0,576	0,838	SMU,2124	SMU,2125	rc
igs1664	-1,6	-1,1	**2,4**	0,316	0,803	0,092	SMU,2125	SMU,2126c	fw
igs1664	1,0	-2,0	1,5	0,987	**0,042**	**0,013**	SMU,2125	SMU,2126c	rc
igs1665	-1,4	1,2	1,1	**0,005**	**0,014**	0,356	SMU,2126c	SMU,2127	fw
igs1665	-1,4	-1,2	-1,1	0,081	0,340	0,832	SMU,2126c	SMU,2127	rc
igs1666	1,1	-1,2	-1,3	0,574	**0,016**	**0,012**	SMU,2127	SMU,2128	fw
igs1666	-1,1	-1,4	1,4	0,890	0,353	0,336	SMU,2127	SMU,2128	rc
igs1667	1,4	1,1	-1,0	0,160	0,675	0,983	SMU,2128	SMU,2129c	fw
igs1667	-1,9	-1,2	-1,2	**0,048**	0,374	0,593	SMU,2128	SMU,2129c	rc
igs1668	1,1	-1,1	-1,1	0,736	0,745	0,762	SMU,2129c	SMU,2130	fw
igs1668	1,0	-1,3	-1,9	0,942	0,090	**0,000**	SMU,2129c	SMU,2130	rc
igs1671	1,6	-1,0	1,8	**0,000**	0,835	**0,002**	SMU,2133c	SMU,2134	fw
igs1671	1,2	-1,0	1,4	0,051	0,761	0,161	SMU,2133c	SMU,2134	rc
igs1672	**3,1**	-1,6	1,8	**0,008**	0,129	0,100	SMU,2134	SMU,2135c	fw
igs1672	1,4	1,3	-1,3	**0,000**	**0,000**	**0,000**	SMU,2134	SMU,2135c	rc
igs1673	1,1	1,2	1,0	0,398	0,336	0,921	SMU,2135c	SMU,2136c	fw
igs1673	-1,1	-1,1	1,1	0,431	0,564	0,496	SMU,2135c	SMU,2136c	rc
igs1678	1,0	-1,3	1,1	0,850	0,161	0,643	SMU,2140c	SMU,2141	fw
igs1678	1,6	-1,2	-1,1	**0,000**	**0,007**	0,269	SMU,2140c	SMU,2141	rc
igs1679	-1,5	-2,0	-1,3	0,124	0,068	0,448	SMU,2141	SMU,2142	fw
igs1679	-1,0	-1,0	-1,2	0,964	0,378	**0,008**	SMU,2141	SMU,2142	rc
igs1680	**2,3**	-1,7	-1,0	0,052	0,183	0,931	SMU,2142	SMU,2143c	fw
igs1680	-1,2	-1,4	1,3	0,050	**0,000**	**0,000**	SMU,2142	SMU,2143c	rc
igs1681	1,1	-1,1	-1,0	0,780	0,773	0,896	SMU,2143c	SMU,2146c	fw
igs1681	1,1	1,1	-1,2	0,480	0,501	0,280	SMU,2143c	SMU,2146c	rc
igs1682	1,7	-1,2	-1,1	0,277	0,634	0,901	SMU,2146c	SMU,2147c	fw
igs1682	-1,6	1,1	1,1	**0,012**	0,486	0,583	SMU,2146c	SMU,2147c	rc
igs1683	-1,0	-1,0	1,9	0,915	0,945	**0,001**	SMU,2147c	SMU,2148c	fw
igs1683	-1,3	1,2	-1,1	0,210	0,468	0,856	SMU,2147c	SMU,2148c	rc
igs1686	-1,1	-1,3	-1,1	0,944	0,817	0,901	SMU,2152c	SMU,2153c	fw
igs1686	-1,4	-1,3	1,2	0,226	**0,018**	0,401	SMU,2152c	SMU,2153c	rc
igs1688	-1,2	-1,1	-1,1	0,774	0,895	0,783	SMU,2154c	SMU,2155	fw
igs1688	1,6	1,2	**2,9**	0,095	0,445	**0,000**	SMU,2154c	SMU,2155	rc
igs1690	1,2	-1,5	-1,0	0,236	0,056	0,984	SMU,2156	SMU,2157	fw
igs1690	-1,6	-1,6	**2,2**	0,129	0,113	**0,017**	SMU,2156	SMU,2157	rc
igs1691	1,2	-1,1	-1,5	0,831	0,912	0,544	SMU,2157	SMU,2158c	fw
igs1691	-2,0	-1,4	1,7	**0,026**	0,341	0,115	SMU,2157	SMU,2158c	rc
igs1692	-1,4	1,0	-1,0	0,105	0,914	0,764	SMU,2158c	SMU,2159	fw
igs1692	1,1	-1,0	1,1	0,699	0,939	0,593	SMU,2158c	SMU,2159	rc
igs1693	1,1	**2,4**	-1,9	0,305	**0,002**	0,140	SMU,2159	SMU,2160	fw
igs1693	1,7	1,0	1,1	0,752	0,985	0,936	SMU,2159	SMU,2160	rc
igs1694	1,3	-1,2	1,1	0,690	0,736	0,904	SMU,2160	SMUt63	fw
igs1694	1,7	1,0	-1,1	0,074	0,973	0,873	SMU,2160	SMUt63	rc
igs1696	-1,2	-1,2	-1,3	0,638	0,588	0,471	SMUt64	SMUt65	fw
igs1696	-1,2	-1,5	-1,1	0,429	0,083	0,781	SMUt64	SMUt65	rc
igs1698	-1,0	-1,3	-1,3	0,997	0,072	0,156	SMU,2161c	SMU,2162c	fw
igs1698	-1,0	-1,2	1,3	0,671	0,152	**0,013**	SMU,2161c	SMU,2162c	rc
igs1699	1,7	1,2	1,2	**0,000**	0,380	0,216	SMU,2162c	SMU,2164	fw
igs1699	1,0	1,1	1,0	0,999	0,663	0,928	SMU,2162c	SMU,2164	rc
igs1700	1,1	1,0	1,2	0,126	0,793	**0,031**	SMU,2164	SMU,2165	fw
igs1700	-1,1	-1,0	1,5	0,527	0,853	**0,024**	SMU,2164	SMU,2165	rc
