# Supplementary material for: Comprehensive Transcriptome Profiles of Streptococcus mutans UA159 Map Core Streptococcal Competence Genes
Source: mSystems. 2016 Apr 12;1(2):e00038-15. doi: 10.1128/mSystems.00038-15 (PMC5069739; doi:10.1128/mSystems.00038-15)
Supplement: Table S6 [file sys002162010st6.pdf]

Table S6. Initial selection of candidate genes of the ComE and ComR regulons.

Gene ID	Mean fold change				Candidate Com E-regulon[a]	Candidate ComR-regulon[b]
	UA159 10 min	UA159 100 min	Δ*comS* 100min	Δ*sigX* 120 min Perry et al.		
SMU.150	51.8	62.4	40.9	5.3	E	
SMU.151	34.7	41.1	46.2	4.9	E	
SMU.152	22.4	36.4	42.0	5.4	E	
SMU.153	17.6	25.9	25.6	5.2	E	
SMU.423	38.8	49.5	54.3	5.8	E	
SMU.424	3.1	5.9	3.1	N	E	
SMU.426	2.9	5.5	3.6	2.5	E	
SMU.427	2.7	4.0	2.3	2.1	E	
SMU.925	3.8	10.3	6.4	2.7	E	
SMU.1902c	2.1	2.5	N	2.5	E	
SMU.1903c	12.4	16.0	24.3	5.0	E	
SMU.1904c	21.1	49.7	61.1	5.1	E	
SMU.1905c	26.4	47.8	43.8	5.1	E	
SMU.1906c	21.3	34.0	45.1	4.8	E	
SMU.1908c	16.4	57.1	35.8	4.9	E	
SMU.1909c	17.6	55.1	85.3	5.7	E	
SMU.1910c	15.5	39.2	61.6	5.6	E	
SMU.1912c	16.4	29.9	67.0	4.9	E	
SMU.1913c	13.6	29.3	49.4	5.2	E	
SMU.1914c	15.9	17.4	31.0	4.5	E	
SMU.926	N	3.7	2.2	N	E	
SMU.41	N	N	N	-3.3		
SMU.63c	N	-2.3		2.7		R
SMU.64	N	5.6		2.7		R
SMU.65	N	4.4		2.8		R
SMU.66	N	2.6		2.1		R
SMU.78	N	N	N	-2.0		
SMU.575c	N	N	-2.1	N		
SMU.799c	N	N	N	2.6		
SMU.877	N	N	N	-2.2		
SMU.878	N	N	N	-2.7		
SMU.879	N	N	N	-2.6		
SMU.880	N	N	N	-2.5		
SMU.881	N	N	N	-2.8		
SMU.882	N	N	N	-2.4		
SMU.883	N	N	N	-2.8		
SMU.887	N	N	N	-2.2		
SMU.1047c	N	N	-2.3	N		
SMU.1808c	N	N	-2.1	N		
SMU.2037	N	N	N	2.1		
SMU.2038	N	N	N	2.3		

[a] E; change in gene expression in UA 159 100 min that was also changed either in UA159 10 min or in ΔcomS 100 min. [b] R; change in gene expression in *sigX* 120 min reported by Perry et al (1) that was also changed in UA 159 100 min, but not in ΔcomS 100 min. Mean fold changes in gene expression (cut off

level of 2). N; no changes in gene expression (below cut off level of 2). Note that the transcriptome of *sigX* has no information on the direction of the transcripts, and no probes for *comS*.

References

1. **Perry, J. A., M. B. Jones, S. N. Peterson, D. G. Cvitkovitch, and C. M. Levesque.** 2009. Peptide alarmone signalling triggers an auto-active bacteriocin necessary for genetic competence. Molecular Microbiology **72:**905-17.
