# Supplementary material for: Comprehensive Transcriptome Profiles of Streptococcus mutans UA159 Map Core Streptococcal Competence Genes
Source: mSystems. 2016 Apr 12;1(2):e00038-15. doi: 10.1128/mSystems.00038-15 (PMC5069739; doi:10.1128/mSystems.00038-15)
Supplement: Table S7 [file sys002162010st7.pdf]

Table S7. Comparison of transcriptome results for late genes in *S. mutans*, *S. pneumoniae*, and *S. thermophilus*. Late genes (red) as determined for *S. mutans* UA159 in this study, and for *S. pneumoniae* Rx (1) and *S. thermophilus* LMD-9 (2). *S. pneumoniae* delayed genes are in blue. Orthologue identification and gene names as in MBGD (3).

	S. mutans	*S. pneumoniae*	*S. thermophilus*	Gene
Late genes common to all (1 to 20) or only to *S. mutans* and *S. pneumoniae* (21 to 25)				
1	SMU_325	SP_0021	STER_1804	*dut*
2	SMU_498	SP_2208	STER_0406	*comFA*
3	SMU_499	SP_2207	STER_0407	*comFC*
4	SMU_500	SP_2206	STER_0408	*raiA*
5	SMU_625	SP_0954	STER_1521	*comEA*
6	SMU_626	SP_0955	STER_1520	*comEC*
7	SMU_645	SP_0979	STER_0489	*pepB*
8	SMU_1001	SP_1266	STER_0922	*smf*
9	SMU_1055	SP_1088	STER_1430	*radC*
10	SMU_1967	SP_1908	STER_1821	*ssb2*
11	SMU_1979C	SP_2045	STER_1835	
12	SMU_1980C	SP_2047	STER_1836	
13	SMU_1981C	SP_2048	STER_1837	*comYD*
14	SMU_1982C	SP_2049	STER_1838	
15	SMU_1983	SP_2050	STER_1839	*comYD*
16	SMU_1984	SP_2051	STER_1840	*comYC*
17	SMU_1985	SP_2052	STER_1841	*comYB*
18	SMU_1987	SP_2053	STER_1842	*comYA*
19	SMU_2085	SP_1940	STER_0077	*recA*
20	SMU_2086	SP_1941	STER_0076	*cinA*
21	SMU_354	SP_1981	STER_1767	*ccs50*
22	SMU_355	SP_1980	STER_1766	*cbf*
23	SMU_539C	SP_1808	STER_0766	*pilD*
24	SMU_769	SP_0782	STER_0728	
25	SMU_837	SP_1478	STER_1326	
Late genes only in *S. mutans* (with orthologues in *S. pneumoniae*)				
1	SMU_109	SP_0379<br>SP_0145	-	*labT*
2	SMU_168	SP_1423	-	
3	SMU_326	SP_1745	-	
4	SMU_352	SP_1983	STER_1769	*rpe*
5	SMU_353	SP_1982	STER_1768	
6	SMU_356	SP_1979	STER_1765	*purR*

7	SMU_400	SP_1448	STER_1629	
8	SMU_505	SP_1431	-	*spnIM*
9	SMU_507	SP_2168	STER_0444	*fcsR*
10	SMU_646	SP_2064	-	
11	SMU_838	SP_0784	STER_0447	*gor*
12	SMU_1003	SP_0943	STER_0927	*gid*
13	SMU_1400C	SP_1786	STER_1473	
14	SMU_1916	SP_0527	STER_1650	*blpH*
15	SMU_1917	SP_2235<br>SP_0526	STER_1649	*comE*
Late genes only in *S. mutans* (without orthologues in *S. pneumoniae*)				
1	SMU_166	-	-	
2	SMU_167	-	-	
3	SMU_506	-	-	
4	SMU_508	-	PL203 ST754	
5	SMU_627	-	STER_0275	
6	SMU_772	-	-	*bglB1*
7	SMU_836 *	-	-	
8	SMU_927	-	-	
9	SMU_928	-	PL405 (STER_1115)	
10	SMU_1374	-	-	
11	SMU_1964C	-	-	
12	SMU_1965C	-	-	
13	SMU_1966C	-	-	*rbsB*
14	SMU_2076C	-	-	
Late genes in *S. pneumoniae* not induced in *S. mutans*				
1	SMU_121	SP_1939	-	*dinF*
2	SMU_328	SP_0024	STER_1800	
3	SMU_538	SP_1811	STER_1548	*trpA*
4	SMU_623C	SP_1479	PL1417 (STER_1523)	*pgdA*
5	SMU_647	SP_0980	STER_0491	
6	SMU_648	SP_0981	STER_0492	*prsA*
7	SMU_651C	SP_2197	-	
8	SMU_653C	SP_2198	-	
9	SMU_797	SP_1092	-	
10	SMU_823	SP_1074	STER_1447	
11	SMU_828	SP_0957	STER_1434	*rgpD*
12	SMU_878	SP_1897	-	*msmE*
13	SMU_1043C	SP_1100	STER_1420	*eutD*
14	SMU_1044C	SP_1099	STER_1421	
15	SMU_1045C	SP_1098	STER_1422	*ppnK*

16	SMU_1046C	SP_1097	STER_1423	
17	SMU_1048	SP_1096	STER_1424	
18	SMU_1050	SP_1095	STER_1425	*prs*
19	SMU_1051	SP_1094	STER_1426	
20	SMU_1412C<br>SMU_864	SP_2013<br>SP_0787	PL497	
21	SMU_2075C	SP_0201<br>SP_0200	STER_1941	*ccs4*
22	SMU_2112<br>SMU_1004<br>SMU_1005<br>SMU_910<br>SMU_772	SP_1937<br>SP_0667<br>SP_0965<br>SP_1573<br>SP_2201<br>SP_0377<br>SP_0378<br>SP_0391<br>SP_0069<br>SP_0930<br>SP_2136<br>SP_0117<br>SP_2190	STER_0495<br>ST62	*lytA*
Late genes only in *S. pneumoniae* (with no orthologues in *S. mutans*)				
1	-	SP_0022	-	
2	-	SP_0025	PL815	
3	-	SP_0026	-	
4	-	SP_0029	-	
5	-	SP_0030	-	
6	-	SP_0031<br>SP_1480	-	
7	-	SP_0124	-	
8	-	SP_0125	-	
9	-	SP_0956	-	
10	-	SP_0958	-	
11	-	SP_1065	PL1816	
12	-	SP_1264	-	
13	-	SP_1809	-	
14	-	SP_1810	-	
15	-	SP_2016	-	
16	-	SP_2017	-	
17	-	SP_2196	-	
18	-	SP_2199	-	
19	-	SP_2200	-	
Delayed genes in *S. pneumoniae*				

1	SMU_80	SP_0515	STER_0161	*hrcA*
2	SMU_81	SP_0516	STER_0162	*grpE*
3	SMU_82	SP_0517	STER_0163	*dnaK*
4	SMU_83	SP_0519	STER_0164	*dnaJ*
5	SMU_788	SP_1029	STER_0702	*rumA-2*
6	SMU_862	SP_0785	STER_0563	*acrA*
7	SMU_863	SP_0786	-	*salX*
8	SMU_864<br>SMU_1412C	SP_0787<br>SP_2013	PL495	
9	SMU_864	SP_0787	PL496	
10	SMU_956	SP_0338	STER_1578	*clpL*
11	SMU_1042	SP_1380	STER_1677	
12	SMU_1111C	SP_1027	STER_1253	
13	SMU_1128	SP_0799	PL758	*ciaH*
14	SMU_1129	SP_0798	PL755<br>PS1040	*ciaR*
15	SMU_1193	SP_1714	STER_1402	
16	SMU_1194	SP_1715	STER_1403	
17	SMU_1195	SP_1715	STER_1404	
18	SMU_1954	SP_1906	STER_0253	*groEL*
19	SMU_1955	SP_1907	STER_0252	*groES*
20	SMU_2164	SP_2239	STER_2002	*htrA*
21	SMU_2165	SP_2240	STER_2003	*spo0J*

-, no orthologue.

References

1. **Peterson, S. N., C. K. Sung, R. Cline, B. V. Desai, E. C. Snesrud, P. Luo, J. Walling, H. Li, M. Mintz, G. Tsegaye, P. C. Burr, Y. Do, S. Ahn, J. Gilbert, R. D. Fleischmann, and D. A. Morrison.** 2004. Identification of competence pheromone responsive genes in *Streptococcus pneumoniae* by use of DNA microarrays. Mol Microbiol **51:**1051-70.
2. **Burghout, P., H. J. Bootsma, T. G. Kloosterman, J. J. Bijlsma, C. E. de Jongh, O. P. Kuipers, and P. W. Hermans.** 2007. Search for genes essential for pneumococcal transformation: the RADA DNA repair protein plays a role in genomic recombination of donor DNA. J Bacteriol **189:**6540-50.
3. **Uchiyama, I., M. Mihara, H. Nishide, and H. Chiba.** 2015. MBGD update 2015: microbial genome database for flexible ortholog analysis utilizing a diverse set of genomic data. Nucleic Acids Res **43:**D270-6.
